# The quantity and quality of scientific evidence about the health of working women in occupational health of Japan: A scoping review

**DOI:** 10.1002/1348-9585.12427

**Published:** 2023-10-16

**Authors:** Kyoko Nomura, Kyoko Kitagawa, Mayumi Tsuji, Miho Iida, Mizuki Aoki, Kasane Miyauchi, Junko Hirayama, Kengo Nagashima, Toru Takebayashi, Akizumi Tsutsumi

**Affiliations:** ^1^ Department of Environmental Health Science and Public Health Akita University Graduate School of Medicine Akita Japan; ^2^ Department of Environmental Health University of Occupational and Environmental Health Kitakyushu Japan; ^3^ Division of Ultrastructural Cell Biology, Department of Anatomy University of Miyazaki Miyazaki Japan; ^4^ Department of Preventive Medicine and Public Health Keio University School of Medicine Tokyo Japan; ^5^ Biostatistics Unit, Clinical and Translational Research Center Keio University Hospital Tokyo Japan; ^6^ Department of Public Health Kitasato University School of Medicine Sagamihara Japan

**Keywords:** health promotion, Ichu‐shi, Japan, PubMed, scoping review, working women

## Abstract

**Objective:**

We aim to investigate the quantity and quality of scientific evidence dealing with comprehensive health issues of working women in occupational health.

**Methods:**

This scoping review of original articles that investigated comprehensive health issues of working women aged 19–64 years in Japan was published in PubMed (1967–2022) and Igaku Chuo Zasshi (or Ichu‐shi, 1982–2022). Using identical broad search terms, we first identified 17 122 English and 6154 Japanese articles. We excluded those with clinically relevant topics, or ethnicity other than Japanese and included 853 English and 855 Japanese articles for review and classified them into nine research areas considered to be critical factors for women in the workforce and five study design groups to investigate the quality of the evidence accumulated.

**Results:**

Among 853 English‐language articles in PubMed, “Mental health” was the most frequent area studied, followed by “Work‐related disease” and “Lifestyle‐related disease.” Among 855 Japanese‐language articles from Ichu‐shi, “Mental health” was the most frequently studied area followed by “Work and balance,” and “Work‐related disease.” “Infertility, pregnancy, and childbirth” and “Menstruation, menopause, and genital disease” were well studied in Ichu‐shi but scarcely published in PubMed. “Harassment and discrimination” were sparsely reported in both databases. As for research designs, many articles in both PubMed and Ichu‐shi employed descriptive or cross‐sectional study designs. However, a few studies employed cohort/longitudinal or interventional studies.

**Conclusion:**

The results underscored the need for higher‐quality study designs with more scientific evidence on working women's health in the field of occupational health.

## 1 INTRODUCTION

According to the 2022 Labor Force Survey,^1^ there are 69.11 million workers in Japan, of which 31.08 million are women. A unique feature of Japan's women workforce is that more than half (56%) of them work as part‐timers.^1^ This is because stereotypes of gender roles are strongly rooted in the public mindset, regardless of gender,^2^ women are less likely than men to participate in the labor market, and as a result, workplaces have been created to make it easier for men to work and harder for women to work.^3^ For example, according to a survey conducted by the Ministry of Economy, Trade, and Industry (METI), menstrual leave was the most common form of health promotion support (21%), followed by cancer screening opportunity (13%), whereas paid leave for medical treatment of cancer or infertility was only 5% and 4%, respectively. Thus, many significant health issues for working women remain unaddressed.^4^

However, Japan is currently an aging nation with a low birthrate, and there is a national need for women to actively participate in the labor market. In 2015, the government established a law to encourage working women (Law for the Promotion of Women's Activities) and positioned it as one of the pillars of its economic growth strategy.^5^ In 2014, the METI announced the concept of “health management,” the idea that investment in the health of workers contributes to corporate growth.^6^ Furthermore, in 2017, in the large company sector, women's health management and promotion was included in the requirements for the “Excellent Health Management Corporation” criteria; from 2021, the health management and promotion of women in the workplace are required in this criteria, regardless of company size. However, as we noted above, the current working environment does not give sufficient consideration for women to active participation in work.

The purpose of the Law for the Promotion of Women's Activities^5^ includes supporting women's careers and balancing work and gender role division of labor, such as childcare and nursing care, while the health issues of working‐age women include reproductive health (sexual and reproductive health), such as breast and cervical cancer screening,^7^ pregnancy and childbirth,^8^ work‐related illnesses requiring consideration from a biological perspective,^9^ and mental health.^10^ In addition, the aforementioned criteria for determining an excellent health management corporation do not include specific guidelines for supporting women in the workplace. Therefore, the purpose of this study is to conduct a scoping review of studies related to health issues of working women in occupational health field to determine the extent to which scientific evidence of basic reference of comprehensive support for women in active participation in work has been studied and accumulated in Japan.

## 2 METHODS

### 2.1 Study design

We conducted a scoping review of original articles that investigated health issues of working women aged 19–64 years in Japan and published in PubMed from 1967 to May 16, 2022, and Ichushi‐Web (Japanese‐language medical literature database, Ichu‐shi, hereafter) from 1982 to August 31, 2022. The “Ichushi‐Web” containing more than 13 million data was built by the Japan Medical Abstract Society (JAMAS), which accumulated medical literature information published in Japan since 1903. We selected these two databases because our purpose was to find out if there is any difference between papers of health issues among working Japanese women published in Japanese and in English in terms of study areas, number, and quality. Inclusion criteria included studies that (1) were original, (2) investigated any health issues of working women, and (3) targeted Japanese working women aged 19–64 years, such as the working population. Exclusion criteria included studies that (1) were published as commentary/letter/bulletin/proceeding/conference or meeting or case reports/protocol; (2) were not relevant to work; (3) focused on clinical issues including clinical trials and clinical experiments; (4) contained physical or animal experiments; (5) focused on infant/children and students; (6) focused on older populations; and (7) took place in foreign settings, including an international comparison studies. The protocol of this review was registered in UMIN (No. 000051075).

### 2.2 Search terms

The search terms we used in PubMed were “female* (the asterisk represents any group of characters),” “woman/women,” “work*,” “employ*,” and “Japan*” with Medical Subject Headings (MESH), and all fields as search areas and then limited to “human” and “adults aged 19 years or older.” Our search strategy for Ichu‐shi included the use of the thesaurus of medical terms, which is a collection of keywords systematically related to terms used in the fields of medicine, such as dentistry, pharmacy, nursing, veterinary medicine, and public health, created by JAMAS.^11^ The thesaurus we used was “work,” including “labor,” “employed,” “job,” “salaried,” “female,” or “women” in Japanese. Our search history for PubMed and Ichu‐shi is shown in Tables 1 and 2, respectively.

**TABLE 1:** Search history by PubMed (May 16, 2022).

#	Query	Filters	Search Details	Results
1	employed		“employ”[All Fields] OR “employing”[All Fields] OR “employment”[MeSH Terms] OR “employment”[All Fields] OR “employed”[All Fields] OR “employs”[All Fields]	747 437
2	work*		“work*”[All Fields]	2 248 084
3	(female) OR (women)		“female”[All Fields] OR “female”[MeSH Terms] OR “female”[All Fields] OR “females”[All Fields] OR “female s”[All Fields] OR “females”[All Fields] OR “woman”[All Fields] OR “women”[MeSH Terms] OR “women”[All Fields] OR “woman”[All Fields] OR “women s”[All Fields] OR “women”[All Fields]	9 974 047
4	(#2) OR (#1)		“work*”[All Fields] OR “employ”[All Fields] OR “employing”[All Fields] OR “employment”[MeSH Terms] OR “employment”[All Fields] OR “employed”[All Fields] OR “employs”[All Fields]	2 824 753
5	(#3) AND (#4)		(“female”[All Fields] OR “female”[MeSH Terms] OR “female”[All Fields] OR “females”[All Fields] OR “female s”[All Fields] OR “females”[All Fields] OR (“woman”[All Fields] OR “women”[MeSH Terms] OR “women”[All Fields] OR “woman”[All Fields] OR “women s”[All Fields] OR “women”[All Fields])) AND (“work*”[All Fields] OR (“employ”[All Fields] OR “employing”[All Fields] OR “employment”[MeSH Terms] OR “employment”[All Fields] OR “employed”[All Fields] OR “employs”[All Fields]))	752 567
6	Japan*		“japan*”[All Fields]	1 854 416
7	(#5) AND (#6)		(“female”[All Fields] OR “female”[MeSH Terms] OR “female”[All Fields] OR “females”[All Fields] OR “female s”[All Fields] OR “females”[All Fields] OR (“woman”[All Fields] OR “women”[MeSH Terms] OR “women”[All Fields] OR “woman”[All Fields] OR “women s”[All Fields] OR “women”[All Fields])) AND (“work*”[All Fields] OR (“employ”[All Fields] OR “employing”[All Fields] OR “employment”[MeSH Terms] OR “employment”[All Fields] OR “employed”[All Fields] OR “employs”[All Fields])) AND “japan*”[All Fields]	26 032
8	(#5) AND (#6)	Humans	((“female”[All Fields] OR “female”[MeSH Terms] OR “female”[All Fields] OR “females”[All Fields] OR “female s”[All Fields] OR “females”[All Fields] OR (“woman”[All Fields] OR “women”[MeSH Terms] OR “women”[All Fields] OR “woman”[All Fields] OR “women s”[All Fields] OR “women”[All Fields])) AND (“work*”[All Fields] OR (“employ”[All Fields] OR “employing”[All Fields] OR “employment”[MeSH Terms] OR “employment”[All Fields] OR “employed”[All Fields] OR “employs”[All Fields])) AND “japan*”[All Fields]) AND (humans[Filter])	21 819
9	(#5) AND (#6)	Humans, Adult: 19+ years	((“female”[All Fields] OR “female”[MeSH Terms] OR “female”[All Fields] OR “females”[All Fields] OR “female s”[All Fields] OR “females”[All Fields] OR (“woman”[All Fields] OR “women”[MeSH Terms] OR “women”[All Fields] OR “woman”[All Fields] OR “women s”[All Fields] OR “women”[All Fields])) AND (“work*”[All Fields] OR (“employ”[All Fields] OR “employing”[All Fields] OR “employment”[MeSH Terms] OR “employment”[All Fields] OR “employed”[All Fields] OR “employs”[All Fields])) AND “japan*”[All Fields]) AND ((humans[Filter]) AND (all adult[Filter]))	17 122

**TABLE 2:** Search history by Ichu‐shi (2022/8/31).

#	Search Word	# of reference
1	労働/TH or 労働/TI or 労働/AB	[61580件]
2	雇用/TH or 雇用/TI or 雇用/AB	[36158件]
3	就労/TI or 就労/AB	[10963件]
4	勤務/TI or 勤務/AB	[23649件]
5	勤労/TI or 勤労/AB	[3224件]
6	仕事/TI or 仕事/AB	[19051件]
7	就業/TI or 就業/AB	[4156件]
8	有職/TI or 有職/AB	[383件]
9	女性/TH or 女性/TI or 女性/AB	[274367件]
10	就労女性/TH or 就労女性/TI or 就労女性/AB	[4140件]
11	#1 or #2 or #3 or #4 or #5 or #6 or #7 or #8	[127765件]
12	#11 and #9	[11206件]
13	#12 or #10	[12848件]
14	(#13) and ((PT = 症例報告・事例除く) AND (PT = 原著論文))	[6154件]

### 2.3 Identification of relevant articles

**FIGURE 1:**
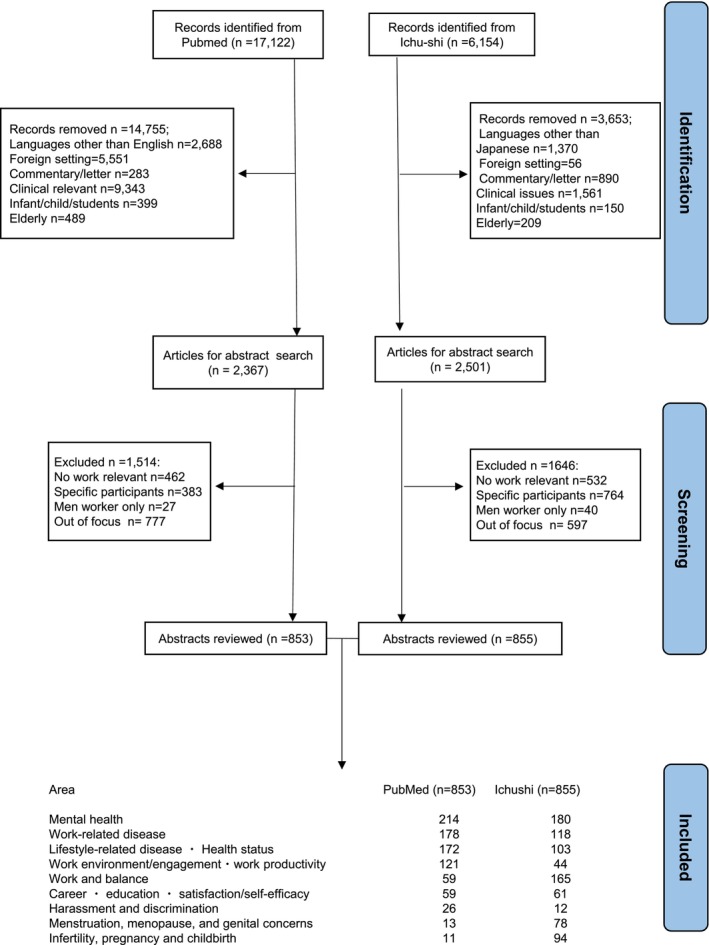
PRISMA flowchart diagram.

Figure 1 shows the PRISMA flow diagram. The database search with the above search words yielded 17 122 English‐language articles and 6154 Japanese‐language articles. In the PubMed search, we performed a title search and excluded when any of the following conditions were met: languages other than English (*n* = 2688), foreign settings (*n* = 5551), commentary/letter (*n* = 283), clinical studies (*n* = 9343), infants/children/students (*n* = 399), and older adults (*n* = 489). Next, we read the abstract of the remaining 2367 and excluded 1511 articles that had no relevance to work (*n* = 462), specific participants (*n* = 383), men workers only (*n* = 27), and out‐of‐scope (*n* = 777). Specific participants included those who experienced disasters had infectious diseases, such as human immunodeficiency virus, coronavirus, influenza, end‐of‐life caregivers, women after retirement, medical personnel whose work is very specialized, for example, bereaved caregivers, a nurse with organ transplantation, first‐year medical doctors and nurses, and in a minimal area of specialty, including nurses working at a psychiatric ward. Finally, we included 853 studies in English in the scoping review. In Ichu‐shi, we performed a title search and excluded studies in languages other than Japanese (*n* = 1370), when the study setting was not Japan or the study participants were not Japanese (*n* = 56), a commentary/letter/bulletin/proceeding/conference or meeting reports/protocol (*n* = 890), investigated “patients” and clinically relevant issues (*n* = 1561), infants/children/students (*n* = 150), or elderly (*n* = 209). Among the remaining 2501 articles, we retrieved abstracts and excluded those not relevant to work (*n* = 532), specific participants (*n* = 764), men‐only (*n* = 40), or out‐of‐focus (*n* = 597). Finally, we included 855 Japanese‐language articles for a scoping review. Three investigators (KN, MA, and KM) independently searched the literature and any discrepancy were discussed with the team members.

### 2.4 Nine research areas of eligible articles

The nine areas were developed with reference to 38 items from the “Checklist for Creating Healthy Workplaces for Men and Women Workers” developed by the working women's health in 2008^12^ and 21 items from the “Survey on Health Promotion for Working Women” conducted by the Ministry of Economy, Trade and Industry in 2017^4^ to support and care for women. The nine areas aimed to clarify which areas of occupational health are lacking and what kind of research needs to be accumulated in the future and were determined by the result of exchanging opinions with experts and academic members at the 94th Congress and symposium of the Japan Society for Occupational Health.^13^ We described the short description of each area as follows:

1. *Mental health:*
  This area covers mental health of workers, including biomarkers, such as salivary cortisol, occupational stress of job and industry, lifestyles of smoking, drinking, physical activity, and mental health, overwork associated mental ill‐health, scale development of mental health at the workplace, help‐seeking behavior, and stress management of workers.
2. *Work‐related disease:*
  This area covers occupation‐related health problems, including muscle and skeletal disorders; occupational cancer; visual display terminals (VDTs); substance exposure, such as chemical substances and organic solvents, polychlorinated biphenyls, asbestos, lead, metal, silica, cadmium, copper, and zinc; physical symptoms associated with work, including asthma, eczema, and respiratory symptoms; health problem associated with shift work; the work environment, including heat, cold, and noise; safety hazards and work‐related injury.
3. *Lifestyle‐related disease/health status*:
  This area covers diet and nutrition, lifestyle including smoking and alcohol, lifestyle‐related diseases including metabolic syndrome, diabetes, hypertension, dyslipidemia, cerebrocardiovascular disease, oral health, physical activity, sarcopenia/locomotive syndrome, and frailty.
4. *Work environment/engagement・work productivity*:
  This area investigated work environment/engagement, retention, presenteeism and absenteeism, and organizational environment including workplace social capital.
5. *Work and balance, such as gender responsibility or disease*:
  This area covers childrearing, family responsibility, gender division of labor, work–life balance/work–family conflict including informal home‐caregiver, workstyles (full‐time/part‐time labor), and work and disease balance including return to work.
6. *Career/education/satisfaction/self‐efficacy*:
  This area covers challenges regarding career development, including full‐time or part‐time work, career discontinuation, rework, and self‐efficacy/satisfaction, and education for professional development or health promotion.
7. *Harassment and discrimination*:
  It has been established that women in the workplace are vulnerable to harassment, bullying, prejudice, or gender discrimination.^14^
8. *Menstruation, menopause, and genital concerns*:
  As the previous METI survey highlighted that efforts regarding women's health have been delayed at the workplace,^4^ this area covers sex‐related health issues at workplace, such as premenstrual syndrome and menopause, and reproductive organs referring to the increased incidence of reproductive cancer.
9. *Infertility, pregnancy, and childbirth*:
  This area covers maternal and perinatal health issues of working women including pregnancy outcomes, maternal and parental leaves, and infertility treatment while working.

Considering the aforementioned nine important research areas, we assigned each study to a particular category based on the focus of that study. For example, we assigned #7 to a study that investigated the effect of the night shift on any health outcomes. However, if a study investigated a relationship between breastfeeding self‐efficacy, and postpartum depression or work‐shifts on pregnancy outcome, we assigned them to #2 instead of #9 and #7, respectively, owing to our focus on issues pertaining to reproductive health.

### 2.5 Study design of eligible studies

Based on their design, studies were categorized into five groups: (1) Descriptive/Cross‐sectional; (2) Case–control; (3) Cohort/Longitudinal; (4) Intervention; (5) Qualitative/Mixed‐method studies.

## 3 RESULTS

### 3.1 Comparison of articles in nine research areas between two databases (Table 3)

**TABLE 3:** Comparison of study design of articles between PubMed and Ichu‐shi databases.

Areas	Descriptive/Cross‐sectional		Case–control		Cohort/Longitudinal		Intervention		Qualitative/Mixed method	
	PubMed	Ichu‐shi	PubMed	Ichu‐shi	PubMed	Ichu‐shi	PubMed	Ichu‐shi	PubMed	Ichu‐shi
Mental health	161	170	1	0	30	4	22	6	0	0
Work‐related disease	146	102	16	1	9	4	7	10	0	1
Lifestyle‐related disease/health status	126	94	7	1	29	3	10	4	0	1
Work environment/engagement・organizational work productivity	92	38	1	0	18	1	10	0	0	5
Work and balance	46	145	0	0	12	3	1	0	0	17
Career /education/satisfaction/self‐efficacy	49	54	0	0	2	3	6	3	2	1
Harassment and discrimination	26	10	0	0	0	0	0	0	0	2
Menstruation, menopause, and genital concerns	10	73	0	0	1	1	1	1	1	3
Infertility, pregnancy, and childbirth	9	79	0	2	2	4	0	1	0	8
Rate occupied	78.04%	89.47%	2.92%	0.47%	12.03%	2.69%	6.66%	2.92%	0.35%	4.44%

**FIGURE 2:**
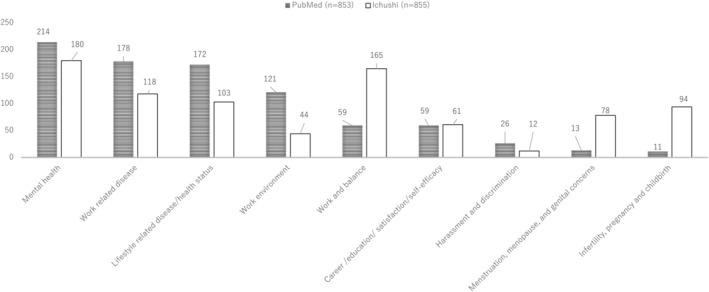
Comparison of the numbers of articles published in PubMed and Ichu‐shi by nine research areas.

Among 853 English‐language articles in PubMed (Table S1), “Mental health” (25%) was the most frequent area studied, followed by “Work‐related disease” (21%) and “Lifestyle‐related disease” (20%; Figure 2). Among 855 Japanese‐language articles from Ichu‐shi (Table S2), “Mental health” was the most frequently studied (21%) followed by “Work and balance” (19%), and “Work‐related disease” (14%). Studies on “Infertility, pregnancy, and childbirth” and “Menstruation, menopause, and genital disease” were prevalent in Ichu‐shi (11% and 9%, respectively) but scarce in PubMed (both 1%; Figure 2). “Harassment and discrimination” were sparsely reported in both databases (3% in PubMed and 1% in Ichu‐shi).

**FIGURE 3:**
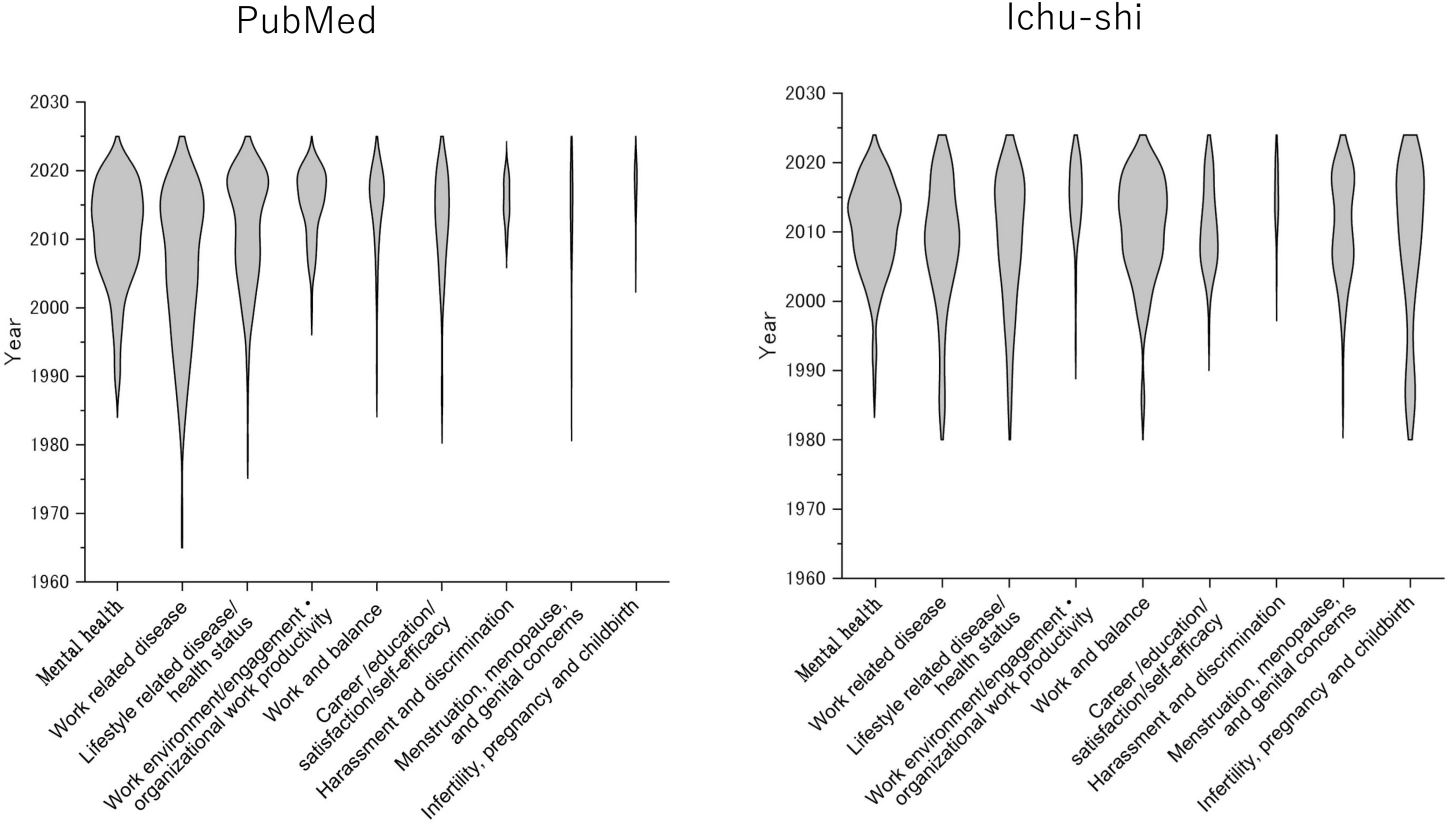
Publication year of English‐ and Japanese‐language articles that investigated health promotion of working women in Japan.

Studies in Japanese on “Infertility, pregnancy, and childbirth,” “Lifestyle‐related disease/status,” and “Work‐related disease” have long been conducted, starting in the 1980s, whereas “Work environment/engagement・work productivity” have only emerged as recent as 2005 (Figure 3). However, English articles began to be published on “Lifestyle‐related disease,” “Work‐related disease,” and “Mental health” before 2000, but most of the articles in the other research areas were published in 2005 or later (Figure 3).

As for study design, a large number of studies, both in PubMed and Ichu‐shi, employed descriptive or cross‐sectional study designs (78% and 90%, respectively; Table 3). There were fewer qualitative studies among English‐language articles as compared to those in Japanese (0.4% and 4.4%, respectively; Table 3). Most (83%) of the qualitative studies in Japanese were published after 2010, whereas the oldest publication was in 2003 with work and life balance (43%) as the most studied area.

### 3.2 Mental health

Mental health was the most frequently studied in both databases with 214 English‐language articles that investigated occupational stress (*n* = 122), lifestyle, such as alcohol and smoking, and mental health (*n* = 24), stress management like coping and resilience (*n* = 37), overwork on psychological distress (*n* = 21), and scale development (*n* = 10). A previous study, with a large sample size of 2220 employees, demonstrated that a high risk of depression was associated with being a woman and having short and/or prolonged sleep durations.^13^ A study with 16 444 men and 3078 women employees demonstrated that women workers may have a more significant occupational class gradient in job strain and job insecurity than men.^15^ Another study also demonstrated that high occupational stresses were considerably associated with insomnia in women.^14^ A study of 4722 employees aged 20–59 years reported that the prevalence rates of excessive daytime sleepiness were 13.3% for women and 7.2% for men.^16^ In addition, 180 studies in Japanese investigated occupational stress (*n* = 138), overwork and mental health (*n* = 5), stress management (*n* = 29), scale development (*n* = 4), and others (*n* = 4).

### 3.3 Work‐related disease

There were 178 studies in English with a focus on musculoskeletal disorder (*n* = 39); shift work (*n* = 32); substance exposure, including specified chemical substance and organic solvent (*n* = 32); comfortable workplace (e.g., heat, humidity, noise, gas, and sick house [*n* = 14]); PCB, asbestos, lead, metal, silica, cadmium, copper, and zinc (*n* = 21); and safety and work‐related injury (*n* = 16); occupational allergic reaction (*n* = 12); and cancer (*n* = 4). The rest included posture, vibration, and VDT (*n* = 8). Studies investigating cancer occurrence at workplace were about cholangiocarcinoma in a printing company.^17^, ^18^ The rest explored nasal cancer among woodworkers^19^ and lung cancer in indium‐exposed workers.^20^ One study based on 1249 women nurses working night shifts reported that 30% had irregular menstrual cycles.^21^

In contrast, 118 studies in Japanese explored occupation‐based diseases such as musculoskeletal disorder (*n* = 53); shift work (*n* = 34); comfortable workplace such as cold, heat, and noise (*n* = 7); specified chemical substance and organic solvent (*n* = 7); and work‐related injury (*n* = 4). The rest included vibration, VDT, and miscellaneous topics (*n* = 13).

### 3.4 Lifestyle‐related disease/status

A total of 172 studies in English investigated diet and nutrition (*n* = 21), lifestyle, including smoking (*n* = 31), alcohol (*n* = 10), obesity (*n* = 8), and others (*n* = 6); lifestyle‐related diseases including cerebrovascular risk (*n* = 21), glucose metabolism (*n* = 8), metabolic syndrome (*n* = 14), lipidemia (*n* = 4), and hypertension (*n* = 7); oral health (*n* = 11); physical activity including sedentary position (*n* = 7); and others (*n* = 9). The remaining investigated miscellaneous topics (*n* = 15), including bowel movement, uric acid, non‐alcoholic fatty liver disease, hypotension, snoring headache, chronic kidney disease, the lifecycle of sleeping, and cancer (*n* = 10). The use of mobile applications as a tool for behavioral change specific to working women is yet to be explored. Nevertheless, one study based on data from the two national databases and a commercial app database assessed smartphone‐recorded steps over the years and reported a long‐term beneficial change in physical activity, suggesting a powerful tool for cardiovascular disease prevention.^22^

A total of 103 studies in Japanese investigated diet and nutrition (*n* = 20); lifestyle including alcohol (*n* = 5), smoking (*n* = 12), physical activity (*n* = 3), mental health (*n* = 4), and others (*n* = 14); lifestyle‐related disease (*n* = 12); oral health (*n* = 8); and physical activity, including sedentary work (*n* = 4) and others (*n* = 16). The rest investigated miscellaneous topics (*n* = 5). Among the three cohort studies, one demonstrated blood pressure elevation in women who worked ≥10 h per day over a 7‐year follow‐up period.^23^ There were three interventional studies, including one interventional study on weight reduction without control,^24^ one RCT of diet program with a picture attached to an email,^25^ and one cluster RCT of salt reduction at the workplace.^26^

### 3.5 Work environment/engagement・work productivity

A total of 121 English‐language articles investigated work productivity (*n* = 48), work engagement (*n* = 23), organizational climate (*n* = 8), workplace support (*n* = 22), and retention (*n* = 20). There were 10 interventional studies that investigated work productivity, performance, engagement, environment, and improvement: Among these, four investigated work productivity with job stress education for supervisors,^27^ telephone cognitive–behavioral therapy,^28^ an Active Rest Program by Workplace Units,^29^ and pain neuroscience education and exercise;^30^ two investigated work performance with cognitive–behavioral therapy^31^ and oral health behavior^32^; three demonstrated a positive effect of cognitive–behavioral therapy,^33^ a job crafting intervention program,^34^ and web‐based stress literacy intervention^35^ on work engagement; and two demonstrated work environment improvement with a work participation program.^36^ Forty‐four Japanese‐language articles investigated retention (*n* = 23), work engagement/commitment (*n* = 16), and work productivity, including presenteeism/absenteeism (*n* = 5).

### 3.6 Work and balance, such as gender responsibility or disease

Fifty‐nine studies in English investigated return to work (*n* = 18), work–family conflict, including childrearing (*n* = 22), work‐illness (cancer except for reproductive cancer) balance (*n* = 4), informal caregiving (*n* = 9), and working style (*n* = 6). As for gender roles, two nationwide survey‐based studies demonstrated that family caregiving negatively affects women employment.^37^, ^38^ Prolonged informal caregiving increased the risk of psychological distress^37^ and working women who began providing five or more hours of care per week were considerably more likely to leave their jobs than non‐caregiving women.^38^ As for work and health balance, a study of 1478 cancer survivors demonstrated that women were more likely to lose their jobs than men, and temporary employees were more likely to lose their jobs than regular employees.^39^ In contrast, 165 studies in Japanese investigated workstyle (*n* = 43), rework from childrearing, or health issues (*n* = 13), work–family conflict or work–life balance (*n* = 31), work and childrearing (*n* = 55), and burden of a caregiver for family members, such as taking care of older adults at home (*n* = 23).

### 3.7 Career/education/satisfaction/self‐efficacy

Fifty‐nine studies in English investigated satisfaction (*n* = 32), career (*n* = 6), education (*n* = 15), and self‐efficacy (*n* = 6). Sixty‐one studies in Japanese investigated satisfaction (*n* = 16), career (*n* = 25), education (*n* = 14), and self‐efficacy (*n* = 6). There were 25 English articles and 15 Japanese articles that investigated medical personnel, including nurses and physicians.

### 3.8 Harassment and discrimination

Twenty‐six studies in English and 12 in Japanese investigated harassment, discrimination, stigma, bullying, aggression, mistreatment, and violence. Three articles in the Japanese language studied maternity harassment.^40^, ^41^, ^42^ One article in the English‐language investigated gender, sexual, and academic harassment among university faculties and demonstrated that women who have seen or heard of someone who experienced harassment had higher burnout scores even though they were not direct victims of any harassment.^43^ A longitudinal study also revealed that division‐level workplace bullying was associated with increased individual‐level psychological distress in the public sector with 3142 employees.^44^ These results suggest that harassment or bullying in the workplace can have a detrimental effect on employees' mental health even if they are not personally and directly victimized.

### 3.9 Menstruation, menopause, and genital disease

There were only 13 studies in English that investigated breast cancer (*n* = 4),^45^, ^46^, ^47^, ^48^ cervical cancer (*n* = 2),^49^, ^50^ menstruation (*n* = 3),^51^, ^52^, ^53^ balance between work and reproductive cancer (*n* = 3),^54^, ^55^, ^56^ and bladder symptom (*n* = 1).^57^ There was only one randomized control trial (RCT) on using mobile applications for menstrual management, which decreased the incidences of depression but had no effect on work productivity.^53^ A study of 105 breast cancer survivors reported that one‐third lost their jobs, and 47.6% reported a decrease in personal income after diagnosis. Furthermore, contract or part‐time workers were more likely to lose their jobs compared to regular, full‐time workers.^46^ Furthermore, there were three studies that investigated a balance between work and cancer treatment.^54^, ^55^, ^56^ One study reported that nearly half of 145 women hospital workers experienced lower urinary tract symptoms, including urinary incontinence.^57^

In contrast, 78 studies in Japanese investigated menstruation (*n* = 25), menopause (*n* = 26), reproductive cancer (*n* = 17), urinary incontinent (*n* = 4), hormone therapy indication (*n* = 4), and uterine myoma (*n* = 2; Table S2). Most studies on reproductive cancer investigated screening participation rates, and only one qualitative study investigated workers who required cancer treatment.^58^ There was only one interventional study to assess breast self‐care programs for early cancer detection, but the intervention was based on pre‐ and post‐test without control.^59^

### 3.10 Infertility, pregnancy, and childbirth

There were only 11 English‐language articles^60^, ^61^, ^62^, ^63^, ^64^, ^65^, ^66^, ^67^, ^68^, ^69^, ^70^ that investigated pregnant women with alcohol consumption,^63^, ^68^ smoking,^66^ psychological stress,^62^ depression,^61^, ^65^ and positive emotion^69^ during pregnancy, and long working hours and shift work on pregnancy outcome,^64^, ^67^ trends in fertility by income and education,^60^ and a desire to have an additional child of dual‐earner couples.^70^ However, there were 94 Japanese‐language studies that investigated perinatal women related to balance with work (*n* = 17), breastfeeding (*n* = 10), infertility (*n* = 11), mental health (*n* = 13), pregnancy outcome (*n* = 3), and quality of life of pregnant women (*n* = 3); safety, work regulation, and perinatal leave (*n* = 17); statistics of single and birthrate with working styles (*n* = 2); and work, nutrition, and lifestyle (*n* = 18). There was only one which employed interventional study design^71^ that evaluated the preconception health education program with a 3‐month interval, but the assessment was based on pre‐ and post‐test without control.

## 4 DISCUSSION

In this scoping review of articles investigating health issued of working women in occupational health of Japan, we found that Japanese and English‐language articles are both dominated by mental health but disproportionately distributed among the remaining eight key areas. We also found that the majority of both Japanese and English studies employed descriptive or cross‐sectional study designs, and very few employed a cohort/longitudinal study or interventional research designs that have more vital evidence for quality. In addition, articles published in PubMed compared to Ichu‐shi used more intervention and longitudinal study designs.

The area of work productivity is a relatively new area of research since year 2000. Our review identified the positive effect of workplace social capital, communication, and consultation on work engagement and productivity,^72^, ^73^, ^74^ but there are still few studies with women perspective. For example, previously it is known that many young women have at least one symptom of premenstrual syndrome^75^ and 80% of middle‐aged women (45–56 years) have at least one of menopause‐related symptoms.^76^ Among the general population, 5% have premenstrual dysphoric disorder,^77^ which significantly influences work engagement, particularly presenteeism rather than absenteeism.^78^ Thus, future research should be conducted to analyze this important topic with work productivity.

As for balance between work and gender responsibility, we identified that family caregiving negatively affects women's employment.^37^, ^38^, ^39^ The strong gender division of labor is embedded into the mindset of Japanese people, and thus, Japanese women have long struggled with family responsibilities, including childrearing and care of older adults. One study demonstrated that single women with lower education levels were likely to be primary family caregivers.^79^ This means that women with less educational opportunity may be more likely to be family caregivers, indicating a person sacrifices their career development and incurs subsequent financial strain by working reduced hours, creating a vicious cycle. In addition, in Japan, the spouse is privileged to have tax exemption if his/her annual income is less than JPY 480 000. Such laws may further hamper any opportunity for career development. This area should be discussed within working style reform with country, municipality, company, and individual workers.

Work‐related diseases are well studied in women with muscle‐skeletal disorders. In Japan, women workers are more dominant in the health and welfare industry, followed by retail and wholesale, where low back pain is the most frequently observed health issue. This review found that pain associated with lumbago decreases work engagement^80^ and increases psychological distress.^81^ As muscle‐required jobs may incur an unexpected burden on health, especially in women, more interventional studies should be warranted to alleviate work‐related physical problems.

The area of harassment and discrimination is sparsely studied. Workplaces where men predominate, and women are in the minority sometimes face this delicate issue. Previously, it was reported that women who experienced harassment, bullying, or gender discrimination were more likely to leave workplace, which sometimes resulted in early retirement.^82^ With the diversity and inclusion of minorities, including LGBT, this area is more important to increase women's motivation and work engagement.

Finally, mental health is the most studied area in this scoping review. Our review confirmed that women are more likely to develop sleep difficulty and psychological distress due to multiple social responsibilities.^83^ Excessive daytime sleepiness is a serious concern in the workplace concerning errors, accidents, absenteeism, and reduced productivity.^16^ More studies should be warranted to overcome these health issues among women. Lastly, mental health was the most frequent area for interventional studies. We found five studies^84^, ^85^, ^86^, ^87^, ^88^ that used an email and internet as a mediator, but few studies have used mobile applications, the recent central tool for health promotion and health management, and this is a method that should be fully explored in the future.

Our broad search strategy may be one of the study's strengths by covering a wide range of areas. However, this review had limitations. First, we used PubMed and Ichu‐shi, wherein results were quite different probably owing to variations in the thesaurus and interface; thus, we used identical search words as much as possible to minimize discrepancies between the two databases. Second, our strategy of the inclusion of “work” or “employed” for PubMed and thesaurus of working women for Ichu‐shi might have failed to identify some studies that investigated unpaid workers, such as caregivers for family members. Although our review was able to capture psychological burden associated with unpaid labor of women, this is an apparent study limitation, and thus, our review result applies to paid women. Third, in “Infertility, pregnancy, and childbirth” and “Menstruation, menopause, and genital disease,” there were more Japanese than English articles. One reason for the discrepancy in the numbers between the Japanese and English articles was that English articles on pregnancy‐related health issues, including pregnancy outcomes, were not limited to workers, while Japanese articles were more likely to be interested in pregnancy and work. In addition, Japanese articles may have been more promptly responded to domestic health policy; for example, in our review, we identified 10 studies that investigated infertility treatment; however, none were in English. Fourth, although we created the nine important areas and grouped literature according to these areas, these areas may change from time to time, along with the agenda that emerges in time. Therefore, future studies should be flexible in setting up these categories.

## 5 CONCLUSION

The results revealed that more scientific evidence needs to be accumulated on the health of working women, especially on reproductive health and gender inequality in Japan, using higher‐quality study designs. In addition, mobile applications are still underutilized in nine research areas, necessitating the active use of “Internet of Things” in the future research.

## AUTHOR CONTRIBUTIONS

Kyoko Nomura, Mayumi Tsuji, Miho Iida, Toru Takebayashi, and Akizumi Tsutsumi contributed to the study's conception. Kyoko Nomura, Mizuki Aoki, Kasane Miyauchi, Junko Hirayama, and Kengo Nagashima performed literature search and collected references. Kyoko Nomura wrote the draft and Junko Hirayama and Kyoko Kitagawa created figures. Mayumi Tsuji, Kyoko Kitagawa, and Akizumi Tsutsumi reviewed the article and provided advice. All authors have read and approved the final manuscript.

## FUNDING INFORMATION

This study was supported by the Japan Agency for Medical Research and Development (No. 22rea522007h0001).

## CONFLICT OF INTEREST STATEMENT

None.

## Supporting information


File S1.

File S2.

Table S1.

Table S2.

## Data Availability

Data sharing is available but not applicable to this article as no new data were created or analyzed in this study.

## References

[1] Statistical Bureau of Japan . Labour Survey. Accessed January 10, 2023. https://www.stat.go.jp/data/roudou/report/2020/pdf/summary1.pdf

[2] Gender Equity Bureau Cabinet Office . [KYOUDOSANKAKU] Joint Participation. Accessed August 4, 2023. https://www.gender.go.jp/public/kyodosankaku/2019/201912/201912_02.html

[3] Japan Society for Occupational Health Policy and Legislation Committee . Supporting the health of working women in Japan: summary report in English. Environ Occup Health Pract. 2021;3(1). doi:10.1539/eohp.2020-0028-OP

[4] Japan Ministry of Economy Trade and Industry . Annual Report of Survey of Health promotion for working women [HATARAKU JOSEI NO KENKOUHOJIZOUSHIN NIKANSURU JITTAICHOUSA]. 2017. Accessed May 22, 2023. https://www.meti.go.jp/policy/mono_info_service/healthcare/downloadfiles/H29kenkoujumyou‐report‐houkokusho‐josei.pdf

[5] The Ministry of Economics Trade and Industry of Japan . The Act on Promotion of Women's Participation and Advancement in the Workplace. Accessed January 10, 2023. 18. https://www.gender.go.jp/english_contents/about_danjo/lbp/pdf/promotion_of_woman.pdf

[6] The Ministry of Economy Trade and Industry . Health and Productivity Management. Accessed August 4, 2023. https://www.meti.go.jp/policy/mono_info_service/healthcare/kenkoukeiei_yuryouhouzin.html

[7] Sauvaget C , Nishino Y , Konno R , Tase T , Morimoto T , Hisamichi S . Challenges in breast and cervical cancer control in Japan. Lancet Oncol. 2016;17(7):e305‐e312. doi:10.1016/S1470-2045(16)30121-8 27396648

[8] Takeuchi M , Rahman M , Ishiguro A , Nomura K . Long working hours and pregnancy complications: women physicians survey in Japan. BMC Pregnancy Childbirth. 2014;14(1):245. doi:10.1186/1471-2393-14-245 25060410 PMC4121483

[9] Nagasu M , Sakai K , Ito A , et al. Prevalence and risk factors for low back pain among professional cooks working in school lunch services. BMC Public Health. 2007;7(1):171. doi:10.1186/1471-2458-7-171 17650300 PMC1971070

[10] Maeda E , Nomura K , Hiraike O , Sugimori H , Kinoshita A , Osuga Y . Domestic work stress and self‐rated psychological health among women: a cross‐sectional study in Japan. Environ Health Prev Med. 2019;24(1):75. doi:10.1186/s12199-019-0833-5 31847805 PMC6918574

[11] NPO Japan Medical Abstracts Society . Ichu‐shi. Accessed January 10, 2023. https://www.jamas.or.jp/english/

[12] Working Womens Health . Checklist for Creating Healthy Workplaces for Men and Women Workers. Accessed August 7, 2023. https://sites.google.com/view/wwh1999/%E7%8F%BE%E5%A0%B4%E3%81%A7%E4%BD%BF%E3%81%88%E3%82%8B%E3%83%84%E3%83%BC%E3%83%AB%E9%9B%86

[13] Fushimi M , Saito S , Shimizu T . Prevalence of depressive symptoms and related factors in Japanese employees as measured by the Center for Epidemiologic Studies Depression Scale (CES‐D). Community Ment Health J. 2013;49(2):236‐242. doi:10.1007/s10597-012-9542-x 23054146

[14] Utsugi M , Saijo Y , Yoshioka E , et al. Relationships of occupational stress to insomnia and short sleep in Japanese workers. Sleep. 2005;28(6):728‐735. doi:10.1093/sleep/28.6.728 16477960

[15] Kawakami N , Haratani T , Kobayashi F , et al. Occupational class and exposure to job stressors among employed men and women in Japan. J Epidemiol. 2004;14(6):204‐211. doi:10.2188/jea.14.204 15617394 PMC8784243

[16] Doi Y , Minowa M . Gender differences in excessive daytime sleepiness among Japanese workers. Soc Sci Med. 2003;56(4):883‐894. doi:10.1016/S0277-9536(02)00089-8 12560020

[17] Sato Y , Kubo S , Takemura S , et al. Different carcinogenic process in cholangiocarcinoma cases epidemically developing among workers of a printing company in Japan. Int J Clin Exp Pathol. 2014;7(8):4745‐4754. 25197345 PMC4152035

[18] Koyama K , Kubo S , Ueki A , et al. MR imaging and MR cholangiopancreatography of cholangiocarcinoma developing in printing company workers. Jpn J Radiol. 2017;35(5):233‐241. doi:10.1007/s11604-017-0626-y 28255646

[19] Yoshimura T , Kono S , Kuratsune M , Tani S . Nasal cancer mortality in areas with a high proportion of wood and furniture workers in Japan. J UOEH. 1983;5(4):433‐439. doi:10.7888/juoeh.5.433 6679650

[20] Nakano M , Omae K , Tanaka A , Hirata M . Possibility of lung cancer risk in indium‐exposed workers: an 11‐year multicenter cohort study. J Occup Health. 2019;61(3):251‐256. doi:10.1002/1348-9585.12050 30895696 PMC6499344

[21] Mayama M , Umazume T , Watari H , Nishiguchi S , Moromizato T , Watari T . Frequency of night shift and menstrual cycle characteristics in Japanese nurses working under two or three rotating shifts. J Occup Health. 2020;62(1):e12180. doi:10.1002/1348-9585.12180 33211393 PMC7676323

[22] Hamaya R , Mori M , Miyake K , Lee I . Association of smartphone‐recorded steps over years and change in cardiovascular risk factors among working‐age adults. J Am Heart Assoc. 2022;11(14):e025689. doi:10.1161/JAHA.121.025689 35861838 PMC9707835

[23] Miura K , Morikawa Y , Ishizaki M , Kido T , Naruse Y , Nakagawa H . A relationship between occupational factors and prolonged blood pressure elevation. Occup Health J. 2003;26(4):53‐58.

[24] Kubota A , Nagata J , Sugiyama M . Effects of a weight loss program with group participation supported by strengthened social support. Nihon Koshu Eisei Zasshi. 2008;55(5):327‐340. 18592983

[25] Ebihara Y , Miura H , Takahashi Y , Yamakawa M . Effectiveness of a diet improvement and weight loss support program using e‐mail and cell phone―based communications and personalized follow―up: a randomized controlled trial. Jap J Health Edu Promot. 2012;20(1):51‐59.

[26] Iriyama Y , Kushida O , Murayama N , Saito T . The effects of a food environment intervention and nutrition education on the amount of salt intake among workers and factors influencing behavior changes. Jpn J Nutr Diet. 2018;76(6):139‐155.

[27] Takao S , Tsutsumi A , Nishiuchi K , Mineyama S , Kawakami N . Effects of the job stress education for supervisors on psychological distress and job performance among their immediate subordinates: a supervisor‐based randomized controlled trial. J Occup Health. 2006;48(6):494‐503. doi:10.1539/joh.48.494 17179643

[28] Furukawa TA , Horikoshi M , Kawakami N , et al. Telephone cognitive‐behavioral therapy for subthreshold depression and presenteeism in workplace: a randomized controlled trial. PloS One. 2012;7(4):e35330. doi:10.1371/journal.pone.0035330 22532849 PMC3330821

[29] Michishita R , Jiang Y , Ariyoshi D , et al. The introduction of an active rest program by workplace units improved the workplace vigor and presenteeism among workers. J Occup Environ Med. 2017;59(12):1140‐1147. doi:10.1097/JOM.0000000000001121 28816734

[30] Imai R , Konishi T , Mibu A , Tanaka K , Nishigami T . Effect of pain neuroscience education and exercise on presenteeism and pain intensity in health care workers: a randomized controlled trial. J Occup Health. 2021;63(1):e12277. doi:10.1002/1348-9585.12277 34587662 PMC8481006

[31] Kimura R , Mori M , Tajima M , et al. Effect of a brief training program based on cognitive behavioral therapy in improving work performance: a randomized controlled trial. J Occup Health. 2015;57(2):169‐178. doi:10.1539/joh.14-0208-OA 25740675

[32] Toyama N , Taniguchi‐Tabata A , Sawada N , et al. Does instruction of oral health behavior for workers improve work performance?—quasi‐randomized trial. Int J Environ Res Public Health. 2018;15(12):2630. doi:10.3390/ijerph15122630 30477210 PMC6313762

[33] Imamura K , Kawakami N , Furukawa TA , et al. Effects of an internet‐based cognitive behavioral therapy intervention on improving work engagement and other work‐related outcomes. J Occup Environ Med. 2015;57(5):578‐584. doi:10.1097/JOM.0000000000000411 25749132

[34] Sakuraya A , Shimazu A , Imamura K , Namba K , Kawakami N . Effects of a job crafting intervention program on work engagement among Japanese employees: a pretest‐posttest study. BMC Psychol. 2016;4(1):49. doi:10.1186/s40359-016-0157-9 27776553 PMC5078879

[35] Imamura K , Kawakami N , Tsuno K , et al. Effects of web‐based stress and depression literacy intervention on improving work engagement among workers with low work engagement: an analysis of secondary outcome of a randomized controlled trial. J Occup Health. 2017;59(1):46‐54. doi:10.1539/joh.16-0187-OA 27885247 PMC5388612

[36] Kobayashi Y , Kaneyoshi A , Yokota A , Kawakami N . Effects of a worker participatory program for improving work environments on job stressors and mental health among workers: a controlled trial. J Occup Health. 2008;50(6):455‐470. doi:10.1539/joh.L7166 19023175

[37] Oshio T . How is an informal caregiver's psychological distress associated with prolonged caregiving? Evidence from a six‐wave panel survey in Japan. Qual Life Res. 2015;24(12):2907‐2915. doi:10.1007/s11136-015-1041-4 26049807

[38] Kikuzawa S , Uemura R . Parental caregiving and employment among midlife women in Japan. Res Aging. 2021;43(2):107‐118. doi:10.1177/0164027520941198 32787717 PMC7786392

[39] Tsuchiya M , Horio Y , Funazaki H , et al. Impact of gender and employment type on job loss among cancer survivors. Jpn J Clin Oncol. 2020;50(7):766‐771. doi:10.1093/jjco/hyaa040 32328623

[40] Makino A , Ohkawa Y . Factors related to ease of work for working pregnant women. Jpn J Matern Health. 2022;62(4):836‐844.

[41] Shimoyama H , Tsukamoto Y . Pregnancy discrimination against working pregnant women: nurses' role recognition and the state of nursing care. Jpn J Matern Health. 2020;61(1):123‐132.

[42] Ikeda T , Tamura C , Tanaka R . Maternity harassment against pregnant women in the workplace and its association with depression and fetal affection. Jpn J Matern Health. 2020;61(1):19‐27.

[43] Takeuchi M , Nomura K , Horie S , Okinaga H , Perumalswami CR , Jagsi R . Direct and indirect harassment experiences and burnout among academic faculty in Japan. Tohoku J Exp Med. 2018;245(1):37‐44. doi:10.1620/tjem.245.37 29760353

[44] Tsuno K , Kawachi I , Kawakami N , Miyashita K . Workplace bullying and psychological distress. J Occup Environ Med. 2018;60(12):1067‐1072. doi:10.1097/JOM.0000000000001433 30124499

[45] Morimoto T , Komaki K , Mori T , et al. The quality of mass screening for breast cancer by physical examination. Surg Today. 1993;23(3):200‐204. doi:10.1007/BF00309228 8467170

[46] Saito N , Takahashi M , Sairenchi T , Muto T . The impact of breast cancer on employment among Japanese women. J Occup Health. 2014;56(1):49‐55. doi:10.1539/joh.13-0140-OA 24430841

[47] Sari GN , Eshak ES , Shirai K , Fujino Y , Tamakoshi A , Iso H . Association of job category and occupational activity with breast cancer incidence in Japanese female workers: the JACC study. BMC Public Health. 2020;20(1):1106. doi:10.1186/s12889-020-09134-1 32664915 PMC7362447

[48] Yamauchi K , Nakashima M , Nakao M . What Japanese women with breast cancer decide: a mixed methods analysis of web‐based open‐ended responses. Asian Pac J Cancer Prev. 2021;22(9):2909‐2915. doi:10.31557/APJCP.2021.22.9.2909 34582661 PMC8850907

[49] Kaso M , Takahashi Y , Nakayama T . Factors related to cervical cancer screening among women of childrearing age: a cross‐sectional study of a nationally representative sample in Japan. Int J Clin Oncol. 2019;24(3):313‐322. doi:10.1007/s10147-018-1350-z 30276496

[50] Kaneko N . Factors associated with cervical cancer screening among young unmarried Japanese women: results from an internet‐based survey. BMC Womens Health. 2018;18(1):132. doi:10.1186/s12905-018-0623-z 30064505 PMC6069882

[51] Smith DR , Mihashi M , Adachi Y , et al. Menstrual disorders and their influence on low back pain among Japanese nurses. Ind Health. 2009;47(3):301‐312. doi:10.2486/indhealth.47.301 19531916

[52] Nohara M , Momoeda M , Kubota T , Nakabayashi M . Menstrual cycle and menstrual pain problems and related risk factors among Japanese female workers. Ind Health. 2011;49(2):228‐234. doi:10.2486/indhealth.MS1047 21173526

[53] Song M , Kanaoka H . Effectiveness of mobile application for menstrual management of working women in Japan: randomized controlled trial and medical economic evaluation. J Med Econ. 2018;21(11):1131‐1138. doi:10.1080/13696998.2018.1515082 30130990

[54] Nishikido N , Sasaki M , Yoshikawa E , Ito M . Development and evaluation of a training program for occupational health nurses regarding support for workers with cancer and their workplaces. J Occup Health. 2019;61(6):489‐497. doi:10.1002/1348-9585.12076 31309684 PMC6842007

[55] Mitsui K , Endo M , Imai Y , et al. Predictors of resignation and sick leave after cancer diagnosis among Japanese breast cancer survivors: a cross‐sectional study. BMC Public Health. 2021;21(1):138. doi:10.1186/s12889-021-10168-2 33446165 PMC7809813

[56] Endo M , Muto G , Imai Y , Mitsui K , Nishimura K , Hayashi K . Predictors of post‐cancer diagnosis resignation among Japanese cancer survivors. J Cancer Surviv. 2020;14(2):106‐113. doi:10.1007/s11764-019-00827-0 31721037

[57] Sako T , Inoue M , Watanabe T , Ishii A , Yokoyama T , Kumon H . Impact of overactive bladder and lower urinary tract symptoms on sexual health in Japanese women. Int Urogynecol J. 2011;22(2):165‐169. doi:10.1007/s00192-010-1250-x 20798921

[58] Kimata A , Ochiai R , Matsuoka S , Makaya M . Factors affecting employment of gynecological cancer survivors. J Jpn Soc Cancer Nurs. 2021;35:261‐272.

[59] Suzuki K , Ohata M , Hayashi N , et al. The effect of educational program of early detection for breast cancer [nyugansoukihakkennjnotamenonyubouself‐carewounagasukyoikuprogramnokouka]. J Jpn Soc Cancer Nurs. 2018;32:12‐22.

[60] Ghaznavi C , Sakamoto H , Yamasaki L , et al. Salaries, degrees, and babies: trends in fertility by income and education among Japanese men and women born 1943–1975—analysis of national surveys. PloS One. 2022;17(4):e0266835. doi:10.1371/journal.pone.0266835 35476638 PMC9045600

[61] Miyake Y , Tanaka K , Okubo H , Sasaki S , Arakawa M . Dietary vitamin D intake and prevalence of depressive symptoms during pregnancy in Japan. Nutrition. 2015;31(1):160‐165. doi:10.1016/j.nut.2014.06.013 25466661

[62] Kawanishi Y , Yoshioka E , Saijo Y , et al. The relationship between prenatal psychological stress and placental abruption in Japan, the Japan Environment and Children's Study (JECS). PloS One. 2019;14(7):e0219379. doi:10.1371/journal.pone.0219379 31283785 PMC6613679

[63] Ohira S , Motoki N , Shibazaki T , et al. Alcohol consumption during pregnancy and risk of placental abnormality: the Japan environment and children's study. Sci Rep. 2019;9(1):10259. doi:10.1038/s41598-019-46760-1 31312010 PMC6635355

[64] Suzumori N , Ebara T , Matsuki T , et al. Effects of long working hours and shift work during pregnancy on obstetric and perinatal outcomes: a large prospective cohort study—Japan environment and children's study. Birth. 2020;47(1):67‐79. doi:10.1111/birt.12463 31667913 PMC7065104

[65] Minamida T , Iseki A , Sakai H , Imura M , Okano T , Tanii H . Do postpartum anxiety and breastfeeding self‐efficacy and bonding at early postpartum predict postpartum depression and the breastfeeding method? Infant Ment Health J. 2020;41(5):662‐676. doi:10.1002/imhj.21866 32578270

[66] Li M , Okamoto R , Tada A , Kiya M . Factors associated with prenatal smoking cessation interventions among public health nurses in Japan. Int J Environ Res Public Health. 2020;17(17):6135. doi:10.3390/ijerph17176135 32846936 PMC7503931

[67] Takeuchi M , Rahman M , Ishiguro A , Nomura K . Long working hours and pregnancy complications: women physicians survey in Japan. Obstet Gynecol Surv. 2014;69(11):649‐651. doi:10.1097/01.ogx.0000458791.93682.2a PMC4121483 25060410

[68] Tamaki T , Kaneita Y , Ohida T , et al. Alcohol consumption behavior of pregnant women in Japan. Prev Med (Baltim). 2008;47(5):544‐549. doi:10.1016/j.ypmed.2008.07.013 18708087

[69] Nakamura Y , Sato M , Watanabe I . Positive emotion and its changes during pregnancy: adjunct study of Japan environment and children's study in Miyagi prefecture. Tohoku J Exp Med. 2018;245(4):223‐230. doi:10.1620/tjem.245.223 30058596

[70] Eguchi H , Shimazu A , Fujiwara T , et al. The effects of workplace psychosocial factors on whether Japanese dual‐earner couples with preschool children have additional children: a prospective study. Ind Health. 2016;54(6):498‐504. doi:10.2486/indhealth.2016-0080 27760893 PMC5136606

[71] Nagusa M , Sasaki A . 3 month evaluation of preconception care health education program for working women in reproductive age [Seijukukishuuroujoseinitaisurupreconseptioncarekenkoprogramno3kagetsu madenohyouka]. Jpn J Health Edu Promot. 2020;28(2):81‐91. doi:10.11260/kenkokyoiku.28.81

[72] Taka F , Nomura K , Horie S , et al. Organizational climate with gender equity and burnout among university academics in Japan. Ind Health. 2016;54(6):480‐487. doi:10.2486/indhealth.2016-0126 27725562 PMC5136604

[73] Inoue A , Tsutsumi A , Eguchi H , et al. Workplace social capital and refraining from seeking medical care in Japanese employees: a 1‐year prospective cohort study. BMJ Open. 2020;10(8):e036910. doi:10.1136/bmjopen-2020-036910 PMC7401998 32747350

[74] Eguchi H , Tsuda Y , Tsukahara T , Washizuka S , Kawakami N , Nomiyama T . The effects of workplace occupational mental health and related activities on psychological distress among workers. J Occup Environ Med. 2012;54(8):939‐947. doi:10.1097/JOM.0b013e31825107bb 22776808

[75] Tanaka E , Momoeda M , Osuga Y , et al. Burden of menstrual symptoms in Japanese women: results from a survey‐based study. J Med Econ. 2013;16(11):1255‐1266. doi:10.3111/13696998.2013.830974 24015668

[76] Du L , Xu B , Huang C , Zhu L , He N . Menopausal symptoms and perimenopausal healthcare‐seeking behavior in women aged 40–60 years: a community‐based cross‐sectional survey in Shanghai, China. Int J Environ Res Public Health. 2020;17(8):2640. doi:10.3390/ijerph17082640 32290565 PMC7215590

[77] Miyaoka Y , Akimoto Y , Ueda K , et al. Fulfillment of the premenstrual dysphoric disorder criteria confirmed using a self‐rating questionnaire among Japanese women with depressive disorders. Biopsychosoc Med. 2011;5(1):5. doi:10.1186/1751-0759-5-5 21535889 PMC3110105

[78] Schoep ME , Adang EMM , Maas JWM , De Bie B , Aarts JWM , Nieboer TE . Productivity loss due to menstruation‐related symptoms: a nationwide cross‐sectional survey among 32 748 women. BMJ Open. 2019;9(6):e026186. doi:10.1136/bmjopen-2018-026186 PMC6597634 31248919

[79] Tokunaga M , Hashimoto H . The socioeconomic within‐gender gap in informal caregiving among middle‐aged women: evidence from a Japanese nationwide survey. Soc Sci Med. 2017;173:48‐53. doi:10.1016/j.socscimed.2016.11.037 27918891

[80] Tsuboi Y , Murata S , Naruse F , Ono R . Association between pain‐related fear and presenteeism among eldercare workers with low back pain. Eur J Pain. 2019;23:495‐502. doi:10.1002/ejp.1323 30289190

[81] Sakamoto Y , Amari T , Shimo S . The relationship between pain psychological factors and job stress in rehabilitation workers with or without chronic pain. Work. 2018;61(3):357‐365. doi:10.3233/WOR-182814 30373991

[82] Nomura K , Gohchi K . Impact of gender‐based career obstacles on the working status of women physicians in Japan. Soc Sci Med. 2012;75(9):1612‐1616. doi:10.1016/j.socscimed.2012.07.014 22867864

[83] Park YM , Seo YJ , Matsumoto K , Shinkoda H , Park KP . Sleep in relation to age, sex, and chronotype in Japanese workers. Percept Mot Skills. 1998;87(1):199‐215. doi:10.2466/pms.1998.87.1.199 9760647

[84] Ubara A , Tanizawa N , Harata M , et al. How does E‐mail‐delivered cognitive behavioral therapy work for young adults (18–28 years) with insomnia? Mediators of changes in insomnia, depression, anxiety, and stress. Int J Environ Res Public Health. 2022;19(8):4423. doi:10.3390/ijerph19084423 35457291 PMC9029643

[85] Kuribayashi K , Imamura K , Watanabe K , et al. Effects of an internet‐based cognitive behavioral therapy (iCBT) intervention on improving depressive symptoms and work‐related outcomes among nurses in Japan: a protocol for a randomized controlled trial. BMC Psychiatry. 2019;19(1):245. doi:10.1186/s12888-019-2221-5 31391029 PMC6686442

[86] Imamura K , Kawakami N , Furukawa TA , Matsuyama Y , Shimazu A , Kasai K . Effects of an internet‐based cognitive behavioural therapy intervention on preventing major depressive episodes among workers: a protocol for a randomised controlled trial. BMJ Open. 2015;5(5):e007590. doi:10.1136/bmjopen-2015-007590 PMC4431123 25968004

[87] Kawai K , Yamazaki Y , Nakayama K . Process evaluation of a web‐based stress management program to promote psychological well‐being in a sample of white‐collar Workers in Japan. Ind Health. 2010;48(3):265‐274. doi:10.2486/indhealth.48.265 20562501

[88] Suzuki E , Tsuchiya M , Hirokawa K , Taniguchi T , Mitsuhashi T , Kawakami N . Evaluation of an internet‐based self‐help program for better quality of sleep among Japanese workers: a randomized controlled trial. J Occup Health. 2008;50(5):387‐399. doi:10.1539/joh.L7154 18716392

