# Supplementary material for: The quantity and quality of scientific evidence about the health of working women in occupational health of Japan: A scoping review
Source: J Occup Health. 2023 Oct 16;65(1):e12427. doi: 10.1002/1348-9585.12427 (PMC10579630; doi:10.1002/1348-9585.12427)
Supplement: Supplementary file 1 — File S1. [file JOH2-65-e12427-s002.pdf]

pmid	No work re	Out of foc	Specific pa	men worke	filed	St_design	classificati	memo	Title	abstract	keywords	Authors	Citation	FirstAutho	Journal	Publication
536678	0	0	0		Lifestyle re	0	diet and nu	Anemia	Follow-up	Haemoglol	Adult; Agri	Futatsuka	J Epidemic	Futatsuka	J Epidemic	1979
1303959	0	0	0		Work relat	1	PCB, asbes	cadmium	Critical lev	The critica	Adult; Age	Nomiyama	IARC Sci P	Nomiyama	IARC Sci P	1992
1399020	0	0	0		Work relat	1	substance e	toluene	Absence o	Possible c	Adult; Che	Nakatsuka	Int Arch Oc	Nakatsuka	Int Arch Oc	1992
1487329	0	0	0		Work relat	1	substance e	methanol	Formic aci	A semiaut	Adult; Chr	Yasugi T, I	Int Arch Oc	Yasugi T	Int Arch Oc	1992
1528581	0	0	0		Work relat	0	PCB, asbes	lead	Frequency	NA	Adolescen	Horiguchi	Osaka City	Horiguchi	Osaka City	1992
1577524	0	0	0		Work relat	1	substance e	Monometh	Occupatio	The relatic	Adult; Air I	Kawai T, Y	Int Arch Oc	Kawai T	Int Arch Oc	1992
1614630	0	0	0		Mental He	0	overtime and mental		Karoshi: d	Japanese I	Adult; Bur	Anders RL	Nurs Healt	Anders RL	Nurs Healt	1992
1628527	0	0	0		Lifestyle re	1	others	lifehabit	Morningne	The Japan	Adult; Agir	Ishihara K	Chronobio	Ishihara K	Chronobio	1992
1765547	0	0	0		Work relat	1	substance e	tetrachlor	Subjective	Subjective	Adolescen	Cai SX, Hu	Ind Health	Cai SX	Ind Health	1991
1830000	0	0	0		Work relat	1	physical sy	allergy	Allergic co	We report	Adult; Der	Okamoto H	Cutis. 199	Okamoto H	Cutis	1991
1842959	0	0	0		Mental He	1	occupational stress		Burnout pl	To identify	Adult; Bur	Hisashige	J Hum Erg	Hisashige	J Hum Erg	1991
1842960	0	0	0		Mental He	0	overtime and mental		Work and	With the o	Adult; Fem	Karaki M.	J Hum Erg	Karaki M	J Hum Erg	1991
1842961	0	0	0		Mental He	1	overtime a		Long work	Two hundr	Adult; Age	Uehata T.	J Hum Erg	Uehata T	J Hum Erg	1991
1844217	0	0	0		Lifestyle re	1	lifestyle	smoke	Interperso	A question	Adult; Awa	Takahashi	Asia Pac J	Takahashi	Asia Pac J	1991
2568704	0	0	0		Work relat	1	PCB, asbes	lead	Serum suc	The relatic	Adult; Age	Ito Y, Fuka	Toxicol Le	Ito Y	Toxicol Le	1989
2641666	0	0	0		Work relat	1	PCB, asbes	asbesto	Relationsh	We investi	Adult; Age	Kishimoto	Chest. 198	Kishimoto	Chest	1989
2739098	0	0	0		Mental He	1	scale dev		Psychome	NA	Adult; Affe	Iwata N, S	Sangyo Iga	Iwata N	Sangyo Iga	1989
2749070	0	0	0		Mental He	0	occupatior		Basic patt	597 sampl	Adult; Fem	Hayashi S,	J UOEH. 19	Hayashi S	J UOEH	1989
2763868	0	0	0		Work and I	0	Returntow	operation	Return to v	One hundr	Adolescen	Shigenobu	Acta Med	Shigenobu	Acta Med	1989
2782740	0	0	0		Work relat	3	physical sy	農夫肺	Five-year f	To examin	Adult; Anti	Kusaka H,	Am Rev Re	Kusaka H	Am Rev Re	1989
2817157	0	0	0		Mental He	0	occupatior		Prevalence	We measu	Adult; Dep	Iwata N, O	Am J Publi	Iwata N	Am J Publi	1989
2853531	0	0	0		Work relat	0	VDT		Effect of v	NA	Accommo	Tokoro T.	Acta Ophtl	Tokoro T	Acta Ophtl	1988
3024484	0	0	0		Work relat	1	substance e	vinyl chlori	Early dete	Health exa	Adult; Car	Sugita M,	Am J Ind M	Sugita M	Am J Ind M	1986
3188004	0	0	0		Work relat	2	substance e	iron	A case-co	A case-co	Adult; Age	Shimizu H,	Tohoku J E	Shimizu H	Tohoku J E	1988
3256938	0	0	0		Work relat	1	substance e	toluene	Urinary hip	The biolog	Adult; Chr	Sugita M,	Tokai J Exp	Sugita M	Tokai J Exp	1988
3360620	0	0	0		Career /ec	1	satisfactio		Factor stru	NA	Adult; Fac	Iwata N, O	Ind Health	Iwata N	Ind Health	1988
3374106	0	0	0		Work relat	0	physical sy	農夫肺	Pollen alle	NA	Adult; Agri	Teranishi I	J Soc Occu	Teranishi I	J Soc Occu	1988
3592580	0	0	0		Career /ec	1	satisfactio		Factors af	The impor	Adult; Allie	Shimizu Y,	Ann Acad	Shimizu Y	Ann Acad	1987
3610455	0	0	0		Work relat	0	physical sy	農夫肺	Some epid	A total of 3	Adult; Age	Konishi E,	Int J Epide	Konishi E	Int J Epide	1987
3710461	0	0	0		Lifestyle re	0	diet and nu	anemia	Hemoglobi	NA	Adult; Age	Watanabe	Hum Biol.	Watanabe	Hum Biol	1986
3924093	0	0	0		Work relat	0	PCB, asbes	PCB	Oculoderm	Oculoderm	Adult; Age	Fischbein	Br J Ind M	Fischbein	Br J Ind M	1985
5431873	0	0	0		Work relat	0	comfortab	gas	Chronic ob	2735 form	Arsenicals	Nishimoto	Am Rev Re	Nishimoto	Am Rev Re	1970
5602855	0	0	0		Work relat	0	physical sy		Clinical ob	618 cases	Adult; Astl	Wada S, N	Hiroshima	Wada S	Hiroshima	1967
6629861	0	0	0		Work relat	1	VDT	VDT	An investi	NA	Adolescen	Yamamura	Ind Health	Yamamura	Ind Health	1983
6679650	0	0	0		Work relat	0	occupatior	cancer	Nasal canc	To see wh	Adolescen	Yoshimura	J UOEH. 19	Yoshimura	J UOEH	1983
6852931	0	0	0		Work relat	0	substance e	n-hexane	A study on	n-Hexane	Adolescen	Iwata M, T	Int Arch Oc	Iwata M	Int Arch Oc	1983
7314084	0	0	0		Work relat	1	PCB, asbes	asbesto	Asbestos a	The cance	Adult; Age	Sera Y, Ka	Tohoku J E	Sera Y	Tohoku J E	1981
7560524	0	0	0		Career /ec	0	satisfactio	Med_perso	Job satisfa	This study	Adult; Car	Yamashita	J Adv Nurs	Yamashita	J Adv Nurs	1995
7593053	0	0	0		Mental He	0	occupatior	sleep	Sleep habi	The relatic	Adolescen	Motohashi	J Biosoc S	Motohashi	J Biosoc S	1995
7625479	0	0	0		Mental He	0	occupatior		Prevalence	Most preva	Adult; Cros	Ozaki N, O	Am J Psycl	Ozaki N	Am J Psycl	1995
7627312	0	0	0		Work relat	1	PCB, asbes	asbesto	Decline in	To examin	Adult; Asb	Nakadate	Occup Env	Nakadate	Occup Env	1995
7672857	0	0	0		Work relat	0	substance e	acetone	Acetone e	To develop	Acetone; A	Satoh T, O	Int Arch Oc	Satoh T	Int Arch Oc	1995
7788517	0	0	0		Work relat	0	physical sy	農夫肺	Clinical an	The increa	Adult; Anir	Shimizu T,	Ann Allerg	Shimizu T	Ann Allerg	1995
7794011	0	0	0		Work relat	0	substance e	ketone	Biological	The relatic	Adult; Age	Yoshikawa	Arch Envir	Yoshikawa	Arch Envir	1995
8014068	0	0	0		Work relat	1	comfortab	gas	Cytogenet	A high inci	Aged; Arse	Shakil FA,	Hiroshima	Shakil FA	Hiroshima	1993
8087336	0	0	0		Work relat	2	comfortab	pulmonary	Idiopathic	Idiopathic	Adolescen	Iwai K, Mo	Am J Resp	Iwai K	Am J Resp	1994
8184416	0	0	0		Lifestyle re	1	diet and nu	anemia	Influence o	Studies we	Adult; Ane	Fujii M, M	Tokushima	Fujii M	Tokushima	1993
8186656	0	0	0		Lifestyle re	1	others	checkup u	A study on	A longitudi	Adult; Age	Nakama H	Clin Invest	Nakama H	Clin Invest	1994
8226113	0	0	0		Work relat	1	PCB, asbes	lead	Interrelatio	Using a flu	Adolescen	Tomokuni	Ind Health	Tomokuni	Ind Health	1993
8243416	0	0	0		Mental He	1	occupatior		Occupatio	To identify	Adult; Bur	Hisashige	Environ Re	Hisashige	Environ Re	1993
8342194	0	0	0		Work and I	3	Returntow	stroke	Factors inf	Few studie	Adolescen	Saeki S, O	Stroke. 19	Saeki S	Stroke	1993
8467170	0	0	0		Menstruat	1	breast ca	screening	The quality	Mass scre	Adult; Age	Morimoto	Surg Toda	Morimoto	Surg Toda	1993
8482589	0	0	0		Work relat	1	substance e	acetate	Determina	In control	Acetates;	Sakai T, A	Int Arch Oc	Sakai T	Int Arch Oc	1993
8543474	0	0	0		Work relat	0	safety and	safety	Some aspe	The object	Adult; Age	Mirbod SM	Ind Health	Mirbod SM	Ind Health	1995
8704861	0	0	0		Work relat	0	PCB, asbes	metal	Decreased	To study ir	Adolescen	Kusaka Y,	Occup Env	Kusaka Y	Occup Env	1996
8768672	0	0	0		Work relat	1	comfortab	noise	Effects of	To investig	Adult; Cate	Sudo A, Ng	Ind Health	Sudo A	Ind Health	1996
8832294	0	0	0		Work relat	1	PCB, asbes	lead	Compariso	To elucida	Adult; Ami	Oishi H, N	Int Arch Oc	Oishi H	Int Arch Oc	1996
8868401	0	0	0		Work relat	1	PCB, asbes	lead	Fluoromet	A fluorome	Adolescen	Oishi H, N	J Anal Tox	Oishi H	J Anal Tox	1996
8877839	0	0	0		Mental He	1	occupatior		Prevalence	To learn th	Adolescen	Kawakami	J Occup Er	Kawakami	J Occup Er	1996
8908844	0	0	0		Lifestyle re	1	LRD	glucose m	Epidemiolo	Hypoglyce	Adult; Ana	Mogi T, W	Ind Health	Mogi T	Ind Health	1996
8923608	0	0	0		Mental He	1	overtime a		Effects of	In accorda	Administra	Maruyama	Scand J W	Maruyama	Scand J W	1996
9001913	0	0	0		Work relat	1	shift	stress	Changes in	The effect	Adult; Affe	Kobayashi	Int Arch Oc	Kobayashi	Int Arch Oc	1997
9009498	0	0	0		Mental He	1	occupatior		The relatic	In order to	Adult; Cros	Shigemi J,	Ind Health	Shigemi J	Ind Health	1997
9127550	0	0	0		Work relat	1	Musle ske	労災	National s	Out of the	Accidents,	Kuwashim	Ind Health	Kuwashim	Ind Health	1997
9127551	0	0	0		Work relat	1	Musle ske	LBP	Low back	A cross-se	Adult; Coo	Ono Y, Shi	Ind Health	Ono Y	Ind Health	1997
9172206	0	0	0		Work relat	0	shift	sleep	Twins und	Two pairs	Adult; Circ	Fukuda K.	Percept M	Fukuda K	Percept M	1997
9201863	0	0	0		Work relat	1	Musle ske	predictor	Risk indica	A question	Adult; Age	Matsui H,	Spine (Phi	Matsui H	Spine (Phi	1997
9243732	0	0	0		Work relat	2	substance e	methyltetr	Specific ar	Occupatio	Adult; Cas	Yokota K,	Scand J W	Yokota K	Scand J W	1997
9296547	0	0	0		Mental He	1	occupatior		Stress and	In order to	Adult; Bur	Nadaoka T	Acta Psych	Nadaoka T	Acta Psych	1997
9473852	0	0	0		Mental He	1	occupatior	anxiety	The Japan	Symptom	Adolescen	Iwata N, M	Ind Health	Iwata N	Ind Health	1998
9621742	0	0	0		Mental He	1	occupatior	anxiety	Positive ar	The factor	Adult; Anx	Iwata N, M	Psychol Re	Iwata N	Psychol Re	1998
9624268	0	0	0		Work relat	1	Musle ske	付き指	Epicondyli	To investig	Adult; Coo	Ono Y, Na	Occup Env	Ono Y	Occup Env	1998
9755989	0	0	0		Lifestyle re	1	others	bowel mov	The relatic	It is said th	Adolescen	Kunimoto	Hepatogas	Kunimoto	Hepatogas	1998
9760647	0	0	0		Mental He	1	occupatior	sleep	Sleep in re	The Morni	Adult; Affe	Park YM, I	Percept M	Park YM	Percept M	1998
9810151	0	0	0		Lifestyle re	1	lifestyle	smoke	Evaluation	This paper	Adult; Ben	Muto T, Na	Ind Health	Muto T	Ind Health	1998
9849826	0	0	0		Lifestyle re	1	lifestyle	obesity	Associatio	This study	Adipose Ti	Nakamura	Eur J Epide	Nakamura	Eur J Epide	1998
9873243	0	0	0		Lifestyle re	1	diet and nu	diet	Eating pro	Fewer stu	Adult; Anti	Nakamura	Psychothe	Nakamura	Psychothe	1999
10319573	0	0	0		Work envir	1	productivit	absence	Sickness a	This study	Absenteei	Muto T, Su	Ind Health	Muto T	Ind Health	1999

10319574	0	0	0	Mental He	0	stress management		Psychosor	This study	Adult; Buri	Araki Y, M	Ind Health	Araki Y	Ind Health	1999
10319575	0	0	0	Mental He	0	occupational stress		Job stress	To examin	Adolescen	Iwata N, K	Ind Health	Iwata N	Ind Health	1999
10328161	0	0	0	Work relat	1	Musle ske	Muskel	Prognosis	Accidental	Accidents,	Shinohara	Tohoku J E	Shinohara	Tohoku J E	1998
10341745	0	0	0	Mental He	3	occupational stress		Perceived	To determ	Adult; Age	Mino Y, Sh	Occup Env	Mino Y	Occup Env	1999
10364747	0	0	0	Work relat	0	physical syı	農夫肺	Hypersens	We report	Adult; Aga	Akizuki N,	Respiratio	Akizuki N	Respiratio	1999
10459693	0	0	0	Work relat	1	shift	Shift	Nurses' w	To assess	Adult; Arou	Fukuda H,	Psychiatry	Fukuda H	Psychiatry	1999
10478405	0	0	0	Lifestyle re	1	lifestyle	smoke	Smoking p	To estimat	Adult; Fem	Ohida T, C	Tob Contro	Ohida T	Tob Contro	1999
10568621	0	0	0	Lifestyle re	1	lifestyle	smoke	Workplace	There are	Adult; Cho	Mizoue T,	Am J Epide	Mizoue T	Am J Epide	1999
10600436	0	0	0	Lifestyle re	1	lifestyle	smoke	Support fo	While vario	Adult; Atti	Mizoue T,	Prev Med.	Mizoue T	Prev Med	1999
10616265	0	0	0	Lifestyle re	1	lifestyle	smoke	The role o	Although s	Adult; Atti	Shigemi J,	J Epidemic	Shigemi J	J Epidemic	1999
10713473	0	0	0	Work relat	0	PCB, asbes	lead	Changes o	To determ	Adult; Age	Jin YP, Kol	Toxicol Le	Jin YP	Toxicol Le	2000
10787134	0	0	0	Work relat	1	substance e	chemical	Improveme	Thermoreg	Aged; Agri	Hayashi C.	Int Arch O	Hayashi C	Int Arch O	2000
10795385	0	0	0	Lifestyle re	1	LRD	CVD	Increased	The purpo	Adult; Bod	Nakamura	Occup Me	Nakamura	Occup Me	2000
10812840	0	0	0	Work relat	1	PCB, asbes	lead	Blood lead	The preser	Adult; Ami	Higashikav	Ind Health	Higashikav	Ind Health	2000
10812842	0	0	0	Work relat	1	substance e	benxzen	Urinary ph	A hand-sa	Acetylcyst	Inoue O, K	Ind Health	Inoue O	Ind Health	2000
10812850	0	0	0	Work relat	1	safety and v	injury	Does job s	To clarify v	Accidents,	Murata K,	Ind Health	Murata K	Ind Health	2000
10851352	0	0	0	Work relat	1	Musle ske	Muskel	An epidem	The factor	Adult; Auto	Miyamoto	J Nippon M	Miyamoto	J Nippon M	2000
10858751	0	0	0	Lifestyle re	1	oral health	oral	Present st	The aim of	Adolescen	Kawamura	Int Dent J.	Kawamura	Int Dent J	1999
10860301	0	0	0	Mental He	3	occupatior	suicide	Depressive	In Japan, r	Adult; Age	Tamakosh	J Epidemic	Tamakosh	J Epidemic	2000
11052206	0	0	0	Work and l	0	Returntow	Returntow	Disability r	Return to v	Aged; Disa	Saeki S.	Disabil Re	Saeki S	Disabil Re	2000
11061483	0	0	0	Lifestyle re	4	lifestyle	smoke	Effectivene	In Japan, t	Adolescen	Kadowaki	Ind Health	Kadowaki	Ind Health	2000
11157125	0	0	0	Work envir	1	retention	Int_Leav	Factors af	Using the	Adult; Fem	Ito H, Eise	Psychiatr S	Ito H	Psychiatr S	2001
11213026	0	0	0	Work relat	1	shift	shift	Influence o	Night shift	Adrenocor	Munakata	Hypertens	Munakata	Hypertens	2001
11227800	0	0	0	Lifestyle re	1	diet and nu	diet	Serum bio	Although s	Adult; Age	Sasaki S, U	J Nutr Sci	Sasaki S	J Nutr Sci	2000
11279837	0	0	0	Career /ec	1	self-effica	medical pe	Occupatio	The preser	Adult; Clin	Imai K.	J UOEH. 20	Imai K	J UOEH	2001
11355294	0	0	0	Mental He	1	occupational stress		Mental hea	In order to	Adult; Ana	Kageyama	Int Arch O	Kageyama	Int Arch O	2001
11409598	0	0	0	Mental He	1	occupational stress		Associatio	This study	Adult; Cros	Tsutsumi A	Scand J W	Tsutsumi A	Scand J W	2001
11422847	0	0	0	Mental He	0	occupatior	sleep	Estimated	Sleep debt	Adult; Buri	Kageyama	Psychiatry	Kageyama	Psychiatry	2001
11474902	0	0	0	Work relat	0	shift	shift	Nutrient in	Based on a	Adult; Circ	Sudo N, O	Int J Food	Sudo N	Int J Food	2001
11682362	0	0	0	Work relat	1	comfortab	sick house	Environme	Sick buildi	Adult; Con	Mizoue T,	Am J Epide	Mizoue T	Am J Epide	2001
11719612	0	0	0	Mental He	0	stress mar	seeking fo	Characteri	This study	Adult; Con	Kurioka S,	Occup Me	Kurioka S	Occup Me	2001
11926510	0	0	0	Work relat	1	PCB, asbes	lead	Computeri	To examin	Adult; Age	Yokoyama	Ind Health	Yokoyama	Ind Health	2002
11926512	0	0	0	Lifestyle re	1	LRD	CVD	Distributio	To examin	Adult; Age	Nakanishi	Ind Health	Nakanishi	Ind Health	2002
11926513	0	0	0	Work relat	1	substance e	chemical	A cross-se	The relatic	Adult; Air I	Yokota K,	Ind Health	Yokota K	Ind Health	2002
11926518	0	0	0	Work relat	0	substance e	ナフタレン	Occupatio	In an elect	Adhesives	Yokota K,	Ind Health	Yokota K	Ind Health	2002
12062914	0	0	0	Work relat	2	PCB, asbes	silica	A case-cor	In southea	Adult; Age	Tsuda T, M	Ann Epide	Tsuda T	Ann Epide	2002
12111570	0	0	0	Lifestyle re	0	oral health	oral	Survey of c	Over 350 b	Adult; Bac	Nomura Y,	J Infect Ch	Nomura Y	J Infect Ch	2002
12164322	0	0	0	Lifestyle re	1	lifestyle	smoke	Change of	Two cross	Adult; Age	Kaetsu A,	J Epidemic	Kaetsu A	J Epidemic	2002
12215017	0	0	0	Work and l	1	Returntow	Returntow	Long-term	For addres	Acute Dise	Suzuki-Tsu	Int J Hema	Suzuki-Tsu	Int J Hema	2002
12242321	0	0	0	Lifestyle re	3	others	UA	Effect of a	Serum uric	Adult; Age	Kuzuya M,	J Gerontol	Kuzuya M	J Gerontol	2002
12406201	0	0	0	Career /ec	1	education	medical pe	Communic	This surve	Adult; Clin	Ito M, Lam	Nurs Healt	Ito M	Nurs Healt	2002
12407474	0	0	0	Lifestyle re	3	lifestyle	smoke	Smoking b	The preval	Adult; Coh	Kitajima T	Public Hea	Kitajima T	Public Hea	2002
12409531	0	0	0	Work relat	1	PCB, asbes	lead	Lead induc	Although l	Adolescen	Nomiyama	Occup Env	Nomiyama	Occup Env	2002
12560020	0	0	0	Mental He	1	occupatior	sleep	Gender dif	Excessive	Adult; Disc	Doi Y, Min	Soc Sci Me	Doi Y	Soc Sci Me	2003
12587612	0	0	0	Lifestyle re	1	LRD	HTN	Gender an	The aim of	Adult; Age	Hori Y, Toy	J Epidemic	Hori Y	J Epidemic	2003
12592578	0	0	0	Work relat	1	substance e	ketone	Methyl iso	To examin	Adult; Age	Kawai T, Z	Int Arch O	Kawai T	Int Arch O	2003
12631166	0	0	0	Lifestyle re	1	oral health	oral	Effectivene	The purpo	Adult; Chi-	Morishita I	J Oral Reh	Morishita I	J Oral Reh	2003
12675114	0	0	0	Lifestyle re	3	LRD	lipid	Job charac	To observe	Adult; Age	Tsutsumi A	J Epidemic	Tsutsumi A	J Epidemic	2003
12713020	0	0	0	Lifestyle re	1	lifestyle	smoke	An epidem	The workp	Adult; Data	Utsunomiy	Keio J Mec	Utsunomiy	Keio J Mec	2003
12725470	0	0	0	Mental He	3	overtime a	overtime	A prospect	sleep	Adult; Age	Tarumi K,	Ind Health	Tarumi K	Ind Health	2003
12763706	0	0	0	Mental He	1	occupational stress		Associatio	Associatio	Adult; Alco	Tsutsumi A	Int J Behav	Tsutsumi A	Int J Behav	2003
12899450	0	0	0	Lifestyle re	1	diet and nu	navy nutrit	New appre	In 1943, or	Adolescen	Hashimoto	Cell Mol B	Hashimoto	Cell Mol B	2003
12900083	0	0	0	Work relat	1	substance e	chemical	Visual dys	The aim of	Adult; Colo	Gong Y, Ki	Neurotoxic	Gong Y	Neurotoxic	2003
12908334	0	0	0	Work relat	1	shift	shift	Fatigue co	For female	Adult; Ana	Sudo N, O	J Hum Erg	Sudo N	J Hum Erg	2002
12916754	0	0	0	Mental He	1	occupatior	SOC	Associatio	We conduc	Adult; Citie	Nasermoa	Ind Health	Nasermoa	Ind Health	2003
12950708	0	0	0	Mental He	3	occupational stress		Predictors	Major dep	Adult; Coh	Tokuyama	Psychiatry	Tokuyama	Psychiatry	2003
14503038	0	0	0	Career /ec	1	education	profession	Relationsh	We investi	Adult; Age	Shimizu T,	J UOEH. 20	Shimizu T	J UOEH	2003
14526133	0	0	0	Mental He	1	stress management		Alexithymi	A number	Adult; Affe	Kojima M,	Psychothe	Kojima M	Psychothe	2003
14564883	0	0	0	Work relat	1	shift	shift	Job stress,	A cross-se	Adolescen	Nakata A,	J Hum Erg	Nakata A	J Hum Erg	2001
14568635	0	0	0	Mental He	1	occupational stress		Reporting	The object	Adult; Age	Nakao M,	J Clin Epid	Nakao M	J Clin Epid	2003
14620670	0	0	0	Career /ec	1	education	profession	Relationsh	The preser	Adaptatior	Shimizu T,	Ind Health	Shimizu T	Ind Health	2003
14646270	0	0	0	Lifestyle re	1	lifestyle	alcohol	Alcohol co	The relatic	Adult; Alco	Yamada Y	J Occup He	Yamada Y	J Occup He	2003
14646273	0	0	0	Work and l	1	WFC		Interperso	To examin	Adult; Buri	Fujiwara K	J Occup He	Fujiwara K	J Occup He	2003
14646296	0	0	0	Mental He	4	occupatior	com	Relationsh	We investi	Adult; Buri	Shimizu T,	J Occup He	Shimizu T	J Occup He	2003
14963255	0	0	0	Work envir	1	productivit	Productivit	Relationsh	To investig	Adult; Effic	Shimizu T,	Occup Me	Shimizu T	Occup Me	2004
15042635	0	0	0	Work relat	2	shift	shift	Biochemic	Female nu	Adult; Cas	Inoue K, K	Res Nurs H	Inoue K	Res Nurs H	2004
15065639	0	0	0	Work relat	1	substance e	toluene	A survey o	Biological	Adult; Air I	Tanaka K,	Fukushima	Tanaka K	Fukushima	2003
15076154	0	0	0	Lifestyle re	1	LRD	HTN	Lack of as	To examin	Adult; Bloc	Suwazono	J Hyperten	Suwazono	J Hyperten	2004
15127783	0	0	0	Mental He	1	occupatior	biomarker	Urinary ca	This study	Adult; Cate	Fujiwara K	Scand J W	Fujiwara K	Scand J W	2004
15154171	0	0	0	Mental He	1	stress management		Job contro	This study	Adaptatior	Shimazu A	Psychol Re	Shimazu A	Psychol Re	2004
15215660	0	0	0	Work relat	1	shift	shift	Changes in	This paper	Adult; Fem	Kaneko SY	J Occup He	Kaneko SY	J Occup He	2004
15215662	0	0	0	Career /ec	4	education	health pro	Relationsh	The preser	Adult; Age	Shimizu T,	J Occup He	Shimizu T	J Occup He	2004
15215665	0	0	0	Mental He	1	lifestyle ar	smoke anc	Relationsh	NA	Adult; Cros	Ota A, Yas	J Occup He	Ota A	J Occup He	2004
15244073	0	0	0	Mental He	1	stress mar	seeking fo	Difference	This study	Adolescen	Soeda S, K	J UOEH. 20	Soeda S	J UOEH	2004
15269897	0	0	0	Work relat	1	substance e	chemical	Were volat	This study	Adult; Air I	Takigawa	Environ To	Takigawa	Environ To	2004
15492453	0	0	0	Lifestyle re	1	lifestyle	smoke	Associatio	The assoc	Adolescen	Yamada Y	J Occup He	Yamada Y	J Occup He	2004
15536885	0	0	0	Work relat	1	shift	shift	Effect of s	This paper	Adult; Dep	Kaneko SY	Fukushima	Kaneko SY	Fukushima	2004
15539058	0	0	0	Lifestyle re	4	physical ac	sedentary	Health ber	Inactivity i	Adult; Bloc	Chan CB, I	Prev Med.	Chan CB	Prev Med	2004
15540627	0	0	0	Work relat	2	PCB, asbes	asbesto	Malignant	Malignant	Adult; Age	Kishimoto	Ind Health	Kishimoto	Ind Health	2004

15540629	0	0	0	Work relat	2	substance e	ether	Health effe	Ethylene g	Acetates; A	Wang RS,	Ind Health	Wang RS	Ind Health	2004
15613764	0	0	0	Lifestyle re	1	others	checkup u	Efficacy ar	To determ	Adult; Alar	Nomura K,	J Occup He	Nomura K	J Occup He	2004
15613769	0	0	0	Career /ec	1	satisfactio	checkup	Factors as	To improve	Adult; Deli	Kudo Y, Sa	J Occup He	Kudo Y	J Occup He	2004
15613773	0	0	0	Mental He	3	occupational stress		Relationsh	NA	Adult; Age	Takeuchi T	J Occup He	Takeuchi T	J Occup He	2004
15617394	0	0	0	Mental He	1	occupational stress		Occupation	The relatic	Adolescen	Kawakami	J Epidemic	Kawakami	J Epidemic	2004
15675537	0	0	0	Lifestyle re	1	oral health	oral	Relationsh	To identify	Adult; Cros	Chikamoto	Am J Healt	Chikamoto	Am J Healt	2005
15690483	0	0	0	Lifestyle re	3	others	NAFLD	Chronolog	The incide	Adult; Age	Suzuki A, A	Hepatolog	Suzuki A	Hepatolog	2005
15732310	0	0	0	Work relat	1	safety and v	injury	Sleep-rela	A cross-se	Accidents,	Nakata A,	Ind Health	Nakata A	Ind Health	2005
15732316	0	0	0	Lifestyle re	1	lifestyle	smoke	Associatio	For the pu	Adult; Cros	Kageyama	Ind Health	Kageyama	Ind Health	2005
15780681	0	0	0	Mental He	1	occupational stress		Temperam	This study	Adult; Ana	Sakai Y, Al	J Affect Di	Sakai Y	J Affect Di	2005
15794590	0	0	0	Work relat	1	VDT	VDT	A very low	Exposure t	8-Hydroxy	Ishihara I,	J UOEH. 20	Ishihara I	J UOEH	2005
15824476	0	0	0	Mental He	1	occupational stress		The relatic	This study	Adult; Burı	Takeda F,	J Occup He	Takeda F	J Occup He	2005
15889302	0	0	0	Work relat	1	substance e	Methyltetr	Methyltetr	To investig	Adult; Data	Yokota K,	Int Arch Oc	Yokota K	Int Arch Oc	2005
15953844	0	0	0	Mental He	1	stress mar	positive m	Effects of	Single-ses	Administra	Tsutsumi A	J Occup He	Tsutsumi A	J Occup He	2005
16039765	0	0	0	Lifestyle re	3	lifestyle	smoke	What acco	Despite th	Adult; Coh	Honjo K, T	Soc Sci Me	Honjo K	Soc Sci Me	2006
16096358	0	0	0	Work envir	3	retention	turnover	Relationsh	The preser	Adult; Foll	Shimizu T,	J Occup He	Shimizu T	J Occup He	2005
16099340	0	0	0	Lifestyle re	3	lifestyle	alcohol	Associatio	This study	Adult; Coh	Higashikav	Arch Med	Higashikav	Arch Med	2005
16174266	0	0	0	Lifestyle re	1	lifestyle	smoke	Associatio	This study	Adolescen	Yamamoto	J Clin Peric	Yamamoto	J Clin Peric	2005
16202660	0	0	0	Work relat	1	shift	shift	Cardiac au	The patho	Adult; Auto	Ishii N, Da	Auton Neu	Ishii N	Auton Neu	2005
16230834	0	0	0	Career /ec	4	satisfactio	edu	Effects of	This study	Adult; Con	Shimazu A	J Occup He	Shimazu A	J Occup He	2005
16268848	0	0	0	Work relat	3	safety and v	injury	Daytime sl	This paper	Accidents,	Suzuki K, (	J Adv Nurs	Suzuki K	J Adv Nurs	2005
16276032	0	0	0	Mental He	3	scale dev		Five-year s	The job ch	Adult; Age	Kayaba K,	J Epidemic	Kayaba K	J Epidemic	2005
16283366	0	0	0	Work envir	3	productivit	absenteeis	Job strain	The preser	Adult; Fem	Kondo K, H	Int Arch Oc	Kondo K	Int Arch Oc	2006
16294925	0	0	0	Work relat	1	PCB, asbes	lead	Compariso	Both tradit	Adult; Ami	Fukui Y, M	Ind Health	Fukui Y	Ind Health	2005
16337277	0	0	0	Mental He	3	occupational stress		Prediction	The preser	Adult; Asia	Nakao M,	J Affect Di	Nakao M	J Affect Di	2006
16369111	0	0	0	Mental He	1	occupational stress		A prospect	The purpo	Adult; Cros	Babazono	J Occup He	Babazono	J Occup He	2005
16418121	0	0	0	Mental He	1	stress management		Gender an	To examin	Adult; Anx	Nakao M,	Women He	Nakao M	Women He	2005
16430969	0	0	0	Mental He	1	occupational stress		Possible c	Oxidative/	Adult; Anti	Tsuboi H,	J Affect Di	Tsuboi H	J Affect Di	2006
16451423	0	0	0	Work and I	1	informal ca	介護者も体	The impac	The purpo	Aged; Age	Tsukasaki	Nurs Healt	Tsukasaki	Nurs Healt	2006
16477960	0	0	0	Mental He	1	occupational stress		Relationsh	The aims (	Adult; Asia	Utsugi M,	Sleep. 200	Utsugi M	Sleep	2005
16484761	0	0	0	Work relat	1	comfortab	substance	Glove sele	This study	Adult; Cho	Sasaki M,	J Occup He	Sasaki M	J Occup He	2006
16484763	0	0	0	Work envir	1	retention	Turnover	Factors af	Rapid turn	Adult; Burı	Suzuki E, I	J Occup He	Suzuki E	J Occup He	2006
16484764	0	0	0	Mental He	1	occupatior	fatigue	Relationsh	NA	Adult; Con	Takaki J, N	J Occup He	Takaki J	J Occup He	2006
16484765	0	0	0	Mental He	1	overtime a	動揺	Effect of o	NA	Adult; Data	Karita K, N	J Occup He	Karita K	J Occup He	2006
16494089	0	0	0	Work and I	1	WFC	仕事と家庭	Work and	To clarify v	Adult; Age	Sekine M,	Sleep. 200	Sekine M	Sleep	2006
16523317	0	0	0	Work envir	1	productivit	absence	Psychosoc	This study	Adult; Cros	Ishizaki M,	Int Arch Oc	Ishizaki M	Int Arch Oc	2006
16612039	0	0	0	Work relat	1	VDT	VDT	Visual disp	The preser	Adult; Con	Kubo T, M	J Occup He	Kubo T	J Occup He	2006
16633813	0	0	0	Mental He	4	stress mar	program	Effects of	To examin	Adaptatior	Shimazu A	Int Arch Oc	Shimazu A	Int Arch Oc	2006
16690187	0	0	0	Mental He	3	occupational stress		Psychosoc	We prospe	Adolescen	Tsutsumi A	Soc Sci Me	Tsutsumi A	Soc Sci Me	2006
16715999	0	0	0	Lifestyle re	1	physical ac	workload	Physical w	Maximum	Adult; Age	Hirai T, Ku	Ind Health	Hirai T	Ind Health	2006
16721522	0	0	0	Work relat	1	Musle ske	Muskel	Cervical sp	Cervical ex	Adult; Age	Takamiya	J Orthop S	Takamiya	J Orthop S	2006
16750561	0	0	0	Lifestyle re	3	lifestyle	smoke	A longitudi	To clarify t	Adult; Age	Watari M,	Prev Med.	Watari M	Prev Med	2006
16754270	0	0	0	Lifestyle re	3	LRD	lipid	Low-densi	We examir	Adult; Ami	Suwazono	Blood Pres	Suwazono	Blood Pres	2006
16758484	0	0	0	Work relat	1	safety and v	injury	Impact of	Workers in	Accidents,	Nakata A,	Am J Ind M	Nakata A	Am J Ind M	2006
16782774	0	0	0	Work relat	0	Musle ske	Muskel	Effects of	Many prev	Adult; Auto	Sakakibara	Occup Me	Sakakibara	Occup Me	2006
16788278	0	0	0	Lifestyle re	4	lifestyle	smoke	Effectiven	To test the	Adult; Fem	Tanaka H,	J Occup He	Tanaka H	J Occup He	2006
16867309	0	0	0	Work relat	1	safety and v	injury	Non-fatal	Active smo	Accidents,	Nakata A,	Soc Sci Me	Nakata A	Soc Sci Me	2006
16889628	0	0	0	Lifestyle re	1	lifestyle	smoke	Associatio	The mecha	Adolescen	Nishida N,	J Clin Peric	Nishida N	J Clin Peric	2006
16949626	0	0	0	Work and I	1	WFC	仕事と家庭	The impac	We examir	Adult; Age	Takeda Y,	Public Hea	Takeda Y	Public Hea	2006
16995564	0	0	0	Lifestyle re	1	oral health	oral	The Japan	To validate	Adult; Age	Ide R, Yam	Communit	Ide R	Communit	2006
17053303	0	0	0	Work relat	1	safety and v	injury	The preval	Workers in	Accidents,	Nakata A,	J Occup He	Nakata A	J Occup He	2006
17085931	0	0	0	Work relat	1	Musle ske	Muskel	Arm pain a	We investi	Adult; Arm	Takahashi	Ind Health	Takahashi	Ind Health	2006
17096372	0	0	0	Mental He	1	scale dev		Validity of	The Cente	Adult; Age	Wada K, T	Am J Ind M	Wada K	Am J Ind M	2007
17179642	0	0	0	Career /ec	1	satisfactio	well-being	Associatio	Although t	Adult; Cros	Ide R, Hos	J Occup He	Ide R	J Occup He	2006
17179643	0	0	0	Work envir	4	productivit	edu	Effects of	As job stre	Adult; Fem	Takao S, T	J Occup He	Takao S	J Occup He	2006
17179644	0	0	0	Work envir	1	retention	Int_Leav	Associatio	In order to	Adult; Fem	Kudo Y, Sa	J Occup He	Kudo Y	J Occup He	2006
17179646	0	0	0	Work relat	2	comfortab	chemical	Genotoxic	The aims (	Adult; Anti	Yoshida J,	J Occup He	Yoshida J	J Occup He	2006
17190723	0	0	0	Work relat	1	shift	shift	Psychosoc	Characteri	Adult; Diso	Takahashi	Chronobio	Takahashi	Chronobio	2006
17284873	0	0	0	Mental He	1	occupational stress		Job stress	Teachers a	Adult; Cros	Muto S, M	Ind Health	Muto S	Ind Health	2007
17284874	0	0	0	Career /ec	4	satisfactio	satis	Effect of li	It is import	Adult; Fem	Ohta M, Ta	Ind Health	Ohta M	Ind Health	2007
17284892	0	0	0	Mental He	1	lifestyle ar	physical ac	Associatio	This study	Adult; Dep	Wada K, S	Ind Health	Wada K	Ind Health	2007
17305210	0	0	0	Work envir	1	engagement		Factor stru	The factor	Adult; Asia	Sumi K.	Psychol Re	Sumi K	Psychol Re	2006
17314466	0	0	0	Mental He	1	lifestyle and mental h		Effect of th	Although s	Activities (	Ohta M, M	J Occup He	Ohta M	J Occup He	2007
17322556	0	0	0	Mental He	3	occupatior	fatigue	Lifestyle a	Fatigue is	Adult; Fati	Yamazaki	Occup Me	Yamazaki	Occup Me	2007
17378369	0	0	0	Lifestyle re	1	LRD	HTN	Workplace	Job strain,	Adult; Bloo	Harada K,	Hypertens	Harada K	Hypertens	2006
17396571	0	0	0	Mental He	1	occupatior	biomarker	Measuring	The purpo	Adrenocor	Kawaguch	Fukuoka Ig	Kawaguch	Fukuoka Ig	2007
17429164	0	0	0	Mental He	1	stress management		Supervisor	We investi	Adult; Ana	Mineyama	J Occup He	Mineyama	J Occup He	2007
17470019	0	0	0	Lifestyle re	3	lifestyle	smoke	Longitudin	Insufficien	Adolescen	Kibayashi	J Periodon	Kibayashi	J Periodon	2007
17471508	0	0	0	Mental He	1	occupational stress		Job stress	Associatio	Adolescen	Saijo Y, Ue	Am J Ind M	Saijo Y	Am J Ind M	2007
17476621	0	0	0	Work relat	1	Musle ske	Muskel	The preval	To estimat	Adult; Age	Toda K.	Scand J Rh	Toda K	Scand J Rh	2007
17485866	0	0	0	Work envir	1	productivit	absence	Sickness a	Associatio	Adult; Cros	Otsuka Y,	Ind Health	Otsuka Y	Ind Health	2007
17485868	0	0	0	Mental He	1	overtime a	overtime	Overtime,	To ascerta	Adult; Fati	Sasaki T, I	Ind Health	Sasaki T	Ind Health	2007
17485869	0	0	0	Mental He	1	occupational stress		Relations (	The aim of	Adult; Fem	Kawahara	Ind Health	Kawahara	Ind Health	2007
17593949	0	0	0	Mental He	1	stress mar	program	The imme	This study	Adult; Affe	Taniguchi	Acta Med	Taniguchi	Acta Med	2007
17609974	0	0	0	Mental He	1	stress management		Effort-rew	The purpo	Adult; Fati	Wada K, S	Int Arch Oc	Wada K	Int Arch Oc	2008
17650300	0	0	0	Work relat	1	Musle ske	Muskel	Prevalence	The preval	Adult; Coo	Nagasu M	BMC Publi	Nagasu M	BMC Publi	2007
17688086	0	0	0	Lifestyle re	1	diet and nu	diet	A content	The purpo	Adult; Asia	Akamatsu	Psychol Re	Akamatsu	Psychol Re	2007
17714229	0	0	0	Career /ec	1	satisfactio	profession	Factors as	This paper	Adult; Ana	Saeki K, Iz	Public Hea	Saeki K	Public Hea	2007
17721059	0	0	0	Lifestyle re	1	others	hypotensic	Investigati	The "Just-	Adult; Bloo	Kabe I, Tsu	Sangyo Eis	Kabe I	Sangyo Eis	2007

17891890	0	0	0	Work relat	1	PCB, asbes	cadmium	Changes ir	The autho	Adult; Cad	Miyamoto	Arch Envir	Miyamoto	Arch Envir	2006
17903353	0	0	0	Work relat	1	physical syr	農夫肺	High preva	In a distric	Adult; Age	Hayashi S,	Int J Immu	Hayashi S	Int J Immu	2007
17934754	0	0	0	Work relat	1	substance e	toluene	Evaluation	The purpo	Adolescen	Kawai T, U	Int Arch O	Kawai T	Int Arch O	2008
17951969	0	0	0	Lifestyle re	1	lifestyle	obesity	Degree of	The object	Adult; Age	Inoue M, T	J Occup H	Inoue M	J Occup H	2007
17980883	0	0	0	Mental He	3	occupation	biomarker	Psychosoc	The aim of	Adult; Age	Hirokawa	Atheroscle	Hirokawa	Atheroscle	2008
18000421	0	0	0	Mental He	0	stress mar	seeking fo	Factors as	The aim of	Adult; Age	Kudo Y, Sa	J Occup Er	Kudo Y	J Occup Er	2007
18075204	0	0	0	Lifestyle re	1	lifestyle	smoke	Associatio	Few studie	Adult; Cro	Hu L, Seki	J Occup H	Hu L	J Occup H	2007
18075213	0	0	0	Mental He	1	occupational stress		Poor ment	School tea	Adaptation	Nagai M, T	J Occup H	Nagai M	J Occup H	2007
18093048	0	0	0	Lifestyle re	1	lifestyle	alcohol	Estimation	In order to	Adult; Age	Suwazono	Risk Anal.	Suwazono	Risk Anal	2007
18216179	0	0	0	Lifestyle re	1	lifestyle	alcohol	Associatio	The object	Adult; Alco	Dakeishi M	Alcohol Alc	Dakeishi M	Alcohol Alc	2008
18270480	0	0	0	Mental He	1	stress management		Self-care f	In order to	Adult; Age	Ishikawa K	Sangyo Eis	Ishikawa K	Sangyo Eis	2008
18281013	0	0	0	Work relat	1	shift	shift	Work sche	Nursing ho	Adult; Card	Takahashi	Appl Ergor	Takahashi	Appl Ergor	2008
18283096	0	0	0	Lifestyle re	1	lifestyle	others	Associatio	The aim of	Adult; Alco	Wakabaya	Alcohol Alc	Wakabaya	Alcohol Alc	2008
18285650	0	0	0	Mental He	3	occupational stress		Associatio	The preser	Adult; Coh	Kawano Y.	J Occup H	Kawano Y	J Occup H	2008
18307714	0	0	0	Lifestyle re	4	lifestyle	others	Interventio	The altere	Adult; Age	Togami T.	Obes Rev.	Togami T	Obes Rev	2008
18314186	0	0	0	Mental He	1	lifestyle ar	smkoing	Active and	To assess	Adolescen	Nakata A,	Prev Med.	Nakata A	Prev Med	2008
18350748	0	0	0	Work relat	1	shift	shift	Anxiety ab	In 1999, th	Adult; Anx	Kubo T, M	J UOEH. 20	Kubo T	J UOEH	2008
18413963	0	0	0	Work relat	1	Musle ske	Muskel	Epidemiolo	A survey o	Activities c	Miyamoto	Ind Health	Miyamoto	Ind Health	2008
18413969	0	0	0	Work envir	0	organizatio	climcate	A pilot stu	To investig	Adult; Atti	Kudo Y, Sa	Ind Health	Kudo Y	Ind Health	2008
18413972	0	0	0	Mental He	1	occupation	work_env	Changes o	To investig	Adult; Emp	Haruyama	Ind Health	Haruyama	Ind Health	2008
18426454	0	0	0	Work envir	1	retention	Int_Leav	Nurses' lea	This paper	Adult; Atti	Takase M,	J Adv Nurs	Takase M	J Adv Nurs	2008
18444020	0	0	0	Mental He	1	stress management		Lagged eff	There have	Adaptation	Shimazu A	Int J Behav	Shimazu A	Int J Behav	2008
18474052	0	0	0	Mental He	1	occupation	sleep	Perceived	Psychosoc	Adolescen	Nakata A,	Communit	Nakata A	Communit	2008
18515303	0	0	0	Career /ec	1	self-effica	literacy	Developing	With the in	Adult; Con	Ishikawa H	Health Pro	Ishikawa H	Health Pro	2008
18569129	0	0	0	Mental He	1	scale dev		Improving	The effort	Adult; Age	Tsutsumi A	Int J Behav	Tsutsumi A	Int J Behav	2008
18586640	0	0	0	Lifestyle re	3	LRD	metabolic	Compariso	Most defin	Aged; Anth	Kato M, Ta	Asia Pac J	Kato M	Asia Pac J	2008
18658036	0	0	0	Lifestyle re	1	LRD	CVD	Education,	Little resea	Adult; Card	Honjo K, Is	Stroke. 20	Honjo K	Stroke	2008
18669180	0	0	0	Work envir	0	organizatio	health sup	Developme	This resea	Adult; Fem	Ariyoshi H	AAOHN J.	Ariyoshi H	AAOHN J	2008
18708087	0	0	0	Infertility,	1	pregnancy	Alcohol	Alcohol co	This study	Adult; Alco	Tamaki T,	Prev Med.	Tamaki T	Prev Med	2008
18716380	0	0	0	Mental He	1	occupation	character	Relationsh	Burnout of	Adaptation	Shimizutar	Ind Health	Shimizutar	Ind Health	2008
18716392	0	0	0	Mental He	4	occupation	internet	Evaluation	The effect	Adult; Cog	Suzuki E, T	J Occup H	Suzuki E	J Occup H	2008
18791890	0	0	0	Mental He	3	stress management		A longitudi	The purpo	Adaptation	Okabayash	Aging Men	Okabayash	Aging Men	2008
18799644	0	0	0	Mental He	1	occupation	gender	Gender-re	This article	Adult; Cro	Ogiwara C	Transcult I	Ogiwara C	Transcult I	2008
18815715	0	0	0	Mental He	3	lifestyle ar	obesity	Influence o	This study	Abdominal	Ishizaki M,	Scand J W	Ishizaki M	Scand J W	2008
18986376	0	0	0	Lifestyle re	1	lifestyle	alcohol	Influences	Alcohol dri	Adult; Age	Wakabaya	Alcohol Cli	Wakabaya	Alcohol Cli	2010
19023175	0	0	0	Work envir	4	organizatio	work envir	Effects of	The Menta	Adult; Ana	Kobayashi	J Occup H	Kobayashi	J Occup H	2008
19057116	0	0	0	Mental He	1	occupational stress		Correlates	Although t	Adult; Age	Ikeda T, N	J Occup H	Ikeda T	J Occup H	2009
19130149	0	0	0	Career /ec	1	satisfactio	medical pe	Physician	Physician	Adult; Cro	Utsugi-Oz	J Gen Inte	Utsugi-Oz	J Gen Inte	2009
19139324	0	0	0	Mental He	3	lifestyle ar	stroke and	Prospectiv	No prospe	Age Distrib	Tsutsumi A	Arch Intern	Tsutsumi A	Arch Intern	2009
19168271	0	0	0	Lifestyle re	3	LRD	CVD	Occupatio	This study	Adult; Age	Hirokawa	Soc Sci M	Hirokawa	Soc Sci M	2009
19272674	0	0	0	Work envir	1	organizatio	climate	Organizati	Although c	Accidents,	Smith DR,	Am J Infec	Smith DR	Am J Infec	2009
19367046	0	0	0	Mental He	1	occupational stress		The interre	A growing	Adult; Burn	Tokuda Y,	Ind Health	Tokuda Y	Ind Health	2009
19367047	0	0	0	Mental He	4	stress mar	program	The effect	The aim of	Adaptation	Kawahara	Ind Health	Kawahara	Ind Health	2009
19377266	0	0	0	Lifestyle re	1	LRD	glucose m	Prevalence	Undiagnos	Adult; Agin	Kawano H	Circ J. 200	Kawano H	Circ J	2009
19453864	0	0	0	Lifestyle re	1	LRD	metabolic	Associatio	Metabolic	Adult; Bod	Morita T, (	J Public He	Morita T	J Public He	2009
19467525	0	0	0	Lifestyle re	1	lifestyle	alcohol	Associatio	Alcohol int	Adult; Alco	Wakabaya	Gend Med	Wakabaya	Gend Med	2009
19483368	0	0	0	Work and	1	WFC	WLB	An examin	The aims o	Adult; Cro	Kato M, Ya	J Occup H	Kato M	J Occup H	2009
19483369	0	0	0	Mental He	4	stress mar	program	Effects of	The purpo	Adult; Fem	Umanodan	J Occup H	Umanodan	J Occup H	2009
19490133	0	0	0	Lifestyle re	0	oral health	oral	Recognitio	The purpo	Administra	Chiba Y, S	Gerodonto	Chiba Y	Gerodonto	2009
19531915	0	0	0	Career /ec	0	satisfactio	satis	Predictors	To seek th	Adolescen	Kudo Y, M	Ind Health	Kudo Y	Ind Health	2009
19531916	0	0	0	Menstruat	1	menstruat	LBP	Menstrual	An increas	Adult; Cro	Smith DR,	Ind Health	Smith DR	Ind Health	2009
19531920	0	0	0	Lifestyle re	4	lifestyle	others	Evaluation	To examin	Adult; Fem	Fujii H, Na	Ind Health	Fujii H	Ind Health	2009
19533887	0	0	0	Lifestyle re	1	lifestyle	obesity	Gender dif	The preval	Adult; Age	Sato K, Ta	Asia Pac J	Sato K	Asia Pac J	2008
19628101	0	0	0	Lifestyle re	1	diet and n	diet	Dietary int	Investigati	Adult; Age	Poudel-Ta	Nutr Res. 2	Poudel-Ta	Nutr Res	2009
19671648	0	0	0	Work envir	1	organizatio	harmony	The Japan	Harmony i	Adult; Atti	Konishi E,	Nurs Ethic	Konishi E	Nurs Ethic	2009
19745553	0	0	0	Work envir	1	retention	turnover	Solutions f	To prevent	Adult; Atti	Tsukada Y	Circ J. 200	Tsukada Y	Circ J	2009
19749493	0	0	0	Lifestyle re	2	LRD	lipid	Associatio	To examin	Adult; Age	Maruyama	J Atherosc	Maruyama	J Atherosc	2009
19834258	0	0	0	Career /ec	1	satisfactio	well-being	Is workaho	The aim of	Adaptation	Shimazu A	Ind Health	Shimazu A	Ind Health	2009
19843687	0	0	0	Career /ec	0	satisfactio	satis	The relatic	Health pro	Adult; Age	Murayama	Health Edu	Murayama	Health Edu	2010
19907109	0	0	0	Mental He	0	lifestyle and mental h		Interactive	Recent pro	Adult; Bod	Takaki J, M	J Occup H	Takaki J	J Occup H	2010
19996540	0	0	0	Work envir	1	engageme	Work_eng	Intragroup	The possit	Adult; Con	Tsuno K, K	Ind Health	Tsuno K	Ind Health	2009
19996541	0	0	0	Mental He	1	occupational stress		Associatio	To improve	Adult; Dep	Takada M,	Ind Health	Takada M	Ind Health	2009
19996543	0	0	0	Mental He	1	occupation	SOC	The assoc	Via a large	Adaptation	Tomotsun	Ind Health	Tomotsun	Ind Health	2009
20021576	0	0	0	Mental He	1	stress management		Relationsh	We aimed	Adaptation	Suzuki E, S	Jpn J Nurs	Suzuki E	Jpn J Nurs	2009
20022836	0	0	0	Lifestyle re	4	lifestyle	smoke	Efficacy of	It is well-k	Adult; Fem	Hishida A,	Cancer Ep	Hishida A	Cancer Ep	2010
20032590	0	0	0	Work relat	0	VDT	VDT	Interactive	The aims o	Autonomic	Takanishi	J Occup H	Takanishi	J Occup H	2010
20169433	0	0	0	Work envir	0	productivit	overtime	How does	The under	Adaptation	Shimazu A	Int J Behav	Shimazu A	Int J Behav	2010
20176429	0	0	0	Work envir	0	workplace	soc_cap	Does low v	While the	Adult; Age	Suzuki E, T	Soc Sci M	Suzuki E	Soc Sci M	2010
20208420	0	0	0	Career /ec	0	satisfactio	medical pe	Enhancing	Proper wo	Adult; Atti	Kudo Y, Ki	Tohoku J E	Kudo Y	Tohoku J E	2010
20216013	0	0	0	Harassme	0	bullying	bullying	Bullying (ij	Workplace	Adult; Burn	Abe K, Hen	Nurs Res.	Abe K	Nurs Res	2010
20222990	0	0	0	Mental He	1	occupational stress		National s	Physicians	Adult; Afte	Wada K, Y	BMC Publi	Wada K	BMC Publi	2010
20411221	0	0	0	Work and	3	Returntow	Returntow	Determina	To examin	Activities c	Saeki S, To	J Rehabil M	Saeki S	J Rehabil M	2010
20437815	0	0	0	Work relat	1	Musle ske	posture	Workload a	Farmers h	Adult; Age	Kumudini	J Hum Erg	Kumudini	J Hum Erg	2009
20448798	0	0	0	Lifestyle re	1	LRD	CVD	The diagno	Many epid	cerebrocar	Ohno Y, H	Vasc Healt	Ohno Y	Vasc Healt	2010
20453668	0	0	0	Lifestyle re	1	lifestyle	smoke	Associatio	Only a few	Adult; Age	Seki M, Ino	J Hyperten	Seki M	J Hyperten	2010
20465747	0	0	0	Work envir	1	retention	Turnover	Factors af	To explore	Adaptation	Suzuki E, T	J Nurs Ma	Suzuki E	J Nurs Ma	2010
20472455	0	0	0	Work relat	0	safety and	injury	Maxillofac	One hundr	Accidents,	Yamamoto	J Cranioma	Yamamoto	J Cranioma	2011
20549903	0	0	0	Mental He	4	stress mar	program	Effects of	We practic	Administra	Ikegami K,	J UOEH. 20	Ikegami K	J UOEH	2010
20561174	0	0	0	Mental He	1	overtime a	overtime	Associatio	The purpo	Adolescen	Nakashima	J Sleep Re	Nakashima	J Sleep Re	2011

20562501	0	0	0	Mental He	4	stress mar	web	Process ev	The purpo	Adult; Age	Kawai K, Y	Ind Health	Kawai K	Ind Health	2010
20562506	0	0	0	Work envir	1	retention	Int_Leav	A longitudi	This study	Attitude of	Tei-Tomin	Ind Health	Tei-Tomin	Ind Health	2010
20562512	0	0	0	Work relat	1	shift	shift	Difference	Shift work	Adult; Atti	Tanaka K,	Ind Health	Tanaka K	Ind Health	2010
20571253	0	0	0	Harassmer	1	bullying	bullying	Measuring	Workplace	Adult; Ana	Tsuno K, K	J Occup He	Tsuno K	J Occup He	2010
20599274	0	0	0	Mental He	3	lifestyle ar	alcohol an	Depressio	Depressio	Adult; Age	Ogasawara	J Affect Di	Ogasawara	J Affect Di	2011
20616458	0	0	0	Mental He	4	occupatior	sleep	Effect of b	To investig	Adult; Ana	Kakinuma	Ind Health	Kakinuma	Ind Health	2010
20616459	0	0	0	Mental He	1	occupatior	sleep	Associatio	The preser	Adaptation	Kubota K,	Ind Health	Kubota K	Ind Health	2010
20616470	0	0	0	Work relat	1	safety and v	injury	Similarity i	Near miss	Adaptation	Tanaka K,	Ind Health	Tanaka K	Ind Health	2010
20616471	0	0	0	Work and l	1	WFC	WFC	Work-fami	The aim of	Adaptation	Shimazu A	Ind Health	Shimazu A	Ind Health	2010
20638650	0	0	0	Mental He	1	overtime a	Tx	Worktime	We investi	Adult; Ana	Takahashi	Appl Ergor	Takahashi	Appl Ergor	2011
20698023	0	0	0	Work relat	2	substance e	chemical	Neuromoto	Health effe	Adolescen	Murata K,	Am J Ind M	Murata K	Am J Ind M	2010
20716334	0	0	0	Work envir	1	workplace	soc_cap	Multi-leve	Social cap	Adult; Age	Suzuki E, I	BMC Publi	Suzuki E	BMC Publi	2010
20728909	0	0	0	Lifestyle re	1	lifestyle	obesity	Lifestyle, v	To investig	Adult; Age	Inoue M, T	Public Hea	Inoue M	Public Hea	2010
20798921	0	0	0	Menstruat	0	bladder symptom		Impact of o	This study	Adult; Fem	Sako T, Inc	Int Urogyn	Sako T	Int Urogyn	2011
20823628	0	0	0	Career /ec	1	satisfactio	satis	Job satisfa	The purpo	Adult; Age	Nakata A,	Ind Health	Nakata A	Ind Health	2011
20924151	0	0	0	Work and l	3	WFC	WF	Work-fami	To examin	Adult; Coh	Shimada K	J Occup He	Shimada K	J Occup He	2010
20944438	0	0	0	Harassmer	1	bullying	bullying	Workplace	The purpo	Adult; Bull	Takaki J, T	J Occup He	Takaki J	J Occup He	2010
21041011	0	0	0	Work and l	1	WFC	WF	Sex differe	Poor physi	Adult; Age	Sekine M,	Soc Sci Me	Sekine M	Soc Sci Me	2010
21083785	0	0	0	Work and l	1	WFC	WLB	Working co	The aim of	Adult; Awa	Tanaka S,	J Clin Nurs	Tanaka S	J Clin Nurs	2011
21173522	0	0	0	Work relat	0	PCB, asbes	lead	Lead expo	In adults, l	Adult; Fem	Vigeh M, S	Ind Health	Vigeh M	Ind Health	2011
21173526	0	0	0	Menstruat	0	menstruation		Menstrual	Women's e	Adolescen	Nohara M,	Ind Health	Nohara M	Ind Health	2011
21235825	0	0	0	Lifestyle re	4	LRD	glucose m	Preventior	A randomi	Adult; Bloo	Sakane N,	BMC Publi	Sakane N	BMC Publi	2011
21265946	0	0	0	Mental He	1	occupatior	depressior	Assessmer	Our aim w	Cross-Sec	Sugawara	Psychiatry	Sugawara	Psychiatry	2011
21273736	0	0	0	Lifestyle re	1	physical ac	workload	Work form	Our previo	Acceleratio	Hirai T, Ku	Ind Health	Hirai T	Ind Health	2011
21280948	0	0	0	Work envir	1	engageme	Work_eng	Crossover	This study	Adult; Atti	Bakker AB	J Occup He	Bakker AB	J Occup He	2011
21307616	0	0	0	Lifestyle re	1	LRD	metabolic	The relatic	The aim of	Adult; Age	Yamamoto	J Physiol A	Yamamoto	J Physiol A	2011
21383384	0	0	0	Mental He	1	occupatior	overtime	Working ho	Physicians	Adult; Cros	Tomioka K	Occup Me	Tomioka K	Occup Me	2011
21431805	0	0	0	Mental He	1	occupational stress		Influences	Psycholog	Adult; Cros	Fukuda Y,	Environ He	Fukuda Y	Environ He	2012
21431810	0	0	0	Mental He	1	lifestyle and mental h		Metabolic	Evidence i	Adult; Age	Kimura Y,	Environ He	Kimura Y	Environ He	2011
21447396	0	0	0	Work relat	3	Musle ske	OAと業種	Associatio	We investi	Aged; Bon	Muraki S,	Osteoarthr	Muraki S	Osteoarthr	2011
21453548	0	0	0	Mental He	3	occupatior	SOC	Sense of c	The ability	Adult; Age	Sairenchi	BMC Publi	Sairenchi	BMC Publi	2011
21471691	0	0	0	Work envir	1	organizatio	organizatio	Developm	The purpo	Adult; Fac	Matsuda Y	J Occup He	Matsuda Y	J Occup He	2011
21476013	0	0	0	Work and l	1	WFC	WLB	Gender dif	A higher p	Adult; Care	Yoshioka E	Soc Psych	Yoshioka E	Soc Psych	2012
21514709	0	0	0	Lifestyle re	3	LRD	CVD	Impact of o	The aims o	Adolescen	Tsutsumi A	Soc Sci Me	Tsutsumi A	Soc Sci Me	2011
21530721	0	0	0	Work and l	1	Returntow	Returntow	Functional	To examin	Adolescen	Tanaka H,	Arch Phys	Tanaka H	Arch Phys	2011
21547569	0	0	0	Mental He	1	occupational stress		Prevalence	The aim of	Adult; Age	Fushimi M	Communit	Fushimi M	Communit	2012
21597232	0	0	0	Work relat	4	shift	shift	Brief morn	To investig	Adult; Circ	Tanaka K,	J Occup He	Tanaka K	J Occup He	2011
21615914	0	0	0	Work relat	1	Musle ske	付き指	Prevalence	Previous s	Adult; Con	Nagasu M	BMC Publi	Nagasu M	BMC Publi	2011
21618947	0	0	0	Work relat	2	shift		Shift work	The aim of	Adult; Cas	Li Y, Sato	Int J Occup	Li Y	Int J Occup	2011
21658347	0	0	0	Mental He	1	occupatior		Work hour	Depressio	Adolescen	Nakata A.	J Clin Psyc	Nakata A	J Clin Psyc	2011
21660970	0	0	0	Mental He	1	occupatior		Effects of	This longit	Adult; Dep	Takagishi	J Clin Psyc	Takagishi	J Clin Psyc	2011
21697622	0	0	0	Work relat	1	comfortab		Subjective	Subjective	Adult; Colo	Inaba R, O	Ind Health	Inaba R	Ind Health	2011
21697625	0	0	0	Mental He	1	occupatior	character	Factors of	Prolonged	Adult; Fac	Shimizu M	Ind Health	Shimizu M	Ind Health	2011
21733607	0	0	0	Career /ec	1	satisfactio	well-being	Workaholi	This study	Adult; Con	Shimazu A	Soc Sci Me	Shimazu A	Soc Sci Me	2011
21871600	0	0	0	Work relat	2	VDT		The impac	To evaluat	Adult; Age	Kojima T,	Am J Opht	Kojima T	Am J Opht	2011
22003348	0	0	0	Work relat	0	shift	sleep	Associatio	The aim of	Physicians	Kaneita Y,	J Clin Slee	Kaneita Y	J Clin Slee	2011
22003989	0	0	0	Mental He	1	scale dev	Psychiatric	Developm	The aim of	Adult; Burı	Yada H, Ab	Psychiatry	Yada H	Psychiatry	2011
22020011	0	0	0	Career /ec	1	self-effica	medical pe	Associatio	Nursing is	Adaptation	Uchiyama	Ind Health	Uchiyama	Ind Health	2011
22070548	0	0	0	Mental He	1	occupatior		Investigati	A cross-se	Adult; Age	Leka S, Ha	J Psychiatı	Leka S	J Psychiatı	2012
22094607	0	0	0	Work relat	1	Musle ske		Characteri	The charac	Adult; Cros	Iizuka Y, S	J Orthop S	Iizuka Y	J Orthop S	2012
22112922	0	0	0	Career /ec	0	education	medical pe	Work moti	Nursing as	Adult; Age	Kudo Y, Ki	Tohoku J E	Kudo Y	Tohoku J E	2011
22124716	0	0	0	Lifestyle re	1	LRD	glucose m	Age-deper	The purpo	Adult; Age	Wakabaya	Diabetes C	Wakabaya	Diabetes C	2012
22156317	0	0	0	Mental He	0	stress mar	seeking fo	Difficulties	The aim of	Adult; Age	Okubo S, Y	J Occup He	Okubo S	J Occup He	2012
22185894	0	0	0	Mental He	4	occupatior	sleep	Randomize	To evaluat	Adult; Beh	Kaku A, Ni	Ind Health	Kaku A	Ind Health	2012
22246538	0	0	0	Work relat	3	Musle ske		Potential r	Two-year,	Adult; Disa	Matsudair	Spine (Phi	Matsudair	Spine (Phi	2012
22293726	0	0	0	Mental He	4	occupatior	sleep	Effects of	Because p	Administra	Nishinoue	Ind Health	Nishinoue	Ind Health	2012
22301988	0	0	0	Mental He	0	occupatior		Influence o	In Japan, r	Adult; Citie	Takagishi	Ind Health	Takagishi	Ind Health	2012
22305608	0	0	0	Work relat	0	safety and v	work mane	The factor	We explore	Adult; Asia	Asaoka S,	Sleep Mec	Asaoka S	Sleep Med	2012
22317645	0	0	0	Work relat	0	Musle ske		Experimen	This study	Adolescen	Motegi N,	Work. 2012	Motegi N	Work	2012
22374551	0	0	0	Work envir	1	engageme	work_perfo	Impact of v	Most studi	Adult; Age	Tsuchiya M	Psychiatry	Tsuchiya M	Psychiatry	2012
22418273	0	0	0	Career /ec	1	satisfactio		Results of	To evaluat	Adult; Dep	Kawada T,	J Occup Er	Kawada T	J Occup Er	2012
22463500	0	0	0	Work envir	1	workplace	sense of c	Associatio	Globally, n	Adult; Age	Ozaki K, M	BMC Publi	Ozaki K	BMC Publi	2012
22522151	0	0	0	Lifestyle re	3	LRD	CVD	Effectiven	Analysis o	Education:	Honjo K, Is	J Epidemic	Honjo K	J Epidemic	2012
22523018	0	0	0	Career /ec	1	satisfactio		100-point	Job satisfa	Adult; Cros	Kawada T,	Work. 2012	Kawada T	Work	2012
22532849	0	0	0	Work envir	4	productivit	presentee	Telephone	Subthresh	Adult; Cog	Furukawa	PLoS One.	Furukawa	PLoS One	2012
22547121	0	0	0	Mental He	1	overtime a		Relationsh	To test the	Adult; Cros	Amagasa T	J Occup Er	Amagasa T	J Occup Er	2012
22562520	0	0	0	Work envir	3	engageme		Job demar	Research o	Adult; Effic	Inoue A, K	Int Arch Oc	Inoue A	Int Arch Oc	2013
22608035	0	0	0	Mental He	1	occupatior	SOC	Sense of c	Job stress	Adult; Anx	Urakawa K	BMC Res I	Urakawa K	BMC Res I	2012
22616854	0	0	0	Lifestyle re	3	LRD	CVD	Determina	Arterial sti	Adult; Age	Mitani S, F	Blood Pres	Mitani S	Blood Pres	2012
22651100	0	0	0	Lifestyle re	1	LRD	metabolic	Long work	The link be	Adult; Age	Kobayashi	BMC Publi	Kobayashi	BMC Publi	2012
22673364	0	0	0	Work envir	1	engageme		Do workah	This study	Adult; Beh	Shimazu A	Ind Health	Shimazu A	Ind Health	2012
22721815	0	0	0	Lifestyle re	1	others	headache	Headache	Headache:	Adult; Age	Sato K, Ha	Public Hea	Sato K	Public Hea	2012
22776808	0	0	0	Work envir	1	workplace	workplace	The effect	To investig	Adolescen	Eguchi H,	J Occup Er	Eguchi H	J Occup Er	2012
22791208	0	0	0	Work relat	1	comfortab	work_env	Workers' lo	Many kitch	Adult; Fem	Matsuzuki	Environ He	Matsuzuki	Environ He	2013
22823222	0	0	0	Work envir	1	retention	turnover	The relatic	The aim of	Adult; Age	Niitsuma M	J Nurs Mar	Niitsuma M	J Nurs Mar	2012
22835844	0	0	0	Mental He	1	stress mar		Coping bel	In Japan, t	Adaptation	Sugawara	J Affect Di	Sugawara	J Affect Di	2012
22929793	0	0	0	Mental He	3	overtime a		Sleep, fati	We examir	Adult; Dep	Takahashi	J Occup Er	Takahashi	J Occup Er	2012
22995447	0	0	0	Work relat	1	Musle ske		A survey o	This study	Adult; Age	Nishiyama	Head Face	Nishiyama	Head Face	2012
23047077	0	0	0	Lifestyle re	1	LRD	CVD	Occupatio	The aim of	Adolescen	Morikawa	Ind Health	Morikawa	Ind Health	2012

23054146	0	0	0	Mental He	1	occupatior	depressior	Prevalence	This study	Adult; Age	Fushimi M	Communit	Fushimi M	Communit	2013
23095329	0	0	0	Work and	0	Returntow	depressior	Recurrenc	There have	Adult; Dep	Endo M, H	Ind Health	Endo M	Ind Health	2013
23096020	0	0	0	Work relat	3	substance e	toner	Distributio	Interleukin	Adult; C-R	Murase T,	Clin Chem	Murase T	Clin Chem	2013
23100333	0	0	0	Lifestyle re	3	lifestyle	smoke	smoking p	To investig	Adult; Age	Sakata R,	BMJ. 2012	Sakata R	BMJ	2012
23196772	0	0	0	Work envir	1	retention	turnover	Factors rel	This study	Adult; Atti	Tei-Tomin	Environ He	Tei-Tomin	Environ He	2013
23242836	0	0	0	Mental He	3	occupatior	sleep	Workaholis	This study	Adaptatior	Kubota K,	Int J Behav	Kubota K	Int J Behav	2014
23266430	0	0	0	Mental He	1	occupatior	character	Job stress	According	Adult; Anx	Kikuchi Y,	Occup Me	Kikuchi Y	Occup Me	2013
23279709	0	0	0	Work relat	4	comfortab	work_env	Efficacy of	To evaluat	dry eye; ne	Hirayama	Acta Ophtl	Hirayama	Acta Ophtl	2013
23338055	0	0	0	Work relat	1	Musle ske		Associatio	To examin	Adaptatior	Fujii T, Ma	Mod Rheu	Fujii T	Mod Rheu	2013
23365114	0	0	0	Work relat	1	safety and	near miss	Effects on	High levels	Adult; Fati	Kubo T, Ta	Occup Me	Kubo T	Occup Me	2013
23383289	0	0	0	Lifestyle re	1	lifestyle	smoke	Nicotine d	Given the	Adult; Cos	Nakamura	PLoS One.	Nakamura	PLoS One	2013
23385118	0	0	0	Career /ec	4	education	health edu	Change in	The aim of	Adult; Age	Haruyama	J Occup He	Haruyama	J Occup He	2013
23385430	0	0	0	Mental He	1	occupatior		Job dissati	Although s	Adult; Fati	Tatsuse T,	Ind Health	Tatsuse T	Ind Health	2013
23385436	0	0	0	Work relat	1	Musle ske		Risk factor	This study	Adult; Bod	Tomita S,	Ind Health	Tomita S	Ind Health	2013
23411666	0	0	0	Harassmer	1	violence ar		Assessing	The object	Adult; Age	Moreno M	J Occup He	Moreno M	J Occup He	2013
23418435	0	0	0	Work and	1	WFC	Psychiatric	Nationwid	Psychiatry	Adult; Bur	Umene-Na	PLoS One.	Umene-Na	PLoS One	2013
23453038	0	0	0	Mental He	1	lifestyle ar		Green tea	To examin	Adult; Age	Pham NM,	Public Hea	Pham NM	Public Hea	2014
23475018	0	0	0	Mental He	4	lifestyle and mental h		The effect	The effect	Adult; Dep	Ikenouchi-	J UOEH. 20	Ikenouchi-	J UOEH	2013
23478367	0	0	0	Career /ec	0	education	profession	Self-perce	The purpo	Adult; Emp	Yaeda J, K	Work. 2013	Yaeda J	Work	2013
23648771	0	0	0	Mental He	1	occupatior		A cross-se	A cross-se	Adaptatior	Suzumura	Ind Health	Suzumura	Ind Health	2013
23677520	0	0	0	Work and	3	Returntow	stroke	Functional	This study	Adult; Age	Tanaka H,	Int Arch Oc	Tanaka H	Int Arch Oc	2014
23719865	0	0	0	Career /ec	1	self-effica	coping	Sociocultu	Although t	Adaptatior	Morimoto	Int J Behav	Morimoto	Int J Behav	2014
23735086	0	0	0	Mental He	1	scale dev		Developme	To develop	Adult; Atti	Ogasawara	Jpn J Nurs	Ogasawara	Jpn J Nurs	2013
23741386	0	0	0	Lifestyle re	1	LRD	glucose m	Plant oils	Fatty acid	Adolescen	Kurotani K	PLoS One.	Kurotani K	PLoS One	2013
23803438	0	0	0	Mental He	1	occupatior		Occupatio	High distre	Brief Job S	Ito S, Fujit	Work. 2014	Ito S	Work	2014
23812028	0	0	0	Harassmer	1	prejudice		Hepatitis s	Laboratory	Adult; Age	Sasaki N,	J Occup He	Sasaki N	J Occup He	2014
23855754	0	0	0	Career /ec	1	education	medical pe	Nursing pr	The purpo	Japan; PES	Anzai E, D	Nurs Healt	Anzai E	Nurs Healt	2014
23860736	0	0	0	Lifestyle re	3	physical ac		Persistenc	No prior in	Age Factor	Kumagai N	Eur J Healt	Kumagai N	Eur J Healt	2014
23874644	0	0	0	Harassmer	1	stigma		Associatio	The stigma	Adult; Age	Wang G, W	PLoS One.	Wang G	PLoS One	2013
23892637	0	0	0	Mental He	1	scale dev		Optimum c	The theore	Adolescen	Kurioka S,	J Occup He	Kurioka S	J Occup He	2014
23892903	0	0	0	Mental He	1	occupatior		Psychosoc	Recent epi	Adult; Cros	Shimazu A	Ind Health	Shimazu A	Ind Health	2013
23912205	0	0	0	Lifestyle re	1	lifestyle	alcohol	Occupatio	Problem d	Adolescen	Hasegawa	Ind Health	Hasegawa	Ind Health	2013
23924827	0	0	0	Mental He	3	overtime a		Relationsh	To clarify I	Adult; Dep	Amagasa T	J Occup Er	Amagasa T	J Occup Er	2013
23930793	0	0	0	Mental He	1	occupatior		Employme	This study	Adult; Con	Sakurai K,	Arch Envir	Sakurai K	Arch Envir	2014
23955653	0	0	0	Work relat	1	Musle ske	cost	Estimates	Little is rep	Adult; Amb	Itoh H, Kit	Ind Health	Itoh H	Ind Health	2013
23982304	0	0	0	Career /ec	1	education	medical pe	Associatio	The aim of	Adult; Clin	Tanaka A,	Environ He	Tanaka A	Environ He	2014
24071921	0	0	0	Harassmer	1	bullying		Associatio	The aim of	Adolescen	Takaki J, T	Int J Enviro	Takaki J	Int J Enviro	2013
24086457	0	0	0	Mental He	1	overtime a		Workaholis	Although it	Absenteeis	Matsudair	PLoS One.	Matsudair	PLoS One	2013
24086765	0	0	0	Harassmer	1	prejudice		Knowledge	Prejudice a	Adult; Age	Eguchi H,	PLoS One.	Eguchi H	PLoS One	2013
24136401	0	0	0	Mental He	1	occupatior	suicidal ide	Insomnia s	Although p	Adult; Age	Kato T.	Int J Behav	Kato T	Int J Behav	2014
24225401	0	0	0	Work relat	1	safety and		How do en	This study	Employme	Sakurai K,	Public Hea	Sakurai K	Public Hea	2013
24312268	0	0	0	Lifestyle re	0	lifestyle	smoke	Relationsh	Chronic ob	Adult; Air;	Horie M, N	PLoS One.	Horie M	PLoS One	2013
24317383	0	0	0	Mental He	1	occupatior	biomarker	Associatio	Some rese	8-Hydroxy	Takaki J.	Int J Enviro	Takaki J	Int J Enviro	2013
24429518	0	0	0	Work relat	1	safety and		Burn and c	To clarify t	Adolescen	Haruyama	Ind Health	Haruyama	Ind Health	2014
24430841	0	0	0	Menstruat	1	breast ca	breast can	The impac	Breast car	Adult; Brea	Saito N, Ta	J Occup He	Saito N	J Occup He	2014
24446725	0	0	0	Work relat	0	safety and		Effect of m	We studied	Adult; Con	Sano H, Sh	Dermatol S	Sano H	Dermatol S	2014
24451614	0	0	0	Work envir	0	productivit	presenteei	Psychome	The 25-ite	Adult; Age	Kono Y, M	J Occup Er	Kono Y	J Occup Er	2014
24460599	0	0	0	Lifestyle re	1	LRD	metabolic	Effect of p	The purpo	metabolic	Hwang WJ	Jpn J Nurs	Hwang WJ	Jpn J Nurs	2014
24477176	0	0	0	Harassmer	1	mistreatm		The percep	Previous s	Adult; Care	Yasukawa	Tohoku J E	Yasukawa	Tohoku J E	2014
24477857	0	0	0	Work relat	1	substance e		Prevalence	We aimed	Adult; Fem	Cui X, Lu X	Environ He	Cui X	Environ He	2014
24481035	0	0	0	Work envir	1	workplace	sense of c	Reliability,	The purpo	Adult; Fem	Takaki J, T	Int J Enviro	Takaki J	Int J Enviro	2014
24492763	0	0	0	Mental He	1	scale dev		Developme	This study	Adult; Fem	Inoue A, K	Ind Health	Inoue A	Ind Health	2014
24498248	0	0	0	Work envir	1	workplace		The bright	A growing	Adolescen	Kobayashi	PLoS One.	Kobayashi	PLoS One	2014
24584219	0	0	0	Work envir	1	engageme		Work perfo	The Work	Adult; Chro	Takegami	J Occup He	Takegami	J Occup He	2014
24602100	0	0	0	Work envir	1	productivit		Greater los	Gastro-eso	gastro-eso	Suzuki H, I	Neurogast	Suzuki H	Neurogast	2014
24628714	0	0	0	Mental He	1	occupatior	actigraphy	Occupatio	Occupatio	health disp	Takahashi	J Sleep Re	Takahashi	J Sleep Re	2014
24642575	0	0	0	Work envir	1	workplace		Roles of ex	Social cap	Adult; Age	Takemura	PLoS One.	Takemura	PLoS One	2014
24643384	0	0	0	Work envir	3	productivit	absenteeis	Risk factor	Depressio	Absenteeis	Endo M, M	Int Arch Oc	Endo M	Int Arch Oc	2015
24696043	0	0	0	Work envir	1	engageme		Workaholis	This study	Adult; Cros	Shimazu A	Int J Behav	Shimazu A	Int J Behav	2015
24739372	0	0	0	Work relat	1	Musle ske		Effort-rew	To clarify e	Adult; Age	Yokoyama	J Occup He	Yokoyama	J Occup He	2014
24806039	0	0	0	Career /ec	1	education	medical pe	Barriers to	As Japan's	Adult; Care	Mizuno-Le	Workplace	Mizuno-Le	Workplace	2014
24844530	0	0	0	Mental He	4	stress mar		Effects of	The purpo	Adult; Cog	Imamura K	PLoS One.	Imamura K	PLoS One	2014
24894127	0	0	0	Mental He	1	occupatior	Psychiatric	Difference	In psychiat	mental hea	Yada H, Ab	Int J Ment	Yada H	Int J Ment	2014
24955876	0	0	0	Work and	1	informal ca	介護者は不	The assoc	It is widely	Family car	Oshio T.	Soc Sci Me	Oshio T	Soc Sci Me	2014
25060410	0	0	0	Infertility,	1	pregnancy	Working	Long work	Previous s	Abortion, T	Takeuchi M	BMC Preg	Takeuchi M	BMC Preg	2014
25055848	0	0	0	Career /ec	1	satisfactio		Socioecon	This study	Adult; Emp	Sekine M,	Ind Health	Sekine M	Ind Health	2014
25065962	0	0	0	Career /ec	1	satisfactio	medical pe	Developme	This study	job satisfa	Muya M, K	Jpn J Nurs	Muya M	Jpn J Nurs	2014
24975108	0	0	0	Mental He	1	scale dev		Developme	This study	Adult; Fem	Inoue A, K	Ind Health	Inoue A	Ind Health	2014
25148581	0	0	0	Mental He	1	occupatior	Psychiatric	Relationsh	Psychiatric	Burnout; G	Yoshizawa	Arch Envir	Yoshizawa	Arch Envir	2016
25168926	0	0	0	Lifestyle re	1	diet and nu	diet	Low intake	Although v	Adult; Age	Katagiri R,	J Occup He	Katagiri R	J Occup He	2014
25180410	0	0	0	Harassmer	0	stigma		Stigma tov	In the pres	Adult; Atti	Tei-Tomin	Int J Ment	Tei-Tomin	Int J Ment	2014
25196795	0	0	0	Work relat	1	Musle ske	Muskel	The preval	Low back	Adolescen	Inoue G, M	J Orthop S	Inoue G	J Orthop S	2015
25197345	0	0	0	Work relat	1	occupatior		Different c	Recently, c	DNA dama	Sato Y, Ku	Int J Clin E	Sato Y	Int J Clin E	2014
25224330	0	0	0	Work relat	1	comfortab	work_env	Prevalence	In Japan, t	Adult; Cros	Inaba R, H	Ind Health	Inaba R	Ind Health	2015
25224331	0	0	0	Work relat	0	substance e	N,N-dimet	Occupatio	We evalua	Acetylcyst	Miyauchi H	Ind Health	Miyauchi H	Ind Health	2014
25228138	0	0	0	Mental He	1	occupatior	biomarker	The effort-	We examin	Adult; Circ	Ota A, Ma	Sci Rep. 20	Ota A	Sci Rep	2014
25229210	0	0	0	Mental He	1	overtime a		Associatio	Long work	Cross-sect	Imai T, Ku	Chronobio	Imai T	Chronobio	2014
25251576	0	0	0	Work relat	1	shift	BMI, 1575	Associatio	Higher boc	Adult; Bod	Tada Y, Ka	Obesity (S	Tada Y	Obesity (S	2014
25263457	0	0	0	Career /ec	1	satisfactio	medical pe	Inverse rol	Emotional	emotional	Tsukamoto	Psychol He	Tsukamoto	Psychol He	2015

25280807	0	0	0	Lifestyle re	1	oral health	oral	Early life-c	We examir	OCCUPAT	Tsuboya T	BMJ Open	Tsuboya T	BMJ Open	2014
25298232	0	0	0	Work relat	1	Musle ske	Posture	Standing p	Varicose v	body mass	Kohno K, I	J Dermato	Kohno K	J Dermatol	2014
25340520	0	0	0	Work envir	3	productivit	presentee	Optimal cu	Sickness a	Absentees	Suzuki T, I	PLoS One.	Suzuki T	PLoS One	2014
25362516	0	0	0	Work envir	1	engageme		Work enga	Evidence c	Adolescen	Eguchi H,	Int Arch O	Eguchi H	Int Arch O	2015
25374422	0	0	0	Work and l	1	WFC		Sex differe	As the nur	Adult; Age	Fujimura Y	J Occup H	Fujimura Y	J Occup H	2014
25382384	0	0	0	Harassmen	1	bullying		Multifacto	This study	Adult; Bull	Tsuno K, K	Ind Health	Tsuno K	Ind Health	2015
25421915	0	0	0	Work envir	1	retention	turnover	Investigati	The aim of	affective o	Takase M,	J Clin Nurs	Takase M	J Clin Nurs	2015
25443978	0	0	0	Mental He	1	occupation	gender	Age and ge	Depressio	Adult; Dep	Sugawara	Compr Psy	Sugawara	Compr Psy	2015
25466661	0	0	0	Infertility,	1	pregnancy	depression	Dietary vita	Although t	Depressive	Miyake Y,	Nutrition. 2	Miyake Y	Nutrition	2015
25472019	0	0	0	Work envir	1	workplace		Change in	Research c	Adolescen	Tsuboya T	Occup Env	Tsuboya T	Occup Env	2015
25475095	0	0	0	Work and l	1	WFC		Impact of v	This study	Adaptation	Makabe S,	Ind Health	Makabe S	Ind Health	2015
25500796	0	0	0	Work relat	1	substance e		The correl	This study	Adult; Aut	Cui X, Lu X	Environ He	Cui X	Environ He	2015
25503892	0	0	0	Mental He	1	occupation		Effects of	Days off, c	Adult; Bur	Saijo Y, Ch	Int J Occup	Saijo Y	Int J Occup	2014
25543127	0	0	0	Lifestyle re	4	lifestyle	alcohol	Brief interv	To investig	Adult; Alco	Ito C, Yuz	Alcohol Alc	Ito C	Alcohol Alc	2015
25550077	0	0	0	Mental He	3	occupation		Do time-in	It is well k	Fixed effec	Oshio T, T	Soc Sci Me	Oshio T	Soc Sci Me	2015
25556337	0	0	0	Mental He	1	occupation	SOC	Sense of c	Sense of c	Adult; Cro	Morita Y, C	Int J Occup	Morita Y	Int J Occup	2014
25562115	0	0	0	Mental He	4	stress mar		Does Inter	In this stu	Depressio	Imamura K	Psychol M	Imamura K	Psychol M	2015
25563547	0	0	0	Work envir	1	productivit		Work prod	The aim of	Absentees	Asami Y, G	J Occup Er	Asami Y	J Occup Er	2015
25572760	0	0	0	Lifestyle re	1	LRD	metabolic	Serum insu	The aim of	Adult; Bior	Kawada T,	Diabetes M	Kawada T	Diabetes M	2011
25592007	0	0	0	Lifestyle re	1	LRD	HTN	High dieta	Acid-base	Acid-base	Akter S, Eg	Nutrition. 2	Akter S	Nutrition	2015
25634811	0	0	0	Career /ec	4	education	health edu	Mortality b	This study	Adult; Fem	Hoshuyam	J Occup Er	Hoshuyam	J Occup Er	2015
25739406	0	0	0	Work relat	1	substance e	organic so	Compariso	The preser	Adult; Air I	Kawai T, T	J Occup H	Kawai T	J Occup H	2015
25740675	0	0	0	Work envir	4	productivit	CBT	Effect of a	Efforts to i	Adult; Cog	Kimura R,	J Occup H	Kimura R	J Occup H	2015
25749132	0	0	0	Work envir	4	engageme	CBT	Effects of	This study	Adolescen	Imamura K	J Occup Er	Imamura K	J Occup Er	2015
25751252	0	0	0	Harassmen	1	bullying		Socioecon	Bullying in	Adult; Bull	Tsuno K, K	PLoS One.	Tsuno K	PLoS One	2015
25752657	0	0	0	Work relat	1	shift	depression	Analysis o	The aim of	Adult; Ana	Baba M, O	J Occup H	Baba M	J Occup H	2015
25752660	0	0	0	Work relat	1	VDT		Screening	The aim of	Adult; Con	Kawashim	J Occup H	Kawashim	J Occup H	2015
25808047	0	0	0	Career /ec	1	satisfactio		Construct	We develo	Dialysis; J	Gu X, Itoh	Ther Aphe	Gu X	Ther Aphe	2015
25817285	0	0	0	Mental He	1	occupation		Gender dif	The purpo	Adaptation	Kataoka M	J Med Inve	Kataoka M	J Med Inve	2015
25879720	0	0	0	Work envir	1	productivit	presentee	Relationsh	Absence d	Depressio	Suzuki T, I	J Affect Di	Suzuki T	J Affect Di	2015
25885051	0	0	0	Mental He	1	occupation		Mental hea	Psychiatri	Adult; Bur	Koreki A, I	BMC Res I	Koreki A	BMC Res I	2015
25968004	0	0	0	Mental He	4	stress mar	internet	Effects of	The aim of	OCCUPAT	Imamura K	BMJ Open	Imamura K	BMJ Open	2015
25986042	0	0	0	Work relat	0	substance e	cyclohexar	Evaluation	The aim of	Adolescen	Takeuchi A	J Occup H	Takeuchi A	J Occup H	2015
26007632	0	0	0	Mental He	1	lifestyle ar		Fish consu	Depressio	Adult; Anir	Yoshikawa	Lipids Hea	Yoshikawa	Lipids Hea	2015
26024382	0	0	0	Work envir	1	engageme		The Assoc	Many prev	Adult; Cro	Shiba K, N	PLoS One.	Shiba K	PLoS One	2015
26038222	0	0	0	Career /ec	1	satisfactio		Relationsh	To examin	Health-rel	Kawabe Y,	Qual Life F	Kawabe Y	Qual Life F	2015
26043897	0	0	0	Work and l	3	workstyle		Employme	Few studie	EMPLOYM	Honjo K, Is	J Epidemic	Honjo K	J Epidemic	2015
26049807	0	0	0	Work and l	1	informal ca	介護者は不	How is an	The provis	Caregiver;	Oshio T.	Qual Life F	Oshio T	Qual Life F	2015
26058488	0	0	0	Lifestyle re	1	diet and n	diet	Associatio	Diet may i	Adipokines	Kashino I,	Nutr J. 201	Kashino I	Nutr J	2015
26225722	0	0	0	Lifestyle re	1	LRD	HTN	Achieveme	Few studie	Achieveme	Kudo N, Yo	PLoS One.	Kudo N	PLoS One	2015
26228520	0	0	0	Mental He	1	overtime and mental		Long work	Long work	Adult; Cro	Bannai A,	J Occup H	Bannai A	J Occup H	2015
26285404	0	0	0	Mental He	1	occupation	SOC	Characteri	The recog	Adult; Atti	Miyata C,	Collegian.	Miyata C	Collegian	2015
26314503	0	0	0	Mental He	1	lifestyle and mental h		Overeating	This study	Late dinne	Suzuki A, S	Obes Res	Suzuki A	Obes Res	2016
26318185	0	0	0	Work and l	3	Cancer	Returntow	Returning	More emp	Cancer su	Endo M, H	J Cancer S	Endo M	J Cancer S	2016
26320729	0	0	0	Mental He	1	stress mar		Communic	When com	Adult; Con	Honda A, I	Ind Health	Honda A	Ind Health	2016
26335001	0	0	0	Work envir	1	engageme		Grit and W	Grit, defin	Achieveme	Suzuki Y, T	PLoS One.	Suzuki Y	PLoS One	2015
26345177	0	0	0	Work envir	1	productivit	presentee	Relationsh	The object	Absentees	Doki S, Sa	J Occup H	Doki S	J Occup H	2015
26345178	0	0	0	Work envir	1	productivit		Developme	The purpo	Adult; Fem	Fujino Y, L	J Occup H	Fujino Y	J Occup H	2015
26384967	0	0	0	Mental He	3	lifestyle ar		Associatio	Leisure-tir	Adult; Coh	Kuwahara	Int J Behav	Kuwahara	Int J Behav	2015
26385476	0	0	0	Lifestyle re	1	physical ac		Socioecon	The aim of	Adult; Age	Matsushita	BMC Publi	Matsushita	BMC Publi	2015
26404528	0	0	0	Career /ec	1	career	medical pe	Mentorshi	Women ha	Adult; Fem	Yorozuya I	World J Su	Yorozuya I	World J Su	2016
26467034	0	0	0	Work and l	1	informal ca	介護者は不	Elder Care	Japan's po	Adult; Age	Kikuzawa	J Cross Cu	Kikuzawa	J Cross Cu	2015
26482027	0	0	0	Lifestyle re	3	LRD	glucose m	One-hour p	To test the	Adult; Asia	Oka R, Aiz	Diabet Me	Oka R	Diabet Me	2016
26482963	0	0	0	Lifestyle re	3	LRD	metabolic	Relationsh	To examin	Adults; Jap	Toga S, Fu	Diabetes M	Toga S	Diabetes M	2016
26690187	0	0	0	Work relat	1	physical sy	allergy	Asthma an	Although a	ECRHS; as	Kurai J, Wa	Int J Enviro	Kurai J	Int J Enviro	2015
26693748	0	0	0	Lifestyle re	3	lifestyle	obesity	Cohort stu	Overweigh	Adult; Coh	Sawada K,	Asia Pac J	Sawada K	Asia Pac J	2016
26722982	0	0	0	Work envir	1	workplace		Null assoc	Research c	Fixed effec	Tsuboya T	Soc Sci Me	Tsuboya T	Soc Sci Me	2016
26729388	0	0	0	Work and l	3	Returntow	stroke	Sickness a	The object	OCCUPAT	Endo M, S	BMJ Open	Endo M	BMJ Open	2016
26781691	0	0	0	Work envir	1	engageme		Effects of	Home-visit	coordinatio	Naruse T,	Jpn J Nurs	Naruse T	Jpn J Nurs	2016
26822016	0	0	0	Mental He	1	occupation	suicide	Associatio	This study	Japanese;	Otsuka Y,	Int J Behav	Otsuka Y	Int J Behav	2016
26829974	0	0	0	Work relat	1	Musle ske	Muskel	Potential r	Katakori is	Adult; Con	Sawada T,	Ind Health	Sawada T	Ind Health	2016
26830072	0	0	0	Harassmen	1	mistreatm		Suturing tl	In Japan, g	Adult; Fem	Okoshi K,	Surgery. 2	Okoshi K	Surgery	2016
26841020	0	0	0	Work envir	3	engagement		Work Enga	This study	Adult; Dep	Imamura K	PLoS One.	Imamura K	PLoS One	2016
26853102	0	0	0	Lifestyle re	1	physical ac	sedentary	Percentag	Does askir	Adult; Fem	Matsuo T,	J Epidemic	Matsuo T	J Epidemic	2016
26875520	0	0	0	Lifestyle re	1	LRD	lipid	A Proposa	The Japan	Adolescen	Saiki Y, Ot	J Atherosc	Saiki Y	J Atherosc	2016
26951053	0	0	0	Lifestyle re	3	physical ac		Leisure-tir	Data are li	Asians; Co	Kuwahara	Endocrine.	Kuwahara	Endocrine	2016
26995853	0	0	0	Harassmen	1	violence ar	aggression	Aggression	Aggression	Adult; Agg	Kanchika I	Osaka City	Kanchika I	Osaka City	2015
27021060	0	0	0	Mental He	1	occupation		Relationsh	Japanese t	Adult; Cro	Nakada A,	Ind Health	Nakada A	Ind Health	2016
27040063	0	0	0	Work relat	1	physical sy	allergy	Prevalence	Although a	Adult; Age	Watanabe	J Med Inve	Watanabe	J Med Inve	2016
27042045	0	0	0	Work envir	1	productivit		Relationsh	The aim of	absentees	Onoue A, C	Int J Chror	Onoue A	Int J Chror	2016
27050819	0	0	0	Lifestyle re	2	lifestyle	smoke	Disparity a	Monitoring	Adult; Cas	Tabuchi T,	PLoS One.	Tabuchi T	PLoS One	2016
27072898	0	0	0	Work and l	1	Cancer	Returntow	Co-worker	This study	Adult; Can	Eguchi H,	Psychoonc	Eguchi H	Psychoonc	2017
27102432	0	0	0	Mental He	1	overtime and mental		Psychosoc	Few studie	Extrinsic n	Watanabe	Int J Behav	Watanabe	Int J Behav	2016
27108643	0	0	0	Mental He	1	occupation	JCS	Validation	The aim of	Adult; Cro	Eguchi H,	J Occup H	Eguchi H	J Occup H	2016
27109836	0	0	0	Work relat	1	comfortab	gas	Long-term	Sulfur mus	Chemical v	Nishimura	Inhal Toxic	Nishimura	Inhal Toxic	2016
27184205	0	0	0	Work envir	3	workplace		Source-sp	This study	C-reactive	Eguchi H,	Am J Ind M	Eguchi H	Am J Ind M	2016
27276005	0	0	0	Career /ec	1	career	medical pe	Factors As	Specialty t	Adult; Age	Chatani Y,	Acad Med.	Chatani Y	Acad Med	2016
27281916	0	0	0	Lifestyle re	1	lifestyle	others	EFFECTS O	The object	Adult; Age	Nomoto M	J Hum Erg	Nomoto M	J Hum Erg	2016
25818268	0	0	0	Career /ec	6	career	medical pe	The difficu			Nomura K,	BMJ Open	Nomura K	BMJ Open	2015

27760893	0	0	0	Infertility,	3	pregnancy	dual-earne	The effect	This study		Eguchi H,	Ind Health	Eguchi H	Ind Health	2016
27862944	0	0	0	Work and	1	WFC	childrening	Factors rel	Aim: Burn		Takayama	Jpn J Nurs	Takayama	Jpn J Nurs	2017
27287238	0	0	0	Work and	1	informal c	介護者は不	Impact of	This study	Alzheimer	Goren A, N	BMC Geria	Goren A	BMC Geria	2016
27383562	0	0	0	Harassme	1	bullying		Workplace	To explore	healthy wo	Yokoyama	J Clin Nurs	Yokoyama	J Clin Nurs	2016
27389797	0	0	0	Work envir	3	productivit		Prospectiv	This study	Absentee	Fujino Y, S	J Occup Er	Fujino Y	J Occup Er	2016
27392676	0	0	0	Mental He	1	occupation		A cross-se	Opportunit	Burnout; C	Higuchi Y,	BMC Publi	Higuchi Y	BMC Publi	2016
27405459	0	0	0	Mental He	1	lifestyle ar		Associatio	Regular ph	Depressiv	Yoshikawa	BMC Publi	Yoshikawa	BMC Publi	2016
27473230	0	0	0	Work and	1	Returntow		Return to	Gynecolog	Gynecolog	Nakamura	BMC Canc	Nakamura	BMC Canc	2016
27488045	0	0	0	Work envir	1	workplace		Buffering	The prese	Adult; Cro	Inoue A, K	J Occup He	Inoue A	J Occup He	2016
27507645	0	0	0	Career /ec	1	education	medical pe	Questionn	Among you	Motivation	Kaibori M,	J Hepatobi	Kaibori M	J Hepatobi	2016
27523777	0	0	0	Work relat	1	physical sy	allergy	Self-repor	Dental wo	alcohol-ba	Minamoto	Contact D	Minamoto	Contact D	2016
27549086	0	0	0	Work and	1	informal c		How do so	It is well k	Informal c	Oshio T, K	Health Qu	Oshio T	Health Qu	2016
27549798	0	0	0	Work envir	2	productivit	absenteeis	Absentee	The aim of	Absentee	Doki S, Sa	Int Arch O	Doki S	Int Arch O	2016
27604809	0	0	0	Lifestyle r	3	LRD	metabolic	Weight gai	We examir	adult; lifes	Toga S, Fu	J Health P	Toga S	J Health P	2018
27608280	0	0	0	Mental He	3	overtime a		Fatigue an	This obser	Actigraphy	Kubo T, Ta	J Occup Er	Kubo T	J Occup Er	2016
27633655	0	0	0	Lifestyle r	1	diet and n	diet	Associatio	Long-chai	Depressiv	Yoshikawa	Lipids Hea	Yoshikawa	Lipids Hea	2016
27725562	0	0	0	Work envir	1	organizatio		Organizati	We investi	Adult; Burn	Taka F, No	Ind Health	Taka F	Ind Health	2016
27776553	0	0	0	Work envir	4	engageme	a job craft	Effects of	Job craftin	Interventic	Sakuraya A	BMC Psyc	Sakuraya A	BMC Psyc	2016
27797982	0	0	0	Mental He	4	lifestyle ar		Does subj	The purpo	PSQI; Slee	Hori H, Ike	BMJ Open	Hori H	BMJ Open	2016
27820794	0	0	0	Work relat	3	Musle ske	Muskel	Epidemiolo	A cross-se	Adult; Age	Coggon D,	Spine (Phi	Coggon D	Spine (Phi	2017
27885247	0	0	0	Work envir	4	engageme	Depressio	Effects of	The purpo	Adult; Cog	Imamura K	J Occup He	Imamura K	J Occup He	2017
27918891	0	0	0	Work and	1	informal c	介護者は不	The socioe	Caregiving	Informal c	Tokunaga	Soc Sci Me	Tokunaga	Soc Sci Me	2017
27931906	0	0	0	Mental He	1	occupation	character	Burnout in	High risk c	Burnout; C	Miyoshi R,	Asian J Ps	Miyoshi R	Asian J Psy	2016
27980314	0	0	0	Work and	3	Returntow	stroke	Comparisc	The condit	Adolescen	Saeki S, M	J UOEH. 20	Saeki S	J UOEH	2016
28045799	0	0	0	Career /ec	4	education	profession	Effects of	Stimulatin	Adult; Con	Sasaki N, S	J Occup Er	Sasaki N	J Occup Er	2017
28049047	0	0	0	Work and	3	WFC		Does work	It is well k	Fixed-effe	Oshio T, In	Soc Sci Me	Oshio T	Soc Sci Me	2017
28053538	0	0	0	Lifestyle r	1	LRD	CVD	Inverse rel	The object	BMI; arter	Nagayama	Vasc Healt	Nagayama	Vasc Healt	2016
29605759	0	0	0	Lifestyle r	1	others	checkup p	Time cost	Women of		Anezaki H,	Soc Sci Me	Anezaki H	Soc Sci Me	2018
29871634	0	0	0	Work envir	1	retention	turnover	Factors as	Backgroun		Suga R, Ts	BMC Wom	Suga R	BMC Wom	2018
30058596	0	0	0	Infertility,	1	pregnancy	Positive er	Positive Er	During pre		Nakamura	Tohoku J E	Nakamura	Tohoku J E	2018
30276496	0	0	0	Menstruat	1	cervical ca	screening	Factors re	Backgroun		Kaso M, T	Int J Clin C	Kaso M	Int J Clin C	2019
28123138	0	0	0	Mental He	3	lifestyle ar	alcohol an	Changes i	This study	Drinking h	Nakagawa	Ind Health	Nakagawa	Ind Health	2017
28132969	0	0	0	Mental He	1	occupation	sleep	Health-rel	Sleep distu	Adolescen	Kageyama	J Occup He	Kageyama	J Occup He	2017
28132971	0	0	0	Work relat	4	Musle skeletal disord		Effects of	To examin	Adult; Disa	Hagiwara	J Occup He	Hagiwara	J Occup He	2017
28163282	0	0	0	Mental He	1	occupation	biomarker	Potential c	The predic	Fatigue; O	Ebata C, T	J Occup He	Ebata C	J Occup He	2017
28235008	0	0	0	Work and	4	WFC	介護者は不	The impac	This study	Adult; Emo	Watanabe	PLoS One.	Watanabe	PLoS One	2017
28235820	0	0	0	Work envir	3	workplace		Workplace	This study	Cohort stu	Sakuraya A	J Epidemic	Sakuraya A	J Epidemic	2017
28255646	0	0	0	Work relat	2	occupation		MR imagir	To retrosp	Cholangio	Koyama K	Jpn J Radi	Koyama K	Jpn J Radi	2017
28267101	0	0	0	Mental He	1	stress mar		Improvem	This cross	Adult; Cro	Watanabe	J Occup Er	Watanabe	J Occup Er	2017
28302927	0	0	0	Harassme	1	mistreatm		Workplace	Although i	Aggressior	Tsuno K, K	J Occup He	Tsuno K	J Occup He	2017
28335426	0	0	0	Lifestyle r	3	physical a	workload	Elevated S	Iron is ess	athletes; i	Ishibashi A	Nutrients.	Ishibashi A	Nutrients	2017
28336104	0	0	0	Lifestyle r	1	LRD	metabolic	Serum 25-	Increasing	25(OH)D;	Akter S, Eg	Nutrition.	Akter S	Nutrition	2017
28357607	0	0	0	Work envir	1	productivit	presentee	Social sup	To elucida	Absentee	Saijo Y, Yo	Int Arch O	Saijo Y	Int Arch O	2017
28391107	0	0	0	Work envir	1	engagement		The relatic	Communic	Communic	Kunie K, K	Int J Nurs	Kunie K	Int J Nurs	2017
28403218	0	0	0	Mental He	1	occupation		Associatio	This study	Adult; Argi	Ogino K, It	PLoS One.	Ogino K	PLoS One	2017
28407025	0	0	0	Mental He	1	occupation		Relationsh	Insomnia	Adult; Fem	Deguchi Y	PLoS One.	Deguchi Y	PLoS One	2017
28486343	0	0	0	Work relat	2	substance	Fractional	Fractional	The object	Acetone; A	Suzuki RA	J Occup Er	Suzuki RA	J Occup Er	2017
28531194	0	0	0	Work relat	1	Musle skeletal disord		Prognostic	Although t	Adult; Cro	Yoshimoto	PLoS One.	Yoshimoto	PLoS One	2017
28534280	0	0	0	Mental He	3	occupation	sleep	A prospect	The role o	Chronic ki	Sasaki S, Y	Sleep Brea	Sasaki S	Sleep Brea	2018
28554985	0	0	0	Work relat	2	PCB, asbes	lead	Evaluation	Lead expo	Adolescen	Karakulak	Anatol J C	Karakulak	Anatol J C	2017
28582826	0	0	0	Lifestyle r	1	diet and n	diet	Associatio	Previous s	Adult; Cro	Kushida O	Asia Pac J	Kushida O	Asia Pac J	2017
28590152	0	0	0	Mental He	1	occupation	sleep	Workers' s	The purpo	autonomic	Toyoshima	Int J Occup	Toyoshima	Int J Occup	2018
28598932	0	0	0	Work envir	1	productivit		Diagnostic	This study	Adult; Area	Nagata T,	J Occup Er	Nagata T	J Occup Er	2017
28634729	0	0	0	Work and	0	workstyle		Analysis o	To assess	Women wo	Kawase K,	Surg Toda	Kawase K	Surg Toda	2018
28662058	0	0	0	Work envir	1	workplace		Psychome	Workplace	Adolescen	Eguchi H,	PLoS One.	Eguchi H	PLoS One	2017
28680003	0	0	0	Mental He	1	occupation	sleep	The relatic	The aim of	Hamilton c	Ikeda H, K	Ind Health	Ikeda H	Ind Health	2017
28685629	0	0	0	Work envir	1	productivit		Examining	Smoking is	Activity im	Suwa K, F	J Med Eco	Suwa K	J Med Eco	2017
28724951	0	0	0	Lifestyle r	3	LRD	CVD	N-termina	Few data	Adult; Age	Tanaka A,	Sci Rep. 20	Tanaka A	Sci Rep	2017
28744987	0	0	0	Career /ec	3	education	medical pe	Is modifie	To evaluat	assertiven	Yoshinaga	J Nurs Ma	Yoshinaga	J Nurs Ma	2018
28750431	0	0	0	Lifestyle r	1	LRD	CVD	Nitric Oxid	<b>Objec	Adult; Ank	Miyata S,	Exp Clin E	Miyata S	Exp Clin E	2017
28768509	0	0	0	Work relat	1	Musle ske	risk factor	Assessme	Most studi	Disabling	Kawaguch	BMC Musc	Kawaguch	BMC Musc	2017
28816734	0	0	0	Work envir	4	productivit	presentee	The Introd	The prese	Adult; Ant	Michishita	J Occup Er	Michishita	J Occup Er	2017
28824058	0	0	0	Lifestyle r	1	diet and n	diet	The SONG	Objective	Salt Check	Isaka Y, M	Intern Mec	Isaka Y	Intern Mec	2017
28830420	0	0	0	Lifestyle r	4	lifestyle	smoke	A randomi	Smoking is	Biomarker	Gale N, Mc	BMC Publi	Gale N	BMC Publi	2017
28840659	0	0	0	Harassme	1	violence a		Violence e	WHAT IS K	community	Fujimoto H	J Psychiatr	Fujimoto H	J Psychiatr	2017
28855438	0	0	0	Mental He	4	occupation	sleep	Verificatio	Short slee	Activity mo	Nakada Y,	Ind Health	Nakada Y	Ind Health	2018
28874315	0	0	0	Work relat	1	physical sy	allergy	Hand ecze	An increas	Food aller	Minami T,	Allergol Int	Minami T	Allergol Int	2018
28905851	0	0	0	Lifestyle r	1	LRD	CVD	Increased	It has bee	Blood Pres	Kimura G,	Hypertens	Kimura G	Hypertens	2017
28915887	0	0	0	Work and	1	WFC		Profession	In Japan, t	Basic Scie	Yamazaki	Hum Reso	Yamazaki	Hum Reso	2017
28921482	0	0	0	Work and	1	workstyle		Analysis o	To assess	Gender rol	Kawase K,	Surg Toda	Kawase K	Surg Toda	2018
28931789	0	0	0	Lifestyle r	2	LRD	CVD	Metabolic	We investi	Cardiovas	Hu H, Nak	Circ J. 201	Hu H	Circ J	2018
28954586	0	0	0	Work relat	1	shift	brain activ	Night duty	Overwork,	Residents;	Nishida M	Med Educ	Nishida M	Med Educ	2017
28963953	0	0	0	Work and	1	WFC		Work, fam	A high leve	Gender; Ja	Koura U, S	Public Hea	Koura U	Public Hea	2017
28967647	0	0	0	Work relat	1	Musle skeletal disord		Associatio	To elucida	cross-sect	Tsuboi Y, I	Int J Occup	Tsuboi Y	Int J Occup	2018
29036512	0	0	0	Work and	1	Cancer	Returntow	Job resign	Despite ac	healthcare	Takahashi	Jpn J Clin O	Takahashi	Jpn J Clin O	2018
29036820	0	0	0	Work and	3	Returntow	resignatior	Job Loss A	Early-onse	Claims dat	Sakata N,	J Alzheime	Sakata N	J Alzheime	2017
29081080	0	0	0	Work relat	1	shift	work	Pediatric r	Although t	pediatric r	Nomura O	Pediatr Int	Nomura O	Pediatr Int	2017
29093366	0	0	0	Work envir	3	productivit	absenteeis	A Japanese	On Decem	Cox propo	Tsutsumi A	J Occup He	Tsutsumi A	J Occup He	2018
29165115	0	0	0	Work and	1	WFC		Effects of	Accumulat	Academic	Chatani Y,	Environ He	Chatani Y	Environ He	2017

29165170	0	0	0	Work relat	1	comfortab	sick house	Evaluating	Psychosoc	Building-re	Azuma K, I	Environ He	Azuma K	Environ He	2017
29165171	0	0	0	Work and I	1	Returntow	depressior	Psychosoc	This study	Mental hea	Eguchi H, '	Environ He	Eguchi H	Environ He	2017
29269634	0	0	0	Mental He	1	occupatior		The Assoc	There is lit	autonomic	Enoki M, N	Tohoku J E	Enoki M	Tohoku J E	2017
29280773	0	0	0	Work envir	1	engageme		Work Enga	The aim of	Adult; Cros	Ishii K, Shi	J Occup Er	Ishii K	J Occup Er	2018
29300497	0	0	0	Work relat	1	shift		Associatio	Rotating sl	Rotating s	Yoshizaki '	Chronobio	Yoshizaki '	Chronobio	2018
29325537	0	0	0	Work relat	1	Musle skeletal disord		Factors as	We conduc	Absence; I	Watanabe	BMC Musc	Watanabe	BMC Musc	2018
29394196	0	0	0	Work envir	1	productivit	absenteeis	Total Heal	This study	Absenteeis	Nagata T,	J Occup Er	Nagata T	J Occup Er	2018
29407345	0	0	0	Work envir	1	retention	turnover	Factors rel	Keeping lo	Cross-sec	Eltaybani S	Int J Nurs S	Eltaybani S	Int J Nurs S	2018
29457499	0	0	0	Lifestyle re	0	lifestyle	alcohol	Factors Re	This study	Japan; alco	Ohida N, C	Asia Pac J	Ohida N	Asia Pac J	2018
29466974	0	0	0	Lifestyle re	1	oral health	oral	Temporary	Temporary	Employme	Sato Y, Ts	BMC Oral	Sato Y	BMC Oral	2018
29471260	0	0	0	Mental He	1	occupational stress		Stress unc	Appropriat	Attitude to	Nakamura	Psychiatry	Nakamura	Psychiatry	2018
29503387	0	0	0	Lifestyle re	1	others	checkup p	Factors Re	This study	Japanese I	Imamura H	J Epidemic	Imamura H	J Epidemic	2018
29506081	0	0	0	Mental He	1	occupatior	sleep	Associatio	Work-relat	Adult; Cros	Iwasaki S,	Occup Me	Iwasaki S	Occup Me	2018
29563368	0	0	0	Work envir	1	engagement		Associatio	We examir	Industrial p	Oshio T, Ir	J Occup He	Oshio T	J Occup He	2018
29593172	0	0	0	Work relat	1	shift	interleukin	Interleukin	Shift work	CVD; Inter	Amano H,	J Atherosc	Amano H	J Atherosc	2018
29618677	0	0	0	Work relat	4	VDT		Impact of I	To evaluat	Dry eye; L	Kawashim	J Occup He	Kawashim	J Occup He	2018
29627596	0	0	0	Lifestyle re	4	diet and nu	diet	Omega-3 f	The effica	Anxiety; B	Watanabe	J Psychiatr	Watanabe	J Psychiatr	2018
29657240	0	0	0	Lifestyle re	1	LRD	CVD	Associatio	This study	Cardiovasc	Ando E, Ka	Ind Health	Ando E	Ind Health	2018
29665820	0	0	0	Career /ec	1	satisfactio	QOL	Work impa	Osteoarthr	Absenteeis	Nakata K,	Health Qua	Nakata K	Health Qua	2018
29669966	0	0	0	Mental He	3	stress mar	program	Effect of tl	This retros	Mental hea	Imamura K	J Occup He	Imamura K	J Occup He	2018
29669967	0	0	0	Work relat	0	shift	platlet	The impac	Rotating sl	Cyclooxyge	Nakao T, Y	J Occup He	Nakao T	J Occup He	2018
29682824	0	0	0	Mental He	1	overtime and mental		The effect	Recent res	involuntary	Watanabe	J Nurs Mar	Watanabe	J Nurs Mar	2018
29731278	0	0	0	Work relat	1	Musle ske	Muskel 15	The hybrid	Work-relat	Hybrid ass	Miura K, K	J Clin Neur	Miura K	J Clin Neur	2018
29760353	0	0	0	Harassmer	1	violence ar		Direct and	The purpo	academic '	Takeuchi M	Tohoku J E	Takeuchi M	Tohoku J E	2018
29765987	0	0	0	Lifestyle re	3	LRD	glucose m	Insulin Sec	To charact	Blood Gluc	Aono D, O	J Diabetes	Aono D	J Diabetes	2018
29852386	0	0	0	Harassmer	1	stigma		An interne	Stigma ass	Healthcare	Fujii T, Ha	Asian J Psy	Fujii T	Asian J Psy	2018
29897506	0	0	0	Mental He	3	overtime and mental		Incidence	Mental dis	Adult; Age	Yamauchi	Occup Me	Yamauchi	Occup Me	2018
29921679	0	0	0	Mental He	1	occupatior		Cross-sect	Stress has	cognitive s	Tohmiya N	BMJ Open	Tohmiya N	BMJ Open	2018
29950467	0	0	0	Work envir	1	productivit	presenteei	Associatio	This study	chronic lov	Tsuji T, Ma	BMJ Open	Tsuji T	BMJ Open	2018
30019174	0	0	0	Mental He	1	occupatior		Gender dif	This study	Gender dif	Kachi Y, H	Int Arch Oc	Kachi Y	Int Arch Oc	2018
30064505	0	0	0	Menstruat	1	cervical ca	cervical ca	Factors as	The preval	Cervical ca	Kaneko N.	BMC Wom	Kaneko N	BMC Wom	2018
30089763	0	0	0	Work envir	1	workplace		Reliability	The study	Feedback	Momotani	Ind Health	Momotani	Ind Health	2019
30099352	0	0	0	Lifestyle re	1	LRD	metabolic	Associatio	Social jetla	Japanese;	Islam Z, Al	Sleep Med	Islam Z	Sleep Med	2018
30124499	0	0	0	Harassmer	1	bullying		Workplace	We sought	Adolescen	Tsuno K, K	J Occup Er	Tsuno K	J Occup Er	2018
30130990	0	0	0	Menstruat	4	menstruat	depressior	Effectivene	Hormonal	I10; I12; M	Song M, K	J Med Eco	Song M	J Med Eco	2018
30130991	0	0	0	Work relat	4	Musle skeletal disord		The effect	<b>Objec	Exercise; f	Oka H, No	Mod Rheu	Oka H	Mod Rheu	2019
30158360	0	0	0	Work and I	1	informal ca		Mental hea	This study	Caregivers	Eguchi H, '	J Occup He	Eguchi H	J Occup He	2018
30175718	0	0	0	Work relat	1	shift	diet, 2600	Dietary int	Shift work	Body mass	Nakamura	J Occup He	Nakamura	J Occup He	2018
30227266	0	0	0	Lifestyle re	3	LRD	CVD	Prediabete	We aimed	Cardiovasc	Hu H, Mizo	Atheroscle	Hu H	Atheroscle	2018
30231562	0	0	0	Mental He	1	lifestyle ar	sleep	Short-Terr	This study	health care	de Jonge J	Int J Enviro	de Jonge J	Int J Enviro	2018
30259880	0	0	0	Lifestyle re	3	LRD	metabolic	The assoc	To investig	Job Conter	Yamaguch	J Occup He	Yamaguch	J Occup He	2018
30289190	0	0	0	Work envir	1	productivit	presenteei	Associatio	Presentee	Adult; Age	Tsuboi Y, I	Eur J Pain.	Tsuboi Y	Eur J Pain	2019
30308617	0	0	0	Lifestyle re	2	lifestyle	smoke	Relationsh	Although s	Adult; Cas	Morita Y, (	J Occup Er	Morita Y	J Occup Er	2018
30334583	0	0	0	Work and I	1	Returntowork		Re-employ	Re-employ	employme	Fukuzawa	Aust Occu	Fukuzawa	Aust Occu	2018
30358090	0	0	0	Work envir	1	retention	turnover	Predicting	Nursing sh	nurses; org	Arslan Yür	Jpn J Nurs	Arslan Yür	Jpn J Nurs	2019
30373991	0	0	0	Mental He	1	occupatior	pain	The relatic	Pain is affe	Catastroph	Sakamoto	Work. 2018	Sakamoto	Work	2018
30395009	0	0	0	Career /ec	3	satisfactio		The Contri	The purpo	Adult; Dep	Tatsuse T,	J Occup Er	Tatsuse T	J Occup Er	2019
30430856	0	0	0	Harassmer	1	mistreatm		Perceived	NA	Japan; age	Harada K,	Int J Aging	Harada K	Int J Aging	2019
30473549	0	0	0	Work envir	1	retention	turnover	Determina	In order to	favorable v	Satoh M, V	Tohoku J E	Satoh M	Tohoku J E	2018
30477210	0	0	0	Work envir	4	productivit	oral health	Does Instr	Oral diseas	behavioral	Toyama N	Int J Enviro	Toyama N	Int J Enviro	2018
30485927	0	0	0	Lifestyle re	1	diet and nu	diet	Soy produc	The aim of	Adult; Asth	Nakamoto	Asia Pac J	Nakamoto	Asia Pac J	2018
30501428	0	0	0	Lifestyle re	1	others	Locomo	Locomotiv	<b>Backg	Epidemiolo	Nishimura	Mod Rheu	Nishimura	Mod Rheu	2020
30531094	0	0	0	Lifestyle re	1	others	checkup p	Associatio	Taking act	Comprehe	Seko R, Ka	Ind Health	Seko R	Ind Health	2019
30531106	0	0	0	Work envir	1	productivit	absenteeis	Relationsh	This study	Health ins	Muramats	Ind Health	Muramats	Ind Health	2019
30532174	0	0	0	Mental He	4	stress management		Developme	In the worl	Adult; Dep	Kubo H, U	PLoS One.	Kubo H	PLoS One	2018
30544456	0	0	0	Work envir	1	retention	turnover	Factors as	In industria	Adult; Age	Tayama J,	Medicine (	Tayama J	Medicine (	2018
30550709	0	0	0	Work relat	1	shift	blood pres	Effects of	Backgroun	Adult; Bloo	Kitada R, I	Osaka City	Kitada R	Osaka City	2016
30556370	0	0	0	Work and I	1	WFC		Associatio	The preser	care burde	Sakka M, (	Geriatr Ge	Sakka M	Geriatr Ge	2019
30580264	0	0	0	Work envir	3	workplace		Associatio	Workplace	mental hea	Eguchi H, '	BMJ Open	Eguchi H	BMJ Open	2018
30586369	0	0	0	Work envir	3	engagement		Is too muc	Most studi	Creativity;	Shimazu A	PLoS One.	Shimazu A	PLoS One	2018
30616127	0	0	0	Lifestyle re	1	lifestyle	obesity	Demograp	This study	25-hydroxy	Nanri A, K	Psychiatry	Nanri A	Psychiatry	2019
30755206	0	0	0	Career /ec	5	career	medical pe	Profession	During pro	Female ph	Matsui T, S	BMC Med	Matsui T	BMC Med	2019
30762243	0	0	0	Work relat	2	Musle skeletal disord		Factory an	To explore	Lumbar sp	Ishimoto Y	Am J Ind M	Ishimoto Y	Am J Ind M	2019
30793826	0	0	0	Work envir	1	engageme		Associatio	The aims o	work enga	Okazaki E,	J Occup He	Okazaki E	J Occup He	2019
30804025	0	0	0	Lifestyle re	1	physical ac	sedentary	Patterns o	To examin	Asia; desc	Kurita S, S	BMJ Open	Kurita S	BMJ Open	2019
30828034	0	0	0	Lifestyle re	1	lifestyle	others	Utility of a	The Japan	Japan; hea	Fukasawa	J Epidemic	Fukasawa	J Epidemic	2020
30845875	0	0	0	Lifestyle re	2	LRD	CVD	Occupatio	Backgroun	Japan; car	Zaitsu M, I	J Am Hear	Zaitsu M	J Am Hear	2019
30867395	0	0	0	Work relat	3	substance e	toner	Health Effe	The main o	biological	Kitamura H	J UOEH. 20	Kitamura H	J UOEH	2019
30879967	0	0	0	Lifestyle re	1	lifestyle	others	Hours of w	This article	Health beh	Okamoto S	Ann Epide	Okamoto S	Ann Epider	2019
30895696	0	0	0	Work relat	3	occupatior		Possibility	We establi	ITO; cohor	Nakano M	J Occup He	Nakano M	J Occup He	2019
30918213	0	0	0	Work relat	1	safety and v		Analysis o	Needle-sti	clean-up p	Matsumot	J Oral Sci.	Matsumot	J Oral Sci	2019
30927579	0	0	0	Mental He	4	stress management		Brief mindf	The effica	Anxiety; B	Watanabe	J Affect Di	Watanabe	J Affect Di	2019
30953383	0	0	0	Work envir	1	productivit	presenteei	Associatio	Chronic lo	acute low	Yokota J, F	J Occup He	Yokota J	J Occup He	2019
30954364	0	0	0	Lifestyle re	3	lifestyle	obesity	Obesity an	The existir	Body mass	Hu H, Tom	Clin Nutr. 2	Hu H	Clin Nutr	2020
31004382	0	0	0	Work relat	1	Musle skeletal disord		Priority ap	The incide	care equip	Iwakiri K, T	J Occup He	Iwakiri K	J Occup He	2019
31014131	0	0	0	Work relat	3	shift		Effect of c	We investi	Morningne	Tomizawa	Chronobio	Tomizawa	Chronobio	2019
31023765	0	0	0	Lifestyle re	1	LRD	HTN	Associatio	Preventior	cross-sect	Umesawa	BMJ Open	Umesawa	BMJ Open	2019
31041854	0	0	0	Mental He	3	overtime and mental		The effect	The purpo	Japan; dep	Hino A, Inc	J Occup He	Hino A	J Occup He	2019
31063040	0	0	0	Lifestyle re	1	LRD	metabolic	Metabolic	This study	Japanese v	Kakinuma	Arch Envir	Kakinuma	Arch Enviro	2020

31063680	0	0	0	Career /ec	1	career	medical pe	Clinical pra	To explore	clinical lad	Hama T, T	J Clin Nurs	Hama T	J Clin Nurs	2019
31105090	0	0	0	Mental He	1	stress mar	seeking fo	Help-Seek	The preser	Japan; hel	Yamauchi	J Epidemic	Yamauchi	J Epidemic	2020
31105117	0	0	0	Lifestyle re	1	oral health	oral	Effort-rew	Oral disea	Dental hea	Sato Y, Ts	Ind Health	Sato Y	Ind Health	2020
31129552	0	0	0	Work relat	1	shift	restless le	The impac	Decreases	Midwifery;	Uekata S,	Int J Nurs	Uekata S	Int J Nurs	2019
31131521	0	0	0	Work envir	1	workplace	work_env	Outcomes	The purpo	facilitators	Yoshikawa	J Occup He	Yoshikawa	J Occup He	2019
31155542	0	0	0	Mental He	3	lifestyle and mental h		Prospectiv	While a gr	Japan; dep	Fukunaga	J Epidemic	Fukunaga	J Epidemic	2020
31167500	0	0	0	Lifestyle re	1	physical ac		Bout Leng	We aimed	Japanese;	Machida M	Int J Enviro	Machida M	Int J Enviro	2019
31207197	0	0	0	Lifestyle re	1	lifestyle	smoke	Influence (	Tobacco s	health ine	Fujita T, B	Popul Hea	Fujita T	Popul Hea	2020
31234390	0	0	0	Work envir	1	retention	turnover	Gender Di	Women in	burnout; g	Minamizor	Int J Enviro	Minamizor	Int J Enviro	2019
31276516	0	0	0	Lifestyle re	1	LRD	CVD	How do ca	To evaluat	Adult; Age	Fu R, Nogu	PLoS One.	Fu R	PLoS One	2019
31278448	0	0	0	Lifestyle re	1	lifestyle	smoke	Smoking is	Pain symp	Disability s	Yamada K	J Anesth. 2	Yamada K	J Anesth	2019
31280581	0	0	0	Lifestyle re	0	lifestyle	smoke	Prevalence	There is a	Japanese r	Jike M, Na	Asia Pac J	Jike M	Asia Pac J	2019
31283785	0	0	0	Infertility,	1	pregnancy	Strees on	The relatio	Prenatal p	Abruptio P	Kawanishi	PLoS One.	Kawanishi	PLoS One	2019
31288766	0	0	0	Work envir	1	workplace		Relationsh	A growing	Japan; Res	Hori D, Ta	BMC Publi	Hori D	BMC Publi	2019
31301551	0	0	0	Career /ec	0	self-effica	litearcy	Preference	To describ	Health info	Adachi T, I	Public Hea	Adachi T	Public Hea	2019
31309684	0	0	0	Menstruat	1	Returntow	両立支援女	Developme	This study	cancer tre	Nishikido I	J Occup He	Nishikido I	J Occup He	2019
31312010	0	0	0	Infertility,	3	pregnancy	Alcohol	Alcohol Co	There have	Abruptio P	Ohira S, M	Sci Rep. 20	Ohira S	Sci Rep	2019
31348424	0	0	0	Work envir	1	productivit	absenteeis	Potential V	The curren	Absenteeis	Chimed-O	J Occup En	Chimed-O	J Occup En	2019
31366851	0	0	0	Career /ec	1	satisfactio	well-being	Measuring	Although t	Eudemonia	Watanabe	Ind Health	Watanabe	Ind Health	2020
31391029	0	0	0	Mental He	4	stress mar	internet	Effects of	Depressio	Cognitive I	Kuribayash	BMC Psyc	Kuribayash	BMC Psyc	2019
31407499	0	0	0	Lifestyle re	2	others	cancer	Risk of car	Little is kn	cancer; oc	Kaneko R,	Cancer Me	Kaneko R	Cancer Me	2019
31437517	0	0	0	Work and	1	workstyle		A Questior	To analyze	Balancing	Maehara T	World Neu	Maehara T	World Neu	2020
31450754	0	0	0	Lifestyle re	1	physical ac	sedentary	Assessing	Various ac	accelerom	Yano S, Ko	Int J Enviro	Yano S	Int J Enviro	2019
31462618	0	0	0	Lifestyle re	1	LRD	metabolic	Relationsh	Obesity is	Atheroscle	Sugiura T,	J Atherosc	Sugiura T	J Atherosc	2020
31493339	0	0	0	Career /ec	1	education	profession	The effect	The aim of	manageme	Adachi H,	J Occup He	Adachi H	J Occup He	2020
31510922	0	0	0	Work and	1	WFC		Do multipl	Like most	Coping; Fe	Okada N, Y	Environ He	Okada N	Environ He	2019
31511285	0	0	0	Work and	3	Returntowork		Risk factor	We aimed	Major dep	Hori H, Ka	BMJ Open	Hori H	BMJ Open	2019
31526613	0	0	0	Mental He	3	lifestyle ar		Diet qualit	It is not cle	Depressive	Nanri A, N	Clin Nutr. 2	Nanri A	Clin Nutr	2020
31546684	0	0	0	Lifestyle re	1	oral health	oral	Influence (	The aim of	Japanese v	Islam MM,	Int J Enviro	Islam MM	Int J Enviro	2019
31613961	0	0	0	Lifestyle re	1	physical ac	sedentary	Bidirectior	Affective e	Arousal; E	Kim J, Con	Ann Behav	Kim J	Ann Behav	2020
31651604	0	0	0	Work envir	1	productivity		Validity an	We aimed	Adult; Dep	Tokutsu K	J Occup En	Tokutsu K	J Occup En	2019
31662983	0	0	0	Harassmer	1	stigma		Relationsh	Stigma rel	Burnout, P	Mitake T, I	Biomed Re	Mitake T	Biomed Re	2019
31667913	0	0	0	Infertility,	1	pregnancy	Working	Effects of	The work p	complicati	Suzumori I	Birth. 2020	Suzumori I	Birth	2020
31685757	0	0	0	Career /ec	1	satisfactio		Objective a	This study	Cardiovasc	Ochiai Y, T	Ind Health	Ochiai Y	Ind Health	2020
31691512	0	0	0	Career /ec	1	satisfactio	medical pe	A predictiv	The purpo	adaptatior	Kim JM, H	Jpn J Nurs	Kim JM	Jpn J Nurs	2020
31703665	0	0	0	Lifestyle re	1	physical ac	sedentary	Social-eco	Although t	Determina	Kurita S, S	BMC Publi	Kurita S	BMC Publi	2019
31721037	0	0	0	Menstruat	1	Returntow	両立支援女	Predictors	In Japan, c	Cancer site	Endo M, M	J Cancer S	Endo M	J Cancer S	2020
31749184	0	0	0	Work envir	1	organizational climate		The effect	Identifying	burnout; e	Takemura	J Nurs Mar	Takemura	J Nurs Mar	2020
31775617	0	0	0	Mental He	1	occupatior		Associatio	Flourishing	Flourishing	Hori D, Oi	Environ He	Hori D	Environ He	2019
31779617	0	0	0	Lifestyle re	1	physical ac		Associatio	High preva	Fear-avoic	Fujii T, Ok	BMC Musc	Fujii T	BMC Musc	2019
31815714	0	0	0	Harassmer	1	stigma		The assoc	Research I	Employme	Ottewell N	Work. 2019	Ottewell N	Work	2019
31815723	0	0	0	Mental He	1	occupatior		Structural	Occupatio	Structural	Teraoka M	Work. 2019	Teraoka M	Work	2019
31826384	0	0	0	Lifestyle re	1	diet and nu	diet	Associatio	The aim of	Adult; Asth	Nakamoto	Asia Pac J	Nakamoto	Asia Pac J	2019
31851058	0	0	0	Work envir	1	productivit	presenteei	The Impac	The aim of	Absenteeis	Zaitsu T, S	J Occup En	Zaitsu T	J Occup En	2020
31868763	0	0	0	Work envir	1	engageme		Is Higher V	We conduc	Adult; Cros	Amano H,	J Occup En	Amano H	J Occup En	2020
31878917	0	0	0	Work relat	2	Musle skeletal disord		Is radiogra	To explore	Agricultura	Ishimoto Y	BMC Musc	Ishimoto Y	BMC Musc	2019
31954287	0	0	0	Mental He	1	occupatior	sleep	A composi	Individual	Depressive	Furihata R	Compr Psy	Furihata R	Compr Psy	2020
31970914	0	0	0	Work envir	3	productivit	absenteeis	BMI and M	In contrast	Absenteeis	Endo M, Ir	Obesity (S	Endo M	Obesity (S	2020
32009152	0	0	0	Work envir	1	productivit	presenteei	Two defini	Two major	Impaired v	Ishimaru T	Occup Me	Ishimaru T	Occup Me	2020
32019535	0	0	0	Work envir	3	retention	turnover	Occupatio	Although s	Administra	Kachi Y, Ir	BMC Publi	Kachi Y	BMC Publi	2020
32077640	0	0	0	Work envir	3	productivit	absenteeis	Cardiovasc	We aimed	Japanese v	Hirokawa I	Brain Beha	Hirokawa I	Brain Beha	2020
32078676	0	0	0	Mental He	4	stress management		An individu	Mental hea	Early inter	Soeda S.	Occup Me	Soeda S	Occup Me	2020
32149939	0	0	0	Work envir	1	retention	turnover	The Assoc	The aim of	Adult; Cros	Iida M, Wa	J Occup En	Iida M	J Occup En	2020
32173661	0	0	0	Mental He	1	scale dev		Construct	The aim of	Absenteeis	Kawakami	Ind Health	Kawakami	Ind Health	2020
32220374	0	0	0	Mental He	1	lifestyle ar		Prospectiv	Oxidative s	Depressio	Miki T, Egu	Clin Nutr E	Miki T	Clin Nutr E	2020
32228490	0	0	0	Work and	0	Returntow	interview	Impact of i	Cancer sur	Cancer sur	Watanabe	BMC Canc	Watanabe	BMC Canc	2020
32281543	0	0	0	Harassmer	1	bullying		School Ijim	Early-life a	Ijime (bully	Ikeda T, H	Tohoku J E	Ikeda T	Tohoku J E	2020
32281586	0	0	0	Harassmer	1	mistreatm		Work-life c	To clarify (	academic	Ono Y, Got	Fukushima	Ono Y	Fukushima	2020
32283628	0	0	0	Mental He	1	occupatior		Gender Di	Dairy farm	Japan; dai	Sato M, Ka	Int J Enviro	Sato M	Int J Enviro	2020
32316142	0	0	0	Harassmer	1	violence ar		Workplace	Workplace	burnout; m	Kobayashi	Int J Enviro	Kobayashi	Int J Enviro	2020
32321951	0	0	0	Work envir	1	engageme		Brain conc	Over the p	Adult; Brai	Kokubun K	Sci Rep. 20	Kokubun K	Sci Rep	2020
32328623	0	0	0	Work and	1	Cancer	cancer	Impact of g	To identify	cancer; job	Tsuchiya M	Jpn J Clin (	Tsuchiya M	Jpn J Clin (	2020
32408638	0	0	0	Lifestyle re	1	LRD	CVD	Occupatio	This study	cardiovasc	Liu ML, Ch	Int J Enviro	Liu ML	Int J Enviro	2020
32421704	0	0	0	Career /ec	1	satisfactio	medical pe	Factors as	As the Jap	Adult; Age	Kitajima M	PLoS One.	Kitajima M	PLoS One	2020
32421808	0	0	0	Work envir	1	workplace		Quality of	Employees	European	Kizuki M, F	Occup Me	Kizuki M	Occup Me	2020
32434997	0	0	0	Work and	3	WFC		The health	High level	Job satisfa	Koura U, S	Ind Health	Koura U	Ind Health	2020
32443446	0	0	0	Work envir	1	productivit		The Effect	The constr	Japan; age	Hashiguch	Int J Enviro	Hashiguch	Int J Enviro	2020
32486232	0	0	0	Lifestyle re	1	LRD	metabolic	The Effect	The effect	leisure act	So R, Mats	Int J Enviro	So R	Int J Enviro	2020
32515911	0	0	0	Mental He	1	lifestyle ar		The assoc	To examin	cross-sect	Fukai K, K	J Occup He	Fukai K	J Occup He	2020
32545918	0	0	0	Lifestyle re	1	diet and nu	diet	Fish Cooki	The aim of	Japanese;	Nanri A, Ta	Nutrients.	Nanri A	Nutrients	2020
32575014	0	0	0	Work and	1	Returntow	resignatior	Seizures, a	Few studie	Employme	Nishida T,	Epilepsy B	Nishida T	Epilepsy B	2020
32578270	0	0	0	Infertility,	1	pregnancy	depressior	Do postpa	We investi	EPDS; bor	Minamida	Infant Mer	Minamida	Infant Mer	2020
32590934	0	0	0	Work relat	1	safety and v		Factors rel	The Japan	Profession	Tei-Tomin	Environ He	Tei-Tomin	Environ He	2020
32595178	0	0	0	Work envir	3	productivit	absenteeis	Job stress	This study	depressive	Hirokawa I	Fukushima	Hirokawa I	Fukushima	2020
32595181	0	0	0	Lifestyle re	1	lifestyle	smoke	Widening S	Japan is or	Japan; epi	Tanaka H,	J Epidemic	Tanaka H	J Epidemic	2021
32612009	0	0	0	Mental He	3	occupatior		Effect of w	While a nu	Depressio	Nishimura	Ind Health	Nishimura	Ind Health	2020
32660575	0	0	0	Mental He	1	stress mar		Effect of s	Burnout ar	Burnout; M	Kijima S, T	BMC Med	Kijima S	BMC Med	2020
32664291	0	0	0	Lifestyle re	1	diet and nu	diet	Cross-Sec	We examir	Japanese;	Nanri H, K	Nutrients.	Nanri H	Nutrients	2020
32664915	0	0	0	Menstruat	3	breast ca	breast can	Associatio	Breast car	Breast car	Sari GN, E	BMC Publi	Sari GN	BMC Publi	2020

32677152	0	0	0	Career /ec	1	satisfactio	medical pe	Quality of	This study	job charac	Komagata	Nurs Healt	Komagata	Nurs Healt	2020
32698340	0	0	0	Mental He	1	stress mar		Experience	Physician	burnout; c	Toyoshima	Int J Envir	Toyoshima	Int J Envir	2020
32713895	0	0	0	Mental He	1	occupation		Psycholog	The under	Childcare	Yaginuma-	Ind Health	Yaginuma-	Ind Health	2020
32713929	0	0	0	Work envir	1	productivit	absenteeis	Loss of W	While muc	death; occ	Inoue Y, N	J Epidemic	Inoue Y	J Epidemic	2021
32715582	0	0	0	Career /ec	1	satisfactio	well-being	Validation	We set ou	health-rela	Chimed-O	J Occup He	Chimed-O	J Occup He	2020
32730035	0	0	0	Lifestyle re	1	lifestyle	Wt	Lifestyle C	To examin	Adult; Age	Suka M, Y	J Occup Er	Suka M	J Occup Er	2020
32747350	0	0	0	Work envir	3	workplace		Workplace	We examir	epidemiolo	Inoue A, T	BMJ Open	Inoue A	BMJ Open	2020
32787717	0	0	0	Work and	1	informal ca	介護者は不	Parental C	In this pap	Japan; em	Kikuzawa	Res Aging.	Kikuzawa	Res Aging	2021
32787774	0	0	0	Work envir	1	workplace		Developme	Although r	Nurses; Ps	Norikoshi	Environ He	Norikoshi	Environ He	2020
32846936	0	0	0	Infertility,	1	pregnancy	smoke	Factors As	This study	Japan; nur	Li M, Okar	Int J Envir	Li M	Int J Envir	2020
32852701	0	0	0	Lifestyle re	1	others	CKD	Occupatio	Although s	Occupatio	Fujii Y, Ya	J Nephrol.	Fujii Y	J Nephrol	2021
32853920	0	0	0	Mental He	1	overtime a		Relationsh	The prese	Commutin	Hori D, Sa	Sleep Med	Hori D	Sleep Med	2020
32883304	0	0	0	Lifestyle re	1	lifestyle	alcohol	Alcohol dri	Both alcoh	Alcohol; S	Lin H, Cha	Subst Abu	Lin H	Subst Abu	2020
32887598	0	0	0	Mental He	1	occupation	SOC	Sense of c	Internation	General he	Kuwato M	BMC Publi	Kuwato M	BMC Publi	2020
32938548	0	0	0	Mental He	1	occupation		Reversal p	Despite ex	Career tra	Takaku R.	Health Pol	Takaku R	Health Pol	2020
32938849	0	0	0	Lifestyle re	1	others	snoring	Relationsh	Objective	dyslipidem	Sekizuka H	Intern Med	Sekizuka H	Intern Med	2020
33010680	0	0	0	Mental He	2	lifestyle ar		Diabetes,	To examin	Case&#x2	Fukunaga	J Psychoso	Fukunaga	J Psychoso	2020
33030773	0	0	0	Work envir	1	workplace		The assoc	To identify	communic	Kida R, To	J Nurs Ma	Kida R	J Nurs Ma	2021
33035239	0	0	0	Mental He	1	occupation		Impact of	The assoc	Adult; Cros	Nagasu M	PLoS One.	Nagasu M	PLoS One	2020
33047825	0	0	0	Work envir	1	productivit		Impact of	The impac	Constipati	Tomita T,	J Gastroen	Tomita T	J Gastroen	2021
33131153	0	0	0	Work envir	1	workplace		Workplace	Despite th	Japan; bur	Murayama	J Occup He	Murayama	J Occup He	2020
33148898	0	0	0	Lifestyle re	1	diet and nu	diet	Dietary div	The aim of	Japanese	Nakamoto	J Med Inve	Nakamoto	J Med Inve	2020
33187281	0	0	0	Lifestyle re	1	diet and nu	Nutrition	Omega-3	<i>Backgr	a sense of	Tsuboi H,	Nutrients.	Tsuboi H	Nutrients	2020
33211393	0	0	0	Work relat	1	shift		Frequency	In Japan, t	irregular m	Mayama M	J Occup He	Mayama M	J Occup He	2020
33229857	0	0	0	Work relat	1	Musle skeletal disord		Relationsh	Physical fu	Low back	Ishikawa D	Ind Health	Ishikawa D	Ind Health	2021
33268468	0	0	0	Work and	1	Returntow	resignation	Newly diag	Since prev	epidemiolo	Takada M,	Occup Env	Takada M	Occup Env	2021
33283972	0	0	0	Lifestyle re	1	LRD	HTN	Self-meas	The effect	home bloo	Tomitani N	J Clin Hype	Tomitani N	J Clin Hype	2021
33291636	0	0	0	Work and	1	workstyle		A Nationw	Sustainabl	gender; hc	Kodama T	Int J Envir	Kodama T	Int J Envir	2020
33301520	0	0	0	Work envir	1	productivit		Associatio	This cross	Adult; Cros	Okawara M	PLoS One.	Okawara M	PLoS One	2020
33413266	0	0	0	Lifestyle re	1	oral health	oral	Associatio	This study	Oral health	Harada Y,	BMC Publi	Harada Y	BMC Publi	2021
33441683	0	0	0	Work relat	1	comfortab	heat	Heat-relat	Heat-relat	Constructi	Kakamu T	Sci Rep. 2	Kakamu T	Sci Rep	2021
33446165	0	0	0	Menstruat	1	Returntow	両立支援女	Predictors	In Japan, 5	Breast car	Mitsui K, E	BMC Publi	Mitsui K	BMC Publi	2021
33477353	0	0	0	Career /ec	1	satisfactio	medical pe	Job Attract	Job attract	dental hyg	Ohara Y, N	Int J Envir	Ohara Y	Int J Envir	2021
33512763	0	0	0	Mental He	1	occupation	JCS	Developme	Team job	hospital nu	Iida M, Wa	Res Nurs H	Iida M	Res Nurs H	2021
33534962	0	0	0	Work relat	1	Musle ske	office worl	Associatio	Our study	epidemiolo	Nakatsuka	J Occup He	Nakatsuka	J Occup He	2021
33605494	0	0	0	Lifestyle re	1	LRD	glucose m	Type and t	Suppressio	aerobic ex	Yoko N, Hi	J Occup He	Yoko N	J Occup He	2021
33683773	0	0	0	Work envir	1	productivit	presenteei	Associatio	To addres	presenteei	Ishimaru T	J Occup He	Ishimaru T	J Occup He	2021
33727738	0	0	0	Mental He	1	occupation		Difference	The Japan	female ma	Shiraki N,	Nagoya J M	Shiraki N	Nagoya J M	2021
33809121	0	0	0	Work envir	1	productivit	sleep	Sleep Deb	Although s	depression	Okajima I,	Int J Envir	Okajima I	Int J Envir	2021
33930791	0	0	0	Work and	1	WFC		Work-fami	The assoc	Insomnia s	Sato S, Liu	Sleep Med	Sato S	Sleep Med	2021
33932320	0	0	0	Mental He	4	stress management		Effect of ir	This study	Japanese;	Tayama J,	J Occup He	Tayama J	J Occup He	2021
34052861	0	0	0	Mental He	1	occupation	QUALY	Associatio	To investig	Demand&	Hidaka Y,	Int Arch O	Hidaka Y	Int Arch O	2021
34112832	0	0	0	Lifestyle re	3	physical ac		Leisure-tir	Physical a	Adult; Age	Yamamoto	Sci Rep. 2	Yamamoto	Sci Rep	2021
34312959	0	0	0	Career /ec	1	satisfactio	medical pe	Factors of	To examin	dementia;	Tei-Tomin	Geriatr Ge	Tei-Tomin	Geriatr Ge	2021
34444937	0	0	0	Lifestyle re	1	diet and nu	diet	Associatio	Breakfast	chrono-nu	Tahara Y,	Nutrients.	Tahara Y	Nutrients	2021
34460148	0	0	0	Work envir	1	productivit		Relationsh	The aim of	behavior;	Sakai K, N	J Occup He	Sakai K	J Occup He	2021
34477148	0	0	0	Work envir	1	retention	turnover	Factors as	The turnov	Adult; Chi-	Matsuo M	Medicine (	Matsuo M	Medicine (	2021
34582661	0	0	0	Menstruat	5	breast ca	life	What Japa	Living with	breast can	Yamauchi	Asian Pac	Yamauchi	Asian Pac	2021
34587662	0	0	0	Work envir	4	productivit	presenteei	Effect of p	Decreased	exercise; c	Imai R, Ko	J Occup He	Imai R	J Occup He	2021
34611942	0	0	0	Work relat	4	shift	diet, 2600	Towards ta	Blood gluc	Theoretica	Gibson R,	Diabet Me	Gibson R	Diabet Me	2022
34613962	0	0	0	Career /ec	1	self-effica	medical pe	Associatio	As populat	Altruism; A	Taguchi A,	PLoS One.	Taguchi A	PLoS One	2021
34679125	0	0	0	Work relat	4	shift		Effects of	Night shift	Adult; Cros	Oriyama S	PLoS One.	Oriyama S	PLoS One	2021
34687118	0	0	0	Mental He	1	occupation		A cross-se	This study	coworker s	Yaginuma-	J Occup He	Yaginuma-	J Occup He	2021
34907236	0	0	0	Lifestyle re	2	LRD	CVD	A case cor	We aimed	Aged; Card	Fukai K, F	Sci Rep. 2	Fukai K	Sci Rep	2021
34957639	0	0	0	Mental He	1	occupation		Role ambig	We aim to	Kessler 68	Oshio T, Ir	J Occup He	Oshio T	J Occup He	2021
34996917	0	0	0	Lifestyle re	1	physical ac	sedentary	Workplace	Workplace	Adult; Buil	Lin CY, Ko	Sci Rep. 2	Lin CY	Sci Rep	2022
35011043	0	0	0	Mental He	1	lifestyle and mental h		Green Tea	Although s	Japanese;	Nanri A, Eg	Nutrients.	Nanri A	Nutrients	2021
35255088	0	0	0	Career /ec	1	career	medical pe	PERSonali	The World	Adult; Bur	Matsuishi	PLoS One.	Matsuishi	PLoS One	2022
35364406	0	0	0	Work envir	1	productivit		On workda	Misalignm	Chronotyp	Shimura A	Sleep Med	Shimura A	Sleep Med	2022
35457291	0	0	0	Mental He	4	stress mar	email	How Does	This study	anxiety; cc	Ubara A, T	Int J Envir	Ubara A	Int J Envir	2022
35476638	0	0	0	Infertility,	1	pregnancy	Trends in	Salaries, d	While ferti	Aged; Chil	Ghaznavi (	PLoS One.	Ghaznavi (	PLoS One	2022
35727869	0	0	0	Work and	1	workstyle		Factors as	Given the	burnout; p	Ishikawa M	Pediatr Int	Ishikawa M	Pediatr Int	2022
35797138	0	0	0	Work envir	3	productivit	presenteei	Associatio	Presentee	Japanese g	Goto E, Ish	J Occup He	Goto E	J Occup He	2022
35861838	0	0	0	Lifestyle re	3	LRD	CVD	Associatio	Backgroun	cardiovasc	Hamaya R	J Am Hear	Hamaya R	J Am Hear	2022
