# Supplementary material for: The quantity and quality of scientific evidence about the health of working women in occupational health of Japan: A scoping review
Source: J Occup Health. 2023 Oct 16;65(1):e12427. doi: 10.1002/1348-9585.12427 (PMC10579630; doi:10.1002/1348-9585.12427)
Supplement: Supplementary file 2 — File S2. [file JOH2-65-e12427-s001.pdf]

id	n worker c	work relev	ific partici	ut of focu	領域	究デザイ	分類	memo	タイトル	抄録	著者名（筆	収載誌名（及び、ISSN、巻	type	キーワード	キーワード	M	doi	citationInfo
1.98E+09	0	0	0	0	egnancy ar	0	safety, wc	perinatal	自営業に從	出産後1～	出口 佐代	保健婦雑誌(0047-1844)38	原著論文	健康; 妊産		ヒト; 女	10.11477/	保健婦雑誌(0047-1844)38巻8号 Page714-719(1982.08)
1.98E+09	0	0	0	0	ed disease,	0	oral healtl	oral	歯周疾患に	日常生活環	渡辺 嘉一(	日本歯周病学会会誌(0385-	原著論文	歯周疾患(		ヒト; 歯学	10.2329/p	日本歯周病学会会誌(0385-0110)24巻3号 Page446-455(1982.09)
1.98E+09	0	0	0	0	related dis	0	Musle ske	others	各種農作業	女子は機械	二塚 信(熊	日本農村医学会雑誌(0468-	原著論文	女性; 農業		ヒト; 女	10.2185/jj	日本農村医学会雑誌(0468-2513)32巻2号 Page112-126(1983.07)
1.98E+09	0	0	0	0	related dis	0	Musle ske	others	比較的近代	営農形態に	渡部 真也(	日本農村医学会雑誌(0468-	原著論文	女性; 微候		ヒト; 女	10.2185/jj	日本農村医学会雑誌(0468-2513)32巻2号 Page101-111(1983.07)
1.98E+09	0	0	0	0	egnancy ar	0	pregnancy	perinatal	勤労婦人と		松本 八千	母性衛生(0388-1512)25巻	原著論文	予防医学;		妊娠; 女; 看		母性衛生(0388-1512)25巻1号 Page121-124(1984.03)
1.99E+09	0	0	0	0	rk and bala	0	workstyle	workstyle	現代女性の		天野 正子(	看護展望(0385-549X)9巻8	原著論文	看護師		看護		看護展望(0385-549X)9巻8号 Page674-679(1984.08)
1.99E+09	0	0	0	0	egnancy ar	0	work, nutr	perinatal	勤労妊婦の		塚田 一郎(	ペリネイタルケア(0910-87	原著論文	妊産婦(運		ヒト; 妊娠		ペリネイタルケア(0910-8718)4巻9号 Page22-26(1985.08)
1.99E+09	0	0	0	0	ed disease,	0	lifestyle	others	北海道内漁	砂原町では	小山 昌正(	日本農村医学会雑誌(0468-	原著論文	保健行動	漁民(調査	ヒト	10.2185/jj	日本農村医学会雑誌(0468-2513)34巻1号 Page1-11(1985.05)
1.99E+09	0	0	0	0	ed disease,	0	lifestyle	others	広島湾にお	「カキ」養	高科 成良(	日本農村医学会雑誌(0468-	原著論文	貝(食用);	漁民	ヒト	10.2185/jj	日本農村医学会雑誌(0468-2513)34巻1号 Page21-33(1985.05)
1.99E+09	0	0	0	0	egnancy ar	0	work, nutr	work, nutr	妊婦の就労		藤本 征一	ペリネイタルケア(0910-87	原著論文	胎児(組織		ヒト; 妊娠		ペリネイタルケア(0910-8718)4巻8号 Page137-144(1985.07)
2.01E+09	0	0	0	0	egnancy ar	0	mental he	mental he	心理・背景	妊娠初期、	冨田 康之(	生物工学会誌(0919-3758)8	原著論文	質問紙法;		ヒト; 妊娠		生物工学会誌(0919-3758)86巻11号 Page519-523(2008.11)
1.99E+09	0	0	0	0	related dis	1	Musle ske	MusleSke	高原野菜栽		松島 松翠(	日本農村医学会雑誌(0468-	原著論文	女性(衛生			10.2185/jj	日本農村医学会雑誌(0468-2513)34巻2号 Page154-165(1985.07)
1.99E+09	0	0	0	0	related dis	0	Musle ske	others	静岡県中部		岡 昭(静岡	日本農村医学会雑誌(0468-	原著論文	女性(衛生			10.2185/jj	日本農村医学会雑誌(0468-2513)34巻2号 Page166-175(1985.07)
1.99E+09	0	0	0	0	rk and bala	0	WFC	WFC	農家主婦の		二塚 信(徳	日本農村医学会雑誌(0468-	原著論文	女性(衛生			10.2185/jj	日本農村医学会雑誌(0468-2513)34巻2号 Page193-208(1985.07)
1.99E+09	0	0	0	0	related dis	0	Musle ske	MusleSke	農村進出工	愛媛県松山	原田 規章(	日本農村医学会雑誌(0468-	原著論文	女性(衛生			10.2185/jj	日本農村医学会雑誌(0468-2513)34巻2号 Page100-103(1985.07)
1.99E+09	0	0	0	0	egnancy ar	0	mental he	mental he	勤労妊婦の		上羽 英子(	助産婦(0389-9063)40巻1号	原著論文	疲労; 妊産		ヒト; 妊娠		助産婦(0389-9063)40巻1号 Page27-34(1986.01)
1.99E+09	0	0	0	0	egnancy ar	0	mental he	mental he	勤労妊婦の		上羽 英子(	助産婦(0389-9063)40巻2号	原著論文	疲労; 妊産		ヒト; 妊娠		助産婦(0389-9063)40巻2号 Page24-28(1986.02)
1.99E+09	0	0	0	0	egnancy ar	0	work, nutr	work, nutr	[妊産褥婦		鈴木 三郎(	ペリネイタルケア(0910-87	原著論文	産褥(組織		看護		ペリネイタルケア(0910-8718)4巻6号 Page598-607(1985.06)
1.99E+09	0	0	0	0	egnancy ar	0	work, nutr	work, nutr	妊婦栄養に		石井 和(女	母性衛生(0388-1512)27巻	原著論文	栄養生理学		ヒト; 妊娠		母性衛生(0388-1512)27巻1号 Page80-90(1986.03)
1.99E+09	0	0	0	0	related dis	2	Musle ske	MusleSke	病院看護婦	公立病院の	久繁 哲徳(	労働科学(0022-443X)61巻	原著論文	看護師(衛		ヒト; 女; 看		労働科学(0022-443X)61巻11号 Page517-528(1985.11)
1.99E+09	0	0	0	0	egnancy ar	0	pregnancy	pregnancy	妊婦の労働		平岡 友良(	母性衛生(0388-1512)27巻	原著論文	労働衛生;		ヒト; 妊娠		母性衛生(0388-1512)27巻2号 Page246-251(1986.06)
2.01E+09	0	0	0	0	opause, an	0	menstrati	menopaus	地域基盤型	北海道在住	島 明子(北	女性心身医学(1345-2894)	原著論文/	質問紙法;	地域差	ヒト; 中年	10.18977/	女性心身医学(1345-2894)12巻1-2号 Page299-308(2007.04)
1.99E+09	0	0	0	0	egnancy ar	0	mental he	mental he	出産体験を		新小田 春	ペリネイタルケア(0910-87	原著論文	妊産婦(心		ヒト; 妊娠		ペリネイタルケア(0910-8718)6巻6号 Page584-590(1987.06)
1.99E+09	0	0	0	0	egnancy ar	0	work, nutr	work, nutr	古典的労働		吉村 正(吉	ペリネイタルケア(0910-87	原著論文	妊産婦(組		ヒト; 妊娠		ペリネイタルケア(0910-8718)6巻12号 Page1346-1353(1987.12)
1.99E+09	0	0	0	0	related dis	0	Musle ske	MusleSke	給食産業に	手指変形だ	野崎 達夫(	産業医学ジャーナル(0388-	原著論文	給食; 職業				産業医学ジャーナル(0388-337X)10巻1号 Page39-53(1987.01)
1.99E+09	0	0	0	0	rk and bala	0	workstyle	workstyle	農村におけ	島根県下3	中川 昭生(	日本農村医学会雑誌(0468-	原著論文	農業; 地方		ヒト	10.2185/jj	日本農村医学会雑誌(0468-2513)36巻2号 Page61-68(1987.07)
1.99E+09	0	0	0	0	rk and bala	0	workstyle	workstyle	女性歯科医	4歯科大学	小島 登喜	歯学(0029-8484)75巻1号	原著論文	歯科医師(		歯学		歯学(0029-8484)75巻1号 Page180-187(1987.06)
1.99E+09	0	0	0	0	opause, an	0	urination	尿失禁	就労女性に	就労女性9	加藤 久美	日本泌尿器科学会雑誌(002	原著論文	尿失禁(調		ヒト; 女	10.5980/jı	日本泌尿器科学会雑誌(0021-5287)77巻9号 Page1501-1505(1986.09)
1.99E+09	0	0	0	0	ed disease,	0	lifestyle	others	農村におけ	広島,昭和,	高科 成良(	日本農村医学会雑誌(0468-	原著論文	健康調査;		ヒト	10.2185/jj	日本農村医学会雑誌(0468-2513)36巻6号 Page1174-1190(1988.03)
1.99E+09	0	0	0	0	egnancy ar	2	work, nutr	work, nutr	妊婦への喫	昭和59年7	中村 正和(	厚生の指標(0452-6104)35	原著論文	タバコ喫煙		ヒト; 妊娠		厚生の指標(0452-6104)35巻6号 Page23-30(1988.06)
2.01E+09	0	0	0	0	lental healt	1	occupatio	SOC	ストレス対	学歴、現在	戸ヶ里 泰	社会医学研究(0910-9919)	原著論文/	質問紙法;		ヒト; 成人		社会医学研究(0910-9919)26巻2号 Page45-52(2009.06)
1.99E+09	0	0	0	0	egnancy ar	0	BF	BF	母乳栄養と		沢田 啓司(	ペリネイタルケア(0910-87	原著論文	労働衛生;		ヒト; 女; 看		ペリネイタルケア(0910-8718)8巻2号 Page140-144(1989.02)
2.01E+09	0	0	0	0	rk and bala	0	workstyle	workstyle	総合病院勤	総合病院で	谷原 弘之(	医学と生物学(0019-1604)	原著論文/	質問紙法;	実態調査	ヒト; 成人		医学と生物学(0019-1604)152巻3号 Page94-102(2008.03)
1.99E+09	0	0	0	0	related dis	1	VDT	VDT	自覚症状調	A:事務用電	平尾 登美(	日本災害医学会会誌(0386-	原著論文	徴候と症状				日本災害医学会会誌(0386-975X)36巻12号 Page861-867(1988.12)
2.01E+09	0	0	0	0	rk and bala	1	WFC	WFC	成人期女性	本研究は、	松浦 素子(	パーソナリティ研究(1348-	原著論文	*葛藤; 質問	Temperan	ヒト; 成人	10.2132/p	パーソナリティ研究(1348-8406)16巻2号 Page149-158(2008.01)
1.99E+09	0	0	0	0	ed disease,	1	LRD	HTN	中年女性を	一企業に勤	駒井 恵美	日本公衆衛生雑誌(0546-17	原著論文	Gamma-G		ヒト; 女		日本公衆衛生雑誌(0546-1766)36巻7号 Page406-412(1989.07)
1.99E+09	0	0	0	0	egnancy ar	1	work, nutr	work, nutr	勤労妊婦の	正常妊娠中	野村 秀幸(	厚生年金病院年報(0388-23	原著論文	栄養調査;		ヒト; 妊娠		厚生年金病院年報(0388-2314)16巻 Page197-201(1990.03)
1.99E+09	0	0	0	0	related dis	1	Musle ske	MusleSke	働く女性の	腰痛症や頸	小野 雄一	体力研究(0389-9071)75号	原著論文	筋骨格系疾		ヒト	10.20793/	体力研究(0389-9071)75号 Page22-32(1990.03)
1.99E+09	0	0	0	0	related dis	1	shift	shift	看護者の健	交代制勤務	黒田 健治(	心身医学(0385-0307)31巻	原著論文	精神看護;		ヒト; 看護	10.15064/	心身医学(0385-0307)31巻4号 Page293-298(1991.04)
1.99E+09	0	0	0	0	ed disease,	0	physical a	exercise	勤労者にお	平成2年4月	大久保 雅	日本災害医学会会誌(0386-	原著論文	運動療法		ヒト		日本災害医学会会誌(0386-975X)39巻6号 Page313-317(1991.06)
1.99E+09	0	0	0	0	ed disease,	1	LRD	CVD	職種ならび	対象はあさ	森河 裕子(	日本循環器管理研究協議会	原著論文	労働衛生;		ヒト	10.11381/	日本循環器管理研究協議会雑誌(0914-7284)26巻2号 Page81-86(1991.10)
1.99E+09	0	0	0	0	ed disease,	1	lifestyle	others	労働者の健	労働者の健	丸山 総一	日本衛生学雑誌(0021-508	原著論文	健康; 労働		ヒト	10.1265/jj	日本衛生学雑誌(0021-5082)45巻6号 Page1082-1094(1991.02)
1.99E+09	0	0	0	0	egnancy ar	0	work, nutr	work, nutr	産むための		森山 郁子(	ペリネイタルケア(0910-87	原著論文	家族計画;		ヒト; 妊娠		ペリネイタルケア(0910-8718)11巻1号 Page23-30(1992.01)
1.99E+09	0	0	0	0	opause, an	0	urination	尿失禁	千葉県の就	千葉県内6	西脇 哲二(	母性衛生(0388-1512)33巻	原著論文	尿失禁(調		看護		母性衛生(0388-1512)33巻2号 Page247-252(1992.06)
2.01E+09	0	0	0	0	rk and bala	0	workstyle	workstyle	保険薬剤師	調剤薬局に	中嶋 正憲(	日本薬剤師会雑誌(0369-67	原著論文/	質問紙法;		ヒト; 成人		日本薬剤師会雑誌(0369-674X)60巻4号 Page483-488(2008.04)
2.01E+09	0	0	0	0	related dis	0	shift	shift	看護・介護	山口リハビ	岡村 久美	看護管理(0917-1355)20巻	原著論文/	質問紙法;		ヒト; 成人	10.11477/	看護管理(0917-1355)20巻1号 Page68-69(2010.01)
2E+09	0	0	0	0	rk and bala	0	rework	rework	母親の疲労	職場に復帰	外間 登美	母性衛生(0388-1512)39巻	原著論文	質問紙法;		ヒト; 女; 看		母性衛生(0388-1512)39巻4号 Page333-336(1998.12)
2E+09	0	0	0	0	lental healt	0	stress ma	social cap	中年期女性	1)フルタイ	吉井 清子(	日本公衆衛生雑誌(0546-17	原著論文	質問紙法;		ヒト; 中年		日本公衆衛生雑誌(0546-1766)46巻10号 Page869-882(1999.10)
2E+09	0	0	0	0	lental healt	0	occupatio	biomarker	就労者の唾	20代から5	織田 弥生(	人間工学(0549-4974)36巻	原著論文	HPLC; Hy	労働者	ヒト; 成人	10.5100/jj	人間工学(0549-4974)36巻6号 Page287-297(2000.12)
2E+09	0	0	0	0	lental healt	1	occupational stress		有職者の精	有職者のE	北村 眞弓(	日本看護医療学会雑誌(134	原著論文	質問紙法;	感情プロフ	ヒト; 看護	10.11477/	日本看護医療学会雑誌(1345-2606)2巻2号 Page7-15(2000.12)
1.99E+09	0	0	0	0	egnancy ar	0	work, nutr	work, nutr	就労妊婦の		佐道 正彦(	産婦人科の実際(0558-472	原著論文	妊娠(統計		ヒト; 妊娠		産婦人科の実際(0558-4728)43巻3号 Page367-373(1994.03)
2E+09	0	0	0	0	lental healt	0	occupational stress		日勤女性労		中田 光紀(	行動医学研究(1341-6790)	原著論文	質問紙法;		ヒト; 女	10.11331/	行動医学研究(1341-6790)7巻1号 Page39-46(2001.05)
2E+09	0	0	0	0	related dis	1	shift	shift	不規則交代		石東 嘉和(	体力研究(0389-9071)85号	原著論文	覚醒; 労働		ヒト; 女	10.20793/	体力研究(0389-9071)85号 Page73-80(1994.03)
2E+09	0	0	0	0	lental healt	1	occupatio	fatigue	工場におけ	P工場の女	名取 徳彦(	日本医事新報(0385-9215)	原著論文	労働衛生;		ヒト; 女		日本医事新報(0385-9215)3680号 Page47-49(1994.11)
2E+09	0	0	0	0	related dis	0	vibration	others	林業労働者	林業労働経	車谷 典男(	労働科学(0022-443X)70巻	原著論文	職業病; 林		ヒト		労働科学(0022-443X)70巻8号 Page376-387(1994.08)
2E+09	0	0	0	0	lental healt	1	occupatio	lifestyle a	女性看護職	看護職にお	中尾 久子(	臨床環境医学(0916-9407)	原著論文	質問紙法;		ヒト; 成人		臨床環境医学(0916-9407)12巻1号 Page8-14(2003.06)
2E+09	0	0	0	0	n/ satisfac	0	satisfaction		女性就労者	対象は某百	宮川 路子(	ストレス科学(1349-4813)	原著論文	労働衛生;	労働者	ヒト; 女		ストレス科学(1349-4813)9巻4号 Page61-69(1995.03)
2E+09	0	0	0	0	rk and bala	0	WFC	child-rear	子供を持つ	子供をもつ	須藤 綾子(	産業衛生学雑誌(1341-072	原著論文	労働衛生;	労働者	ヒト; 女	10.1539/s	産業衛生学雑誌(1341-0725)37巻4号 Page245-252(1995.07)
2E+09	0	0	0	0	ed disease,	0	diet and n	bone	中高年女性	中高年女性	梶田 悦子(	日本衛生学雑誌(0021-508	原著論文	光子吸収分		ヒト; 中年	10.1265/jj	日本衛生学雑誌(0021-5082)50巻4号 Page893-900(1995.10)
2E+09	0	0	0	0	ed disease,	1	physical a	physical a	身体活動の	自動車部品	佐々木 千	日本衛生学雑誌(0021-508	原著論文	労働衛生;	労働者	ヒト	10.1265/jj	日本衛生学雑誌(0021-5082)50巻4号 Page876-885(1995.10)
2E+09	0	0	0	0	related dis	1	Musle ske	MusleSke	腰痛経験に	特別養護老	衣笠 隆(筑	体育学研究(0484-6710)40	原著論文	介護者; 加		ヒト; 女	10.5432/jj	体育学研究(0484-6710)40巻3号 Page151-160(1995.09)
2E+09	0	0	0	0	ed disease,	1	other	lifestyle	職場の人間	平成5年度	中田 嘉夫(	日本災害医学会会誌(0386-	原著論文	飲酒; タバ		ヒト		日本災害医学会会誌(0386-975X)43巻11号 Page769-779(1995.11)
2E+09	0	0	0	0	related dis	1	Musle ske	MusleSke	冷凍食品加	最近1ヵ月	溝上 哲也(	産業衛生学雑誌(1341-072	原著論文	筋骨格系;	労働者	ヒト; 女		産業衛生学雑誌(1341-0725)38巻5号 Page223-229(1996.09)
2E+09	0	0	0	0	egnancy ar	0	BF	BF	勤労婦人に	１２７名7	鳥越 郁代(	母性衛生(0388-1512)37巻	原著論文	女性; 母乳		ヒト; 女; 看		母性衛生(0388-1512)37巻4号 Page423-429(1996.12)
2E+09	0	0	0	0	related dis	1	Musle ske	MusleSke	障害児学校	障害児学校	垰田 和史(	労働科学(0022-443X)73巻	原著論文	質問紙法;		ヒト		労働科学(0022-443X)73巻1号 Page1-22(1997.01)
2E+09	0	0	0	0	ed disease,	1	physical a	exercise	運動習慣な	習慣的身体	田畑 泉(国	Osteoporosis Japan(0919-	原著論文	骨密度; 習		ヒト; 中年		Osteoporosis Japan(0919-6307)4巻3号 Page548-555(1996.07)
2.01E+09	0	0	0	0	lental healt	4	occupatio	sleep	ストレッチ	就床前の他	永松 俊哉(	体力研究(0389-9071)106号	原著論文/	質問紙法;		ヒト; 成人	10.20793/	体力研究(0389-9071)106号 Page1-8(2008.04)
2E+09	0	0	0	0	rk and bala	0	workstyle	workstyle	日本におけ	現在の業務	田中 朱美(	医学教育(0386-9644)28巻	原著論文	質問紙法;		ヒト; 女	10.11307/	医学教育(0386-9644)28巻3号 Page181-186(1997.06)
2E+09	0	0	0	0	rk and bala	3	WFC	child-rear	母親の疲労	32人の母親	外間 登美	母性衛生(0388-1512)38巻	原著論文	質問紙法;		ヒト; 女; 看		母性衛生(0388-1512)38巻2号 Page179-181(1997.06)
2E+09	0	0	0	0	ed disease,	0	lifestyle	others	某大学歯学	大学臨床系	岸本 美香	口腔衛生学会雑誌(0023-28	原著論文	健康; 歯科		ヒト; 歯学	10.5834/jo	口腔衛生学会雑誌(0023-2831)47巻3号 Page240-249(1997.07)
2E+09	0	0	0	0	opause, an	0	menstrati	月経	勤労女性の	勤労女性(	古橋 信晃(	産婦人科の実際(0558-472	原著論文	質問紙法;		ヒト; 女		産婦人科の実際(0558-4728)46巻12号 Page1883-1888(1997.11)
2E+09	0	0	0	0	ed disease,	1	LRD	lipid	就業女性に	成人病健診	高成 秀樹(	社会保険医学雑誌(0911-11	原著論文	労働衛生;		ヒト; 成人		社会保険医学雑誌(0911-1158)37巻1号 Page8-13(1997.12)
2E+09	0	0	0	0	related dis	0	Musle ske	MusleSke	母子保健	腰痛発症に	古橋 信晃(	産婦人科治療(0558-471X)	原著論文	質問紙法;		ヒト; 女; 看		産婦人科治療(0558-471X)76巻2号 Page236-239(1998.02)
2E+09	0	0	0	0	related dis	0	Musle ske	MusleSke	勤労女性の	アンケート	古橋 信晃(	産婦人科の実際(0558-472	原著論文	健康調査;		ヒト; 女		産婦人科の実際(0558-4728)47巻3号 Page417-422(1998.03)
2E+09	0	0	0	0	ed disease,	1	physical a	exercise	労働者の平	製造業関係	入江 正洋(	産業衛生学雑誌(1341-072	原著論文	健康; 歩行	労働者	ヒト	10.1539/s	産業衛生学雑誌(1341-0725)40巻1号 Page7-14(1998.01)
2E+09	0	0	0	0	ed disease,	1	lifestyle	alcohol	女性有職者	女性有職者	中村 敏昭(	精神医学(0488-1281)40巻	原著論文	Aldehyde	労働者	ヒト; 女	10.11477/	精神医学(0488-1281)40巻5号 Page525-530(1998.05)
2.01E+09	0	0	0	0	lental healt	0	occupatio	fatigue	総合病院に	総合病院に	市江 和子(	日赤医学(0387-1215)59巻	原著論文	質問紙法;	実態調査;	ヒト; 成人		日赤医学(0387-1215)59巻2号 Page459-467(2009.03)
2E+09	0	0	0	0	rk and bala	1	WFC	caregiver	介護休業制	就業しなた	田崎 美弥	日本社会精神医学会雑誌(0	原著論文	介護者; 看		ヒト; 女; 看		日本社会精神医学会雑誌(0919-1372)6巻2号 Page171-184(1998.02)
2E+09	0	0	0	0	opause, an	0	menopaus	教育	就業更年期	1)更年期症	渡利 恵理	アルメイダ医報(0913-734	原著論文	健康教育(		ヒト; 女		アルメイダ医報(0913-7343)23巻2号 Page152-154(1998.06)
2E+09	0	0	0	0	egnancy ar	0	BF	BF	4ヵ月健診	1)仕事と也	福士 真都	健生病院医報(0289-3304)	原著論文	質問紙法;		ヒト; 女		健生病院医報(0289-3304)24巻 Page71-74(1998.03)
2E+09	0	0	0	0	opause, an	0	urination	尿失禁	小・中学校	尿失禁の列	小林 益江(	母性衛生(0388-1512)39巻	原著論文	教員; 女性		ヒト; 女; 看		母性衛生(0388-1512)39巻4号 Page370-374(1998.12)
2.01E+09	0	0	0	0	lental healt	0	occupatio	fatigue	総合病院に	看護職678	市江 和子(	日赤医学(0387-1215)59巻	原著論文/	質問紙法;	労働時間	ヒト; 成人		日赤医学(0387-1215)59巻2号 Page469-473(2009.03)
2E+09	0	0	0	0	rk and bala	1	workstyle	workstyle	女性の職業	対象は大学	黄 ぴん淑(	日本社会精神医学会雑誌(0	原著論文	自己概念;		ヒト; 成人		日本社会精神医学会雑誌(0919-1372)7巻1号 Page29-39(1998.09)
2E+09	0	0	0	0	rk and bala	1	workstyle	workstyle	女性の就労	対象を管理	西松 能子(	日本社会精神医学会雑誌(0	原著論文	自己概念;		ヒト; 女		日本社会精神医学会雑誌(0919-1372)7巻3号 Page239-249(1999.02)
2E+09	0	0	0	0	rk and bala	1	WFC	child-rear	働く女性の		山内 葉月(	公衆衛生(0368-5187)63巻	原著論文	育児; 育児		ヒト; 女	10.11477/	公衆衛生(0368-5187)63巻8号 Page590-593(1999.08)
2E+09	0	0	0	0	ed disease,	1	physical a	physical a	女性勤労者		須山 靖男(	体力研究(0389-9071)97号	原著論文	女性; ライ	労働者	ヒト; 女	10.20793/	体力研究(0389-9071)97号 Page17-21(1999.06)
2E+09	0	0	0	0	rk and bala	0	WFC	child-rear	就業してい		羽生田 護	ペリネイタルケア(0910-87	原著論文	育児(調査		ヒト; 女; 看		ペリネイタルケア(0910-8718)19巻1号 Page49-53(2000.01)
2E+09	0	0	0	0	rk and bala	0	WFC	child-rear	乳幼児を持つ母		村上 京子(	小児保健研究(0037-4113)	原著論文	児童心理学		ヒト; 女		小児保健研究(0037-4113)59巻2号 Page342-349(2000.03)
2E+09	0	0	0	0	n/ satisfac	0	career	就労格差	医学部・医	1998年9～	杉浦 ミド	医学教育(0386-9644)31巻	原著論文	質問紙法;			10.11307/	医学教育(0386-9644)31巻2号 Page87-91(2000.04)
2.01E+09	0	0	0	0	lental healt	0	occupatio	physical a	勤労者にお	勤労者にお	永松 俊哉(	体力研究(0389-9071)111号	原著論文	飲酒; *運動		ヒト; 成人	10.20793/	体力研究(0389-9071)111号 Page16-19(2013.04)
2E+09	0	0	0	0	ed disease,	0	oral healtl	oral	職域におけ	男性の20~	吉野 浩一(	歯科学報(0037-3710)100巻	原著論文	疫学; 成人		ヒト; 歯学		歯科学報(0037-3710)100巻3号 Page247-254(2000.03)
2E+09	0	0	0	0	opause, an	1	menopaus	stress	有職女性の	対象は看護	宮岡 久子(	母性衛生(0388-1512)41巻	原著論文	更年期; 自		ヒト; 女; 看		母性衛生(0388-1512)41巻2号 Page283-289(2000.06)
2.01E+09	0	0	0	0	lental healt	4	stress management		職場におけ	低強度認知	衛藤 理砂(	認知療法研究(1883-2296)(	原著論文/	*健康教育		ヒト; 男; 女		認知療法研究(1883-2296)6巻2号 Page150-152(2013.08)
2.01E+09	0	0	0	0	k environm	1	productivi	absenteei	健康職場推	目的:健康	土屋 政雄(	労働安全衛生総合研究所特	原著論文/	*労働衛生		ヒト; 成人		労働安全衛生総合研究所特別研究報告(1882-8329)42号 Page127-133(2012.11)
2E+09	0	0	0	0	ed disease,	0	oral healtl	oral	職域の歯科	健康保険法	吉野 浩一(	歯科学報(0037-3710)100巻	原著論文	歯科診療費	受療行動	ヒト; 歯学		歯科学報(0037-3710)100巻7号 Page699-705(2000.07)
2E+09	0	0	0	0	related dis	0	Musle ske	MusleSke	某企業関連	平成10年度	萱岡 道泰(	日本職業・災害医学会会誌	原著論文	質問紙法;		ヒト		日本職業・災害医学会会誌(1345-2592)48巻3号 Page248-254(2000.05)
2E+09	0	0	0	0	rk and bala	1	WFC	WFC	共働き世帯	因子分析、	福丸 由佳(	家族心理学研究(0915-062	原著論文	質問紙法;		ヒト		家族心理学研究(0915-0625)14巻2号 Page151-162(2000.11)
2E+09	0	0	0	0	ed disease,	0	lifestyle	smoke	職場の喫煙	パートを含	青木 つね	地域環境保健福祉研究(134	原著論文	タバコ喫煙		ヒト		地域環境保健福祉研究(1343-9286)4巻1号 Page82-85(2000.08)
2E+09	0	0	0	0	opause, an	0	menstrati	月経	客室乗務員	女性客室乗	田嶋 富美	航空医学実験隊報告(0023-	原著論文	月経; 航空		ヒト; 女		航空医学実験隊報告(0023-2858)40巻1号 Page17-26(2000.04)
2E+09	0	0	0	0	related dis	0	Musle ske	finger	水産加工業	水産加工業	中原 慶亮(	日本職業・災害医学会会誌	原著論文	水産業; 手		ヒト		日本職業・災害医学会会誌(1345-2592)48巻1号 Page61-65(2000.01)
2.02E+09	0	0	0	0	rk and bala	3	WFC	child-rear	幼児をもつ	1歳6ヵ月児	園田 和子(	小児保健研究(0037-4113)	原著論文	*育児; 家族		ヒト; 成人		小児保健研究(0037-4113)75巻1号 Page34-39(2016.01)
2E+09	0	0	0	0	opause, an	1	menstrati	月経	20～30歳代	20～39歳(	関 愼二(日	女性心身医学(1345-2894)	原著論文	月経前症候		ヒト; 女	10.18977/	女性心身医学(1345-2894)5巻2号 Page142-149(2000.12)
2E+09	0	0	0	0	ed disease,	0	other	checkup	職域におけ	東京都産業	牧野 茂徳(	産業医学ジャーナル(0388-	原著論文	労働衛生;		ヒト		産業医学ジャーナル(0388-337X)24巻2号 Page12-19(2001.03)
2E+09	0	0	0	0	egnancy ar	0	balance w	balance w	出産経験者	出産経験を	中山 和美(	母性衛生(0388-1512)42巻	原著論文	意識; 家族		ヒト; 女; 看		母性衛生(0388-1512)42巻1号 Page222-229(2001.03)
2E+09	0	0	0	0	n/ satisfac	3	education	医療従事者	看護婦の！	看護婦は相	丸山 貴美	看護展望(0385-549X)26巻	原著論文	質問紙法;		ヒト; 看護		看護展望(0385-549X)26巻5号 Page618-623(2001.04)
2E+09	0	0	0	0	rk and bala	0	workstyle	workstyle	要因分解法		坂井 尚子(	社会薬学(0911-0585)18巻	原著論文	医師; 歯科		ヒト; 女; 歯		社会薬学(0911-0585)18巻1号 Page23-35(1999.12)
2E+09	0	0	0	0	egnancy ar	1	mental he	mental he	妊娠期・出	札幌市内8	丸山 知子(	女性心身医学(1345-2894)(	原著論文	質問紙法;	自己評価式	ヒト; 妊娠	10.18977/	女性心身医学(1345-2894)6巻1号 Page93-99(2001.06)
2E+09	0	0	0	0	opause, an	1	menopaus	更年期	努力報酬不	努力報酬不	荒木 葉子(	産業医学ジャーナル(0388-	原著論文	大うつ病性		ヒト; 女		産業医学ジャーナル(0388-337X)24巻4号 Page23-29(2001.07)
2.02E+09	0	0	0	0	ed disease,	1	physical a	exercise	勤労者の労	勤労者の労	道下 竜馬(	産業衛生学雑誌(1341-072	原著論文/	*運動負荷	*労働時間	ヒト; 成人	10.1539/s	産業衛生学雑誌(1341-0725)58巻1号 Page11-20(2016.01)
2E+09	0	0	0	0	rk and bala	1	WFC	WFC	未婚女性の	東京都内の	彭 潤希(順	厚生の指標(0452-6104)48	原著論文	育児; 結婚		ヒト; 青年		厚生の指標(0452-6104)48巻1号 Page26-32(2001.01)
2E+09	0	0	0	0	related dis	0	Musle ske	MusleSke	製造業を中	企業関連病	萱岡 道泰(	日本腰痛学会雑誌(1345-9(	原著論文	産業医学;		ヒト	10.3753/y	日本腰痛学会雑誌(1345-9074)7巻1号 Page94-99(2001.10)
2E+09	0	0	0	0	related dis	0	Musle ske	MusleSke	頸肩腕症候	従業員1万	多田 浩一(	臨床整形外科(0557-0433)	原著論文	質問紙法;	頸肩腕症候	ヒト	10.11477/	臨床整形外科(0557-0433)36巻11号 Page1247-1255(2001.11)
2E+09	0	0	0	0	ed disease,	1	lifestyle	others	企業労働者	1998～199	馬越 順子(	臨床成人病(0387-1231)31	原著論文	環境; 健康	労働者	ヒト		臨床成人病(0387-1231)31巻10号 Page1357-1362(2001.10)
2E+09	0	0	0	0	related dis	0	Musle ske	MusleSke	理学療法士	職業性筋骨	笠原 敏史(	日本職業・災害医学会会誌	原著論文	筋骨格系疾		ヒト		日本職業・災害医学会会誌(1345-2592)49巻6号 Page585-589(2001.11)
2E+09	0	0	0	0	k environm	0	retention	turnover	女性介護者	就労してい	植田 恵子(	日本在宅ケア学会誌(1346-	原著論文	質問紙法;		ヒト; 女; 看		日本在宅ケア学会誌(1346-9649)5巻1号 Page67-75(2001.12)
2E+09	0	0	0	0	n/ satisfac	0	education		市町村自治	市町村自治	福永 一郎(	公衆衛生(0368-5187)66巻	原著論文	質問紙法;		ヒト; 看護	10.11477/	公衆衛生(0368-5187)66巻6号 Page445-448(2002.06)
2E+09	0	0	0	0	egnancy ar	1	work, nutr	work, nutr	妊娠・出産	高知県の保	東條 公美(	日本ウーマンズヘルス学会	原著論文	意識; ライ		ヒト; 妊娠		日本ウーマンズヘルス学会誌(1347-5894)1巻 Page59-66(2002.04)
2E+09	0	0	0	0	related dis	0	comfortab	cold	夏期の冷房	女性の冷房	井奈波 良	日本職業・災害医学会会誌	原著論文	季節; 労働	アメニティ	ヒト		日本職業・災害医学会会誌(1345-2592)50巻2号 Page113-120(2002.03)
2E+09	0	0	0	0	rk and bala	0	WFC	child-rear	勤労者女性	勤労者女性	山崎 剛(大	小児保健研究(0037-4113)(	原著論文	質問紙法;		ヒト; 女		小児保健研究(0037-4113)61巻4号 Page561-567(2002.07)
2E+09	0	0	0	0	related dis	1	shift	shift	三交替勤務	愛知県A病	佐藤 美紀(	日本看護医療学会雑誌(134	原著論文/	質問紙法;		ヒト; 女; 看	10.11477/	日本看護医療学会雑誌(1345-2606)4巻1号 Page28-35(2002.06)
2.02E+09	0	0	0	0	lental healt	0	stress management		介護職の職	介護職者に	太田 真貴(	産業ストレス研究(1340-77	原著論文/	リラクゼー	やる気	ヒト; 成人		産業ストレス研究(1340-7724)25巻4号 Page421-433(2018.11)
2E+09	0	0	0	0	rk and bala	1	WFC	child-rear	都市の社会		佐藤 秀紀(	日本保健福祉学会誌(1340-	原著論文/	育児; 社会		ヒト; 成人	10.20681/	
2E+09	0	0	0	0	rk and bala	0	workstyle	workstyle	女性によ	在宅ワーク	高橋 誠(大	労働科学(0022-443X)78巻	原著論文/	質問紙法;		ヒト; 女		労働科学(0022-443X)78巻4号 Page189-202(2002.07)
2E+09	0	0	0	0	rk and bala	0	WFC	child-rear	母親の育児		佐々木 綾	日本母性看護学会誌(1345-	原著論文/	質問紙法;		ヒト; 成人		日本母性看護学会誌(1345-773X)2巻1号 Page27-38(2001.07)
2E+09	0	0	0	0	rk and bala	1	WFC	child-rear	出産・育児	出生率をノ	滋野 由紀	社会保障研究(0387-3064)	原著論文	質問紙法;		ヒト; 成人		社会保障研究(0387-3064)39巻1号 Page43-54(2003.06)
2E+09	0	0	0	0	rk and bala	6	WFC	child-rear	働く母の育	公立保育所	中添 和代(	地域環境保健福祉研究(134	原著論文	*育児; 児童		ヒト; 成人		地域環境保健福祉研究(1343-9286)6巻2号 Page39-46(2003.03)
2E+09	0	0	0	0	ed disease,	1	LRD	glucose	山形県の産	全国の労働	若林 一郎(	厚生の指標(0452-6104)50	原著論文/	栄養調査;		ヒト; 成人		厚生の指標(0452-6104)50巻6号 Page24-31(2003.06)
2E+09	0	0	0	0	ed disease,	3	LRD	HTN	職業要因と	製造業に従	三浦 克之(	産業医学ジャーナル(0388-	原著論文/	危険因子;	労働時間	ヒト; 成人		産業医学ジャーナル(0388-337X)26巻4号 Page53-58(2003.07)

1.99E+09	0	0	0	0	lental healt	1	scale development o		農村におい	1)茨城県西	佐藤 親次(	社会精神医学(0387-2564)1	原著論文	うつ病(病		ヒト; 中年		社会精神医学(0387-2564)10巻1号 Page68-77(1987.03)
2E+09	0	0	0	0	rk and bala	0	WFC	child-rear	乳幼児を抱	「1歳6ヵ月	田中 満由美	母性衛生(0388-1512)44巻	原著論文	質問紙法;	家事	ヒト; 成人		母性衛生(0388-1512)44巻2号 Page281-288(2003.06)
2E+09	0	0	0	0	regnancy ar	0	safety, wc	safety, wc	女性小児科	女性医師の	小林 邦彦(	日本医事新報(0385-9215)4	原著論文	質問紙法;		ヒト; 妊娠		日本医事新報(0385-9215)4133号 Page59-62(2003.07)
1.99E+09	0	0	0	0	lental healt	1	occupational stress		先端産業従	先端産業に	原谷 隆史(	産業医学ジャーナル(0388-	原著論文	うつ病; 労				産業医学ジャーナル(0388-337X)11巻5号 Page34-39(1988.09)
2E+09	0	0	0	0	opause, an	0	hormone t	ホルモン	低用量ピル	低用量ピル	小林 拓郎(	産婦人科の世界(0386-987	原著論文	質問紙法;		ヒト; 青年		産婦人科の世界(0386-9873)55巻9号 Page1051-1056(2003.09)
2E+09	0	0	0	0	regnancy ar	1	safety, wc	safety, wc	就業や産科	周産・育児	濱 耕子(長	周産期医学(0386-9881)33	原著論文/	質問紙法;		ヒト; 妊娠		周産期医学(0386-9881)33巻9号 Page1177-1182(2003.09)
2E+09	0	0	0	0	related dis	3	Musle ske	MusleSke	生活協同組	2002年8月	井奈波 良一	日本職業・災害医学会会誌	原著論文/	温度; 肩; 看	夏季	ヒト; 成人		日本職業・災害医学会会誌(1345-2592)51巻5号 Page358-363(2003.09)
2E+09	0	0	0	0	opause, an	0	menstrati	月経	腹部所見を	腹部所見を	松生 恒夫(	新薬と臨牀(0559-8672)52	原著論文	*月経前症		ヒト; 成人		新薬と臨牀(0559-8672)52巻12号 Page1690-1697(2003.12)
2E+09	0	0	0	0	related dis	1	Musle ske	MusleSke	勤労者腰痛	勤労者に生	竹光 義治(	日本職業・災害医学会会誌	原著論文/	質問紙法;	患者情報;	ヒト; 成人		日本職業・災害医学会会誌(1345-2592)51巻4号 Page298-306(2003.07)
2E+09	0	0	0	0	rk and bala	0	WFC	child-rear	女性医師の	自治医科大	大木 いずみ	医学教育(0386-9644)34巻	原著論文	質問紙法;	地域資源	ヒト; 成人	10.11307/	医学教育(0386-9644)34巻5号 Page343-348(2003.10)
2E+09	0	0	0	0	related dis	1	Organic	Organic	仏壇製造に	標題の影響	嶽石 美和子	産業衛生学雑誌(1341-072	原著論文	Toluene(有	自律神経機	ヒト; 成人	10.1539/s	産業衛生学雑誌(1341-0725)45巻5号 Page194-196(2003.09)
1.99E+09	0	0	0	0	regnancy ar	0	balance w	balance w	労働形態別	妊婦149名	羽生田 護(	母性衛生(0388-1512)30巻	原著論文	労働衛生;		ヒト; 妊娠		母性衛生(0388-1512)30巻2号 Page294-298(1989.06)
2E+09	0	0	0	0	regnancy ar	6	safety, wc	safety, wc	産前産後の	産前の就業	濱 耕子(長	母性衛生(0388-1512)44巻	原著論文	*育児; 意		ヒト; 妊娠		母性衛生(0388-1512)44巻4号 Page385-393(2003.12)
2E+09	0	0	0	0	ed disease,	0	oral healt	oral	口腔衛生教	職域の40歳	高田 康光(	厚生の指標(0452-6104)51	原著論文/	患者教育;		ヒト; 成人		厚生の指標(0452-6104)51巻3号 Page25-29(2004.03)
2E+09	0	0	0	0	related dis	1	Musle ske	MusleSke	勤労者に伴	勤労者に伴	富永 俊克(	日本職業・災害医学会会誌	原著論文/	質問紙法;	SF-36; 労	ヒト; 成人		日本職業・災害医学会会誌(1345-2592)51巻6号 Page423-427(2003.11)
1.99E+09	0	0	0	0	lental healt	0	occupatio	personal c	労働ストレ	総合検診を	福西 勇夫(	タイプA(0917-1223)3巻1号	原著論文	A型人格;		ヒト		タイプA(0917-1223)3巻1号 Page79-82(1992.05)
2E+09	0	0	0	0	ed disease,	1	lifestyle	others	一般職域集	一般人口の	松下 年子(	日本社会精神医学会雑誌(0	原著論文/	質問紙法;	SF-36	ヒト; 成人		日本社会精神医学会雑誌(0919-1372)12巻3号 Page285-295(2004.03)
2E+09	0	0	0	0	related dis	3	Musle ske	MusleSke	新入社員の	高卒繊維加	熊澤 光正(	労働科学(0022-443X)79巻	原著論文/	意識性; *労		ヒト; 成人		労働科学(0022-443X)79巻5号 Page251-260(2003.11)
2E+09	0	0	0	0	related dis	0	shift	shift	看護職の交	総合病院に	中山 晃志(	看護管理(0917-1355)14巻	原著論文	質問紙法;		ヒト; 女; 看	10.11477/	看護管理(0917-1355)14巻5号 Page408-411(2004.05)
2E+09	0	0	0	0	n/ satisfac	0	career	医療従事者	介護支援専	佐賀県の介	木村 裕美(	日本看護福祉学会誌(1344-	原著論文	質問紙法;	実態調査	ヒト; 成人		日本看護福祉学会誌(1344-4875)9巻2号 Page64-74(2004.03)
1.99E+09	0	0	0	0	lental healt	0	occupatio	predictor	女性医師の	女性医師の	忠井 俊明(	社会精神医学(0387-2564)1	原著論文	医師; うつ		ヒト; 女		社会精神医学(0387-2564)15巻4号 Page296-301(1992.12)
2E+09	0	0	0	0	regnancy ar	0	safety, wc	safety, wc	産前産後休	産後1年6ヵ	濱 耕子(長	母性衛生(0388-1512)45巻	原著論文/	質問紙法;	実態調査	ヒト; 妊娠		母性衛生(0388-1512)45巻1号 Page77-86(2004.04)
1.99E+09	0	0	0	0	lental healt	1	occupational stress		テクノスト	テクノ症的	春日 伸予(	心身医学(0385-0307)32巻	原著論文	コンピュー		ヒト	10.15064/	心身医学(0385-0307)32巻7号 Page547-551(1992.10)
2E+09	0	0	0	0	n/ satisfac	1	career	医療従事者	看護職のキ	看護師のキ	山内 京子(	看護学統合研究(1346-069	原著論文	質問紙法;	看護実践	ヒト; 成人		看護学統合研究(1346-0692)5巻2号 Page6-17(2004.03)
2E+09	0	0	0	0	opause, an	0	menopaus	menopaus	更年期から	更年期外来	川島 由起子	更年期と加齢のヘルスケア	原著論文/	Calcium;	地域差	ヒト; 成人		更年期と加齢のヘルスケア(1347-801X)3巻1号 Page112-117(2004.04)
2E+09	0	0	0	0	rk and bala	0	rework	rework	脳卒中発症	脳卒中症例	平松 和嗣	リハビリテーション医学(0	原著論文/	質問紙法;	機能的自立	ヒト; 成人	10.2490/jj	リハビリテーション医学(0034-351X)41巻7号 Page465-471(2004.07)
2E+09	0	0	0	0	regnancy ar	0	safety, wc	safety, wc	就業女性の	産前休業に	濱 耕子(長	母性衛生(0388-1512)45巻	原著論文	意識; 母性		ヒト; 成人		母性衛生(0388-1512)45巻2号 Page233-245(2004.07)
2E+09	0	0	0	0	lental healt	1	occupational stress		テクノスト	テクノ症的	春日 伸予(	心身医学(0385-0307)34巻	原著論文	コンピュー		ヒト; 成人	10.15064/	心身医学(0385-0307)34巻6号 Page451-455(1994.08)
2.01E+09	0	0	0	0	opause, an	0	menstrati	月経	本邦におい	25～45歳	川瀬 良美(	女性心身医学(1345-2894)	原著論文/	黄体期; 経		ヒト; 成人	10.18977/	女性心身医学(1345-2894)9巻2号 Page119-133(2004.07)
2.01E+09	0	0	0	0	opause, an	1	menstrati	月経	成熟期女性	141名の日	森 和代(桜	女性心身医学(1345-2894)	原著論文/	質問紙法;		ヒト; 成人	10.18977/	女性心身医学(1345-2894)9巻2号 Page134-145(2004.07)
2.01E+09	0	0	0	0	related dis	1	injury	injury	看護師の暴	最近6ヵ月	吉田 由紀(	精神医学(0488-1281)46巻	原著論文	質問紙法;	強迫症状(	ヒト; 成人	10.11477/	精神医学(0488-1281)46巻7号 Page723-730(2004.07)
2.01E+09	0	0	0	0	related dis	0	shift	shift	交代制勤務	日勤-深夜	藤内 美保(	日本看護研究学会雑誌(028	原著論文/	質問紙法;	生活時間	ヒト; 成人	10.15065/	日本看護研究学会雑誌(0285-9262)27巻4号 Page17-24(2004.09)
2.01E+09	0	0	0	0	n/ satisfac	1	satisfactic	医療従事者	女性看護師	本研究では	澤田 忠幸(	日本看護研究学会雑誌(028	原著論文/	因子分析;		ヒト; 成人	10.15065/	日本看護研究学会雑誌(0285-9262)27巻4号 Page45-52(2004.09)
2.01E+09	0	0	0	0	related dis	1	shift	shift	交代制勤務	公立の総合	藤内 美保(	日本公衆衛生雑誌(0546-17	原著論文/	育児; *看護	家事; 生活	ヒト; 女; 看	10.11236/	日本公衆衛生雑誌(0546-1766)51巻10号 Page874-883(2004.10)
2.01E+09	0	0	0	0	rk and bala	0	WFC	child-rear	乳幼児をも	育児期の女	平岡 康子(	小児保健研究(0037-4113)(	原著論文/	質問紙法;		ヒト; 女		小児保健研究(0037-4113)63巻6号 Page647-652(2004.11)
2.01E+09	0	0	0	0	rk and bala	0	WFC	child-rear	産後の就業		濱 耕子(長	保健の科学(0018-3342)47	原著論文/	質問紙法;	実態調査	ヒト; 成人		保健の科学(0018-3342)47巻1号 Page63-69(2005.01)
2.01E+09	0	0	0	0	rk and bala	0	workstyle	workstyle	女性麻酔科	麻酔科にお	上田 聡子(	日本臨床麻酔学会誌(0285-	原著論文	質問紙法;	家事	ヒト; 成人	10.2199/jj	日本臨床麻酔学会誌(0285-4945)24巻10号 Page573-578(2004.12)
2.01E+09	0	0	0	0	opause, an	1	menstrati	月経	就業女性に	月経随伴症	大川 洋子(	母性衛生(0388-1512)45巻	原著論文/	質問紙法;		ヒト; 青年		母性衛生(0388-1512)45巻4号 Page414-422(2005.01)
2E+09	0	0	0	0	ed disease,	1	LRD	lipid	仕事におけ	健康な男性	戎 利光(福	保健の科学(0018-3342)39	原著論文	Cholester		ヒト		保健の科学(0018-3342)39巻4号 Page279-282(1997.04)
2E+09	0	0	0	0	lental healt	1	occupational stress		看護婦の	宮崎県内の	細見 潤(宮	看護研究(0022-8370)32巻	原著論文	看護師(調		ヒト; 看護	10.11477/	看護研究(0022-8370)32巻6号 Page497-505(1999.12)
2.01E+09	0	0	0	0	lental healt	1	overtime a	overwork	長時間残業	勤労者250	大久保 靖	産業精神保健(1340-2862)	原著論文	*労働衛生	自己評価	ヒト; 成人		産業精神保健(1340-2862)12巻4号 Page271-276(2004.12)
2.01E+09	0	0	0	0	lental healt	1	overtime a	overwork	長時間残業	大阪府ここ	夏目 誠(大	産業精神保健(1340-2862)	原著論文	A型人格; *	労働時間	ヒト; 成人		産業精神保健(1340-2862)12巻4号 Page277-290(2004.12)
2E+09	0	0	0	0	regnancy ar	0	fertility		CMIによる	外来初診時	山口 美穂(	日本受精着床学会雑誌(091	原著論文	コーネル医		ヒト; 女		日本受精着床学会雑誌(0914-6776)17巻1号 Page218-220(2000.02)
2E+09	0	0	0	0	lental healt	1	occupatio	personal c	【現代女性	「日本の産	土井 由利子	タイプA(0917-1223)11巻1	原著論文/	A型人格(		ヒト; 女		タイプA(0917-1223)11巻1号 Page25-31(2000.05)
2.01E+09	0	0	0	0	n/ satisfac	1	career	医療従事者	国公私立大	国公立大学	長友 みゆき	病院管理(0386-9571)42巻	原著論文/	質問紙法;		ヒト; 成人		病院管理(0386-9571)42巻1号 Page89-97(2005.01)
2.01E+09	0	0	0	0	opause, an	0	menstrati	月経	20歳代から	子宮癌検診	武田 卓(大	産婦人科の進歩(0370-844	原著論文	質問紙法;		ヒト; 成人	10.11437/	産婦人科の進歩(0370-8446)57巻1号 Page68-69(2005.02)
2.01E+09	0	0	0	0	rk and bala	0	workstyle	workstyle	資格職とし		造酒 錦代(	介護福祉学(1340-8178)10	原著論文/	質問紙法;	実態調査;	ヒト; 成人		介護福祉学(1340-8178)10巻1号 Page104-110(2003.10)
2E+09	0	0	0	0	lental healt	0	scale development o		バーンアウ	Maslachと	原田 和宏(	東京保健科学学会誌(1344-	原著論文	介護者; 心	共分散	ヒト	10.24531/	東京保健科学学会誌(1344-3844)3巻1号 Page38-46(2000.06)
2.01E+09	0	0	0	0	n/ satisfac	0	career	医療従事者	看護職者の	看護職者の	寺門 とも	日本赤十字看護学会誌(134	原著論文	質問紙法;		ヒト; 成人		日本赤十字看護学会誌(1346-1346)5巻1号 Page106-116(2005.03)
2E+09	0	0	0	0	lental healt	0	occupational stress		A県におけ	回答のあっ	竹村 祥恵(	日本社会精神医学会雑誌(0	原著論文	学校保健;	GHQ (精神	ヒト		日本社会精神医学会雑誌(0919-1372)9巻1号 Page1-10(2000.07)
2.01E+09	0	0	0	0	rk and bala	1	WFC	child-rear	小児科医の	2002年に	上原 里程(	日本小児科学会雑誌(0001-	原著論文	育児; *医師	育児相談	ヒト; 成人		日本小児科学会雑誌(0001-6543)109巻4号 Page530-535(2005.04)
2E+09	0	0	0	0	lental healt	0	occupational stress		A県の中規	某県内の従	竹村 祥恵(	日本社会精神医学会雑誌(0	原著論文	健康調査;	GHQ (精神	ヒト; 男; 女		日本社会精神医学会雑誌(0919-1372)10巻1号 Page33-45(2001.07)
2.01E+09	0	0	0	0	opause, an	0	menopaus	social fact	日本の都市	日本の都市	工藤 芳子(	日本更年期医学会雑誌(091	原著論文/	質問紙法;	地域差; ほ	ヒト; 中年		日本更年期医学会雑誌(0919-5033)13巻1号 Page47-54(2005.04)
2.01E+09	0	0	0	0	ed disease,	0	physical a	exercise	10年間にお	勤労者の	坂手 誠治(	体育の科学(0039-8985)55	原著論文/	加齢; 健康	体力テスト	ヒト; 成人		体育の科学(0039-8985)55巻6号 Page463-469(2005.06)
2.01E+09	0	0	0	0	n/ satisfac	0	career	医療従事者	歯科衛生士	1952～19	吉田 直美(	口腔病学会雑誌(0300-914	原著論文	質問紙法;		ヒト; 成人		口腔病学会雑誌(0300-9149)71/72巻4/1号 Page77-83(2005.03)
2E+09	0	0	0	0	ed disease,	2	lifestyle	smoke	病院勤務者	中部地方の	河野 由理(	日本公衆衛生雑誌(0546-17	原著論文	看護師; タ		ヒト; 看護	10.11236/	日本公衆衛生雑誌(0546-1766)49巻2号 Page126-131(2002.02)
2E+09	0	0	0	0	lental healt	0	occupational stress		勤労者の被	地方中規模	渡邉 温知(	産業精神保健(1340-2862)	原著論文/	質問紙法;	GHQ (精神	ヒト; 男; 女		産業精神保健(1340-2862)10巻2号 Page71-82(2002.06)
2.01E+09	0	0	0	0	n/ satisfac	0	career	就労格差	女性医師の	医師・歯科	長瀬 啓介(	日本臨床麻酔学会誌(0285-	原著論文	*病院診療		ヒト; 成人	10.2199/jj	日本臨床麻酔学会誌(0285-4945)25巻5号 Page487-493(2005.09)
2.01E+09	0	0	0	0	related dis	1	Organic	benzen	ppbレベル	ppbレベル	井上 修(東	日本職業・災害医学会会誌	原著論文	*Benzene	Phenylme	ヒト; 成人		日本職業・災害医学会会誌(1345-2592)53巻1号 Page6-11(2005.01)
2E+09	0	0	0	0	lental healt	1	occupational stress		病院勤務者	病院勤務者	増田 安代(	日本保健福祉学会誌(1340-	原著論文/	看護師; 労	GHQ (精神	ヒト; 女	10.20681/	日本保健福祉学会誌(1340-8194)9巻1号 Page15-24(2002.10)
2.01E+09	0	0	0	0	related dis	4	Musle ske	MusleSke	腰痛症に対	介護職に多	徳森 公彦(	日本職業・災害医学会会誌	原著論文	介護者; *住		ヒト; 男; 女		日本職業・災害医学会会誌(1345-2592)53巻3号 Page171-175(2005.05)
2.01E+09	0	0	0	0	rk and bala	0	workstyle	workstyle	次世代育成	若手医師の	大澤 真木子	小児科臨床(0021-518X)58	原著論文	質問紙法;		ヒト; 成人		小児科臨床(0021-518X)58巻11号 Page2325-2332(2005.11)
2.01E+09	0	0	0	0	n/ satisfac	0	career	医療従事者	女性医師の	平成12年度	長瀬 啓介(	性差と医療(1349-4589)2巻	原著論文	病院診療科		ヒト; 成人		性差と医療(1349-4589)2巻9号 Page1095-1100(2005.08)
2E+09	0	0	0	0	lental healt	3	other	gender	性役割と職	Demand-(	森 美加(東	産業精神保健(1340-2862)	原著論文/	質問紙法;		ヒト; 成人		産業精神保健(1340-2862)11巻2号 Page71-78(2003.06)
2.01E+09	0	0	0	0	related dis	1	Musle ske	MusleSke	介護職員の	介護職員の	廣橋 容子(	介護福祉学(1340-8178)12	原著論文	質問紙法;	介護負担	ヒト; 青年		介護福祉学(1340-8178)12巻1号 Page118-125(2005.10)
2.01E+09	0	0	0	0	rk and bala	1	WFC	child-rear	子供を持つ	子どもをも	大西 久男(	Journal of Rehabilitation a	原著論文	質問紙法;		ヒト; 成人		Journal of Rehabilitation and Health Sciences(1348-6756)1巻 Page42-48(2003.12)
2.01E+09	0	0	0	0	regnancy ar	1	fertility		【周産期の	不妊検査・	小泉 智恵(	周産期医学(0386-9881)35	原著論文/	質問紙法;	GHQ (精神	ヒト; 成人		周産期医学(0386-9881)35巻10号 Page1377-1383(2005.10)
2.01E+09	0	0	0	0	regnancy ar	0	balance w	balance w	【生活,環境	看護師の妊	小林 康祐(	周産期学シンポジウム(134	原著論文/	質問紙法;		ヒト; 妊娠		周産期学シンポジウム(1342-0526)23号 Page93-99(2005.09)
2.01E+09	0	0	0	0	ed disease,	1	physical a	exercise	20歳代・3	20歳代・3	中谷 久恵(	日本地域看護学会誌(1346-	原著論文	質問紙法;	実態調査;	ヒト; 成人	10.20746/	日本地域看護学会誌(1346-9657)8巻1号 Page41-45(2005.09)
2.01E+09	0	0	0	0	ed disease,	1	physical a	exercise	健常若年者	生体の運動	竹谷 晋二(	理学療法学(0289-3770)32	原著論文	*運動負荷	データ相関	ヒト; 成人	10.15063/	理学療法学(0289-3770)32巻7号 Page429-432(2005.12)
2E+09	0	0	0	0	lental healt	0	occupational stress		A県の中規	A県内の従	竹村 祥恵(	日本社会精神医学会雑誌(0	原著論文/	質問紙法;	GHQ (精神	ヒト; 女		日本社会精神医学会雑誌(0919-1372)12巻1号 Page1-12(2003.07)
2.01E+09	0	0	0	0	related dis	0	Musle ske	MusleSke	【肩こりの	30代の看護	矢吹 省司(	脊椎脊髄ジャーナル(0914-	原著論文/	質問紙法;	医療行為;	ヒト; 成人		脊椎脊髄ジャーナル(0914-4412)18巻12号 Page1208-1213(2005.12)
2.01E+09	0	0	0	0	rk and bala	0	WFC	caregiver	就労者の家	都市部大学	美ノ谷 新子	日本看護学会誌(0917-513	原著論文	質問紙法;	介護負担;	ヒト; 成人		日本看護学会誌(0917-513X)14巻2号 Page118-129(2005.03)
2.01E+09	0	0	0	0	opause, an	0	menstrati	月経	看護師の夜	近年働く女	尾谷 功(和	日本職業・災害医学会会誌	原著論文/	Melatonin		ヒト; 成人		日本職業・災害医学会会誌(1345-2592)53巻6号 Page284-288(2005.11)
2.01E+09	0	0	0	0	opause, an	0	Breast_ca	乳がん	40歳代超音	40歳代女性	栗山 進一(	日本乳癌検診学会誌(0918-	原著論文	質問紙法;		ヒト; 成人	10.3804/jj	日本乳癌検診学会誌(0918-0729)15巻1号 Page100-105(2006.03)
2.01E+09	0	0	0	0	opause, an	1	menopaus	menopaus	更年期女性	研究目的は	梅野 貴恵(	母性衛生(0388-1512)47巻	原著論文/	質問紙法;	*Health L	ヒト; 成人		母性衛生(0388-1512)47巻1号 Page143-152(2006.04)
2.01E+09	0	0	0	0	n/ satisfac	1	career	医療従事者	日本小児歯	女性歯科医	森 榮(森歯	小児歯科学雑誌(0583-119	原著論文	質問紙法;	実態調査;	ヒト; 成人	10.11411/	小児歯科学雑誌(0583-1199)44巻1号 Page31-36(2006.03)
2.01E+09	0	0	0	0	opause, an	0	menstrati	月経	働く女性の	本研究は,	大平 肇子(	人間工学(0549-4974)42巻	原著論文/	*リラクゼ	計算; 自律	ヒト; 成人	10.5100/jj	人間工学(0549-4974)42巻2号 Page105-111(2006.04)
2.01E+09	0	0	0	0	n/ satisfac	0	career	医療従事者	A病院に勤	看護師の職	永野 光子(	医療看護研究(1349-8630)	原著論文	質問紙法;		ヒト; 成人		医療看護研究(1349-8630)2巻1号 Page59-65(2006.03)
2.01E+09	0	0	0	0	regnancy ar	2	work, nutr	work, nutr	非妊娠時の	褥婦1192	鈴木 史明(	産婦人科の進歩(0370-844	原著論文/	質問紙法;	実態調査	ヒト; 妊娠	10.11437/	産婦人科の進歩(0370-8446)58巻2号 Page120-129(2006.05)
2E+09	0	0	0	0	rk and bala	1	WFC	WFC	乳幼児をも	補償モデル	福丸 由佳(	心理臨床学研究(0289-192	原著論文/	育児; 心理	家庭	ヒト; 成人		心理臨床学研究(0289-1921)21巻4号 Page416-421(2003.10)
2.01E+09	0	0	0	0	opause, an	1	menopaus	osteoporo	30歳前後の	主に閉経前	小宮 康裕(	日本衛生学雑誌(0021-508	原著論文/	回帰分析;		ヒト; 成人	10.1265/jj	日本衛生学雑誌(0021-5082)61巻3号 Page327-331(2006.05)
2.01E+09	0	0	0	0	lental healt	1	occupatio	biomarker	睡眠時間と	睡眠時間と	松本 由紀(	日本衛生学雑誌(0021-508	原著論文/	バイオマー		ヒト; 成人	10.1265/jj	日本衛生学雑誌(0021-5082)61巻3号 Page357-365(2006.05)
2.01E+09	0	0	0	0	ed disease,	0	lifestyle	others	一食品製造	年中無休,	新井 俊彦(	総合健診(1347-0086)33巻	原著論文	*ライフス		ヒト; 成人	10.7143/jl	総合健診(1347-0086)33巻3号 Page396-399(2006.05)
2E+09	0	0	0	0	lental healt	1	occupational stress		介護支援専	ケアマネー	古瀬 みど	日本在宅ケア学会誌(1346-	原著論文	在宅介護支		ヒト; 成人		日本在宅ケア学会誌(1346-9649)7巻1号 Page61-67(2003.12)
2.01E+09	0	0	0	0	rk and bala	0	workstyle	workstyle	女性医師活	2002年の	児玉 知子(	日本医事新報(0385-9215)4	原著論文	労働衛生;	地域差; 労	ヒト; 成人		日本医事新報(0385-9215)4279号 Page74-79(2006.04)
2.01E+09	0	0	0	0	lental healt	1	occupatio	predictor	職域におけ	職域におけ	鈴木 恭子(	お茶の水医学雑誌(0472-46	原著論文/	質問紙法;		ヒト; 成人		お茶の水医学雑誌(0472-4674)54巻2号 Page71-77(2006.06)
2.01E+09	0	0	0	0	ed disease,	1	lifestyle	smoke	臨床応用	CYP2A6遺	渡辺 洋子(	DNA多型(2188-3815)14巻	原著論文	Nicotine;	Steroid 7	ヒト; 成人		DNA多型(2188-3815)14巻 Page256-259(2006.05)
2.01E+09	0	0	0	0	rk and bala	0	workstyle	workstyle	女性麻酔科	女性麻酔科	上田 聡子(	日本臨床麻酔学会誌(0285-	原著論文	質問紙法;		ヒト; 成人	10.2199/jj	日本臨床麻酔学会誌(0285-4945)26巻4号 Page418-428(2006.07)
2E+09	0	0	0	0	lental healt	0	other	gender	中高年期世	中高年の職	合田 加代	地域環境保健福祉研究(134	原著論文/	質問紙法;		ヒト; 成人		地域環境保健福祉研究(1343-9286)7巻1号 Page19-23(2004.03)
2.01E+09	0	0	0	0	ed disease,	3	lifestyle	obesity	日本人40歳	S健康事業	桂 敏樹(京	日本健康医学会雑誌(1343-	原著論文/	コホート研		ヒト; 成人	10.20685/	日本健康医学会雑誌(1343-0025)15巻1号 Page24-28(2006.04)
2E+09	0	0	0	0	lental healt	0	occupational stress		介護支援専	関東地方の	豊嶋 三枝	日本看護福祉学会誌(1344-	原著論文	質問紙法;		ヒト; 成人		日本看護福祉学会誌(1344-4875)9巻2号 Page127-135(2004.03)
2.01E+09	0	0	0	0	opause, an	0	menopaus	social fact	都市部に居	都市部に居	坂根 綾子(	更年期と加齢のヘルスケア	原著論文/	質問紙法;	労働時間	ヒト; 中年		更年期と加齢のヘルスケア(1347-801X)5巻1号 Page56-62(2006.06)
2.01E+09	0	0	0	0	ed disease,	1	lifestyle	alcohol	日本人女性	定期健診を	若林 一郎(	日本老年医学会雑誌(0300-	原著論文/	*飲酒; 加齢		ヒト; 成人	10.3143/g	日本老年医学会雑誌(0300-9173)43巻4号 Page525-530(2006.07)
2.01E+09	0	0	0	0	n/ satisfac	6	education	health prc	ポジティブ	ポジティブ	新納 美美(	日本地域看護学会誌(1346-	原著論文	意欲; *コミ	GHQ (精神	ヒト; 成人	10.20746/	日本地域看護学会誌(1346-9657)8巻2号 Page5-13(2006.03)
2.01E+09	0	0	0	0	rk and bala	1	WFC	child-rear	産後3ヵ月	産後3ヵ月	榮 玲子(香	小児保健研究(0037-4113)(	原著論文/	質問紙法;		ヒト; 成人		小児保健研究(0037-4113)65巻2号 Page306-313(2006.03)
2.01E+09	0	0	0	0	related dis	5	shift	shift	交替制勤務	MEDLINE	荒川 千秋(	日本看護管理学会誌(1347-	原著論文/	*看護師; *		ヒト; 男; 女	10.19012/	日本看護管理学会誌(1347-0140)10巻1号 Page30-36(2006.08)
2E+09	0	0	0	0	k environm	0	productivi	absenteei	精神障害に	10企業にお	島 悟(東京	産業精神保健(1340-2862)	原著論文/	*うつ病; 労		ヒト; 青年		産業精神保健(1340-2862)12巻1号 Page46-53(2004.03)
2.01E+09	0	0	0	0	n/ satisfac	1	satisfaction		主観的健康	目的　金融	豊川 智之(	厚生の指標(0452-6104)53	原著論文/	*健康への		ヒト; 成人		厚生の指標(0452-6104)53巻10号 Page1-6(2006.09)
2E+09	0	0	0	0	lental healt	0	occupational stress		高度救命救	大学附属病	岡本 博照(	産業精神保健(1340-2862)	原著論文/	質問紙法;	労働時間	ヒト; 成人		産業精神保健(1340-2862)12巻1号 Page54-60(2004.03)
2.01E+09	0	0	0	0	rk and bala	1	WFC	caregiver	家族介護者	目的　介護	平松 誠(日	厚生の指標(0452-6104)53	原著論文/	質問紙法;	*介護負担	ヒト; 中年		厚生の指標(0452-6104)53巻11号 Page19-24(2006.10)
2.01E+09	0	0	0	0	ed disease,	1	LRD	metabolic	職域におけ	虚血性心疾	山本 修一郎	肥満研究(1343-229X)12巻	原著論文/	*Insulin(血	ホメオスタ	ヒト; 成人		肥満研究(1343-229X)12巻2号 Page152-158(2006.08)
2E+09	0	0	0	0	lental healt	1	occupational stress		共分散構造	労働者108	中尾 久子(	臨床環境医学(0916-9407)	原著論文	*ライフス		ヒト; 成人		臨床環境医学(0916-9407)13巻1号 Page17-25(2004.06)
2.01E+09	0	0	0	0	rk and bala	0	workstyle	workstyle	女性医師調	僻地勤務を	大木 いずみ	日本医事新報(0385-9215)4	原著論文	質問紙法;	介護負担;	ヒト; 成人		日本医事新報(0385-9215)4302号 Page68-72(2006.10)
2.01E+09	0	0	0	0	opause, an	0	Breast_ca	乳がん	職域におけ	目的:乳が,	橋口 克頼(	人間ドック(1880-1021)21	原著論文	*乳房腫瘍		ヒト; 成人	10.11320/	人間ドック(1880-1021)21巻3号 Page656-660(2006.09)
2.01E+09	0	0	0	0	lental healt	0	occupational stress		職業性スト	管理職39名	石川 浩二(	産業ストレス研究(1340-77	原著論文	質問紙法;		ヒト		産業ストレス研究(1340-7724)11巻3号 Page187-192(2004.08)
2.01E+09	0	0	0	0	ed disease,	0	lifestyle	others	人間ドック	目的:働く	田村 由美	人間ドック(1880-1021)21	原著論文/	*更年期; 生	労働時間	ヒト; 成人	10.11320/	人間ドック(1880-1021)21巻3号 Page708-712(2006.09)
2.01E+09	0	0	0	0	lental healt	1	occupational stress		職業ストレ	職業性スト	松下 年子(	日本社会精神医学会雑誌(0	原著論文/	質問紙法;		ヒト; 成人		日本社会精神医学会雑誌(0919-1372)13巻1号 Page13-21(2004.07)
2.01E+09	0	0	0	0	rk and bala	0	workstyle	workstyle	50歳前後の	女性歯科医	軽部 裕代(	日本歯科医療管理学会雑誌	原著論文	質問紙法;	実態調査;	ヒト; 中年		日本歯科医療管理学会雑誌(0387-5687)41巻3号 Page180-187(2006.11)
2.01E+09	0	0	0	0	rk and bala	1	WFC	caregiver	家族介護者	家族介護者	広瀬 美千代	社会福祉学(0911-0232)47	原著論文	介護者; 個	介護負担	ヒト; 成人	10.24469/	社会福祉学(0911-0232)47巻3号 Page3-15(2006.11)
2.01E+09	0	0	0	0	n/ satisfac	0	self-effica	confidenc	農村部にお	本研究の目	宮岡 久子(	母性衛生(0388-1512)47巻	原著論文/	質問紙法;		ヒト; 成人		母性衛生(0388-1512)47巻4号 Page590-596(2007.01)
2.01E+09	0	0	0	0	lental healt	0	occupational stress		某アパレル	アパレル産	豊島 裕子(	産業衛生学雑誌(1341-072	原著論文	医師; 産業		ヒト; 成人	10.1539/s	産業衛生学雑誌(1341-0725)47巻2号 Page70-78(2005.03)
2.01E+09	0	0	0	0	related dis	0	shift	shift	女性クリニ	看護師やオ	宮内 文久(	日本職業・災害医学会会誌	原著論文/	*卵胞刺激		ヒト; 女		日本職業・災害医学会会誌(1345-2592)54巻5号 Page231-233(2006.09)
2.01E+09	0	0	0	0	related dis	1	Musle ske	MusleSke	膝関節深屈	単一造船所	山口 和博(	膝(0911-1425)30巻1号 Pa	原著論文/	労働衛生;		ヒト; 青年		膝(0911-1425)30巻1号 Page41-44(2005.10)
2.01E+09	0	0	0	0	lental healt	0	occupational stress		女性の発掘	発掘遺物整	井奈波 良一	日本職業・災害医学会会誌	原著論文/	質問紙法;		ヒト; 成人		日本職業・災害医学会会誌(1345-2592)52巻5号 Page265-269(2004.09)
2.01E+09	0	0	0	0	rk and bala	0	workstyle	workstyle	ソーシャル	社会福祉専	田中 希世子	医療と福祉(0287-1521)40	原著論文	質問紙法;	実態調査;	ヒト; 成人		医療と福祉(0287-1521)40巻2号 Page69-75(2007.03)
2.01E+09	0	0	0	0	lental healt	0	occupational stress		「勤労者心	自院で実施	太田 理恵子	日本職業・災害医学会会誌	原著論文	カウンセリ	傾聴	ヒト; 成人		日本職業・災害医学会会誌(1345-2592)52巻5号 Page315-320(2004.09)
2.01E+09	0	0	0	0	ed disease,	0	oral healt	oral	就業者にお	産業歯科保	山村 直(ラ	日本歯科衛生学会雑誌(188	原著論文	質問紙法;		ヒト; 成人		日本歯科衛生学会雑誌(1884-5193)1巻1号 Page122-123(2006.10)
2.01E+09	0	0	0	0	lental healt	1	occupational stress		ケアマネジ	ケアマネジ	田川 元也(	病院管理(0386-9571)42巻	原著論文	在宅介護支	*介護負担	ヒト; 男; 女		病院管理(0386-9571)42巻1号 Page29-39(2005.01)
2.01E+09	0	0	0	0	rk and bala	0	workstyle	workstyle	「新しい自	本研究では	藤野 善久(	産業衛生学雑誌(1341-072	原著論文	*労働衛生	*労働時間	ヒト	10.1539/s	産業衛生学雑誌(1341-0725)49巻2号 Page45-53(2007.03)
2.01E+09	0	0	0	0	related dis	0	Musle ske	MusleSke	肩こりの病	県内の病院	篠崎 哲也(	臨床整形外科(0557-0433)4	原著論文	質問紙法;		ヒト; 成人	10.11477/	臨床整形外科(0557-0433)42巻5号 Page409-412(2007.05)
2.01E+09	0	0	0	0	lental healt	1	occupational stress		青年海外協	青年海外協	加藤 章子(	産業衛生学雑誌(1341-072	原著論文/	質問紙法;	国際協力機	ヒト; 成人	10.1539/s	産業衛生学雑誌(1341-0725)46巻6号 Page191-200(2004.11)
2.01E+09	0	0	0	0	lental healt	0	occupatio	fatigue	一食品製造	食品製造業	新井 俊彦(	総合健診(1347-0086)32巻	原著論文/	質問紙法;	工場	ヒト; 成人	10.7143/jl	総合健診(1347-0086)32巻2号 Page236-243(2005.03)
2.01E+09	0	0	0	0	n/ satisfac	0	career	workstyle	形成外科女	近年、全国	平野 由美(	日本形成外科学会会誌(038	原著論文	質問紙法;		ヒト; 成人		日本形成外科学会会誌(0389-4703)27巻6号 Page417-423(2007.06)
2.01E+09	0	0	0	0	related dis	1	Musle ske	MusleSke	福祉用具の	わが国は既	冨岡 公子(	産業衛生学雑誌(1341-072	原著論文/	介護者; *椅	主観的運動	ヒト; 成人	10.1539/s	産業衛生学雑誌(1341-0725)49巻4号 Page113-121(2007.07)
2.01E+09	0	0	0	0	related dis	0	other	hypotensi	女性の静止	生産性効率	加部 勇(古	産業衛生学雑誌(1341-072	原著論文/	休息; *労働	起居動作;	ヒト; 青年	10.1539/s	産業衛生学雑誌(1341-0725)49巻4号 Page122-126(2007.07)
2.01E+09	0	0	0	0	lental healt	0	occupational stress		勤労者のメ	男性1691名	數川 悟(富	産業精神保健(1340-2862)	原著論文/	志気; 心理	GHQ (精神	ヒト; 成人		産業精神保健(1340-2862)13巻1号 Page40-48(2005.03)
2.01E+09	0	0	0	0	related dis	1	shift	shift	看護師の深	800床の大	谷口 明美(	和歌山医学(0043-0013)58	原著論文/	質問紙法;		ヒト; 成人		和歌山医学(0043-0013)58巻2号 Page60-68(2007.06)
2.01E+09	0	0	0	0	rk and bala	0	workstyle	workstyle	地域医療従	自治医科大	佐藤 正章(	地域医学(0914-4277)21巻	原著論文	質問紙法;		ヒト; 女		地域医学(0914-4277)21巻11号 Page984-988(2007.11)

2.01E+09	0	0	0	0	opause, an	0	urination		勤労女性に		別府 正典(	日本ウロギネコロジー研究	原著論文/	質問紙法;	SF-36	ヒト; 成人		日本ウロギネコロジー研究会誌2巻1号 Page57-59(2005.05)
2.01E+09	0	0	0	0	opause, an	0	menopaus	menopaus	更年期症状	更年期症状	西村 正子(	看護・保健科学研究誌(134	原著論文/	質問紙法;	実態調査	ヒト; 成人		看護・保健科学研究誌(1345-983X)7巻1号 Page33-42(2006.10)
2.01E+09	0	0	0	0	lental healt	0	stress management		医療・福祉	医療・福祉	中島 朱美(	介護福祉学(1340-8178)12	原著論文	医師; 看護		ヒト; 看護		介護福祉学(1340-8178)12巻1号 Page63-73(2005.10)
2.01E+09	0	0	0	0	ed disease,	3	LRD	glucose	空腹時血糖	大規模な事	金 大成(東	総合健診(1347-0086)34巻	原著論文/	Alanine Tı	*空腹時血	ヒト; 成人	10.7143/jl	総合健診(1347-0086)34巻6号 Page623-629(2007.11)
2.01E+09	0	0	0	0	related dis	0	Musle ske	MusleSke	大阪府内新	新設介護老	冨岡 公子(	産業衛生学雑誌(1341-072	原著論文/	質問紙法;	*頸肩腕症	ヒト; 成人	10.1539/s	産業衛生学雑誌(1341-0725)49巻5号 Page216-222(2007.09)
2.01E+09	0	0	0	0	lental healt	3	occupational stress		市町村に勤	市町村に勤	水島 ゆか	保健師ジャーナル(1348-83	原著論文/	質問紙法;	GHQ (精神	ヒト; 成人	10.11477/	保健師ジャーナル(1348-8333)62巻2号 Page146-151(2006.02)
2.01E+09	0	0	0	0	related dis	4	VDT	VDT	女性のVDT	女性事務作	川上 慶(慶	人間工学(0549-4974)43巻	原著論文	*椅子; 機器		ヒト; 女	10.5100/jj	人間工学(0549-4974)43巻5号 Page252-260(2007.10)
2.01E+09	0	0	0	0	opause, an	3	menstratio	月経	月経周辺期	月経周辺期	今田 藍(桜	女性心身医学(1345-2894)	原著論文/	*感情; 心理		ヒト; 成人	10.18977/	女性心身医学(1345-2894)12巻3号 Page498-505(2007.11)
2.01E+09	0	0	0	0	lental healt	1	occupatio	SOC	企業労働者	SOC日本語	中村 百合子	広島国際大学看護学ジャー	原著論文/	質問紙法;	労働時間;	ヒト; 成人		広島国際大学看護学ジャーナル(1349-5917)4巻 Page15-24(2007.03)
2.01E+09	0	0	0	0	rk and bala	0	workstyle	workstyle	日本の看護	女性潜在看	川口 章(同	社会保険旬報(1343-5728)	原著論文	意思決定;		ヒト; 青年		社会保険旬報(1343-5728)2344号 Page38-47(2008.03)
2.01E+09	0	0	0	0	lental healt	0	occupational stress		職業性スト	当社の九州	白原 昶(日	消化器心身医学(1340-884	原著論文/	質問紙法;		ヒト; 男; 女		消化器心身医学(1340-8844)13巻1号 Page26-30(2006.04)
2.01E+09	0	0	0	0	related dis	0	Musle ske	MusleSke	保育所に勤	保育所勤務	磯野 富美子	小児保健研究(0037-4113)	原著論文	質問紙法;	*頸肩腕症	ヒト; 成人		小児保健研究(0037-4113)66巻6号 Page789-796(2007.11)
2.01E+09	0	0	0	0	rk and bala	1	WFC	caregiver	在宅要介護	在宅要介護	牧迫 飛雄馬	日本老年医学会雑誌(0300-	原著論文/	質問紙法;	*介護負担	ヒト; 成人	10.3143/g	日本老年医学会雑誌(0300-9173)45巻1号 Page59-67(2008.01)
2.01E+09	0	0	0	0	ed disease,	1	lifestyle a	lifestyle a	仕事のスト	日本の職域	西條 泰明(	産業医学ジャーナル(0388-	原著論文/	足首; 質問	労働時間	ヒト; 成人		産業医学ジャーナル(0388-337X)29巻3号 Page69-75(2006.05)
2.01E+09	0	0	0	0	n/ satisfac	1	self-effica	motivation	保育所で働	保育所勤務	磯野 富美子	小児保健研究(0037-4113)	原著論文/	質問紙法;		ヒト; 成人		小児保健研究(0037-4113)67巻2号 Page367-374(2008.03)
2.01E+09	0	0	0	0	rk environm	0	retention	turnover	日本産科婦	産婦人科医	宮城 悦子,	日本産科婦人科学会神奈川	原著論文	質問紙法;	実態調査	ヒト; 男; 女		日本産科婦人科学会神奈川地方部会会誌(0910-2485)44巻2号 Page174-178(2008.01)
2.01E+09	0	0	0	0	rk and bala	0	workstyle	workstyle	看護師の就	背景:人口の	緒方 泰子(	医療経済研究(1340-895X)	原著論文/	環境; *看護		ヒト; 成人		医療経済研究(1340-895X)19巻3号 Page233-252(2008.03)
2.01E+09	0	0	0	0	lental healt	1	occupational stress		看護師にお	看護師にお	佐藤 育子(	ナーシング(0389-8326)26	原著論文/	質問紙法;		ヒト; 成人		ナーシング(0389-8326)26巻9号 Page98-103(2006.08)
2.01E+09	0	0	0	0	lental healt	1	stress management		勤労者のス	勤労者にお	浦川 加代子	三重看護学誌(1344-6983)	原著論文	質問紙法;	感情プロフ	ヒト; 成人		三重看護学誌(1344-6983)10巻 Page89-92(2008.03)
2.01E+09	0	0	0	0	lental healt	0	stress ma	workplace	【総合病院	総合病院勤	黒木 宣夫(	総合病院精神医学(0915-58	原著論文/	質問紙法;		ヒト; 成人		総合病院精神医学(0915-5872)18巻2号 Page122-130(2006.05)
2.01E+09	0	0	0	0	lental healt	1	other	SOC	女性看護師	本研究目的	松浦 恒仁(	富山大学看護学会誌(1882-	原著論文/	質問紙法;		ヒト; 成人		富山大学看護学会誌(1882-191X)7巻2号 Page7-14(2008.03)
2.01E+09	0	0	0	0	lental healt	0	occupatio	personal c	A総合病院	総合病院に	原田 貴史(	強迫性障害の研究7巻 Page	原著論文/	質問紙法;	*強迫症状	ヒト; 成人		強迫性障害の研究7巻 Page7-13(2006.05)
2.01E+09	0	0	0	0	lental healt	0	occupational stress		勤労者のス	心の健康づ	水野 恵理子	山梨大学看護学会誌(1347-	原著論文/	質問紙法;	*労働者	ヒト; 成人		山梨大学看護学会誌(1347-7714)6巻2号 Page31-36(2008.03)
2.01E+09	0	0	0	0	lental healt	1	occupational stress		警察官にお	本研究は、	上田 鼓(神	トラウマティック・ストレ	原著論文/	質問紙法;		ヒト; 成人		トラウマティック・ストレス(1348-0944)4巻2号 Page167-175(2006.09)
2.01E+09	0	0	0	0	egnancy ar	1	mental he	mental he	産後3ヵ月	産後3ヵ月	久保 奈美子	山梨県母性衛生学会誌(134	原著論文/	質問紙法;	自己評価式	ヒト; 成人		山梨県母性衛生学会誌(1349-4287)7巻1号 Page1-7(2008.03)
2.01E+09	0	0	0	0	n/ satisfac	1	satisfaction		労働者の主	電気機器製	池田 和子(	保健師ジャーナル(1348-83	原著論文/	質問紙法;	労働者	ヒト; 青年	10.11477/	保健師ジャーナル(1348-8333)64巻6号 Page542-547(2008.06)
2.01E+09	0	0	0	0	related dis	1	Musle ske	MusleSke	新設介護老	改善が進ま	冨岡 公子(	産業衛生学雑誌(1341-072	原著論文/	質問紙法;	介護者-患	ヒト; 青年	10.1539/s	産業衛生学雑誌(1341-0725)50巻3号 Page86-91(2008.05)
2.01E+09	0	0	0	0	related dis	4	comfortab	cold	夏期におい	【目的】夏	井垣 通人(	日本職業・災害医学会会誌	原著論文	温度感覚;	*罨法; 夏季	ヒト; 成人		日本職業・災害医学会会誌(1345-2592)55巻6号 Page233-238(2007.11)
2.01E+09	0	0	0	0	ed disease,	4	diet and n	group soc	ソーシャル	目的 本研	久保田 晃生	日本公衆衛生雑誌(0546-17	原著論文/	質問紙法;		ヒト; 成人	10.11236/	日本公衆衛生雑誌(0546-1766)55巻5号 Page327-340(2008.05)
2.01E+09	0	0	0	0	opause, an	1	menopaus	work fami	更年期女性	本研究は、	中西 伸子(	母性衛生(0388-1512)48巻	原著論文/	質問紙法;		ヒト; 成人		母性衛生(0388-1512)48巻4号 Page514-521(2008.01)
2.01E+09	0	0	0	0	n/ satisfac	0	satisfactio	well-being	看護師の心	特定機能病	酒井 淳子(	日本看護科学会誌(0287-53	原著論文	質問紙法;		ヒト; 成人	10.5630/ja	日本看護科学会誌(0287-5330)26巻3号 Page32-40(2006.09)
2.01E+09	0	0	0	0	rk and bala	6	workstyle	workstyle	既婚看護師	既婚看護師	村田 梢(大	看護・保健科学研究誌(134	原著論文	育児; *看護		ヒト; 女; 看		看護・保健科学研究誌(1345-983X)8巻1号 Page1-11(2008.02)
2.01E+09	0	0	0	0	n/ satisfac	1	self-effica	confidenc	看護師の病	本研究は、	市江 和子(	日本赤十字看護学会誌(134	原著論文/	質問紙法;		ヒト; 成人		日本赤十字看護学会誌(1346-1346)8巻1号 Page51-59(2008.03)
2.01E+09	0	0	0	0	egnancy ar	0	mental he	mental he	妊娠期の抑	近年、妊娠	安藤 智子(	小児保健研究(0037-4113)	原著論文/	葛藤; 質問	自己評価式	ヒト; 妊娠		小児保健研究(0037-4113)65巻5号 Page666-674(2006.09)
2.01E+09	0	0	0	0	ed disease,	1	lifestyle a	lifestyle a	朝食摂取と	北海道内の	西 基(北海	北海道医療大学看護福祉学	原著論文/	質問紙法;	*労働者	ヒト; 成人		北海道医療大学看護福祉学部学会誌(1349-8967)4巻1号 Page79-80(2008.03)
2.01E+09	0	0	0	0	egnancy ar	1	balance w	balance w	就労妊婦の	就労妊婦の	奥野 雅子(	母性衛生(0388-1512)49巻	原著論文/	質問紙法;	少子化	ヒト; 妊娠		母性衛生(0388-1512)49巻2号 Page245-252(2008.07)
2.01E+09	0	0	0	0	egnancy ar	0	balance w	balance w	就労妊婦の	日本におい	宮木 ゆかり	母性衛生(0388-1512)49巻	原著論文/	質問紙法;	少子化	ヒト; 妊娠		母性衛生(0388-1512)49巻2号 Page253-260(2008.07)
2.01E+09	0	0	0	0	related dis	1	Musle ske	MusleSke	移乗介助に	日本の介護	冨岡 公子(	産業衛生学雑誌(1341-072	原著論文/	*介護者; 生	リフター	ヒト; 成人	10.1539/s	産業衛生学雑誌(1341-0725)50巻4号 Page103-110(2008.07)
2.01E+09	0	0	0	0	ed disease,	1	lifestyle	others	生活習慣病	主に中年層	大塚 盛男(	日本健康医学会雑誌(1343-	原著論文/	Cholester		ヒト; 成人	10.20685/	日本健康医学会雑誌(1343-0025)15巻2号 Page2-6(2006.07)
2.01E+09	0	0	0	0	rk and bala	6	WFC	caregiver	認知症高齢	少子高齢化	渡辺 千枝子	日本看護研究学会雑誌(028	原著論文	介護者; *在	介護者-患	ヒト; 中年	10.15065/	日本看護研究学会雑誌(0285-9262)31巻4号 Page75-85(2008.09)
2.01E+09	0	0	0	0	ed disease,	1	lifestyle a	lifestyle a	事業所にお	労働衛生に	田甫 久美子	金沢大学つるま保健学会誌	原著論文/	*健康への		ヒト; 成人		金沢大学つるま保健学会誌(1346-8502)32巻1号 Page77-83(2008.07)
2.01E+09	0	0	0	0	lental healt	0	occupational stress		施設におい	施設別高齢	三徳 和子(	川崎医療福祉学会誌(0917-	原著論文/	質問紙法;	介護負担	ヒト; 成人		川崎医療福祉学会誌(0917-4605)18巻1号 Page121-128(2008.07)
2.01E+09	0	0	0	0	related dis	1	Organic	ホルムアル	病院病理室	病院病理室	横沢 冊子(	日本職業・災害医学会会誌	原著論文/	*Formalde		ヒト; 成人		日本職業・災害医学会会誌(1345-2592)56巻5号 Page198-201(2008.09)
2.01E+09	0	0	0	0	lental healt	1	occupatio	fatigue	中小企業の	中小企業3	宮島 啓子(	大阪府立公衆衛生研究所研	原著論文/	質問紙法;	*労働時間	ヒト; 成人		大阪府立公衆衛生研究所研究報告(1343-2923)46号 Page51-54(2008.10)
2.01E+09	0	0	0	0	related dis	1	VDT	VDT	細胞検査士	目的:近年、	金城 満(新	日本臨床細胞学会雑誌(038	原著論文/	質問紙法;	*細胞検査	ヒト; 成人	10.5795/jj	日本臨床細胞学会雑誌(0387-1193)47巻6号 Page407-415(2008.11)
2.01E+09	0	0	0	0	lental healt	1	occupatio	personal c	成人女性の	25〜65歳の	松浦 素子(	心理学研究(0021-5236)77	原著論文/	質問紙法;	GHQ (精神	ヒト; 成人	10.4992/jj	心理学研究(0021-5236)77巻1号 Page48-55(2006.04)
2.01E+09	0	0	0	0	rk and bala	1	WFC	WFC	ワークライ	仕事領域と	長見 まき	産業精神保健(1340-2862)	原著論文/	*葛藤; 質問	GHQ (精神	ヒト; 成人		産業精神保健(1340-2862)16巻4号 Page224-230(2008.12)
2.01E+09	0	0	0	0	n/ satisfac	1	satisfaction		都市部青壮	都市部では	高 燕(首都	社会医学研究(0910-9919)	原著論文/	質問紙法;		ヒト; 成人		社会医学研究(0910-9919)25号 Page29-35(2007.12)
2.01E+09	0	0	0	0	lental healt	1	occupatio	SOC	富山県内事	職場のメン	老子 善康(	逓信医学(0387-1320)60巻	原著論文	質問紙法;		ヒト; 成人		逓信医学(0387-1320)60巻5号 Page305-310(2008.12)
2.01E+09	0	0	0	0	opause, an	0	menstratio	月経	性成熟期女	国内在住の	五十嵐 久人	新薬と臨牀(0559-8672)58	原著論文/	質問紙法;	実態調査	ヒト; 成人		新薬と臨牀(0559-8672)58巻1号 Page27-36(2009.01)
2.01E+09	0	0	0	0	opause, an	1	menstratio	月経	性周期の睡	性周期の睡	吉田 吏江(	労働安全衛生研究(1882-68	原著論文/	*月経周期		ヒト; 成人	10.2486/jo	労働安全衛生研究(1882-6822)1巻3号 Page205-209(2008.11)
2.01E+09	0	0	0	0	egnancy ar	0	safety, wo	safety, wo	女性労働者	2005年6月	渡部 真弓(	産業医学ジャーナル(0388-	原著論文	質問紙法;		ヒト; 成人		産業医学ジャーナル(0388-337X)32巻1号 Page90-95(2009.01)
2.01E+09	0	0	0	0	rk and bala	0	workstyle	workstyle	院内保育所	医師の確保	江口 成美(	病院(0385-2377)68巻2号 P	原著論文	質問紙法;	実態調査		10.11477/	病院(0385-2377)68巻2号 Page132-136(2009.02)
2.01E+09	0	0	0	0	lental healt	0	occupational stress		病院職員の	総合病院職	池田 美樹(	日本社会精神医学会雑誌(0	原著論文/	質問紙法;	GHQ (精神	ヒト; 成人		日本社会精神医学会雑誌(0919-1372)15巻3号 Page199-207(2007.03)
2.01E+09	0	0	0	0	opause, an	0	menopaus	更年期	中高年女性	都内の40~	宮内 清子(	日本更年期医学会雑誌(091	原著論文	質問紙法;	データ相関	ヒト; 成人		日本更年期医学会雑誌(0919-5033)16巻2号 Page238-245(2008.10)
2.01E+09	0	0	0	0	related dis	4	Musle ske	MusleSke	女性労働者	女性労働者	武澤 千尋(	産業看護(1883-0501)1巻2	原著論文	*リラクゼ	VDT症候群	ヒト; 成人		産業看護(1883-0501)1巻2号 Page199-205(2009.03)
2.01E+09	0	0	0	0	rk and bala	0	workstyle	workstyle	女性医師の	2007年9月	浜本 芳彦(	日本小児科医会会報(0912-	原著論文	質問紙法;		ヒト; 成人		日本小児科医会会報(0912-1781)36号 Page133-137(2008.10)
2.01E+09	0	0	0	0	n/ satisfac	3	career	workstyle	女性医師の	本稿では、	川村 顕(医	保健医療社会学論集(1343-	原著論文/	育児; *プラ		ヒト; 成人	10.18918/	保健医療社会学論集(1343-0203)19巻2号 Page94-104(2009.02)
2.01E+09	0	0	0	0	rk and bala	0	WFC	child-rear	働く女性の		有吉 浩美(	産業保健人間工学研究(134	原著論文	*労働衛生		ヒト; 妊娠		産業保健人間工学研究(1345-1731)8巻1号 Page19-26(2007.01)
2.01E+09	0	0	0	0	rk and bala	0	workstyle	workstyle	産業の場に		有吉 浩美(	産業保健人間工学研究(134	原著論文	健康増進;		ヒト; 成人		産業保健人間工学研究(1345-1731)7巻増補 Page100-103(2005.10)
2.01E+09	0	0	0	0	rk and bala	0	workstyle	soc_sup	働く母親の		山田 英津子	産業保健人間工学研究(134	原著論文	質問紙法;	家事	ヒト; 女		産業保健人間工学研究(1345-1731)7巻増補 Page104-107(2005.10)
2.01E+09	0	0	0	0	related dis	0	Musle ske	MusleSke	漁獲物仕分		佐伯 公康(	産業保健人間工学研究(134	原著論文	水産業; *労	動作分析	ヒト; 女		産業保健人間工学研究(1345-1731)7巻増補 Page124-127(2005.10)
2.01E+09	0	0	0	0	rk and bala	0	WFC	WFC	Family to		山田 泰行(	産業保健人間工学研究(134	原著論文	質問紙法;	家庭環境;	ヒト; 成人		産業保健人間工学研究(1345-1731)8巻増補 Page93-96(2006.11)
2.01E+09	0	0	0	0	lental healt	4	stress ma	relaxation	看護職の慢	看護師のス	柳 誠一(宇	山口県看護研究学会学術集	原著論文	*リラクゼ	腹式呼吸	ヒト; 成人		山口県看護研究学会学術集会プログラム・集録8回 Page34-36(2009.03)
2.01E+09	0	0	0	0	egnancy ar	1	balance w	balance w	妊婦が認知	妊婦が認知	中島 久美子	母性衛生(0388-1512)50巻	原著論文	質問紙法;	家事	ヒト; 妊娠		母性衛生(0388-1512)50巻1号 Page49-56(2009.04)
2.01E+09	0	0	0	0	egnancy ar	0	balance w	balance w	産後の女性	【目的】産	松永 佳子(	母性衛生(0388-1512)50巻	原著論文	質問紙法;	育児相談;	ヒト; 成人		母性衛生(0388-1512)50巻1号 Page118-124(2009.04)
2.01E+09	0	0	0	0	n/ satisfac	1	career		行政分野で	行政保健師	大倉 美佳(	日本地域看護学会誌(1346-	原著論文/	質問紙法;	価値観	ヒト; 成人		日本地域看護学会誌(1346-9657)11巻1号 Page53-58(2008.09)
2.01E+09	0	0	0	0	related dis	0	Musle ske	MusleSke	高齢者施設	本研究は東	百瀬 由美子	日本看護福祉学会誌(1344-	原著論文/	質問紙法;	頸肩腕症候	ヒト; 成人		日本看護福祉学会誌(1344-4875)14巻2号 Page87-99(2009.03)
2.01E+09	0	0	0	0	related dis	0	comfortab	heat	「クールビ	夏期におい	榎本 ヒカル	労働安全衛生研究(1882-68	原著論文/	質問紙法;	速度; *ビル	ヒト; 成人	10.2486/jo	労働安全衛生研究(1882-6822)2巻1号 Page5-10(2009.03)
2.01E+09	0	0	0	0	related dis	1	Musle ske	MusleSke	介護労働者	介護労働者	冨岡 公子(	労働科学(0022-443X)85巻	原著論文/	*介護者; 筋		ヒト; 成人		労働科学(0022-443X)85巻1号 Page1-10(2009.02)
2.01E+09	0	0	0	0	rk and bala	6	WFC	child-rear	A新聞社に		山田 英津子	産業保健人間工学研究(134	原著論文	質問紙法;		ヒト; 成人		産業保健人間工学研究(1345-1731)8巻2号 Page50-56(2007.07)
2.01E+09	0	0	0	0	n/ satisfac	1	education	professinc	病院勤務の	看護職アイ	吉田 なよ子	日本赤十字看護学会誌(134	原著論文	質問紙法;		ヒト; 成人		日本赤十字看護学会誌(1346-1346)7巻1号 Page68-77(2007.03)
2.01E+09	0	0	0	0	opause, an	1	menopaus	osteopros	更年期女性	労働安全衛	森 佳子(東	日本食育学会誌(1882-477	原著論文/	月経; *更年	骨質; 食物	ヒト; 成人	10.14986/	日本食育学会誌(1882-4773)3巻2号 Page91-97(2009.04)
2.01E+09	0	0	0	0	lental healt	1	occupational stress		自殺多発地	自殺多発地	岩戸 清香(	岩手医学雑誌(0021-3284)	原著論文/	質問紙法;	自己評価式	ヒト; 成人		岩手医学雑誌(0021-3284)61巻1号 Page1-15(2009.04)
2.01E+09	0	0	0	0	n/ satisfac	1	satisfaction		都市部常勤	目的 本研	高 燕(首都	日本公衆衛生雑誌(0546-17	原著論文/	質問紙法;		ヒト; 成人	10.11236/	日本公衆衛生雑誌(0546-1766)56巻3号 Page155-162(2009.03)
2.01E+09	0	0	0	0	egnancy ar	0	fertility	不妊	調節卵巣刺	調節卵巣刺	清水 清美(	日本生殖看護学会誌(1884-	原著論文/	質問紙法;	待ち時間	ヒト; 成人		日本生殖看護学会誌(1884-2232)6巻1号 Page46-54(2009.06)
2.01E+09	0	0	0	0	rk and bala	0	WFC	child-rear	0〜3歳児を	本研究は0	佐々木 睦子	日本母性看護学会誌(1345-	原著論文	質問紙法;	家事; 内容	ヒト; 成人		日本母性看護学会誌(1345-773X)9巻1号 Page37-45(2009.03)
2.01E+09	0	0	0	0	lental healt	1	occupatio	predictor	企業労働者	本研究は、	横田 京子(	日本保健科学学会誌(1880-	原著論文/	自己評価(		ヒト; 成人	10.24531/	日本保健科学学会誌(1880-0211)9巻4号 Page217-224(2007.03)
2.01E+09	0	0	0	0	rk and bala	1	WFC	caregiver	在宅介護者	本研究の目	坪井 章雄(	作業療法(0289-4920)28巻	原著論文/	質問紙法;	*介護負担	ヒト; 成人	10.11477/	作業療法(0289-4920)28巻3号 Page298-308(2009.06)
2.01E+09	0	0	0	0	rk and bala	1	WFC	child-rear	働く母親の	本研究では	日下 知子(	母性衛生(0388-1512)48巻	原著論文/	質問紙法;	育児相談	ヒト; 成人		母性衛生(0388-1512)48巻2号 Page271-281(2007.07)
2.01E+09	0	0	0	0	egnancy ar	0	BF	BF	O市内に在	授乳期にあ	上原 和代(	日本母乳哺育学会雑誌(188	原著論文	質問紙法;		ヒト; 成人		日本母乳哺育学会雑誌(1882-4242)3巻1号 Page17-26(2009.06)
2.01E+09	0	0	0	0	opause, an	0	Breast_ca	乳がん	東京での職	2003年度	坂 佳奈子(	日本乳癌検診学会誌(0918-	原著論文/	触診; *乳房	精密検査	ヒト; 成人	10.3804/jj	日本乳癌検診学会誌(0918-0729)18巻2号 Page182-188(2009.06)
2.01E+09	0	0	0	0	egnancy ar	1	work, nutr	work, nutr	現代の妊婦	目的 本研	新川 治子(	日本助産学会誌(0917-635	原著論文/	質問紙法;	眠気(疲労)	ヒト; 妊娠	10.3418/jj	日本助産学会誌(0917-6357)23巻1号 Page48-58(2009.06)
2.01E+09	0	0	0	0	lental healt	1	scale development o		勤労者を対	勤労者の労	熊井 三治(	日本健康医学会雑誌(1343-	原著論文	労働衛生;		ヒト; 成人	10.20685/	日本健康医学会雑誌(1343-0025)16巻1号 Page8-16(2007.04)
2.01E+09	0	0	0	0	lental healt	0	occupatio	lifestyle a	勤労者のメ	製造業に従	梅沢 有美子	日本社会精神医学会雑誌(0	原著論文/	質問紙法;	自己評価式	ヒト; 成人		日本社会精神医学会雑誌(0919-1372)16巻2号 Page97-111(2007.11)
2.01E+09	0	0	0	0	rk and bala	1	WFC	caregiver	特別養護老	目的:A県に	木村 誠子(	老年看護学(1346-9665)12	原著論文/	質問紙法;	SF-36; 介護	ヒト; 成人	10.20696/	老年看護学(1346-9665)12巻1号 Page94-100(2007.11)
2.01E+09	0	0	0	0	lental healt	1	occupatio	sleep	睡眠不足に	目的:深夜勤	桝田 浩三(	あたらしい眼科(0910-181	原著論文/	眼圧; 睡眠		ヒト; 成人		あたらしい眼科(0910-1810)25巻1号 Page119-122(2008.01)
2.01E+09	0	0	0	0	lental healt	1	stress management		医療従事者	医療従事者	豊増 功次(	心身医学(0385-0307)48巻	原著論文/	質問紙法;	感情プロフ	ヒト; 成人	10.15064/	心身医学(0385-0307)48巻5号 Page349-358(2008.05)
2.01E+09	0	0	0	0	opause, an	0	menstratio	月経	日本女性の	日本女性の	有馬 牧子(	更年期と加齢のヘルスケア	原著論文/	質問紙法;		ヒト; 成人		更年期と加齢のヘルスケア(1347-801X)8巻1号 Page60-66(2009.06)
2.01E+09	0	0	0	0	opause, an	0	hormone t	更年期	更年期前の	更年期前の	宇山 恵子	更年期と加齢のヘルスケア	原著論文	質問紙法;		ヒト; 成人		更年期と加齢のヘルスケア(1347-801X)8巻1号 Page101-108(2009.06)
2.01E+09	0	0	0	0	lental healt	1	occupational stress		看護・介護	本研究の目	松岡 治子(	産業衛生学雑誌(1341-072	原著論文/	質問紙法;		ヒト; 成人	10.1539/s	産業衛生学雑誌(1341-0725)50巻2号 Page49-57(2008.03)
2.01E+09	0	0	0	0	lental healt	0	occupatio	predictor	精神科看護	民間の精神	野中 真由美	心身健康科学(1882-6881)	原著論文/	質問紙法;		ヒト; 成人	10.11427/	心身健康科学(1882-6881)4巻1号 Page47-53(2008.02)
2.01E+09	0	0	0	0	ed disease,	0	physical a	exercise	保健指導を	緒言:日常生	森山 賢治(	肥満研究(1343-229X)15巻	原著論文/	質問紙法;	*保健指導	ヒト; 成人		肥満研究(1343-229X)15巻2号 Page170-178(2009.08)
2.01E+09	0	0	0	0	egnancy ar	1	fertility		妊孕力の信	目的 本研	山崎 圭子(	日本母子看護学会誌(1882-	原著論文	質問紙法;	価値観	ヒト; 妊娠	10.11477/	日本母子看護学会誌(1882-2495)3巻2号 Page23-31(2009.10)
2.01E+09	0	0	0	0	lental healt	1	scale development o		労働者にお	【背景・目	山口 実穂(	The Kitakanto Medical Jo	原著論文/	質問紙法;	*自己評価	ヒト; 成人	10.2974/k	The Kitakanto Medical Journal(1343-2826)59巻3号 Page231-240(2009.08)
2.01E+09	0	0	0	0	related dis	0	Musle ske	MusleSke	腰痛研究の	これまで1	伊藤 友一(	日本腰痛学会雑誌(1345-9	原著論文	質問紙法;	実態調査	ヒト; 成人	10.3753/y	日本腰痛学会雑誌(1345-9074)15巻1号 Page39-44(2009.10)
2.01E+09	0	0	0	0	rk and bala	0	WFC	child-rear	子育てと仕	著者らは、	林田 りか(	Quality of Life Journal10巻	原著論文/	質問紙法;		ヒト; 成人		Quality of Life Journal10巻1号 Page31-40(2009.09)
2.01E+09	0	0	0	0	ed disease,	1	lifestyle	smoke	総合病院で	【目的】本	今村 桃子(	日本循環器病予防学会誌(1	原著論文/	質問紙法;	実態調査	ヒト; 成人		日本循環器病予防学会誌(1346-6267)44巻3号 Page161-168(2009.10)
2.01E+09	0	0	0	0	related dis	0	other	work man	中小食品製	労働衛生外	竹内 由利子	産業保健人間工学研究(134	原著論文	質問紙法;		ヒト; 成人		産業保健人間工学研究(1345-1731)11巻増補 Page20-23(2009.10)
2.01E+09	0	0	0	0	ed disease,	0	diet and n	diet and n	働く女性に	外食産業だ	洲崎 好香(	産業保健人間工学研究(134	原著論文	質問紙法;		ヒト; 成人		産業保健人間工学研究(1345-1731)11巻増補 Page58-60(2009.10)
2.01E+09	0	0	0	0	related dis	0	VDT	VDT	性差が副次	VDT作業た	高西 敏正(	産業保健人間工学研究(134	原著論文	因子分析;	眠気	ヒト; 成人		産業保健人間工学研究(1345-1731)11巻増補 Page66-69(2009.10)
2.01E+09	0	0	0	0	lental healt	0	occupatio	lifestyle a	看護師にお	ストレス理	川又 華代(	順天堂スポーツ健康科学研	原著論文/	質問紙法;	*運動量; 自	ヒト; 成人		順天堂スポーツ健康科学研究(1884-3808)1巻2号 Page305-306(2009.09)
2.01E+09	0	0	0	0	lental healt	1	stress management		中・高等学	中学・高等	鬼頭 愛子(	産業ストレス研究(1340-77	原著論文/	質問紙法;		ヒト; 成人		産業ストレス研究(1340-7724)15巻3号 Page213-222(2008.08)
2.01E+09	0	0	0	0	related dis	0	shift	shift	2夜の睡眠	派遣労働者	佐々木 司(	労働科学(0022-443X)86巻	原著論文	*睡眠; デパ	運動量	ヒト; 成人		労働科学(0022-443X)86巻1号 Page33-42(2010.02)
2.01E+09	0	0	0	0	lental healt	0	occupatio	SOC	【看護にS	ストレス対	竹内 朋子(	看護研究(0022-8370)42巻	原著論文/	質問紙法;	労働時間	ヒト; 成人	10.11477/	看護研究(0022-8370)42巻7号 Page517-526(2009.12)
2.01E+09	0	0	0	0	rk and bala	1	WFC	child-rear	幼児をもつ		青木 聡子(	発達心理学研究(0915-902	原著論文	質問紙法;		ヒト; 男; 女	10.11201/	発達心理学研究(0915-9029)20巻4号 Page382-392(2009.12)
2.01E+09	0	0	0	0	ed disease,	1	lifestyle	smoke	高齢者施設	【目的】本	三徳 和子(	日本禁煙学会雑誌(1882-68	原著論文/	質問紙法;		ヒト; 成人		日本禁煙学会雑誌(1882-6806)5巻1号 Page10-17(2010.03)
2.01E+09	0	0	0	0	rk and bala	0	workstyle	workstyle	看護師の「	働く女性の	杉原 トヨ子	インターナショナルNursir	原著論文/	質問紙法;		ヒト; 成人		インターナショナルNursing Care Research(1347-1341)8巻2号 Page117-128(2009.01)
2.01E+09	0	0	0	0	related dis	1	shift	shift	未病や疾病	未病や疾病	矢倉 宏祐(	日本未病システム学会雑誌	原著論文	胃潰瘍; *労		ヒト; 成人		日本未病システム学会雑誌(1347-5541)15巻2号 Page365-369(2010.03)
2.01E+09	0	0	0	0	opause, an	0	Breast_ca	乳がん	ライフスタ	京都市の乳	大越 香江(	日本乳癌検診学会誌(0918-	原著論文	質問紙法;	受診率; 精	ヒト; 成人	10.3804/jj	日本乳癌検診学会誌(0918-0729)19巻1号 Page53-59(2010.03)
2.01E+09	0	0	0	0	related dis	0	Organic	ortho-pl	内視鏡消毒	目的と方法	宮島 啓子(	産業衛生学雑誌(1341-072	原著論文/	*o-Phthal	*機器の洗	ヒト; 男; 女	10.1539/s	産業衛生学雑誌(1341-0725)52巻2号 Page74-80(2010.03)
2.01E+09	0	0	0	0	opause, an	0	Breast_ca	乳がん	乳房視触診	乳房視触診	坂 佳奈子(	日本がん検診・診断学会誌	原著論文	質問紙法;		ヒト; 成人		日本がん検診・診断学会誌(1881-8846)17巻3号 Page217-222(2010.03)
2.01E+09	0	0	0	0	n/ satisfac	1	self-effica	confidenc	介護労働者	目的 介護	谷垣 静子(	厚生の指標(0452-6104)57	原著論文/	質問紙法;		ヒト; 成人		厚生の指標(0452-6104)57巻5号 Page16-20(2010.05)
2.01E+09	0	0	0	0	related dis	0	physical s	skin	職業性皮膚	職業性皮膚	杉浦 啓二(	日本職業・環境アレルギー	原著論文	*職業病(診		ヒト; 成人		日本職業・環境アレルギー学会雑誌(1349-5461)17巻2号 Page11-16(2010.05)
2.01E+09	0	0	0	0	ed disease,	1	lifestyle	smoke	職域におい	目的:職域で	松本 泉美(	禁煙科学3巻3号 Page1-13	原著論文/	質問紙法;		ヒト; 成人		禁煙科学3巻3号 Page1-13(2010.01)
2.01E+09	0	0	0	0	related dis	4	comfortab	cold	寒冷環境下	【目的】冬	井垣 通人(	日本職業・災害医学会会誌	原著論文/	*温熱療法	夏季; 冬季	ヒト; 成人		日本職業・災害医学会会誌(1345-2592)58巻3号 Page128-134(2010.05)
2.01E+09	0	0	0	0	opause, an	0	Breast_ca	乳がん	大学病院職	順天堂大学	中井 克也(	日本乳癌検診学会誌(0918-	原著論文	質問紙法;	受診率; 受	ヒト; 成人	10.3804/jj	日本乳癌検診学会誌(0918-0729)19巻2号 Page159-163(2010.06)
2.01E+09	0	0	0	0	lental healt	1	occupatio	gender	PSWの抑う	精神保健福	岡田 栄作(	北海道医療大学看護福祉学	原著論文/	質問紙法;	*精神保健	ヒト; 成人		北海道医療大学看護福祉学部学会誌(1349-8967)6巻1号 Page93-96(2010.03)
2.01E+09	0	0	0	0	rk and bala	0	WFC	child-rear	育児中の母	本研究では	清水 嘉子(	母性衛生(0388-1512)51巻	原著論文/	質問紙法;		ヒト; 成人		母性衛生(0388-1512)51巻2号 Page367-375(2010.07)
2.01E+09	0	0	0	0	egnancy ar	1	balance w	balance w	出産・育児	本研究の目	北川 良子(	母性衛生(0388-1512)51巻	原著論文	質問紙法;	実態調査	ヒト; 成人		母性衛生(0388-1512)51巻2号 Page416-424(2010.07)
2.01E+09	0	0	0	0	egnancy ar	0	BF	BF	長期母乳育	本研究は、	梅野 貴恵(	母性衛生(0388-1512)51巻	原著論文	質問紙法;		ヒト; 成人		母性衛生(0388-1512)51巻2号 Page498-505(2010.07)
2.01E+09	0	0	0	0	lental healt	1	occupational stress		労働者のス		遠藤 架児(	臨床心理学研究(1348-122	原著論文/	質問紙法;		ヒト; 成人		臨床心理学研究(1348-1223)8号 Page77-91(2010.03)
2.01E+09	0	0	0	0	related dis	0	Musle ske	MusleSke	施設介護労	施設介護労	大西 徳明(	労働科学(0022-443X)86巻	原著論文	介護者; 筋	足底圧	ヒト; 成人		労働科学(0022-443X)86巻3号 Page139-150(2010.06)
2.01E+09	0	0	0	0	related dis	3	shift	shift	交替制勤務	本研究では	荒川 千秋(	日本看護管理学会誌(1347-	原著論文/	質問紙法;	労働時間	ヒト; 成人	10.19012/	日本看護管理学会誌(1347-0140)14巻1号 Page42-50(2010.07)
2.01E+09	0	0	0	0	rk environm	1	engagement		看護師の職	看護師の専	澤田 忠幸(	心理学研究(0021-5236)80	原著論文	因子分析;		ヒト; 成人	10.4992/jj	心理学研究(0021-5236)80巻2号 Page131-137(2009.06)
2.01E+09	0	0	0	0	egnancy ar	3	work, nutr	work, nutr	日本人正常	目的 本研	濱 耕子(愛	日本助産学会誌(0917-635	原著論文/	妊娠管理;	SF-36; 妊娠	ヒト; 妊娠	10.3418/jj	日本助産学会誌(0917-6357)24巻1号 Page96-107(2010.06)
2.01E+09	0	0	0	0	lental healt	0	occupatio	personal c	就労女性に		生越 由夏(	産業カウンセリング研究(1	原著論文/	*A型人格;	労働時間	ヒト; 成人		産業カウンセリング研究(1880-9669)11巻1号 Page23-31(2008.09)
2.01E+09	0	0	0	0	ed disease,	0	physical a	exercise	30-40歳代	クラスター	須藤 英彦(	体力科学(0039-906X)59巻	原著論文/	質問紙法;	買い物	ヒト; 成人	10.7600/js	体力科学(0039-906X)59巻3号 Page323-331(2010.06)
2.01E+09	0	0	0	0	rk and bala	0	WFC	WLB	働く女性の	働く女性の	中村 延江(	女性心身医学(1345-2894)	原著論文	質問紙法;	労働時間	ヒト; 成人	10.18977/	女性心身医学(1345-2894)15巻1号 Page91-97(2010.07)

2.01E+09	0	0	0	0	rk and bala	0	WFC	WLB	"組織力向	20～30歳	熊切 こず	日本臨床工学技士会会誌(1	原著論文	質問紙法;	*家庭環境	ヒト; 成人		日本臨床工学技士会会誌(1341-3171)37号 Page168-170(2009.10)
2.01E+09	0	0	0	0	lental healt	3	occupational stress		病院の職場	病院の職場	福岡 悦子(	地域環境保健福祉研究(134	原著論文/	質問紙法;		ヒト; 成人		地域環境保健福祉研究(1343-9286)12巻1号 Page75-85(2009.03)
2.01E+09	0	0	0	0	related dis	0	shift	shift	交代制勤務	本研究は、	吉崎 貴大(	日本栄養・食糧学会誌(028	原著論文/	質問紙法;	生活時間	ヒト; 成人	10.4327/js	日本栄養・食糧学会誌(0287-3516)63巻4号 Page161-167(2010.08)
2.01E+09	0	0	0	0	n/ satisfac	4	education	health pro	中高年女性	目的:本研究	宮内 清子(	日本健康教育学会誌(1340-	原著論文/	*健康教育	SF-36; 自	ヒト; 成人	10.11260/	日本健康教育学会誌(1340-2560)18巻3号 Page186-198(2010.08)
2.01E+09	0	0	0	0	n/ satisfac	0	satisfaction		大手教育関	大手教育関	梁 洋子(順	民族衛生(0368-9395)76巻	原著論文/	健康状態		ヒト; 青年	10.3861/js	民族衛生(0368-9395)76巻4号 Page164-173(2010.07)
2.01E+09	0	0	0	0	lental healt	1	stress management		小規模事業	メンタルヘ	洲崎 好香(	日本健康医学会雑誌(1343-	原著論文	質問紙法;		ヒト; 成人	10.20685/	日本健康医学会雑誌(1343-0025)18巻2号 Page64-69(2009.07)
2.01E+09	0	0	0	0	related dis	1	physical s	pretibial e	下肢のむく	著者らは立	須藤 元喜(	日本生理人類学会誌(1342-	原著論文/	質問紙法;		ヒト; 成人		日本生理人類学会誌(1342-3215)15巻3号 Page21-26(2010.08)
2.01E+09	0	0	0	0	n/ satisfac	0	career		宮城県にお	宮城県内に	福與 なお	日本小児科学会雑誌(0001-	原著論文	質問紙法;	実態調査;	ヒト; 成人		日本小児科学会雑誌(0001-6543)114巻10号 Page1516-1525(2010.10)
2.01E+09	0	0	0	0	opause, an	1	Uterine_ca	子宮がん	女性労働者	目的 金融	兼任 千恵(	厚生の指標(0452-6104)57	原著論文/	質問紙法;	*受療行動	ヒト; 成人		厚生の指標(0452-6104)57巻13号 Page1-7(2010.11)
2.01E+09	0	0	0	0	ed disease,	0	lifestyle	smoke	わが国の勤	わが国の勤	和田 耕治(	日本医師会雑誌(0021-449	原著論文	質問紙法;		ヒト; 成人		日本医師会雑誌(0021-4493)139巻9号 Page1894-1899(2010.12)
2.01E+09	0	0	0	0	k environm	0	retention	turnover	女性医師の	1)卒後5年	山崎 由花(	医学教育(0386-9644)41巻	原著論文/	質問紙法;		ヒト; 女	10.11307/	医学教育(0386-9644)41巻6号 Page411-416(2010.12)
2.01E+09	0	0	0	0	opause, an	0	Uterine_ca	子宮がん	企業におい	目的:企業	松浦 祐介(	日本臨床細胞学会雑誌(038	原著論文	質問紙法;	受診率	ヒト; 成人	10.5795/jj	日本臨床細胞学会雑誌(0387-1193)50巻1号 Page6-10(2011.01)
2.01E+09	0	0	0	0	rk and bala	1	WFC	caregiver	有配偶女性	本論文の目	中西 泰子(	老年社会科学(0388-2446)	原著論文/	意識; 親子		ヒト; 成人	10.34393/	老年社会科学(0388-2446)32巻4号 Page413-421(2011.01)
2.01E+09	0	0	0	0	rk and bala	6	WFC	child-rear	子育てと仕	本研究の目	藤本 美由	母性衛生(0388-1512)51巻	原著論文	*育児; *労	労働時間	ヒト; 成人		母性衛生(0388-1512)51巻4号 Page704-710(2011.01)
2.01E+09	0	0	0	0	rk and bala	0	workstyle	workstyle	助産師の出	目的 本研	北川 良子(	日本助産学会誌(0917-635	原著論文/	*育児; 意		ヒト; 成人	10.3418/jj	日本助産学会誌(0917-6357)24巻2号 Page345-357(2010.12)
2.01E+09	0	0	0	0	ed disease,	0	lifestyle	others	中高年女性	中高年女性	山中 近子(	予防医学(0388-1296)52号	原著論文/	Cholester		ヒト; 成人		予防医学(0388-1296)52号 Page101-105(2010.12)
2.01E+09	0	0	0	0	ed disease,	1	lifestyle a	fatigue	主介護者の	在宅で要介	鈴木 洋子(	栄養学雑誌(0021-5147)67	原著論文/	質問紙法;	*介護負担	ヒト; 成人	10.5264/e	栄養学雑誌(0021-5147)67巻4号 Page168-177(2009.08)
2.01E+09	0	0	0	0	lental healt	4	stress management		中高年女性	本研究は、	宮内 清子(	母性衛生(0388-1512)50巻	原著論文/	質問紙法;	自己評価	ヒト; 成人		母性衛生(0388-1512)50巻4号 Page646-655(2010.01)
2.01E+09	0	0	0	0	rk and bala	0	WFC	child-rear	育児中の	育児をし	中井 芙美	看護学統合研究(1346-069	原著論文	質問紙法;		ヒト; 成人		看護学統合研究(1346-0692)12巻1号 Page24-41(2010.10)
2.01E+09	0	0	0	0	lental healt	1	stress ma	social cap	職場のう	仕事ストレ	辻田 敏(日	日本未病システム学会雑誌	原著論文	質問紙法;		ヒト; 男; 女		日本未病システム学会雑誌(1347-5541)15巻2号 Page376-378(2010.03)
2.01E+09	0	0	0	0	lental healt	0	occupational stress		働く人々の	ソフトウェ	北岡 和代(	日本未病システム学会雑誌	原著論文/	質問紙法;	労働時間	ヒト; 成人		日本未病システム学会雑誌(1347-5541)15巻2号 Page398-401(2010.03)
2.01E+09	0	0	0	0	k environm	3	retention	turnover	女性看護師	本研究では	荒川 千秋(	日本看護研究学会雑誌(028	原著論文/	*看護師; コ	労働時間	ヒト; 成人	10.15065/	日本看護研究学会雑誌(0285-9262)34巻1号 Page85-92(2011.04)
2.01E+09	0	0	0	0	k environm	0	retention	turnover	リハビリテ	鹿児島大学	根路銘 周	The Japanese Journal of R	原著論文	質問紙法;	労働時間	ヒト; 女		The Japanese Journal of Rehabilitation Medicine(1881-3526)48巻5号 Page331-337(2011.05)
2.01E+09	0	0	0	0	related dis	0	comfortab	heat	ゴルフ場	【目的】夏	井奈波 良	日本職業・災害医学会会誌	原著論文/	季節; *筋	夏季; 更衣	ヒト; 成人		日本職業・災害医学会会誌(1345-2592)59巻2号 Page63-68(2011.03)
2.01E+09	0	0	0	0	ed disease,	1	lifestyle	alcohol	地方公務員	目的:本研	高松 詩織(	産業衛生学雑誌(1341-072	原著論文/	質問紙法;		ヒト; 成人	10.1539/s	産業衛生学雑誌(1341-0725)52巻1号 Page1-11(2010.01)
2.01E+09	0	0	0	0	lental healt	1	occupatio	predictor	労働者の抑	北海道・東	檀上 和真(	体力・栄養・免疫学雑誌(1	原著論文/	回帰分析;	自己評価	ヒト; 青年		体力・栄養・免疫学雑誌(1341-0865)19巻2号 Page94-96(2009.11)
2.01E+09	0	0	0	0	k environm	1	retention	turnover	病棟に勤務	目的 マグ	緒方 泰子(	日本公衆衛生雑誌(0546-17	原著論文	*医療関係	*看護実践	ヒト; 成人	10.11236/	日本公衆衛生雑誌(0546-1766)58巻6号 Page409-419(2011.06)
2.01E+09	0	0	0	0	n/ satisfac	1	career	就労格差	女性医師の	目的 わが	野村 恭子(	日本公衆衛生雑誌(0546-17	原著論文/	医師-患者		ヒト; 成人	10.11236/	日本公衆衛生雑誌(0546-1766)58巻6号 Page433-445(2011.06)
2.01E+09	0	0	0	0	rk and bala	6	WFC	caregiver	中年期就労	中年期の家	越智 若菜(	日本地域看護学会誌(1346-	原著論文	看護師; *在	*介護負担	ヒト; 成人	10.20746/	日本地域看護学会誌(1346-9657)13巻2号 Page140-145(2011.03)
2.01E+09	0	0	0	0	lental healt	1	occupatio	biomarker	事務系企業	事務系企業	入江 正洋(	健康科学(0387-7175)33巻	原著論文/	質問紙法;		ヒト; 成人		健康科学(0387-7175)33巻 Page33-38(2011.03)
2.01E+09	0	0	0	0	n/ satisfac	0	career	医療従事者	女性総合内	日本内科学	小崎 真規	日本内科学会雑誌(0021-53	原著論文/	質問紙法;		ヒト; 男; 女	10.2169/n	日本内科学会雑誌(0021-5384)100巻7号 Page2020-2031(2011.07)
2.01E+09	0	0	0	0	related dis	1	comfortab	noice	騒音職場勤	目的 喫煙	高田 康光(	厚生の指標(0452-6104)58	原著論文/	回帰分析;		ヒト; 成人		厚生の指標(0452-6104)58巻8号 Page30-33(2011.08)
2.01E+09	0	0	0	0	n/ satisfac	1	satisfaction		成人中・後	サクセスフ	丹下 智香	日本未病システム学会雑誌	原著論文/	*幸福; 個人	自己評価	ヒト; 成人		日本未病システム学会雑誌(1347-5541)16巻2号 Page345-348(2011.03)
2.01E+09	0	0	0	0	k environm	1	engageme	comittmen	地域在住中	職種と仕事	森山 雅子(	日本未病システム学会雑誌	原著論文/	個人的満足	自己評価	ヒト; 成人		日本未病システム学会雑誌(1347-5541)16巻2号 Page349-351(2011.03)
2.01E+09	0	0	0	0	n/ satisfac	1	satisfaction		派遣社員の	本研究では	田上 明日	カウンセリング研究(0914-	原著論文	質問紙法;		ヒト; 成人	10.11544/	カウンセリング研究(0914-8337)43巻2号 Page141-149(2010.06)
2.01E+09	0	0	0	0	ed disease,	0	diet and n	bone	企業で実施	企業内で行	曽我部 夏	日本食育学会誌(1882-477	原著論文/	光子吸収分	定量的超音	ヒト; 成人	10.14986/	日本食育学会誌(1882-4773)5巻4号 Page203-207(2011.10)
2.01E+09	0	0	0	0	related dis	1	injury	injury	職業性スト	仕事のスト	原谷 隆史(	労働安全衛生総合研究所特	原著論文/	葛藤; 怒り	家庭環境;	ヒト; 成人		労働安全衛生総合研究所特別研究報告(1882-8329)40号 Page169-173(2010.12)
2.01E+09	0	0	0	0	n/ satisfac	1	education	professinc	特別養護老	特別養護老	増原 真砂	自立支援介護学(2185-355	原著論文/	質問紙法;		ヒト; 成人		自立支援介護学(2185-355X)4巻2号 Page174-180(2011.04)
2.01E+09	0	0	0	0	lental healt	0	occupational stress		介護支援専	介護支援専	細羽 竜也(	人間と科学: 県立広島大学	原著論文/	質問紙法;		ヒト; 成人		人間と科学: 県立広島大学保健福祉学部誌(1346-3217)11巻1号 Page41-52(2011.03)
2.01E+09	0	0	0	0	egnancy ar	0	balance w	balance w	結婚・出産	女性医師に	野村 恭子(	医学教育(0386-9644)42巻	原著論文/	*結婚; *職		ヒト; 成人	10.11307/	医学教育(0386-9644)42巻4号 Page209-215(2011.08)
2.01E+09	0	0	0	0	rk and bala	0	WFC	child-rear	埼玉県内の	埼玉県理学	荒木 智子(	理学療法-臨床・研究・教育	原著論文	質問紙法;	労働時間	ヒト; 成人	10.11350/	理学療法-臨床・研究・教育(1880-893X)18巻 Page15-21(2011.03)
2.01E+09	0	0	0	0	ed disease,	0	lifestyle	smoke	人間ドック	目的:学校	谷口 治子(	人間ドック(1880-1021)26	原著論文/	質問紙法;		ヒト; 成人	10.11320/	人間ドック(1880-1021)26巻4号 Page607-614(2011.12)
2.01E+09	0	0	0	0	egnancy ar	6	fertility		不妊治療中	本研究では	林谷 啓美(	母性衛生(0388-1512)52巻	原著論文	労働衛生;		ヒト; 成人		母性衛生(0388-1512)52巻4号 Page492-499(2012.01)
2.01E+09	0	0	0	0	rk and bala	1	WFC	WFC	ワーク・フ	働く女性	山田 泰行(	産業保健人間工学研究(134	原著論文/	質問紙法;	家事	ヒト; 成人		産業保健人間工学研究(1345-1731)12巻増補 Page48-51(2010.10)
2.01E+09	0	0	0	0	nt and disc	6	discrimina	gender	女性勤労者	女性勤労者	柴田 恵子(	日本ウーマンズヘルス学会	原著論文	質問紙法;		ヒト; 成人		日本ウーマンズヘルス学会誌(1347-5894)10巻1号 Page43-50(2011.10)
2.01E+09	0	0	0	0	rk and bala	0	workstyle	workstyle	小児保健分	小児保健分	三善 陽子(	小児保健研究(0037-4113)	原著論文	質問紙法;	家庭環境;	ヒト; 成人		小児保健研究(0037-4113)71巻1号 Page60-66(2012.01)
2.01E+09	0	0	0	0	ed disease,	4	diet and n	diet and n	写真付き簡	目的:就労	海老原 泰	日本健康教育学会誌(1340-	原著論文/	エネルギー	空腹時血糖	ヒト; 成人	10.11260/	日本健康教育学会誌(1340-2560)20巻1号 Page51-59(2012.02)
2.01E+09	0	0	0	0	nt and disc	1	bullying	いじめ	介護老人福	目的:本研	谷口 敏代(	産業衛生学雑誌(1341-072	原著論文/	*労働衛生	実態調査	ヒト; 青年	10.1539/s	産業衛生学雑誌(1341-0725)54巻1号 Page1-9(2012.01)
2.01E+09	0	0	0	0	related dis	1	shift	shift	介護老人保	昼間と夜間	多田 由紀(	日本循環器病予防学会誌(1	原著論文/	栄養評価;		ヒト; 成人		日本循環器病予防学会誌(1346-6267)47巻1号 Page1-12(2012.01)
2.01E+09	0	0	0	0	rk and bala	0	workstyle	workstyle	2011年病院		日本看護協	日本看護協会調査研究報告	原著論文	精神保健;	実態調査;	ヒト; 成人		日本看護協会調査研究報告(0911-0844)85号 Page1-116(2012.03)
2.01E+09	0	0	0	0	n/ satisfac	0	career		時間的展望	目的 時間	中垣 明美(	日本看護医療学会雑誌(134	原著論文/	質問紙法;		ヒト; 成人	10.11477/	日本看護医療学会雑誌(1345-2606)13巻1号 Page1-10(2011.06)
2.01E+09	0	0	0	0	rk and bala	6	WFC	WLB	仕事と育児	本研究は、	中井 芙美	看護学統合研究(1346-069	原著論文	*育児; 母;		ヒト; 中年		看護学統合研究(1346-0692)13巻2号 Page1-15(2012.03)
2.01E+09	0	0	0	0	egnancy ar	0	balance w	balance w	就労女性の	目的:女性	大原 賢了(	産業衛生学雑誌(1341-072	原著論文/	質問紙法;		ヒト; 妊娠	10.1539/s	産業衛生学雑誌(1341-0725)54巻2号 Page61-70(2012.03)
2.01E+09	0	0	0	0	lental healt	1	occupatio	personal c	病院看護師	【目的】看	小野 桂子(	日本職業・災害医学会会誌	原著論文/	*A型人格;		ヒト; 女; 看		日本職業・災害医学会会誌(1345-2592)59巻1号 Page1-7(2011.01)
2.01E+09	0	0	0	0	opause, an	0	menopaus	更年期	地方公務員	経済危機も	森脇 智秋(	インターナショナルNursin	原著論文/	質問紙法;		ヒト; 成人		インターナショナルNursing Care Research(1347-1341)11巻2号 Page21-30(2012.05)
2.01E+09	0	0	0	0	rk and bala	0	WFC	WFC	訪問看護師	本研究では	山口 善子(	日本看護福祉学会誌(1344-	原著論文/	看護師; *労		ヒト; 成人		日本看護福祉学会誌(1344-4875)17巻2号 Page27-37(2012.03)
2.01E+09	0	0	0	0	lental healt	0	occupational stress		北海道・東	北海道・東	三宅 浩次(	北海道公衆衛生学雑誌(091	原著論文	質問紙法;	自己評価	ヒト; 成人		北海道公衆衛生学雑誌(0914-2630)25巻2号 Page175-178(2012.03)
2.01E+09	0	0	0	0	lental healt	1	occupational stress		中・高等学	本研究は、	堀 匡(東北	ストレス科学(1349-4813)	原著論文	質問紙法;		ヒト; 成人		ストレス科学(1349-4813)25巻3号 Page221-229(2010.12)
2.01E+09	0	0	0	0	egnancy ar	1	BF	BF	弘前市内保	厚生労働省	福士 真都	健生病院医報(0289-3304)	原著論文	質問紙法;	弘前市	ヒト; 成人		健生病院医報(0289-3304)35巻 Page41-43(2012.03)
2.01E+09	0	0	0	0	k environm	1	engageme	scale deve	Work Limi	目的:出勤	井田 浩正(	産業衛生学雑誌(1341-072	原著論文	質問紙法;	集中維持機	ヒト; 成人	10.1539/s	産業衛生学雑誌(1341-0725)54巻3号 Page101-107(2012.05)
2.01E+09	0	0	0	0	k environm	6	retention	turnover	子育ての支	本研究の目	岩下 真由	日本看護管理学会誌(1347-	原著論文	*育児; *看		ヒト; 成人	10.19012/	日本看護管理学会誌(1347-0140)16巻1号 Page45-56(2012.07)
2.01E+09	0	0	0	0	ed disease,	1	lifestyle	others	労働者にお	本研究は、	神崎 匠世(	日本農村医学会雑誌(0468-	原著論文/	*行動療法	保健指導	ヒト; 成人	10.2185/jj	日本農村医学会雑誌(0468-2513)61巻1号 Page55-66(2012.05)
2.01E+09	0	0	0	0	related dis	1	shift	shift	夜間労働時	夜間労働時	宮内 文久(	産業ストレス研究(1340-77	原著論文/	サーカディ		ヒト; 成人		産業ストレス研究(1340-7724)19巻3号 Page249-254(2012.07)
2.01E+09	0	0	0	0	related dis	0	Musle ske	MusleSke	職種別にみ	非特異的腰	砂川 隆英(	日本職業・災害医学会会誌	原著論文/	*X線診断;		ヒト; 男; 女		日本職業・災害医学会会誌(1345-2592)60巻4号 Page231-234(2012.07)
2.01E+09	0	0	0	0	ed disease,	1	diet and n	diet and n	日本語版D	本研究では	高山 直子(	日本健康医学会雑誌(1343-	原著論文/	質問紙法;		ヒト; 青年	10.20685/	日本健康医学会雑誌(1343-0025)21巻2号 Page87-94(2012.07)
2.01E+09	0	0	0	0	n/ satisfac	3	satisfaction		中高年日本	本研究の目	柴田 博(人	応用老年学(1882-6245)6巻	原著論文	因子分析;	家事; 心身	ヒト; 中年		応用老年学(1882-6245)6巻1号 Page21-38(2012.07)
2.01E+09	0	0	0	0	ed disease,	0	lifestyle	others	平成5年と	本研究は、	谷口 いつ	総合健診(1347-0086)39巻	原著論文/	*血圧; 血糖	空腹時血糖	ヒト; 成人	10.7143/jh	総合健診(1347-0086)39巻5号 Page567-574(2012.09)
2.01E+09	0	0	0	0	rk and bala	1	WFC	WFC	労働者のワ	仕事・家庭	鈴木 淳平(	産業精神保健(1340-2862)	原著論文/	*育児; *労		ヒト; 成人		産業精神保健(1340-2862)20巻3号 Page237-249(2012.09)
2.01E+09	0	0	0	0	k environm	0	retention	turnover	女性看護師	総務省「就	宮崎 悟(同	日本医療・病院管理学会誌	原著論文/	質問紙法;	労働時間	ヒト; 青年	10.11303/	日本医療・病院管理学会誌(1882-594X)49巻3号 Page147-157(2012.07)
2.01E+09	0	0	0	0	egnancy ar	0	safety, wo	safety, wo	理学療法士	著者らは理	荒木 智子(	理学療法-臨床・研究・教育	原著論文	質問紙法;		ヒト; 妊娠	10.11350/	理学療法-臨床・研究・教育(1880-893X)19巻1号 Page49-53(2012.03)
2.01E+09	0	0	0	0	rk and bala	0	WFC	WLB	医師におけ	1)医師にお	安川 康介(	医学教育(0386-9644)43巻	原著論文/	育児; *医師	*家事; *労	ヒト; 成人	10.11307/	医学教育(0386-9644)43巻4号 Page315-319(2012.08)
2.01E+09	0	0	0	0	lental healt	1	occupational stress		地域包括支	地域包括支	村山 洋史(	日本地域看護学会誌(1346-	原著論文	コミュニケ	コミュニテ	ヒト; 成人	10.20746/	日本地域看護学会誌(1346-9657)13巻2号 Page125-132(2011.03)
2.01E+09	0	0	0	0	rk and bala	6	WFC	caregiver	疾患をもつ	介護保険制	松本 明美(	ヘルスサイエンス研究(134	原著論文	*在宅介護	*介護負担	ヒト; 中年		ヘルスサイエンス研究(1343-3393)16巻1号 Page31-36(2012.10)
2.01E+09	0	0	0	0	rk and bala	3	WFC	child-rear	未就学児を	目的 従来	島田 恭子(	厚生の指標(0452-6104)59	原著論文	*育児; 回帰		ヒト; 成人		厚生の指標(0452-6104)59巻15号 Page10-18(2012.12)
2.01E+09	0	0	0	0	related dis	0	shift	shift	夜間勤務、	夜間勤務で	矢倉 宏祐(	日本未病システム学会雑誌	原著論文/	下痢; 口内	*舌診	ヒト; 成人		日本未病システム学会雑誌(1347-5541)18巻2号 Page102-106(2012.06)
2.01E+09	0	0	0	0	egnancy ar	0	work, nutr	work, nutr	妊娠前の2	Body Mas	美甘 祥子(	母性衛生(0388-1512)53巻	原著論文/	*食行動; *	価値観; 実	ヒト; 成人		母性衛生(0388-1512)53巻4号 Page522-529(2013.01)
2.01E+09	0	0	0	0	lental healt	1	stress ma	resillience	20代女性労	本研究は2	大谷 喜美	産業精神保健(1340-2862)	原著論文/	質問紙法;	GHQ (精神	ヒト; 成人		産業精神保健(1340-2862)19巻3号 Page188-203(2011.09)
2.01E+09	0	0	0	0	lental healt	0	occupatio	sleep	睡眠と覚醒	[目的]訪問	原野 かお	介護福祉学(1340-8178)18	原著論文/	*労働衛生	眠気(病因,	ヒト; 成人		介護福祉学(1340-8178)18巻2号 Page145-154(2011.10)
2.01E+09	0	0	0	0	rk and bala	0	rework	Rework	事業場と医	事業場と医	五十嵐 良	産業医学ジャーナル(0388-	原著論文/	質問紙法;		ヒト; 成人		産業医学ジャーナル(0388-337X)36巻1号 Page73-79(2013.01)
2.01E+09	0	0	0	0	lental healt	0	occupational stress		医療ソーシ	医療ソーシ	山川 敏久(	医療と福祉(0287-1521)45	原著論文	質問紙法;	*医療ソー	ヒト; 成人		医療と福祉(0287-1521)45巻1号 Page34-41(2011.11)
2.01E+09	0	0	0	0	rk and bala	0	WFC	WFC	47都道府県		熊切 こず	日本臨床工学技士会会誌(1	原著論文	質問紙法;	家庭環境	ヒト; 女		日本臨床工学技士会会誌(1341-3171)44号 Page12-16(2012.03)
2.01E+09	0	0	0	0	lental healt	0	occupational stress		児童養護施	児童養護施	竹野 夏美(	産業ストレス研究(1340-77	原著論文	質問紙法;	GHQ (精神	ヒト; 成人		産業ストレス研究(1340-7724)19巻2号 Page155-165(2012.04)
2.01E+09	0	0	0	0	rk and bala	1	WFC	child-rear	未就学児の	目的:仕事	丸山 昭子(	日本看護科学会誌(0287-53	原著論文/	育児; 看護	労働時間	ヒト; 成人	10.5630/ja	日本看護科学会誌(0287-5330)32巻2号 Page44-53(2012.06)
2.01E+09	0	0	0	0	lental healt	1	occupational stress		介護業務に	目的 本研	立花 直樹(	厚生の指標(0452-6104)59	原著論文/	質問紙法;		ヒト; 成人		厚生の指標(0452-6104)59巻8号 Page29-37(2012.08)
2.01E+09	0	0	0	0	related dis	1	Musle ske	MusleSke	女性家族介	目的:女性	鈴木 岸子(	日本看護医療学会雑誌(134	原著論文/	高血圧; 身	介護負担	ヒト; 中年	10.11477/	日本看護医療学会雑誌(1345-2606)14巻2号 Page13-22(2012.12)
2.01E+09	0	0	0	0	related dis	0	shift	shift	女性看護師	愛媛労災病	宮内 文久(	日本職業・災害医学会会誌	原著論文/	サーカディ		ヒト; 成人		日本職業・災害医学会会誌(1345-2592)60巻6号 Page348-352(2012.11)
2.01E+09	0	0	0	0	nt and disc	6	violence	患者からの	暴力被害に	[研究目的]	青木 洋明(	日本精神科看護学術集会誌	原著論文	*精神看護		ヒト; 男; 女		日本精神科看護学術集会誌55巻2号 Page167-171(2012.08)
2.01E+09	0	0	0	0	lental healt	4	stress management		勤労女性の	勤労女性の	加藤 由紀	保健の科学(0018-3342)54	原著論文	看護師; 労	GHQ (精神	ヒト; 成人		保健の科学(0018-3342)54巻11号 Page783-789(2012.11)
2.01E+09	0	0	0	0	opause, an	1	menstrati	月経	女性看護職	本研究の目	大滝 千智(	日本母性看護学会誌(1345-	原著論文/	*月経; *月	価値観	ヒト; 成人		日本母性看護学会誌(1345-773X)13巻1号 Page1-8(2013.03)
2.01E+09	0	0	0	0	opause, an	0	Breast_ca	乳がん	日本のすみ	日本での子	坂 佳奈子(	日本乳癌検診学会誌(0918-	原著論文	*集団検診	受療行動	ヒト; 成人	10.3804/jj	日本乳癌検診学会誌(0918-0729)22巻1号 Page31-36(2013.03)
2.01E+09	0	0	0	0	lental healt	1	occupational stress		社会格差と	本研究では	島津 明人(	ストレス科学(1349-4813)	原著論文	質問紙法;		ヒト; 成人		ストレス科学(1349-4813)27巻1号 Page60-66(2012.07)
2.01E+09	0	0	0	0	egnancy ar	3	mental he	mental he	社会格差と	雇用状態,	三宅 吉博(	ストレス科学(1349-4813)	原著論文/	質問紙法;	自己評価	ヒト; 成人		ストレス科学(1349-4813)27巻1号 Page75-80(2012.07)
2.01E+09	0	0	0	0	opause, an	1	menopaus	QOL低下	更年期女性	【目的】2	須賀 万智(	日本女性医学学会雑誌(218	原著論文/	質問紙法;		ヒト; 中年		日本女性医学学会雑誌(2185-8861)20巻3号 Page391-398(2013.04)
2.01E+09	0	0	0	0	egnancy ar	1	fertility		不妊治療の	目的:不妊	鶴巻 陽子(	母性衛生(0388-1512)54巻	原著論文/	人工授精;	SF-36	ヒト; 成人		母性衛生(0388-1512)54巻1号 Page78-85(2013.04)
2.01E+09	0	0	0	0	related dis	1	shift	shift	アクティグ	夜勤勤務を	野原 理子(	日本ウーマンズヘルス学会	原著論文	育児; 労働		ヒト; 成人		日本ウーマンズヘルス学会誌(1347-5894)11巻1号 Page67-71(2012.10)
2.01E+09	0	0	0	0	opause, an	1	Uterine_ca	子宮がん	20歳代勤労	本研究の目	中越 利佳(	母性衛生(0388-1512)54巻	原著論文/	質問紙法;	*受療行動	ヒト; 成人		母性衛生(0388-1512)54巻1号 Page164-172(2013.04)
2.01E+09	0	0	0	0	lental healt	1	stress ma	resillienac	看護師のレ	看護師のレ	清野 純子(	日本ウーマンズヘルス学会	原著論文	質問紙法;		ヒト; 成人		日本ウーマンズヘルス学会誌(1347-5894)11巻1号 Page127-134(2012.10)
2.01E+09	0	0	0	0	rk and bala	0	WFC	WFC	中壮年期に	【目的】本	熊谷 たま	医療看護研究(1349-8630)	原著論文/	*葛藤; 回帰	労働時間	ヒト; 成人		医療看護研究(1349-8630)9巻2号 Page36-44(2013.03)
2.01E+09	0	0	0	0	opause, an	0	hormone t	ホルモン	「ホルモン	1985年の	峯村 昌子(	更年期と加齢のヘルスケア	原著論文	*エストロ		ヒト; 女		更年期と加齢のヘルスケア(1347-801X)12巻1号 Page122-127(2013.06)
2.01E+09	0	0	0	0	lental healt	1	occupatio	SOC	看護師がス	看護師がス	高橋 ゆか	ヘルスサイエンス研究(134	原著論文	質問紙法;		ヒト; 成人		ヘルスサイエンス研究(1343-3393)16巻1号 Page53-56(2012.10)
2.01E+09	0	0	0	0	rk and bala	1	WFC	child-rear	乳幼児を持	目的 育児	池田 隆英(	厚生の指標(0452-6104)60	原著論文	質問紙法;	母集団	ヒト; 成人		厚生の指標(0452-6104)60巻3号 Page9-17(2013.03)
2.01E+09	0	0	0	0	n/ satisfac	0	satisfaction		ワークライ	本研究の目	渡邊 郁子(	日本看護管理学会誌(1347-	原著論文/	看護師; *労	自己実現;	ヒト; 成人	10.19012/	日本看護管理学会誌(1347-0140)17巻1号 Page37-47(2013.06)
2.01E+09	0	0	0	0	rk and bala	0	WFC	caregiver	高齢者ケア	本稿の目的	山口 麻衣(	老年社会科学(0388-2446)	原著論文	*在宅介護	労働時間	ヒト; 中年		老年社会科学(0388-2446)26巻1号 Page58-67(2004.04)
2.01E+09	0	0	0	0	related dis	1	shift	shift	生活活動と	疲労の回復	久保 智英(	産業衛生学雑誌(1341-072	原著論文	因子分析;	外出; 労働	ヒト; 成人	10.1539/s	産業衛生学雑誌(1341-0725)55巻3号 Page90-102(2013.05)
2.01E+09	0	0	0	0	lental healt	1	occupatio	lifestyle a	勤労者のメ	西日本を拠	本田 貴紀(	健康科学(0387-7175)35巻	原著論文/	質問紙法;		ヒト; 成人		健康科学(0387-7175)35巻 Page33-43(2013.03)
2.01E+09	0	0	0	0	rk and bala	1	WFC	child-rear	首都圏で幼	就業の有無	岩崎 りほ(	小児保健研究(0037-4113)	原著論文/	*育児; 回帰	東京都23区	ヒト; 成人		小児保健研究(0037-4113)72巻3号 Page377-385(2013.05)
2.01E+09	0	0	0	0	lental healt	0	occupatio	fatigue	精神科看護	【はじめに	石井 拓哉(	日本精神科看護学術集会誌	原著論文/	*精神看護		ヒト; 成人		日本精神科看護学術集会誌55巻2号 Page351-354(2012.08)
2.01E+09	0	0	0	0	related dis	1	Musle ske	MusleSke	小規模事業	A社会福祉	冨岡 公子(	産業医学ジャーナル(0388-	原著論文	質問紙法;		ヒト; 成人		産業医学ジャーナル(0388-337X)36巻4号 Page45-51(2013.07)
2.01E+09	0	0	0	0	egnancy ar	6	balance w	balance w	キャリア追	本研究は、	天野 道代(	母性衛生(0388-1512)54巻	原著論文	経産回数;		ヒト; 妊娠		母性衛生(0388-1512)54巻2号 Page354-365(2013.07)
2.01E+09	0	0	0	0	egnancy ar	0	fertility		山口県にお	山口県産婦	佐世 正勝(	産婦人科の実際(0558-472	原著論文/	質問紙法;	実態調査;	ヒト; 妊娠		産婦人科の実際(0558-4728)62巻8号 Page1133-1139(2013.08)
2.01E+09	0	0	0	0	lental healt	1	occupatio	SOC	ソフトウェ	日本のコン	守田 祐作(	産業ストレス研究(1340-77	原著論文	回帰分析;		ヒト; 成人		産業ストレス研究(1340-7724)20巻2号 Page163-168(2013.04)
2.01E+09	0	0	0	0	lental healt	1	occupatio	predictor	20歳代およ	近年、職場	濱西 誠司(	ヒューマンケア研究学会誌	原著論文/	家族特性;	労働時間	ヒト; 成人		ヒューマンケア研究学会誌(2187-2813)4巻2号 Page31-36(2013.03)
2.01E+09	0	0	0	0	lental healt	1	occupatio	sleep	ストレスと	ストレスと	佐藤 勝昌(	医学と生物学(0019-1604)	原著論文/	飲酒; 会話	眠気; 実態	ヒト; 成人		医学と生物学(0019-1604)157巻5号 Page708-712(2013.05)
2.01E+09	0	0	0	0	ed disease,	1	LRD	HTN	都内勤務者	目的 高血	田島 美紀(	厚生の指標(0452-6104)60	原著論文/	質問紙法;		ヒト; 成人		厚生の指標(0452-6104)60巻8号 Page1-8(2013.08)
2.01E+09	0	0	0	0	lental healt	1	occupatio	SOC	職業性スト	某食品製造	新谷 奈苗(	産業保健人間工学研究(134	原著論文	質問紙法;		ヒト		産業保健人間工学研究(1345-1731)14巻増補 Page33-36(2012.10)
2.01E+09	0	0	0	0	rk and bala	1	WFC	WFC	就労女性に	就労女性の	星野 藍子(	作業療法(0289-4920)32巻	原著論文	質問紙法;	*家事; 自己	ヒト; 成人	10.11477/	作業療法(0289-4920)32巻4号 Page335-344(2013.08)
2.01E+09	0	0	0	0	related dis	0	vibration	others	立位・座位	従来,長時間	柴田 延幸(	労働安全衛生総合研究所特	原著論文/	加速度; *振	*不快症状	ヒト; 成人		労働安全衛生総合研究所特別研究報告(1882-8329)42号 Page205-211(2012.11)
2.01E+09	0	0	0	0	rk and bala	0	WFC	caregiver	家族介護者	ショートス	立松 麻衣	日本家政学会誌(0913-522	原著論文	在宅介護;	*介護負担	ヒト; 成人	10.11428/	日本家政学会誌(0913-5227)64巻9号 Page577-590(2013.09)
2.01E+09	0	0	0	0	lental healt	1	occupatio	predictor	中小規模事	中小規模事	足立 安正(	日本地域看護学会誌(1346-	原著論文/	家族特性;	自己評価	ヒト; 成人	10.20746/	日本地域看護学会誌(1346-9657)16巻1号 Page48-55(2013.07)
2.01E+09	0	0	0	0	lental healt	1	occupatio	biomarker	唾液中コル	比較的短時	加納 亜紀(	CAMPUS HEALTH(1341-4	原著論文/	*Hydrocor	感情プロフ	ヒト; 成人		CAMPUS HEALTH(1341-4313)50巻1号 Page289-291(2013.03)
2.01E+09	0	0	0	0	rk and bala	0	rework	rework	復帰支援に	【背景・方	井手野 由	医学教育(0386-9644)44巻	原著論文	医師; 不安		ヒト; 成人	10.11307/	医学教育(0386-9644)44巻4号 Page237-242(2013.08)
2.01E+09	0	0	0	0	lental healt	0	occupational stress		大学医学部	本学(大学)	尾崎 岩太(	CAMPUS HEALTH(1341-4	原著論文	質問紙法;	実態調査	ヒト; 男; 女		CAMPUS HEALTH(1341-4313)50巻1号 Page574-576(2013.03)
2.01E+09	0	0	0	0	rk and bala	0	workstyle	workstyle	歯学部・歯	日本の歯学	村上 多恵	口腔衛生学会雑誌(0023-28	原著論文/	質問紙法;		ヒト; 男; 女	10.5834/jo	口腔衛生学会雑誌(0023-2831)63巻5号 Page444-452(2013.10)
2.01E+09	0	0	0	0	lental healt	0	stress ma	counseling	【地域小規	背景と目的	池野 多美	ストレス科学(1349-4813)	原著論文/	*一次予防		ヒト; 成人		ストレス科学(1349-4813)28巻1号 Page17-22(2013.06)
2.01E+09	0	0	0	0	rk and bala	1	WFC	child-rear	保育園児の	保育園児の	須藤 茉衣	民族衛生(0368-9395)79巻	原著論文	*育児; 家族		ヒト; 成人	10.3861/js	民族衛生(0368-9395)79巻6号 Page149-158(2013.11)
2.01E+09	0	0	0	0	lental healt	1	occupational stress		開業歯科医	開業歯科医	佐々 千晶(	日本歯科医療管理学会雑誌	原著論文/	月経異常;	自己評価	ヒト; 女; 歯		日本歯科医療管理学会雑誌(0387-5687)48巻2号 Page155-164(2013.08)
2.01E+09	0	0	0	0	ed disease,	1	lifestyle	alcohol	勤労女性に	目的:効果	足達 淑子(	臨床栄養(0485-1412)124巻	原著論文/	アルコール	労働時間	ヒト; 成人		臨床栄養(0485-1412)124巻2号 Page243-248(2014.02)
2.01E+09	0	0	0	0	nt and disc	1	bullying	いじめ	先輩看護師	本研究は、	大鳥 和子(	心身健康科学(1882-6881)	原著論文/	*看護師; *	心身状態	ヒト; 成人	10.11427/	心身健康科学(1882-6881)10巻1号 Page33-42(2014.02)
2.01E+09	0	0	0	0	lental healt	0	occupational stress		労働者にお	13事業所の	女屋 千代	予防医学ジャーナル(0285-	原著論文/	*労働衛生	労働時間	ヒト; 成人		予防医学ジャーナル(0285-0877)471号 Page84-86(2013.07)

2.01E+09	0	0	0	0	n/ satisfac	1	education	health prc	中小企業	中小企業	荒巻 悦子(	医療福祉経営マーケティン	原著論文/	*労働衛生		ヒト; 成人		医療福祉経営マーケティング研究(1881-297X)8巻1号 Page11-17(2013.10)
2.01E+09	0	0	0	0	lental healt	1	occupatio	sleep	規則性・	目的:睡眠	松本 悠貴(	産業衛生学雑誌(1341-072	原著論文	質問紙法;	標準化	ヒト; 青年	10.1539/s	産業衛生学雑誌(1341-0725)55巻5号 Page154-164(2013.09)
2.01E+09	0	0	0	0	rk and bala	1	workstyle	workstyle	女性理学	本研究は、	山野 薫(宝	理学療法兵庫(1341-3910)	原著論文	質問紙法;		ヒト; 成人		理学療法兵庫(1341-3910)19号 Page26-30(2014.01)
2.01E+09	0	0	0	0	n/ satisfac	1	satisfaction		筑波研究	目的 研究	平井 康仁(	厚生の指標(0452-6104)61	原著論文/	労働衛生;	実態調査;	ヒト; 成人		厚生の指標(0452-6104)61巻2号 Page22-28(2014.02)
2.01E+09	0	0	0	0	related dis	4	Musle ske	MusleSke	勤労者に	職種の違い	高野 賢一	日本職業・災害医学会会誌	原著論文/	労作; 眼精	実態調査	ヒト; 成人		日本職業・災害医学会会誌(1345-2592)62巻1号 Page32-37(2014.01)
2.01E+09	0	0	0	0	rk and bala	1	rework	Rework	リワークに	デイケアで	堀 弘子(あ	病院・地域精神医学(0910-	原著論文	うつ病(リ		ヒト; 成人		病院・地域精神医学(0910-4798)56巻2号 Page94-97,99(2014.02)
2.01E+09	0	0	0	0	lental healt	3	occupatio	sleep	勤務時間	本研究では	久保 智英(	労働安全衛生総合研究所特	原著論文/	質問紙法;	*労働時間	ヒト; 成人		労働安全衛生総合研究所特別研究報告(1882-8329)43号 Page137-141(2013.11)
2.01E+09	0	0	0	0	egnancy ar	1	mental he	mental he	妊婦のレ	【目的】	藤田 佳代	日本ウーマンズヘルス学会	原著論文/	*経産回数	GHQ (精神	ヒト; 妊娠		日本ウーマンズヘルス学会誌(1347-5894)12巻1号 Page1-10(2013.10)
2.01E+09	0	0	0	0	rk and bala	0	WFC	WFC	ワークファ		水野 千奈	日本ウーマンズヘルス学会	原著論文	*葛藤; スト		ヒト; 成人		日本ウーマンズヘルス学会誌(1347-5894)12巻1号 Page97-102(2013.10)
2.01E+09	0	0	0	0	rk and bala	1	WFC	child-rear	出産後の	目的 妊娠	松永 佳子(	日本母子看護学会誌(1882-	原著論文/	*育児; *人	*家事; 家庭	ヒト; 妊娠	10.11477/	日本母子看護学会誌(1882-2495)7巻2号 Page22-32(2014.02)
2.01E+09	0	0	0	0	lental healt	1	occupatio	SOC	看護師のS	目的:看護	吉田 えり(	日本看護研究学会雑誌(028	原著論文	回帰分析;		ヒト; 成人	10.15065/	日本看護研究学会雑誌(0285-9262)36巻5号 Page25-33(2013.12)
2.01E+09	0	0	0	0	related dis	4	VDT	VDT	低色温度	2009年に	杉野 友啓(	日本未病システム学会雑誌	原著論文/	色; *眼精	*VDT症候	ヒト; 成人		日本未病システム学会雑誌(1347-5541)19巻3号 Page9-18(2013.12)
2.01E+09	0	0	0	0	n/ satisfac	0	career	workstyle	今後の医	日本神経	深浦 彦彰(	臨床神経学(0009-918X)53	原著論文	質問紙法;		ヒト; 成人	10.5692/c	臨床神経学(0009-918X)53巻11号 Page1354-1357(2013.11)
2.01E+09	0	0	0	0	rk and bala	1	WFC	child-rear	乳幼児期	乳幼児期	富田 早苗(	小児保健研究(0037-4113)	原著論文/	*育児; 回		ヒト; 成人		小児保健研究(0037-4113)73巻2号 Page308-315(2014.03)
2.01E+09	0	0	0	0	lental healt	1	occupatio	predictor	就労うつ	就労うつ	星野 藍子(	作業療法(0289-4920)33巻	原著論文/	質問紙法;	*家事	ヒト; 成人	10.11477/	作業療法(0289-4920)33巻1号 Page42-52(2014.02)
2.01E+09	0	0	0	0	ed disease,	1	physical a	exercise	自己効力	本研究では	渡辺 和広(	行動医学研究(1341-6790)	原著論文	質問紙法;		ヒト	10.11331/	行動医学研究(1341-6790)20巻1号 Page17-23(2014.03)
2.01E+09	0	0	0	0	n/ satisfac	1	career	医療従事者	クリティカ	目的:クリ	中井 夏子(	日本臨床救急医学会雑誌(1	原著論文/	看護生涯	実態調査	ヒト; 成人	10.11240/	日本臨床救急医学会雑誌(1345-0581)16巻6号 Page831-838(2013.12)
2.01E+09	0	0	0	0	related dis	1	Musle ske	MusleSke	職務形態の	フルタイム	内間 康知(	日本職業・災害医学会会誌	原著論文/	質問紙法;	除脂肪体重	ヒト; 成人		日本職業・災害医学会会誌(1345-2592)62巻2号 Page96-100(2014.03)
2.01E+09	0	0	0	0	egnancy ar	1	pregnancy	pregnancy	早産に関	多施設共同	塩崎 有宏(	助産雑誌(1347-8168)68巻	原著論文/	危険因子;	労働時間	ヒト; 妊娠	10.11477/	助産雑誌(1347-8168)68巻5号 Page426-430(2014.05)
2.01E+09	0	0	0	0	opause, ar	1	menopaus	QOL低下	一般更年	不安や抑う	花田 富美	日本健康医学会雑誌(1343-	原著論文/	*更年期;	自己評価	ヒト; 成人	10.20685/	日本健康医学会雑誌(1343-0025)23巻1号 Page26-30(2014.04)
2.01E+09	0	0	0	0	n/ satisfac	1	self-effica	literacy	性成熟期	目的 ヘル	河田 志帆(	日本公衆衛生雑誌(0546-1	原著論文	因子分析;		ヒト; 成人	10.11236/	日本公衆衛生雑誌(0546-1766)61巻4号 Page186-196(2014.04)
2.01E+09	0	0	0	0	lental healt	1	occupatio	personal (	就労者の	就労者を対	太田 真貴(	認知療法研究(1883-2296)	原著論文	因子分析;	GHQ (精神	ヒト; 成人		認知療法研究(1883-2296)7巻1号 Page76-83(2014.02)
2.01E+09	0	0	0	0	rk environm	1	engagement		介護老人	介護職と看	緒形 明美(	日本看護医療学会雑誌(134	原著論文/	*葛藤; 専門	労働時間	ヒト; 成人	10.11477/	日本看護医療学会雑誌(1345-2606)15巻2号 Page23-39(2014.01)
2.01E+09	0	0	0	0	related dis	1	injury	injury	女性看護	目的:A県	相撲 佐希	医療の質・安全学会誌(18	原著論文/	コミュニケ	GHQ (精神	ヒト; 成人		医療の質・安全学会誌(1881-3658)9巻1号 Page5-11(2014.02)
2.01E+09	0	0	0	0	rk and bala	6	workstyle	workstyle	女性医師の	本研究は、	米本 倉基(	日本医療・病院管理学会誌	原著論文	育児; 性同		ヒト; 成人	10.11303/	日本医療・病院管理学会誌(1882-594X)51巻2号 Page117-125(2014.04)
2.01E+09	0	0	0	0	lental healt	1	occupatio	sleep	労働者の	【目的】	小山 文彦(	日本職業・災害医学会会誌	原著論文	バイオマー	LC-MS法;	ヒト; 成人		日本職業・災害医学会会誌(1345-2592)62巻3号 Page143-148(2014.05)
2.01E+09	0	0	0	0	rk and bala	1	WFC	child-rear	就労する	本研究では	酒井 厚(山	小児保健研究(0037-4113)	原著論文/	質問紙法;		ヒト; 成人		小児保健研究(0037-4113)73巻2号 Page316-323(2014.03)
2.01E+09	0	0	0	0	lental healt	1	occupational stress		労働者に	ホワイトカ	谷原 弘之(	産業ストレス研究(1340-7	原著論文/	質問紙法;	自己評価	ヒト; 成人		産業ストレス研究(1340-7724)21巻2号 Page201-206(2014.04)
2.01E+09	0	0	0	0	rk environm	1	productivi	presentee	企業社員に	ユーモア	段上 朋子(	民族衛生(0368-9395)80巻	原著論文	*ウイット		ヒト; 成人	10.3861/j	民族衛生(0368-9395)80巻3号 Page127-143(2014.05)
2.01E+09	0	0	0	0	ed disease,	1	physical a	exercise	運動習慣の	健康の獲得	河合 美香(	日本健康医学会雑誌(1343-	原著論文/	タバコ喫煙	興味	ヒト; 成人	10.20685/	日本健康医学会雑誌(1343-0025)23巻2号 Page80-96(2014.07)
2.01E+09	0	0	0	0	related dis	0	shift	shift	客観的・主	目的:日勤	松本 光寛(	産業衛生学雑誌(1341-072	原著論文/	質問紙法;		ヒト; 成人	10.1539/s	産業衛生学雑誌(1341-0725)56巻3号 Page67-73(2014.05)
2.02E+09	0	0	0	0	lental healt	0	occupational stress		IT企業に	IT企業の	西 基(北海	北方産業衛生(0911-3363)	原著論文/	うつ病(診	自己評価	ヒト; 成人		北方産業衛生(0911-3363)50号 Page2-6(2014.08)
2.02E+09	0	0	0	0	rk and bala	0	rework	Rework	メンタル	メンタル	杉本 洋子(	産業ストレス研究(1340-7	原著論文/	うつ病(リ		ヒト; 成人		産業ストレス研究(1340-7724)21巻3号 Page259-269(2014.07)
2.02E+09	0	0	0	0	rk and bala	0	WFC	caregiver	在宅認知	本研究の目	藤原 和彦(	日本作業療法研究学会雑誌	原著論文/	*在宅介護	*介護負担	ヒト; 成人		日本作業療法研究学会雑誌(1882-0948)17巻1号 Page31-40(2014.06)
2.01E+09	0	0	0	0	rk environm	0	productivity		女性看護	【目的】	井奈波 良	日本職業・災害医学会会誌	原著論文/	*看護師; *	労働時間	ヒト; 成人		日本職業・災害医学会会誌(1345-2592)62巻3号 Page173-178(2014.05)
2.02E+09	0	0	0	0	ed disease,	4	oral healtl	oral	職域にお	背景:健康	影山 淳(ヤ	産業衛生学雑誌(1341-072	原著論文/	教育評価;		ヒト; 成人	10.1539/s	産業衛生学雑誌(1341-0725)56巻5号 Page141-151(2014.09)
2.01E+09	0	0	0	0	lental healt	1	occupatio	sleep	労働者に	【背景】	中島 俊(東	ストレス科学(1349-4813)	原著論文	質問紙法;		ヒト; 成人		ストレス科学(1349-4813)28巻4号 Page275-285(2014.04)
2.02E+09	0	0	0	0	rk and bala	1	WFC	WFC	女性医師の	目的:女性	片岡 仁美(	医学教育(0386-9644)45巻	原著論文	*労働衛生	実態調査;	ヒト; 女	10.11307/	医学教育(0386-9644)45巻5号 Page365-375(2014.10)
2.02E+09	0	0	0	0	ed disease,	1	diet and n	diet and n	血液中酸化	血液中酸化	渡辺 洋子(	DNA多型(2188-3815)22巻	原著論文/	NADPH D	p22-phox;	ヒト; 成人		DNA多型(2188-3815)22巻1号 Page203-206(2014.07)
2.02E+09	0	0	0	0	rk environm	1	engagement		日本人労	本研究では	窪田 和巳(	行動医学研究(1341-6790)	原著論文	質問紙法;	構成概念	ヒト; 成人	10.11331/	行動医学研究(1341-6790)20巻2号 Page69-76(2014.10)
2.02E+09	0	0	0	0	rk and bala	0	WFC	caregiver	介護が就業	わが国では	岸田 研作(	医療経済研究(1340-895X)	原著論文	*在宅介護	介護負担;	ヒト; 成人		医療経済研究(1340-895X)26巻1号 Page43-58(2014.10)
2.01E+09	0	0	0	0	lental healt	1	occupational stress		生活保護	目的 生活	赤間 由美(	日本公衆衛生雑誌(0546-1	原著論文/	*ソーシャ	精神保健	ヒト; 成人	10.11236/	日本公衆衛生雑誌(0546-1766)61巻7号 Page342-353(2014.07)
2.02E+09	0	0	0	0	lental healt	1	occupatio	sleep	睡眠問題と	勤労者の睡	北村 尚人(	産業医科大学雑誌(0387-8	原著論文/	質問紙法;	労働時間	ヒト; 青年	10.7888/j	産業医科大学雑誌(0387-821X)36巻4号 Page295-300(2014.12)
2.02E+09	0	0	0	0	ed disease,	1	diet and n	diet and n	成人女性の	【目的】	西田 江里(	栄養学雑誌(0021-5147)72	原著論文/	質問紙法;	食物摂取	ヒト; 成人		栄養学雑誌(0021-5147)72巻6号 Page31-37(2014.12)
2.02E+09	0	0	0	0	ed disease,	0	diet and n	diet and n	成人男女に	本研究は、	高田 由美(	肥満研究(1343-229X)20巻	原著論文/	回帰分析;	実態調査;	ヒト; 青年		肥満研究(1343-229X)20巻3号 Page168-175(2014.12)
2.02E+09	0	0	0	0	ed disease,	1	diet and n	diet and n	働く女性の	近年わが国	平澤 芳恵(	日本職業・災害医学会会誌	原著論文/	育児; *食	実態調査	ヒト; 成人		日本職業・災害医学会会誌(1345-2592)62巻6号 Page399-403(2014.11)
2.02E+09	0	0	0	0	nt and disc	1	bullying	いじめ	職場のいじ	問題と目的	草海 由香	こころの健康(0912-6945)	原著論文	質問紙法;	構成概念	ヒト; 成人		こころの健康(0912-6945)29巻2号 Page59-69(2014.12)
2.02E+09	0	0	0	0	egnancy ar	1	balance w	balance w	妊娠期の	【目的】	中島 久美	母性衛生(0388-1512)55巻	原著論文/	回帰分析;	家事	ヒト; 成人		母性衛生(0388-1512)55巻4号 Page668-676(2015.01)
2.02E+09	0	0	0	0	rk environm	1	retention	turnover	看護師の	就労10年	加藤 礼識(	日本衛生学雑誌(0021-508	原著論文/	質問紙法;		ヒト; 成人	10.1265/jj	日本衛生学雑誌(0021-5082)70巻1号 Page33-39(2015.01)
2.02E+09	0	0	0	0	ed disease,	1	diet and n	diet and n	成人就労	名古屋、	上原 美穂,	日本衛生学雑誌(0021-508	原著論文/	食行動; *		ヒト; 成人	10.1265/jj	日本衛生学雑誌(0021-5082)70巻1号 Page54-61(2015.01)
2.01E+09	0	0	0	0	lental healt	0	stress management		心身両面の	職場のメン	亀井 真由	予防医学ジャーナル(0285-	原著論文/	質問紙法;	*保健指導	ヒト; 成人		予防医学ジャーナル(0285-0877)477号 Page66-70(2014.07)
2.02E+09	0	0	0	0	ed disease,	1	oral healtl	oral	勤労者に	目的:成人	市橋 透(ラ	産業衛生学雑誌(1341-072	原著論文/	質問紙法;		ヒト; 成人	10.1539/s	産業衛生学雑誌(1341-0725)57巻1号 Page1-8(2015.01)
2.02E+09	0	0	0	0	rk and bala	0	WFC	child-rear	乳幼児を	全国の200	丸山 昭子(	性とこころ(1884-7005)6巻	原著論文/	*育児; 看		ヒト; 女;		性とこころ(1884-7005)6巻2号 Page144-151(2014.12)
2.02E+09	0	0	0	0	related dis	1	shift	shift	40歳以上の	夜勤を担う	脇坂 浩(三	看護実践の科学(0385-428	原著論文/	質問紙法;	労働時間	ヒト; 中年		看護実践の科学(0385-4280)40巻5号 Page54-60(2015.05)
2.02E+09	0	0	0	0	rk and bala	1	WFC	child-rear	母親の育児	因子分析	寺見 陽子(	Journal of the Faculty of H	原著論文	質問紙法;	北区(神戸	ヒト; 成人		Journal of the Faculty of Human Sciences, Kobe Shoin Women's University(2186-3849)4号 Page59
2.02E+09	0	0	0	0	lental healt	1	occupatio	sleep	睡眠の位相	目的:ピッ	松本 悠貴(	産業衛生学雑誌(1341-072	原著論文	質問紙法;	自己評価	ヒト; 青年	10.1539/s	産業衛生学雑誌(1341-0725)56巻5号 Page128-140(2014.09)
2.02E+09	0	0	0	0	lental healt	1	occupational stress		女性訪問	女性訪問	峯岸 高裕(	北海道公衆衛生学雑誌(091	原著論文/	*労働衛生	自己評価	ヒト; 成人		北海道公衆衛生学雑誌(0914-2630)28巻2号 Page129-134(2015.03)
2.02E+09	0	0	0	0	related dis	0	comfortab	odo	病院環境に	看護職者を	佐藤 公美	札幌保健科学雑誌(2186-6	原著論文	*匂い; *病	*ベッドサ	ヒト; 成人		札幌保健科学雑誌(2186-621X)4号 Page25-32(2015.03)
2.02E+09	0	0	0	0	rk and bala	6	WFC	child-rear	働きながら	研究の目的	藤田 藍津	インターナショナルNursir	原著論文	*育児; 家族	家庭環境	ヒト; 成人		インターナショナルNursing Care Research(1347-1341)14巻2号 Page19-26(2015.05)
2.02E+09	0	0	0	0	lental healt	0	occupational stress		病院薬剤	【目的】	井奈波 良	日本職業・災害医学会会誌	原著論文/	労働衛生;	*病棟薬剤	ヒト; 成人		日本職業・災害医学会会誌(1345-2592)62巻5号 Page322-327(2014.09)
2.02E+09	0	0	0	0	lental healt	1	occupational stress		労働者に	目的 日本	土屋 政雄(	労働安全衛生研究(1882-6	原著論文/	*労働衛生		ヒト; 成人	10.2486/j	労働安全衛生研究(1882-6822)7巻2号 Page59-66(2014.09)
2.02E+09	0	0	0	0	lental healt	1	stress ma	workplace	製造業に	目的:NIOS	堀田 裕司(	産業衛生学雑誌(1341-072	原著論文	質問紙法;		ヒト; 成人	10.1539/s	産業衛生学雑誌(1341-0725)56巻6号 Page259-267(2014.11)
2.02E+09	0	0	0	0	lental healt	1	stress management		女性看護	【目的】	井奈波 良	日本職業・災害医学会会誌	原著論文/	*看護師;		ヒト; 成人		日本職業・災害医学会会誌(1345-2592)63巻2号 Page81-87(2015.03)
2.02E+09	0	0	0	0	egnancy ar	3	balance w	balance w	就労妊婦に	近年、有配	小川 彩(東	母性衛生(0388-1512)56巻	原著論文/	質問紙法;	アメニティ	ヒト; 妊娠		母性衛生(0388-1512)56巻2号 Page292-300(2015.07)
2.02E+09	0	0	0	0	egnancy ar	1	fertility		就労女性の	就労女性の	坪井 陽子(	母性衛生(0388-1512)56巻	原著論文	*労働衛生		ヒト; 成人		母性衛生(0388-1512)56巻2号 Page391-398(2015.07)
2.02E+09	0	0	0	0	lental healt	1	occupational stress		「職場で	職場で対応	川上 憲人(	産業医学ジャーナル(0388-	原著論文	因子分析;		ヒト; 成人		産業医学ジャーナル(0388-337X)38巻3号 Page42-48(2015.05)
2.02E+09	0	0	0	0	rk environm	1	retention	turnover	若年労働者	昨今,20〜	井田 浩正(	ストレス科学(1349-4813)	原著論文	危険因子;		ヒト; 成人		ストレス科学(1349-4813)29巻3号 Page293-307(2014.12)
2.02E+09	0	0	0	0	lental healt	1	stress management		急性期型	【目的】	中神 克之(	日本クリティカルケア看護	原著論文/	*看護師;		ヒト; 成人	10.11153/	日本クリティカルケア看護学会誌(1880-8913)10巻3号 Page1-9(2014.10)
2.02E+09	0	0	0	0	lental healt	1	occupatio	biomarker	【働く女性	筆者らの	小山 文彦(	産業精神保健(1340-2862)	原著論文/	Hydrocort	*視床下部	ヒト; 成人		産業精神保健(1340-2862)23巻特別号 Page89-93(2015.06)
2.02E+09	0	0	0	0	rk and bala	1	WFC	child-rear	未就学児の	未就学児の	鈴木 康宏(	日本健康医学会雑誌(1343-	原著論文/	コミュニケ		ヒト; 成人	10.20685/	日本健康医学会雑誌(1343-0025)24巻2号 Page114-129(2015.07)
2.02E+09	0	0	0	0	lental healt	1	occupational stress		精神科病棟	精神科病棟	福崎 俊貴(	産業ストレス研究(1340-7	原著論文	回帰分析;		ヒト; 成人		産業ストレス研究(1340-7724)22巻2号 Page153-161(2015.04)
2.02E+09	0	0	0	0	ed disease,	1	lifestyle	smoke	歯科大学附	喫煙は、	内山 恵理(	日本歯科人間ドック学会誌	原著論文/	Nicotine(		ヒト; 成人		日本歯科人間ドック学会誌(1345-9910)10巻1号 Page34-40(2015.08)
2.02E+09	0	0	0	0	lental healt	1	stress ma	workplace	上司のリー	部下が評価	津野 香奈	産業ストレス研究(1340-7	原著論文	*労働衛生	社会関係	ヒト; 成人		産業ストレス研究(1340-7724)22巻2号 Page163-171(2015.04)
2.02E+09	0	0	0	0	lental healt	0	stress management		介護老人福	介護老人福	足利 和子(	ストレス科学(1349-4813)	原著論文/	質問紙法;	*介護負担	ヒト; 成人		ストレス科学(1349-4813)29巻3号 Page318-322(2014.12)
2.02E+09	0	0	0	0	n/ satisfac	1	career	scale deve	「女性医師	医師不足	堀江 早喜(	日本衛生学雑誌(0021-508	原著論文	因子分析;		ヒト; 成人	10.1265/jj	日本衛生学雑誌(0021-5082)70巻3号 Page264-270(2015.09)
2.02E+09	0	0	0	0	n/ satisfac	4	education	health prc	職場におい	目的:職場	堀田 裕司(	産業衛生学雑誌(1341-072	原著論文	質問紙法;		ヒト; 成人	10.1539/s	産業衛生学雑誌(1341-0725)57巻5号 Page219-229(2015.09)
2.02E+09	0	0	0	0	opause, ar	1	Uterine_c	子宮がん	若年女性	目的:20歳	河田 志帆(	日本公衆衛生看護学会誌(2	原著論文/	結婚; *子	*受療行動	ヒト; 成人	10.15078/	日本公衆衛生看護学会誌(2187-7122)4巻1号 Page41-47(2015.06)
2.02E+09	0	0	0	0	lental healt	0	occupational stress		病理医の	【目的】	井奈波 良	日本職業・災害医学会会誌	原著論文/	*医師; 飲	労働時間	ヒト; 成人		日本職業・災害医学会会誌(1345-2592)63巻4号 Page225-231(2015.07)
2.02E+09	0	0	0	0	lental healt	1	occupational stress		労働者の	目的 収入	堤 明純(北	厚生の指標(0452-6104)62	原著論文/	質問紙法;	労働時間	ヒト; 成人		厚生の指標(0452-6104)62巻11号 Page1-8(2015.09)
2.02E+09	0	0	0	0	rk and bala	1	WFC	child-rear	子育てをし	本研究は、	近末 清美(	日本看護管理学会誌(1347-	原著論文/	育児; 看護		ヒト; 成人	10.19012/	日本看護管理学会誌(1347-0140)19巻2号 Page67-75(2015.12)
2.02E+09	0	0	0	0	nt and disc	0	bullying	いじめ	民間事業所	本研究では	山本 沙弥	心的トラウマ研究(1880-2	原著論文/	質問紙法;	実態調査	ヒト; 成人		心的トラウマ研究(1880-2109)11号 Page9-17(2015.11)
2.02E+09	0	0	0	0	rk and bala	0	WFC	child-rear	ジェンダー		石 暁玲(東	家族心理学研究(0915-062	原著論文	質問紙法;	GHQ (精神	ヒト; 成人		家族心理学研究(0915-0625)29巻2号 Page99-113(2015.12)
2.02E+09	0	0	0	0	egnancy ar	0	QOL	QOL	数量化III類	本研究は、	西川 みゆ	母性衛生(0388-1512)56巻	原著論文/	質問紙法;		ヒト; 妊娠		母性衛生(0388-1512)56巻4号 Page548-557(2016.01)
2.02E+09	0	0	0	0	lental healt	1	occupatio	fatigue	女性看護	目的:女性	菊地 由紀	産業衛生学雑誌(1341-072	原著論文/	質問紙法;		ヒト; 成人	10.1539/s	産業衛生学雑誌(1341-0725)57巻5号 Page230-240(2015.09)
2.02E+09	0	0	0	0	lental healt	4	stress ma	physical a	女性勤労者	検診事業	中原 雄一	体力研究(0389-9071)113	原著論文/	*情動; 睡眠		ヒト; 成人	10.20793/	体力研究(0389-9071)113号 Page15-18(2015.04)
2.02E+09	0	0	0	0	rk and bala	1	workstyle	workstyle	働く女性に		榊原 圭子	ストレス科学研究(1341-9	原著論文	質問紙法;		ヒト; 成人	10.5058/s	ストレス科学研究(1341-9986)30巻 Page83-89(2015.11)
2.02E+09	0	0	0	0	lental healt	1	occupational stress		自己価値の	職場での自	笹川 果央	ストレス科学研究(1341-9	原著論文	回帰分析;		ヒト; 成人	10.5058/s	ストレス科学研究(1341-9986)30巻 Page131-137(2015.11)
2.02E+09	0	0	0	0	lental healt	1	occupational stress		女性看護	【目的】	井奈波 良	日本職業・災害医学会会誌	原著論文/	*看護師; *		ヒト; 成人		日本職業・災害医学会会誌(1345-2592)63巻5号 Page290-296(2015.09)
2.02E+09	0	0	0	0	ed disease,	1	diet and n	diet and n	血液中酸化	医療機関の	渡辺 洋子(	DNA多型(2188-3815)23巻	原著論文/	*過酸化水	p22-phox;	ヒト; 成人		DNA多型(2188-3815)23巻1号 Page181-184(2015.07)
2.02E+09	0	0	0	0	opause, ar	0	Repro_ca	婦人科がん	就業女性(	就業女性(	赤星 琴美(	看護展望(0385-549X)41巻	原著論文/	一次予防;	受診率; *	ヒト; 成人		看護展望(0385-549X)41巻5号 Page0503-0507(2016.03)
2.02E+09	0	0	0	0	egnancy ar	0	QOL	QOL	妊娠初期の	本研究の目	中田 覚子(	母性衛生(0388-1512)57巻	原著論文/	家族特性;	実態調査	ヒト; 妊娠		母性衛生(0388-1512)57巻1号 Page131-137(2016.04)
2.02E+09	0	0	0	0	egnancy ar	0	work, nutr	work, nutr	就労妊婦の	近年、有配	中村 康香(	日本母性看護学会誌(1345-	原著論文/	育児; *運動	*運動量	ヒト; 妊娠		日本母性看護学会誌(1345-773X)16巻1号 Page33-40(2016.03)
2.02E+09	0	0	0	0	ed disease,	0	diet and n	diet and n	ストレス対	【目的】	鈴木 亜紀	栄養学雑誌(0021-5147)73	原著論文/	*過食; 労働	満腹	ヒト; 成人	10.5264/e	栄養学雑誌(0021-5147)73巻6号 Page221-229(2015.12)
2.02E+09	0	0	0	0	lental healt	1	occupatio	predictor	労働者の	労働者の	原谷 隆史(	労働安全衛生総合研究所特	原著論文	*労働衛生	自己評価	ヒト; 成人		労働安全衛生総合研究所特別研究報告(1882-8329)45号 Page53-60(2015.12)
2.02E+09	0	0	0	0	rk and bala	0	WFC	child-rear	家庭および	２４０名訓	川畑 早紀(	病児保育研究(2187-6118)	原著論文/	咳嗽; *家	*家庭環境	ヒト; 成人		病児保育研究(2187-6118)5号 Page37-41(2014.07)
2.02E+09	0	0	0	0	rk environm	1	retention	turnover	行政保健	目的 保健	井口 理(東	日本公衆衛生雑誌(0546-1	原著論文	因子分析;		ヒト; 成人	10.11236/	日本公衆衛生雑誌(0546-1766)63巻5号 Page227-240(2016.05)
2.02E+09	0	0	0	0	lental healt	1	stress management		対人援助	A県在住の	竹田 伸也(	ストレス科学研究(1341-9	原著論文/	質問紙法;		ヒト; 成人	10.5058/s	ストレス科学研究(1341-9986)30巻 Page44-51(2015.11)
2.02E+09	0	0	0	0	rk and bala	0	workstyle	workstyle	女性医師の	目的:女性	片岡 仁美(	医学教育(0386-9644)47巻	原著論文/	労働衛生;	実態調査	ヒト; 成人	10.11307/	医学教育(0386-9644)47巻2号 Page111-123(2016.04)
2.02E+09	0	0	0	0	lental healt	1	occupational stress		看護師の	看護師を対	大植 崇(兵	インターナショナルNursir	原著論文	質問紙法;		ヒト; 成人		インターナショナルNursing Care Research(1347-1341)15巻1号 Page75-84(2016.04)
2.02E+09	0	0	0	0	related dis	0	shift	shift	日中短時間	本研究は、	細名 水生(	インターナショナルNursir	原著論文/	看護師; *	感情プロフ	ヒト; 成人		インターナショナルNursing Care Research(1347-1341)15巻2号 Page103-111(2016.05)
2.02E+09	0	0	0	0	opause, ar	0	menopaus	職場支援・	更年期世代	(目的)更年	三羽 良枝(	日本女性医学学会雑誌(21	原著論文	質問紙法;		ヒト; 成人		日本女性医学学会雑誌(2185-8861)23巻2号 Page328-334(2016.04)
2.02E+09	0	0	0	0	opause, ar	0	menopaus	実態・意識	更年期にあ	本研究では	兼宗 美幸(	更年期と加齢のヘルスケア	原著論文/	高血圧(合		ヒト; 成人		更年期と加齢のヘルスケア(1347-801X)15巻1号 Page22-26(2016.06)
2.02E+09	0	0	0	0	opause, ar	1	hormone t	ホルモン	ホルモン補	【背景・目	峯村 昌子(	更年期と加齢のヘルスケア	原著論文/	*エストロ		ヒト; 成人		更年期と加齢のヘルスケア(1347-801X)15巻1号 Page70-80(2016.06)
2.02E+09	0	0	0	0	rk environm	6	retention	turnover	女性看護	女性看護	齋藤 千秋(	地域と住民(0288-4917)34	原著論文	育児; *看	*労働時間	ヒト; 成人		地域と住民(0288-4917)34号 Page57-66(2016.03)
2.02E+09	0	0	0	0	opause, ar	1	Breast_ca	乳がん	職域乳がん	2006年4月	山口 孝子(	総合健診(1347-0086)43巻	原著論文	*集団検診	受診率; 精	ヒト; 成人	10.7143/j	総合健診(1347-0086)43巻3号 Page419-428(2016.05)
2.02E+09	0	0	0	0	rk and bala	0	WFC	caregiver	勤労者にお	目的 介護	中原 雄一	厚生の指標(0452-6104)63	原著論文/	質問紙法;		ヒト; 成人		厚生の指標(0452-6104)63巻5号 Page1-6(2016.05)
2.02E+09	0	0	0	0	lental healt	1	occupational stress		女性病院	【目的】	井奈波 良	日本職業・災害医学会会誌	原著論文/	看護師; 眼		ヒト; 成人		日本職業・災害医学会会誌(1345-2592)64巻3号 Page145-149(2016.05)
2.02E+09	0	0	0	0	nt and disc	0	harassme	ハラスメン	【援助職と	A県内の訪	開田 ひと	性とこころ(1884-7005)7巻	原著論文/	*看護倫理		ヒト; 成人		性とこころ(1884-7005)7巻1号 Page45-60(2015.10)
2.02E+09	0	0	0	0	rk environm	1	engagement		病棟看護	看護師を対	加賀田 聡	行動医学研究(1341-6790)	原著論文	回帰分析;		ヒト; 成人	10.11331/	行動医学研究(1341-6790)21巻2号 Page83-90(2015.10)
2.02E+09	0	0	0	0	n/ satisfac	1	satisfaction		認知症ケア	認知症ケア	笹賀 美代	日本認知症ケア学会誌(18	原著論文	因子分析;	実態調査	ヒト; 成人		日本認知症ケア学会誌(1882-0255)15巻2号 Page457-469(2016.07)
2.02E+09	0	0	0	0	related dis	0	shift	shift	16時間夜勤	本研究は、	松元 俊(大	労働科学(0022-443X)91巻	原著論文	*人間活動	*生活時間	ヒト; 成人	10.11355/	労働科学(0022-443X)91巻3-4号 Page39-44(2015.06)
2.02E+09	0	0	0	0	rk environm	0	retention	turnover	自治医科大	自治医科大	牧野 伸子(	地域医学(0914-4277)30巻	原著論文	病院診療		ヒト; 成人		地域医学(0914-4277)30巻6号 Page462-466(2016.06)
2.02E+09	0	0	0	0	ed disease,	0	physical a	exercise	事務的職業	本研究では	塩満 智子(	日本健康医学会雑誌(1343-	原著論文/	質問紙法;	体力テスト	ヒト; 成人	10.20685/	日本健康医学会雑誌(1343-0025)25巻2号 Page107-113(2016.07)
2.02E+09	0	0	0	0	ed disease,	1	diet and n	diet and n	勤労者にお	野菜および	串田 修(新	生活科学研究誌(1348-692	原著論文	因子分析;	食物摂取	ヒト; 成人		生活科学研究誌(1348-6926)14巻 Page21-28(2016.04)
2.02E+09	0	0	0	0	rk and bala	0	WFC	child-rear	岐阜大学医	医師・歯科	操 奈美(岐	地域医学(0914-4277)30巻	原著論文	質問紙法;		ヒト; 成人		地域医学(0914-4277)30巻8号 Page656-665(2016.08)
2.02E+09	0	0	0	0	ed disease,	1	physical a	exercise	都市規模に	目的 近年	井原 正裕(	日本公衆衛生雑誌(0546-1	原著論文/	*栄養調査		ヒト; 成人	10.11236/	日本公衆衛生雑誌(0546-1766)63巻9号 Page549-559(2016.09)
2.02E+09	0	0	0	0	rk environm	1	engagement		介護福祉士	目的 介護	時實 亮(岡	厚生の指標(0452-6104)63	原著論文	回帰分析;		ヒト; 成人		厚生の指標(0452-6104)63巻12号 Page7-13(2016.10)
2.02E+09	0	0	0	0	rk and bala	1	WFC	caregiver	在宅介護者	【目的】	栗林 佑希	日本医学看護学教育学会誌	原著論文/	回帰分析;	介護者-患	ヒト; 中年		日本医学看護学教育学会誌25-2号 Page28-39(2016.10)
2.02E+09	0	0	0	0	rk environm	1	engageme	scale deve	複数企業の	目的:プレ	井田 浩正(	産業衛生学雑誌(1341-072	原著論文	因子分析;	労働時間	ヒト; 成人	10.1539/s	産業衛生学雑誌(1341-0725)59巻1号 Page1-8(2017.01)
2.02E+09	0	0	0	0	rk environm	1	productivi	presentee	日本版Pre	目的 日本	荒木田 美	厚生の指標(0452-6104)63	原著論文	質問紙法;	SF-36	ヒト; 成人		厚生の指標(0452-6104)63巻13号 Page6-12(2016.11)
2.02E+09	0	0	0	0	egnancy ar	1	mental he	mental he	4ヵ月児健	本研究は、	山崎 恭子(	お茶の水医学雑誌(0472-4	原著論文/	質問紙法;	自己評価	ヒト; 成人		お茶の水医学雑誌(0472-4674)65巻1号 Page79-85(2017.01)
2.02E+09	0	0	0	0	rk and bala	6	workstyle	workstyle	企業内ワー	本研究は、	井上 奈穂	カウンセリング研究(0914-	原著論文	育児; *労		ヒト; 成人	10.11544/	カウンセリング研究(0914-8337)48巻4号 Page175-188(2015.11)
2.02E+09	0	0	0	0	n/ satisfac	0	career	医療従事者	日本大学歯	近年、女性	河越 邦子(	日本歯科医学教育学会雑誌	原著論文	質問紙法;	進路選択	ヒト; 成人	10.24744/	日本歯科医学教育学会雑誌(0914-5133)32巻3号 Page173-183(2016.12)
2.02E+09	0	0	0	0	lental healt	1	occupational stress		介護付有料	A県のB介	田中 康雄(	社会福祉科学研究(2186-7	原著論文	労働衛生;		ヒト; 成人		社会福祉科学研究(2186-7798)5号 Page45-55(2016.07)
2.02E+09	0	0	0	0	egnancy ar	0	work, nutr	work, nutr	「就労妊婦	本研究は、	山中 絵美(	母性衛生(0388-1512)57巻	原著論文	労働衛生;		ヒト; 妊娠		母性衛生(0388-1512)57巻4号 Page625-634(2017.01)
2.02E+09	0	0	0	0	rk and bala	1	WFC	WFC	就労してい	【目的】	今井 充子(	日本ウーマンズヘルス学会	原著論文	*育児; *人	GHQ (精神	ヒト; 成人		日本ウーマンズヘルス学会誌(1347-5894)15巻2号 Page41-48(2017.03)
2.02E+09	0	0	0	0	rk and bala	1	WFC	WFC	女性病院	【目的】	井奈波 良	日本職業・災害医学会会誌	原著論文/	病院診療		ヒト; 成人		日本職業・災害医学会会誌(1345-2592)64巻6号 Page319-325(2016.11)
2.02E+09	0	0	0	0	rk and bala	1	workstyle	workstyle	既婚女性	本研究では	本島 茉那	日本健康医学会雑誌(1343-	原著論文/	*看護師; *	ロールモデ	ヒト; 成人	10.20685/	日本健康医学会雑誌(1343-0025)26巻1号 Page7-16(2017.04)
2.02E+09	0	0	0	0	rk environm	1	retention	turnover	両立支援	目的:両立	下條 祐也(	日本看護科学会誌(0287-5	原著論文	因子分析;		ヒト; 成人	10.5630/j	日本看護科学会誌(0287-5330)36巻 Page51-59(2016.12)

2.02E+09	0	0	0	0	opause, an	1	menstrati	月経	働く女性の	本研究の目	大滝 千智(	日本母性看護学会誌(1345-	原著論文/	温熱療法;		ヒト; 成人		日本母性看護学会誌(1345-773X)17巻1号 Page53-60(2017.03)
2.02E+09	0	0	0	0	ed disease,	1	LRD	CVD	勤労者の心	肥満は心血	水野 杏一(	総合健診(1347-0086)44巻	原著論文/	Triglyceric	空腹時血糖	ヒト; 男; 女	10.7143/jl	総合健診(1347-0086)44巻3号 Page492-498(2017.05)
2.02E+09	0	0	0	0	rk and bala	1	WFC	caregiver	要介護者を	要介護者を	桜井 志保	保健の科学(0018-3342)59	原著論文/	Secretory	*介護負担	ヒト; 中年		保健の科学(0018-3342)59巻6号 Page423-429(2017.06)
2.02E+09	0	0	0	0	rk and bala	0	WFC	caregiver	都道府県別	目的 介護	渡邊 多永	厚生の指標(0452-6104)64	原著論文	健康調査;	地域差	ヒト; 成人		厚生の指標(0452-6104)64巻6号 Page16-20(2017.06)
2.02E+09	0	0	0	0	related dis	1	Musle ske	MusleSke	医療介護	[目的]医療	木村 文佳(	理学療法科学(1341-1667)	原著論文/	月経異常;		ヒト; 成人	10.1589/r	理学療法科学(1341-1667)32巻2号 Page243-247(2017.04)
2.02E+09	0	0	0	0	n/ satisfac	1	satisfactic	医療従事者	臨床工学技	臨床工学技	木村 政義(	日本臨床工学技士会会誌(1	原著論文/	質問紙法;		ヒト; 成人		日本臨床工学技士会会誌(1341-3171)59号 Page91-96(2017.01)
2.02E+09	0	0	0	0	n/ satisfac	0	self-effica	motivatior	【予防と早	急性期病院	梅田 ルミ(	看護部長通信15巻2号 Pag	原著論文/	看護師; *労	実態調査	ヒト; 成人		看護部長通信15巻2号 Page32-36(2017.06)
2.02E+09	0	0	0	0	k environm	1	retention	turnover	介護福祉士	[目的]本研	柏原 正尚(	介護福祉学(1340-8178)23	原著論文	質問紙法;		ヒト; 成人		介護福祉学(1340-8178)23巻2号 Page137-144(2017.04)
2.02E+09	0	0	0	0	lental healt	1	overtime a	overtime c	総労働時間	[目的]過労	福山 和恵(	日本職業・災害医学会会誌	原著論文/	回帰分析;	自己評価ま	ヒト; 成人		日本職業・災害医学会会誌(1345-2592)65巻3号 Page147-152(2017.05)
2.02E+09	0	0	0	0	rk and bala	0	WFC	WFC	男性看護師	男性看護師	鈴木 康宏(	日本健康医学会雑誌(1343-	原著論文/	看護師; *労		ヒト; 成人	10.20685/	日本健康医学会雑誌(1343-0025)26巻2号 Page86-92(2017.07)
2.02E+09	0	0	0	0	related dis	4	shift	shift	夜間にとる	本研究の目	折山 早苗(	労働科学(0022-443X)93巻	原著論文/	*運動活性	眠気(診断)	ヒト; 成人	10.11355/	労働科学(0022-443X)93巻3号 Page67-79(2017.06)
2.02E+09	0	0	0	0	related dis	3	Musle ske	MusleSke	体力指標を	腰痛は全職	吉丸 優希(	総合保健体育科学(0289-54	原著論文	*体力; *腰		ヒト; 成人		総合保健体育科学(0289-5412)40巻1号 Page15-20(2017.06)
2.02E+09	0	0	0	0	ed disease,	1	diet and n	sarcopeni	中高年勤労	【背景】高	渡会 敦子(	日本職業・災害医学会会誌	原著論文	*運動活性		ヒト; 成人		日本職業・災害医学会会誌(1345-2592)65巻5号 Page269-275(2017.09)
2.02E+09	0	0	0	0	opause, an	0	Uterine_m	子宮筋腫	就労女性た	【目的】女	宮内 文久(	日本職業・災害医学会会誌	原著論文	質問紙法;	受療行動	ヒト; 成人		日本職業・災害医学会会誌(1345-2592)65巻5号 Page276-282(2017.09)
2.02E+09	0	0	0	0	n/ satisfac	1	career		女性病院看	【目的】女	井奈波 良一	日本職業・災害医学会会誌	原著論文/	看護師; *労	労働時間	ヒト; 成人		日本職業・災害医学会会誌(1345-2592)65巻4号 Page160-165(2017.07)
2.02E+09	0	0	0	0	lental healt	0	occupational stress		調理業務に	目的 近年	小濱 絵美(	東海公衆衛生雑誌(2187-73	原著論文	機器の汚染	機器の洗浄	ヒト; 成人	10.24802/	東海公衆衛生雑誌(2187-736X)5巻1号 Page84-88(2017.07)
2.02E+09	0	0	0	0	lental healt	1	other	pain	就労環境に	目的:就労	山田 恵子(	産業衛生学雑誌(1341-0725	原著論文/	危険因子;	実態調査	ヒト; 成人	10.1539/s	産業衛生学雑誌(1341-0725)59巻5号 Page125-134(2017.09)
2.02E+09	0	0	0	0	rk and bala	1	WFC	WFC	職業性スト	メンタルヘ	足立 勝宣(	日本精神科看護学術集会誌	原著論文	質問紙法;		ヒト; 成人		日本精神科看護学術集会誌59巻2号 Page358-362(2017.07)
2.02E+09	0	0	0	0	opause, an	0	menstrati	月経	女性特有の	子宮筋腫を	宮内 文久(	日本職業・災害医学会会誌	原著論文/	質問紙法;		ヒト; 成人		日本職業・災害医学会会誌(1345-2592)65巻6号 Page350-357(2017.11)
2.02E+09	0	0	0	0	ed disease,	1	lifestyle	smoke	喫煙歴とた	目的 がん検	山田 恵子(	厚生の指標(0452-6104)64	原著論文/	*タバコ喫	*受診率	ヒト; 成人		厚生の指標(0452-6104)64巻12号 Page1-6(2017.10)
2.02E+09	0	0	0	0	lental healt	1	occupational stress		特別養護老	特別養護老	田中 康雄(	地域福祉サイエンス(2188-	原著論文/	労働衛生;		ヒト; 成人		地域福祉サイエンス(2188-2886)3号 Page57-66(2016.10)
2.02E+09	0	0	0	0	rk and bala	1	WFC	caregiver	看護師の仕	本研究は、	松本 明美(	ヘルスサイエンス研究(134	原著論文	因子分析;	*アンビバ	ヒト; 成人		ヘルスサイエンス研究(1343-3393)21巻1号 Page3-13(2017.11)
2.02E+09	0	0	0	0	rk and bala	1	WFC	WFC	乳幼児を育	本研究は、	梅田 弘子(	母性衛生(0388-1512)58巻	原著論文	*育児; 因子	育児相談	ヒト; 成人		母性衛生(0388-1512)58巻4号 Page541-548(2018.01)
2.02E+09	0	0	0	0	egnancy ar	1	stat	stat	母親の年齢	目的 人口動	仙田 幸子(	厚生の指標(0452-6104)65	原著論文/	経産回数;		ヒト; 妊娠		厚生の指標(0452-6104)65巻1号 Page1-7(2018.01)
2.02E+09	0	0	0	0	k environm	0	retention	turnover	透析医療に	全国の私立	菅野 丈夫(	日本透析医学会雑誌(1340-	原著論文	質問紙法;		ヒト; 男; 女	10.4009/js	日本透析医学会雑誌(1340-3451)51巻1号 Page71-73(2018.01)
2.02E+09	0	0	0	0	related dis	0	shift	shift	女性看護師	女性看護師	田中 美穂(	産業保健人間工学研究(134	原著論文	*看護師; 労		ヒト; 成人		産業保健人間工学研究(1345-1731)19巻増補 Page45-48(2017.09)
2.02E+09	0	0	0	0	lental healt	1	occupatio	predictor	職業性スト	【目的】ス	足立 勝宣(	日本職業・災害医学会会誌	原著論文	労働衛生;	自己評価ま	ヒト; 青年		日本職業・災害医学会会誌(1345-2592)66巻1号 Page33-39(2018.01)
2.02E+09	0	0	0	0	related dis	1	Musle ske	MusleSke	女性家族介	目的:女性	鈴木 岸子(	日本看護研究学会雑誌(028	原著論文/	質問紙法;	介護者-患	ヒト; 中年	10.15065/	日本看護研究学会雑誌(0285-9262)40巻4号 Page623-630(2017.09)
2.02E+09	0	0	0	0	opause, an	0	menstrati	月経	婦人科検診	就労女性の	五十嵐 信一	日本農村医学会雑誌(0468-	原著論文/	月経困難症		ヒト; 成人	10.2185/jj	日本農村医学会雑誌(0468-2513)66巻5号 Page562-566(2018.01)
2.02E+09	0	0	0	0	rk and bala	0	WFC	child-rear	リハビリテ	[目的]妊娠	藤原 愛作(	理学療法科学(1341-1667)	原著論文	質問紙法;		ヒト; 妊娠	10.1589/r	理学療法科学(1341-1667)33巻1号 Page13-18(2018.02)
2.02E+09	0	0	0	0	rk and bala	0	workstyle	workstyle	宮崎県にお	【はじめに	福本 周子(	臨床と理学療法(2188-550	原著論文	志気; ライ	実態調査	ヒト; 成人		臨床と理学療法(2188-5508)5巻1号 Page38-46(2018.01)
2.02E+09	0	0	0	0	ed disease,	1	other	pain	慢性痛を呈	【背景】近	甘利 貴志(	Pain Rehabilitation(2186-	原著論文/	*運動活性	歩数計	ヒト; 成人		Pain Rehabilitation(2186-2702)8巻1号 Page34-42(2018.03)
2.02E+09	0	0	0	0	egnancy ar	0	safety, wc	safety, wc	大学病院	日本小児科	福原 里恵,	日本小児科学会雑誌(0001-	原著論文	質問紙法;	労働時間	ヒト; 男; 女		日本小児科学会雑誌(0001-6543)122巻4号 Page844-848(2018.04)
2.02E+09	0	0	0	0	lental healt	0	occupatio	biomarker	福祉の現場	本研究は、	松浦 義昌(	地域ケアリング(1345-012	原著論文/	サーカディ		ヒト; 成人		地域ケアリング(1345-0123)20巻5号 Page100-102(2018.05)
2.02E+09	0	0	0	0	opause, an	6	menstrati	月経	働く若年女	本研究は、	樋口 紫音(	臨床心理学(1345-9171)18	原著論文	葛藤; 質問	*苦痛; 眠気	ヒト; 成人		臨床心理学(1345-9171)18巻3号 Page347-357(2018.05)
2.02E+09	0	0	0	0	rk and bala	1	WFC	child-rear	事業場にお		青柳 美樹(	日本健康学会誌(2432-671	原著論文	*育児; *労	労働時間	ヒト; 成人	10.3861/k	日本健康学会誌(2432-6712)84巻3号 Page95-108(2018.05)
2.02E+09	0	0	0	0	rk and bala	1	WFC	child-rear	育児期女性		伊藤 里菜(	発達心理臨床研究(1346-04	原著論文	質問紙法;		ヒト; 成人		発達心理臨床研究(1346-0471)24巻 Page17-26(2018.03)
2.02E+09	0	0	0	0	lental healt	1	stress ma	stress che	ストレスチ	職業性スト	堤 明純(北	ストレス科学(1349-4813)	原著論文	質問紙法;		ヒト; 成人		ストレス科学(1349-4813)32巻3号 Page219-228(2018.03)
2.02E+09	0	0	0	0	opause, an	1	Uterine_m	子宮筋腫	子宮筋腫J	全国の労災	宮内 文久(	日本職業・災害医学会会誌	原著論文/	質問紙法;	受療行動	ヒト; 成人		日本職業・災害医学会会誌(1345-2592)66巻2号 Page129-137(2018.03)
2.02E+09	0	0	0	0	rk and bala	1	WFC	child-rear	女性看護職	本研究は、	岸本 長代(	インターナショナルNursir	原著論文	*育児; *労		ヒト; 成人		インターナショナルNursing Care Research(1347-1341)17巻2号 Page9-17(2018.05)
2.02E+09	0	0	0	0	egnancy ar	0	BF	BF	働く女性の		島 沙槻(東	東京母性衛生学会誌34巻1	原著論文/	質問紙法;	労働時間	ヒト; 成人		東京母性衛生学会誌34巻1号 Page54-61(2018.03)
2.02E+09	0	0	0	0	rk and bala	1	WFC	caregiver	親の介護を	親の介護を	高林 知佳	日本健康医学会雑誌(1343-	原著論文	*葛藤; 因子		ヒト; 成人	10.20685/	日本健康医学会雑誌(1343-0025)27巻1号 Page34-43(2018.04)
2.02E+09	0	0	0	0	lental healt	0	overtime a	overtime c	【教職員の	大阪府A市	大川 尚子(	労働の科学(0035-7774)73	原著論文/	質問紙法;	自律神経	ヒト; 成人		労働の科学(0035-7774)73巻5号 Page271-275(2018.05)
2.02E+09	0	0	0	0	ed disease,	1	LRD	lipid	職域集団の	2013年の	旭 久美子(	日本食生活学会誌(1346-97	原著論文	HDL Chol		ヒト; 成人	10.2740/ji	日本食生活学会誌(1346-9770)29巻1号 Page23-33(2018.06)
2.02E+09	0	0	0	0	ed disease,	0	physical a	exercise	職場体操た	目的:本研	谷 直道(日	産業衛生学雑誌(1341-0725	原著論文	質問紙法;	*身体能力	ヒト; 成人	10.1539/s	産業衛生学雑誌(1341-0725)60巻4号 Page85-93(2018.07)
2.02E+09	0	0	0	0	rk and bala	0	rework	rework	女性医師・	近年、ライ	加藤 沙弥(	日本シミュレーション医療	原著論文	質問紙法;	ロボット	ヒト; 成人		日本シミュレーション医療教育学会雑誌(2187-9281)5巻 Page66-70(2017.08)
2.02E+09	0	0	0	0	lental healt	1	occupational stress		コールセン	睡眠時間に	榎 真美子(	秋田県公衆衛生学雑誌(134	原著論文/	回帰分析;		ヒト; 成人		秋田県公衆衛生学雑誌(1348-9305)14巻1号 Page15-21(2018.05)
2.02E+09	0	0	0	0	egnancy ar	6	BF	BF	出産後に仕	目的 本研究	中田 かお	日本助産学会誌(0917-635	原著論文	*労働衛生		ヒト; 成人	10.3418/jj	日本助産学会誌(0917-6357)32巻1号 Page49-59(2018.06)
2.02E+09	0	0	0	0	lental healt	0	occupational stress		中小企業	日本の自殺	船古 崇徳(	昭和学士会雑誌(2187-719	原著論文/	*労働衛生		ヒト; 成人	10.14930/	昭和学士会雑誌(2187-719X)78巻1号 Page38-47(2018.02)
2.02E+09	0	0	0	0	opause, an	1	menstrati	月経	就業看護職	目的:就業	萩原 真美,	母性衛生(0388-1512)59巻	原著論文/	因子分析;		ヒト; 成人		母性衛生(0388-1512)59巻2号 Page329-335(2018.07)
2.02E+09	0	0	0	0	rk and bala	1	WFC	WFC	未就学児を	本研究の目	荒生 有花(	母性衛生(0388-1512)59巻	原著論文	*育児; 因子		ヒト; 成人		母性衛生(0388-1512)59巻2号 Page432-440(2018.07)
2.02E+09	0	0	0	0	related dis	1	Musle ske	MusleSke	TAMAツー	療養病棟で	田丸 朋子(	Health and Behavior Scier	原著論文/	質問紙法;		ヒト; 成人	10.32269/	Health and Behavior Sciences(1348-0898)17巻1号 Page7-14(2018.09)
2.02E+09	0	0	0	0	related dis	0	Musle ske	MusleSke	介護職職員	【目的】介	片山 良仁(	日本職業・災害医学会会誌	原著論文/	*X線診断;		ヒト; 成人		日本職業・災害医学会会誌(1345-2592)66巻5号 Page368-376(2018.09)
2.02E+09	0	0	0	0	lental healt	0	occupational stress		病院薬剤師	【目的】病	井奈波 良一	日本職業・災害医学会会誌	原著論文/	労働衛生;		ヒト; 成人		日本職業・災害医学会会誌(1345-2592)66巻3号 Page149-155(2018.05)
2.02E+09	0	0	0	0	k environm	6	retention	turnover	病院に就業	目的 キャ	高桑 優子(	医療看護研究(1349-8630)	原著論文/	*看護師; 労	中範囲理論	ヒト; 成人		医療看護研究(1349-8630)15巻1号 Page8-20(2018.10)
2.02E+09	0	0	0	0	ed disease,	0	lifestyle	smoke	教職員の喫	本学に在籍	和泉 恵子(	CAMPUS HEALTH(1341-4	原著論文/	*タバコ喫		ヒト; 成人		CAMPUS HEALTH(1341-4313)55巻1号 Page238-240(2018.03)
2.02E+09	0	0	0	0	related dis	0	shift	shift	夜勤勤務	2014年10	佐々木 信	看護のチカラ23巻504号 P	原著論文	*労働衛生	眠気(診断)	ヒト; 成人		看護のチカラ23巻504号 Page48-53(2018.11)
2.02E+09	0	0	0	0	opause, an	1	menopaus	work life b	就労更年期	【目的】近	永谷 実穂(	更年期と加齢のヘルスケア	原著論文	質問紙法;		ヒト; 中年		更年期と加齢のヘルスケア(1347-801X)17巻2号 Page165-174(2018.12)
2.02E+09	0	0	0	0	ed disease,	4	diet and n	diet and n	勤労者を対	【目的】社	入山 八江(	栄養学雑誌(0021-5147)76	原著論文/	*減塩食; 労		ヒト; 成人	10.5264/e	栄養学雑誌(0021-5147)76巻6号 Page139-155(2018.12)
2.02E+09	0	0	0	0	ed disease,	1	diet and n	locomo	姿勢重心計	本研究では	塩満 智子(	日本健康医学会雑誌(1343-	原著論文/	*筋骨格系	計測器; 体	ヒト; 成人	10.20685/	日本健康医学会雑誌(1343-0025)27巻2号 Page118-124(2018.07)
2.02E+09	0	0	0	0	egnancy ar	0	safety, wc	safety, wc	本邦におけ	妊娠中のM	山口 さち	日本磁気共鳴医学会雑誌(0	原著論文	質問紙法;		ヒト; 成人	10.2463/jj	日本磁気共鳴医学会雑誌(0914-9457)38巻4号 Page103-119(2018.11)
2.02E+09	0	0	0	0	rk and bala	1	rework	rework	がんサバイ	がんサバイ	清水 佐智	Palliative Care Research(	原著論文/	質問紙法;	労働時間	ヒト; 成人	10.2512/js	Palliative Care Research(1880-5302)13巻3号 Page209-218(2018.09)
2.02E+09	0	0	0	0	lental healt	1	occupatio	burnout a	女性病院看	【目的】女	井奈波 良一	日本職業・災害医学会会誌	原著論文/	*運動活性		ヒト; 成人		日本職業・災害医学会会誌(1345-2592)66巻4号 Page253-258(2018.07)
2.02E+09	0	0	0	0	nt and disc	1	bullying	いじめ	職場におけ	【問題と目	草海 由香	ストレスマネジメント研究	原著論文	質問紙法;		ヒト; 成人		ストレスマネジメント研究(1348-5180)14巻2号 Page58-66(2018.09)
2.02E+09	0	0	0	0	rk and bala	1	WFC	WLB	看護・社会	看護と社会	馬場 保子(	キャリアと看護研究(1884-	原著論文	質問紙法;	GHQ (精神	ヒト; 成人		キャリアと看護研究(1884-6947)8巻1号 Page3-13(2018.10)
2.02E+09	0	0	0	0	lental healt	1	occupatio	lifestyle a	職業性スト	職業性スト	足立 勝宣(	日本精神科看護学術集会誌	原著論文/	質問紙法;		ヒト; 成人		日本精神科看護学術集会誌60巻2号 Page357-361(2018.10)
2.02E+09	0	0	0	0	k environm	1	engagement		ワーク・エ	ワーク・エ	足立 勝宣(	日本精神科看護学術集会誌	原著論文/	回帰分析;		ヒト; 成人		日本精神科看護学術集会誌60巻2号 Page362-366(2018.10)
2.02E+09	0	0	0	0	k environm	1	retention	turnover	女性看護師	本研究は、	吉村 千草(	日本医療・病院管理学会誌	原著論文	因子分析;		ヒト; 成人	10.11303/	日本医療・病院管理学会誌(1882-594X)55巻4号 Page173-183(2018.10)
2.02E+09	0	0	0	0	lental healt	1	occupatio	sleep	40～50歳	目的:日本	足利 一美(	人間ドック(1880-1021)33	原著論文	質問紙法;	労働時間	ヒト; 成人	10.11320/	人間ドック(1880-1021)33巻4号 Page595-601(2018.12)
2.02E+09	0	0	0	0	ed disease,	1	diet and n	locomo	壮・中年者	目的:壮・	脇本 敏裕(	人間ドック(1880-1021)33	原著論文/	*筋骨格系	実態調査;	ヒト; 成人	10.11320/	人間ドック(1880-1021)33巻4号 Page602-608(2018.12)
2.02E+09	0	0	0	0	lental healt	1	occupational stress		私立総合大	女性看護師	吉岡 希(帝	日本衛生学雑誌(0021-508	原著論文/	看護師; *労	労働時間	ヒト; 成人	10.1265/jj	日本衛生学雑誌(0021-5082)73巻3号 Page388-394(2018.09)
2.02E+09	0	0	0	0	lental healt	1	occupatio	biomarker	労働者の前	【目的】本	佐藤 公子(	日本未病システム学会雑誌	原著論文/	L-Lactate	*保健指導	ヒト; 成人		日本未病システム学会雑誌(1347-5541)24巻3号 Page12-21(2018.12)
2.02E+09	0	0	0	0	egnancy ar	0	work, nutr	work, nutr	妊娠経過に	本研究は、	中村 康香(	母性衛生(0388-1512)59巻	原著論文/	*運動活性	*運動量; 妊	ヒト; 妊娠		母性衛生(0388-1512)59巻4号 Page712-718(2019.01)
2.02E+09	0	0	0	0	rk and bala	6	rework	rework	初産婦にJ	本研究の目	稲 桂(千葉	母性衛生(0388-1512)59巻	原著論文	*育児; 経産		ヒト; 成人		母性衛生(0388-1512)59巻4号 Page745-753(2019.01)
2.02E+09	0	0	0	0	lental healt	0	occupational stress		病院薬剤師	【目的】病	井奈波 良一	日本職業・災害医学会会誌	原著論文/	看護師; 分	医薬情報担	ヒト; 成人		日本職業・災害医学会会誌(1345-2592)66巻6号 Page418-423(2018.11)
2.02E+09	0	0	0	0	opause, an	0	menopaus	実態・意識	労災病院に	全国の労災	宮内 文久(	日本職業・災害医学会会誌	原著論文	加齢; 看護	受療行動	ヒト; 成人		日本職業・災害医学会会誌(1345-2592)66巻6号 Page486-491(2018.11)
2.02E+09	0	0	0	0	related dis	1	physical s	pretibial e	女性看護職	病棟で勤務	城賀本 晶	健康支援(1345-0174)21巻	原著論文/	質問紙法;		ヒト; 成人		健康支援(1345-0174)21巻1号 Page1-9(2019.02)
2.02E+09	0	0	0	0	n/ satisfac	1	satisfactic	医療従事者	女性看護師	女性看護師	井奈波 良一	日本健康医学会雑誌(1343-	原著論文/	看護師; *ま		ヒト; 成人	10.20685/	日本健康医学会雑誌(1343-0025)27巻4号 Page294-302(2019.01)
2.02E+09	0	0	0	0	opause, an	6	menopaus	職場支援・	更年期にあ	目的:勤務	酒井 洋子(	医療の質・安全学会誌(188	原著論文	看護師; 更		ヒト; 中年		医療の質・安全学会誌(1881-3658)14巻1号 Page11-22(2019.01)
2.02E+09	0	0	0	0	opause, an	4	Breast_ca	乳がん	乳がん早期	＜文献概要	鈴木 久美(	日本がん看護学会誌(0914-	原著論文	癌看護; *早	受診率; 受	ヒト; 成人		日本がん看護学会誌(0914-6423)32巻 Page12-22(2018.01)
2.02E+09	0	0	0	0	lental healt	1	stress management		女性看護師	【目的】女	井奈波 良一	日本職業・災害医学会会誌	原著論文/	*看護師; 労		ヒト; 成人		日本職業・災害医学会会誌(1345-2592)67巻1号 Page8-14(2019.01)
2.02E+09	0	0	0	0	opause, an	1	menopaus	実態・意識	中年勤労女	(目的)女性	茂木 順子(	日本職業・災害医学会会誌	原著論文/	血液化学分		ヒト; 成人		日本職業・災害医学会会誌(1345-2592)67巻1号 Page22-29(2019.01)
2.02E+09	0	0	0	0	lental healt	0	occupatio	fatigue	特別養護老	本研究は、	渡邊 美幸(	福祉と看護の研究誌(2433-	原著論文/	性因子; *特	自己評価ま	ヒト; 成人		福祉と看護の研究誌(2433-6947)5号 Page50-59(2018.12)
2.02E+09	0	0	0	0	rk and bala	6	WFC	WFC	未就学児を	わが国では	荻 邑子(京	人間看護学研究(1349-272	原著論文	*育児; 看護		ヒト; 成人		人間看護学研究(1349-2721)17号 Page67-76(2019.03)
2.02E+09	0	0	0	0	lental healt	1	occupatio	predictor	在宅認知症	目的:本研究	齋藤 嘉宏(	日本看護研究学会雑誌(028	原著論文/	因子分析;	介護負担(	ヒト; 成人		日本看護研究学会雑誌(0285-9262)42巻1号 Page87-98(2019.04)
2.02E+09	0	0	0	0	egnancy ar	1	safety, wc	safety, wc	【労働災害	本研究では	山口 さち	労働安全衛生研究(1882-68	原著論文/	質問紙法;		ヒト	10.2486/jc	労働安全衛生研究(1882-6822)12巻1号 Page3-12(2019.02)
2.02E+09	0	0	0	0	egnancy ar	6	balance w	balance w	就業継続を	目的:就業	永田 智子(	母性衛生(0388-1512)60巻	原著論文	葛藤; 育児		ヒト; 成人		母性衛生(0388-1512)60巻1号 Page110-117(2019.04)
2.02E+09	0	0	0	0	rk and bala	1	WFC	caregiver	中高年女性	本研究は、	菊澤 佐江	老年社会科学(0388-2446)	原著論文	*在宅介護	労働時間	ヒト; 中年	10.34393/	老年社会科学(0388-2446)41巻1号 Page9-17(2019.04)
2.02E+09	0	0	0	0	ed disease,	1	diet and n	diet and n	労働者にお	日本人労働	高島 直子(	地域環境保健福祉研究(134	原著論文	栄養価; エ		ヒト; 成人		地域環境保健福祉研究(1343-9286)21巻1号 Page41-46(2018.12)
2.02E+09	0	0	0	0	opause, an	1	menopaus	work enga	キャリア発	【目的】非	兼宗 美幸(	更年期と加齢のヘルスケア	原著論文/	質問紙法;		ヒト; 成人		更年期と加齢のヘルスケア(1347-801X)18巻1号 Page19-30(2019.06)
2.02E+09	0	0	0	0	rk and bala	0	WFC	child-rear	育児中の作	育児中・非	片岡 聡子(	作業療法(0289-4920)38巻	原著論文/	質問紙法;	実態調査	ヒト; 成人		作業療法(0289-4920)38巻3号 Page285-293(2019.06)
2.02E+09	0	0	0	0	rk and bala	1	rework	rework	復職準備性	復職準備性	堀井 清香(	精神神経学雑誌(0033-265	原著論文	うつ病(診断	レディネス	ヒト; 成人		精神神経学雑誌(0033-2658)121巻6号 Page445-456(2019.06)
2.02E+09	0	0	0	0	related dis	1	injury	injury	小売業勤労	【目的】本	原田 和弘(	日本転倒予防学会誌(2188-	原著論文/	質問紙法;		ヒト; 成人		日本転倒予防学会誌(2188-5702)6巻1号 Page25-34(2019.06)
2.02E+09	0	0	0	0	ed disease,	1	lifestyle	obesity	肥満にかか	目的 20代	千葉 綾乃(	肥満研究(1343-229X)25巻	原著論文/	危険因子;	労働時間	ヒト; 成人		肥満研究(1343-229X)25巻1号 Page38-51(2019.04)
2.02E+09	0	0	0	0	lental healt	0	occupatio	JOC	女性病院看	【目的】女	井奈波 良一	日本職業・災害医学会会誌	原著論文/	看護師; *労		ヒト; 成人		日本職業・災害医学会会誌(1345-2592)67巻2号 Page81-86(2019.03)
2.02E+09	0	0	0	0	related dis	1	Musle ske	MusleSke	勤労者の肩	本研究は勤	加藤 剛平(	日本職業・災害医学会会誌	原著論文/	*労働衛生	筋量	ヒト; 青年		日本職業・災害医学会会誌(1345-2592)67巻2号 Page87-94(2019.03)
2.02E+09	0	0	0	0	opause, an	1	menopaus	stress	更年期勤労	勤労女性の	井谷 美幸(	日本職業・災害医学会会誌	原著論文/	コーネル医	*保健指導	ヒト; 成人		日本職業・災害医学会会誌(1345-2592)67巻2号 Page125-130(2019.03)
2.02E+09	0	0	0	0	lental healt	1	occupatio	sleep	大学病院に	＜文献概要	星野 清香(	日本看護医療学会雑誌(134	原著論文/	質問紙法;	実態調査	ヒト; 成人	10.11477/	日本看護医療学会雑誌(1345-2606)20巻2号 Page64-71(2018.12)
2.02E+09	0	0	0	0	egnancy ar	1	mental he	mental he	経産婦にお	目的:経産	絹川 朋美(	母性衛生(0388-1512)60巻	原著論文/	育児; *運動	感情プロフ	ヒト; 妊娠		母性衛生(0388-1512)60巻2号 Page386-394(2019.07)
2.02E+09	0	0	0	0	ed disease,	1	physical a	sedentary	労働者生活	本研究では	蘇 リナ(労	労働安全衛生研究(1882-68	原著論文/	質問紙法;	自己評価ま	ヒト; 成人	10.2486/jc	労働安全衛生研究(1882-6822)12巻3号 Page127-133(2019.09)
2.02E+09	0	0	0	0	egnancy ar	0	safety, wc	safety, wc	看護師にお	【目的】看	吉田 麻美(	日本産業看護学会誌(2188-	原著論文	看護師; 健	実態調査	ヒト; 妊娠		日本産業看護学会誌(2188-6377)6巻1号 Page8-15(2019.10)
2.02E+09	0	0	0	0	ed disease,	0	diet and n	sarcopeni	職場におけ	本学(大学)	田中 智美(	CAMPUS HEALTH(1341-4	原著論文/	質問紙法;	起居動作;	ヒト; 成人		CAMPUS HEALTH(1341-4313)56巻1号 Page216-218(2019.03)
2.02E+09	0	0	0	0	rk and bala	1	WFC	WLB	看護師を対	目的:看護	松尾 まき(	日本健康医学会雑誌(1343-	原著論文	葛藤; 因子		ヒト; 成人	10.20685/	日本健康医学会雑誌(1343-0025)28巻3号 Page323-334(2019.10)
2.02E+09	0	0	0	0	rk and bala	0	workstyle	workstyle	宮崎県にお	【はじめに	福本 周子(	臨床と理学療法(2188-550	原著論文	家族特性;	実態調査	ヒト; 成人		臨床と理学療法(2188-5508)6巻2号 Page34-43(2019.09)
2.02E+09	0	0	0	0	ed disease,	1	LRD	CVD	職場環境た	【目的】職	加藤 剛平(	日本職業・災害医学会会誌	原著論文/	回帰分析;		ヒト; 中年		日本職業・災害医学会会誌(1345-2592)67巻4号 Page317-324(2019.07)
2.02E+09	0	0	0	0	lental healt	1	occupational stress		職業性スト	本研究は、	米山 雅子(	心身健康科学(1882-6881)	原著論文/	質問紙法;		ヒト; 成人	10.11427/	心身健康科学(1882-6881)15巻2号 Page71-81(2019.10)
2.02E+09	0	0	0	0	n/ satisfac	1	education	health prc	職業性スト	本研究は、	福永 ひと	心身健康科学(1882-6881)	原著論文	自己概念;		ヒト; 成人	10.11427/	心身健康科学(1882-6881)15巻2号 Page82-93(2019.10)
2.02E+09	0	0	0	0	opause, an	0	menopaus	sleep	更年期女性	【目的】女	高島 えり	更年期と加齢のヘルスケア	原著論文	質問紙法;		ヒト; 中年		更年期と加齢のヘルスケア(1347-801X)18巻2号 Page204-213(2019.12)
2.02E+09	0	0	0	0	opause, an	0	menopaus	職場支援・	働く更年期	【目的】	峯村 昌子(	更年期と加齢のヘルスケア	原著論文	質問紙法;		ヒト; 成人		更年期と加齢のヘルスケア(1347-801X)18巻2号 Page214-218(2019.12)
2.02E+09	0	0	0	0	ed disease,	1	physical a	sedentary	座位行動・	デスクワー	小野寺 由	体力科学(0039-906X)68巻	原著論文	*運動活性	オフィス	ヒト; 成人	10.7600/js	体力科学(0039-906X)68巻4号 Page261-268(2019.08)
2.02E+09	0	0	0	0	related dis	0	Musle ske	MusleSke	女性看護・	介護職の腰	木林 勉(金	性とこころ(1884-7005)10	原著論文	質問紙法;	介護・福祉	ヒト; 成人		性とこころ(1884-7005)10巻1号 Page155-160(2019.03)
2.02E+09	0	0	0	0	n/ satisfac	1	education	health prc	院内教育に	本研究の目	星野 純子(	日本看護管理学会誌(1347-	原著論文	質問紙法;		ヒト; 成人	10.19012/	日本看護管理学会誌(1347-0140)23巻1号 Page128-139(2019.12)
2.02E+09	0	0	0	0	rk and bala	1	WFC	child-rear	子育て期の	目的 子育	岩佐 一(福	日本公衆衛生雑誌(0546-17	原著論文	*育児; 因子		ヒト; 成人	10.11236/	日本公衆衛生雑誌(0546-1766)67巻1号 Page42-50(2020.01)
2.02E+09	0	0	0	0	rk and bala	0	WFC	child-rear	子育て女性	【目的】働	大平 肇子(	母性衛生(0388-1512)60巻	原著論文/	*育児; *労	家事; 生活	ヒト; 成人		母性衛生(0388-1512)60巻4号 Page614-624(2020.01)
2.02E+09	0	0	0	0	egnancy ar	1	safety, wc	safety, wc	女性看護職	看護職が自	摺元 歩美(	母性衛生(0388-1512)60巻	原著論文/	*労働衛生	労働時間;	ヒト; 成人		母性衛生(0388-1512)60巻4号 Page634-643(2020.01)
2.02E+09	0	0	0	0	lental healt	1	occupatio	sleep	運動習慣お	筆者らは、	足立 勝宣(	日本精神科看護学術集会誌	原著論文/	質問紙法;		ヒト; 成人		日本精神科看護学術集会誌61巻2号 Page334-338(2020.02)
2.02E+09	0	0	0	0	ed disease,	1	physical a	sedentary	専業主婦と	専業主婦と	北川 智美(	Journal of Rehabilitation a	原著論文	運動活性;	SF-36	ヒト; 成人		Journal of Rehabilitation and Health Sciences(1348-6756)15巻 Page31-39(2018.12)
2.02E+09	0	0	0	0	opause, an	1	menopaus	predictor	閉経前後の	(目的)女性	茂木 順子(	日本職業・災害医学会会誌	原著論文	Insulin-Lil	*生活指導	ヒト; 成人		日本職業・災害医学会会誌(1345-2592)67巻6号 Page501-509(2019.11)
2.02E+09	0	0	0	0	rk and bala	0	workstyle	workstyle	回復期リハ		関根 聡子(	ヘルスプロモーション・リ	原著論文/	質問紙法;	リハビリテ	ヒト; 成人		ヘルスプロモーション・リサーチ11巻1号 Page28-38(2018.12)
2.02E+09	0	0	0	0	egnancy ar	1	safety, wc	safety, wc	妊娠就業者	先行研究(	前谷津 文	JART: 日本診療放射線技師	原著論文	質問紙法;	X線テレビ	ヒト; 妊娠		JART: 日本診療放射線技師会誌(2187-2538)67巻3号 Page238-247(2020.03)
2.02E+09	0	0	0	0	related dis	0	shift	shift	夜勤業務を	【目的】彩	宮下 みゆ	日本職業・災害医学会会誌	原著論文	血液化学分	実態調査	ヒト; 成人		日本職業・災害医学会会誌(1345-2592)68巻1号 Page23-30(2020.01)
2.02E+09	0	0	0	0	related dis	0	shift	shift	毛髪に含ま	毛髪に含ま	宮内 文久(	日本職業・災害医学会会誌	原著論文	*Hydrocor		ヒト; 成人		日本職業・災害医学会会誌(1345-2592)68巻1号 Page71-76(2020.01)
2.02E+09	0	0	0	0	n/ satisfac	1	education	health prc	スタッフ看	スタッフ看	佐々木 美	医療の広場60巻2号 Page1	原著論文	看護師; 労		ヒト; 成人		医療の広場60巻2号 Page15-19(2020.02)
2.02E+09	0	0	0	0	n/ satisfac	4	education	health prc	院内研修会	ディエスカ	三宅 健太(	日本精神科看護学術集会誌	原著論文/	*保健医療		ヒト; 男; 女		日本精神科看護学術集会誌62巻1号 Page42-43(2019.06)
2.02E+09	0	0	0	0	nt and disc	0	violence	患者からの	精神科病棟	精神科病棟	西谷 理香(	日本精神科看護学術集会誌	原著論文	精神看護;	実態調査	ヒト; 男; 女		日本精神科看護学術集会誌62巻1号 Page142-143(2019.06)
2.02E+09	0	0	0	0	ed disease,	1	physical a	sedentary	仕事中の強	目的:今日	福島 教照(	産業衛生学雑誌(1341-0725	原著論文	質問紙法;	工場; *労働	ヒト; 成人	10.1539/s	産業衛生学雑誌(1341-0725)62巻2号 Page61-71(2020.03)
2.02E+09	0	0	0	0	related dis	0	shift	shift	12時間の二	目的:12時	折山 早苗(	日本看護科学会誌(0287-53	原著論文	質問紙法;	*眠気; 周波	ヒト; 成人	10.5630/ja	日本看護科学会誌(0287-5330)39巻 Page19-28(2019.12)
2.02E+09	0	0	0	0	related dis	1	shift	shift	変則夜間勤	蛋白尿の発	林 恭子(愛	生物試料分析(0913-3763)	原著論文/	看護師; *栄	Fatty Acid	ヒト; 成人		生物試料分析(0913-3763)43巻2号 Page140-147(2020.03)
2.02E+09	0	0	0	0	opause, an	0	Breast_ca	乳がん	治療と仕事	【目的】治	竹村 淳也(	保健医療学雑誌(2185-039	原著論文	質問紙法;		ヒト; 女	10.15563/	保健医療学雑誌(2185-0399)11巻1号 Page48-52(2020.04)

2.02E+09	0	0	0	0	ed disease,	0	oral healtl	oral	レセプト・	本研究は全	上根 昌子(	口腔衛生学会雑誌(0023-28	原著論文	医療費; *5	*受療行動	ヒト; 成人	10.5834/jo	口腔衛生学会雑誌(0023-2831)70巻2号 Page94-102(2020.04)
2.02E+09	0	0	0	0	egnancy ar	1	balance w	balance w	妊娠中の女	目的:妊娠	冨樫 千秋(	日本健康医学会雑誌(1343-	原著論文	質問紙法;		ヒト; 妊娠	10.20685/	日本健康医学会雑誌(1343-0025)29巻1号 Page9-16(2020.04)
2.02E+09	0	0	0	0	k environm	1	engagement		女性病院看	女性看護師	井奈波 良	日本健康医学会雑誌(1343-	原著論文/	看護師; *職		ヒト; 成人	10.20685/	日本健康医学会雑誌(1343-0025)29巻1号 Page39-45(2020.04)
2.02E+09	0	0	0	0	nt and disc	1	Maternity	Maternity	就労妊婦に	本研究は就	池田 智子(	母性衛生(0388-1512)61巻	原著論文/	経産回数;	実態調査	ヒト; 妊娠		母性衛生(0388-1512)61巻1号 Page19-27(2020.04)
2.02E+09	0	0	0	0	nt and disc	0	Maternity	Maternity	就労妊婦の	本研究の目	下山 博子(	母性衛生(0388-1512)61巻	原著論文/	因子分析;		ヒト; 成人		母性衛生(0388-1512)61巻1号 Page123-132(2020.04)
2.02E+09	0	0	0	0	egnancy ar	1	mental he	mental he	子育て期の	目的 子育て	岩佐 一(福	厚生の指標(0452-6104)67	原著論文/	質問紙法;		ヒト; 成人		厚生の指標(0452-6104)67巻6号 Page8-14(2020.06)
2.02E+09	0	0	0	0	k environm	1	retention	turnover	働く女性の		金井 篤子(	心理学研究(0021-5236)65	原著論文	質問紙法;		ヒト; 青年	10.4992/jj	心理学研究(0021-5236)65巻2号 Page112-120(1994.06)
2.02E+09	0	0	0	0	egnancy ar	4	work, nutr	work, nutr	成熟期就労	目的:成熟期	名草 みど	日本健康教育学会誌(1340-	原著論文	*健康教育	食物摂取頻	ヒト; 成人	10.11260/	日本健康教育学会誌(1340-2560)28巻2号 Page81-91(2020.05)
2.02E+09	0	0	0	0	rk and bala	1	WFC	caregiver	親の介護を	目的:親の介	高林 知佳	日本地域看護学会誌(1346-	原著論文	回帰分析;	*介護負担	ヒト; 成人	10.20746/	日本地域看護学会誌(1346-9657)23巻1号 Page13-22(2020.04)
2.02E+09	0	0	0	0	k environm	1	retention	turnover	看護専門学	看護専門学	井ノ上 ル	看護実践の科学(0385-428	原著論文	質問紙法;	*価値観	ヒト; 成人		看護実践の科学(0385-4280)45巻8号 Page78-92(2020.08)
2.02E+09	0	0	0	0	rk and bala	6	WFC	child-rear	小学1・2年	本研究は、	石井 美里(	母性衛生(0388-1512)61巻	原著論文	育児; *労働		ヒト; 成人		母性衛生(0388-1512)61巻2号 Page280-288(2020.07)
2.02E+09	0	0	0	0	ed disease,	1	lifestyle	others	高ストレス	目的:近年、	西山 里枝(	人間ドック(1880-1021)35	原著論文/	質問紙法;		ヒト; 成人	10.11320/	人間ドック(1880-1021)35巻2号 Page185-193(2020.09)
2.02E+09	0	0	0	0	egnancy ar	1	fertility		不妊治療を	【研究の目	冨田 志織,	看護科学研究(2424-0052)	原著論文/	感情; *心理	実態調査	ヒト; 成人	10.20705/	看護科学研究(2424-0052)18巻1号 Page1-11(2020.06)
2.02E+09	0	0	0	0	related dis	1	Organic	chemical	ワイン製造	歯の酸蝕症	鈴木 誠太	労働安全衛生研究(1882-68	原著論文/	*う蝕(化学	曝露評価;	ヒト; 成人	10.2486/jo	労働安全衛生研究(1882-6822)13巻2号 Page167-171(2020.09)
2.02E+09	0	0	0	0	lental healt	1	occupatio	sleep	女性病院看	目的:女性病	黒岩 千翔(	産業精神保健(1340-2862)	原著論文	回帰分析;	*眠気(診断	ヒト; 成人		産業精神保健(1340-2862)28巻3号 Page236-247(2020.10)
2.02E+09	0	0	0	0	ed disease,	1	lifestyle	weight	労働者のイ	目的 近年,	中村 優(島	厚生の指標(0452-6104)67	原著論文	性因子; ラ		ヒト; 成人		厚生の指標(0452-6104)67巻11号 Page29-34(2020.09)
2.02E+09	0	0	0	0	egnancy ar	0	mental he	mental he	日本人妊婦	現代におけ	牛越 幸子(	睡眠医療(1882-2096)14巻	原著論文/	覚醒; *睡眠	実態調査	ヒト; 妊娠		睡眠医療(1882-2096)14巻3号 Page319-324(2020.09)
2.02E+09	0	0	0	0	ed disease,	0	other	checkup	職員健康診	本学では毎	鵜川 奈津	CAMPUS HEALTH(1341-	原著論文	時間; *集団	受診率	ヒト; 男; 女		CAMPUS HEALTH(1341-4313)57巻1号 Page99-101(2020.03)
2.02E+09	0	0	0	0	lental healt	1	occupatio	sleep	女性教職員	日本人の睡	松本 由佳(	CAMPUS HEALTH(1341-	原著論文/	育児; 催眠	実態調査	ヒト; 成人		CAMPUS HEALTH(1341-4313)57巻1号 Page166-168(2020.03)
2.02E+09	0	0	0	0	opause, an	0	menstratio	月経	福祉の現場	月経は、女	甲斐村 美	地域ケアリング(1345-012	原著論文/	月経困難症		ヒト; 成人		地域ケアリング(1345-0123)22巻14号 Page60-62(2020.12)
2.02E+09	0	0	0	0	rk and bala	0	rework	rework	同一出生世	女性の労働	安藤 雄一(	ヘルスサイエンス・ヘルス	原著論文	*歯科衛生		ヒト; 成人		ヘルスサイエンス・ヘルスケア(1881-1892)19巻1号 Page16-22(2019.09)
2.02E+09	0	0	0	0	opause, an	1	menstratio	月経	看護職の月	本研究の目	八巻 ちひ	日本母性看護学会誌(1345-	原著論文/	月経; *月経		ヒト; 成人		日本母性看護学会誌(1345-773X)21巻1号 Page19-28(2020.10)
2.02E+09	0	0	0	0	rk and bala	0	workstyle	workstyle	整形外科医	日本整形外	土屋 明大,	日本整形外科学会雑誌(002	原著論文	質問紙法;	*労働時間	ヒト; 男; 女		日本整形外科学会雑誌(0021-5325)94巻10号 Page907-917(2020.10)
2.02E+09	0	0	0	0	n/ satisfac	1	career		育児期女性	本研究は育	飯田 麻衣	日本家政学会誌(0913-522	原著論文	質問紙法;		ヒト; 成人	10.11428/	日本家政学会誌(0913-5227)71巻12号 Page775-782(2020.12)
2.02E+09	0	0	0	0	egnancy ar	1	safety, wc	safety, wc	有職女性の	目的 日本	木島 楓(東	厚生の指標(0452-6104)67	原著論文	断面研究;	実態調査;	ヒト; 成人		厚生の指標(0452-6104)67巻12号 Page7-12(2020.10)
2.02E+09	0	0	0	0	lental healt	1	stress ma	laguhing	笑いの頻度	職域におけ	足立 勝宣(	日本精神科看護学術集会誌	原著論文/	質問紙法;		ヒト; 成人		日本精神科看護学術集会誌62巻2号 Page9-13(2020.10)
2.02E+09	0	0	0	0	lental healt	0	stress ma	stress che	ストレスチ	ストレスチ	足立 勝宣(	日本精神科看護学術集会誌	原著論文	労働衛生;		ヒト; 成人		日本精神科看護学術集会誌62巻2号 Page19-23(2020.10)
2.02E+09	0	0	0	0	lental healt	1	stress management		笑いとワー	笑いとワー	足立 勝宣(	日本精神科看護学術集会誌	原著論文/	労働衛生;		ヒト; 成人		日本精神科看護学術集会誌62巻2号 Page24-28(2020.10)
2.02E+09	0	0	0	0	rk and bala	1	WFC	WFC	中小企業雇	目的 本研究	熊谷 麻紀(	日本公衆衛生雑誌(0546-17	原著論文/	*葛藤; *家	労働時間	ヒト; 成人	10.11236/	日本公衆衛生雑誌(0546-1766)67巻12号 Page850-859(2020.12)
2.02E+09	0	0	0	0	egnancy ar	3	stat	stat	日本人女性	目的 本研究	奥井 佑(九	日本公衆衛生雑誌(0546-17	原著論文/	*結婚; コホ		ヒト; 成人	10.11236/	日本公衆衛生雑誌(0546-1766)67巻12号 Page892-903(2020.12)
2.02E+09	0	0	0	0	rk and bala	6	workstyle	workstyle	女性テレワ	目的:女性の	大平 肇子(	母性衛生(0388-1512)61巻	原著論文	*労働衛生	内容分析;	ヒト; 成人		母性衛生(0388-1512)61巻4号 Page525-533(2021.01)
2.02E+09	0	0	0	0	egnancy ar	6	BF	BF	働く女性が	【目的】働	梶井 敬子(	母性衛生(0388-1512)61巻	原著論文	母子関係;	信念	ヒト; 成人		母性衛生(0388-1512)61巻4号 Page649-657(2021.01)
2.02E+09	0	0	0	0	egnancy ar	1	fertility	stat	都道府県別	目的 わが国	田辺 和俊(	厚生の指標(0452-6104)67	原著論文	回帰分析;	少子化; 地	ヒト; 成人		厚生の指標(0452-6104)67巻13号 Page15-21(2020.11)
2.02E+09	0	0	0	0	related dis	1	other	scale deve	リハビリテ	目的:作業療	三宅 優紀(	日本予防医学会雑誌(1881-	原著論文	因子分析;	項目反応理	ヒト; 成人		日本予防医学会雑誌(1881-4271)14巻1号 Page7-13(2020.02)
2.02E+09	0	0	0	0	egnancy ar	0	safety, wc	safety, wc	妊娠中のM	本研究では	山口 さち	労働安全衛生総合研究所特	原著論文	質問紙法;		ヒト		労働安全衛生総合研究所特別研究報告(1882-8329)50号 Page169-175(2020.11)
2.02E+09	0	0	0	0	egnancy ar	1	safety, wc	safety, wc	MRI検査業	本研究では	山口 さち	労働安全衛生総合研究所特	原著論文	質問紙法;		ヒト; 成人		労働安全衛生総合研究所特別研究報告(1882-8329)50号 Page177-185(2020.11)
2.02E+09	0	0	0	0	related dis	0	shift	shift	交替勤務が	【目的】交	足立 勝宣(	日本職業・災害医学会会誌	原著論文/	労働衛生;		ヒト; 成人		日本職業・災害医学会会誌(1345-2592)69巻2号 Page59-64(2021.03)
2.02E+09	0	0	0	0	related dis	0	Organic	hormaldel	ホルマリン	ホルマリン	関口 久男(	埼臨技会誌(0287-1823)67	原著論文	Methanol;	Methylhip	ヒト		埼臨技会誌(0287-1823)67巻3号 Page111-119(2020.03)
2.02E+09	0	0	0	0	related dis	4	shift	shift	夜勤のある		三好 陽子(	女性健康科学研究会受賞研	原著論文	看護師; *		ヒト; 成人		女性健康科学研究会受賞研究報告集8巻1号 Page38-42(2019.04)
2.02E+09	0	0	0	0	lental healt	0	occupatio	SOC	女性看護師	目的:女性看	櫻沢 早人	日本看護科学会誌(0287-53	原著論文/	回帰分析;		ヒト; 成人	10.5630/ja	日本看護科学会誌(0287-5330)40巻 Page553-561(2020.12)
2.02E+09	0	0	0	0	rk and bala	0	WFC	child-rear	未就学児育	2019年1月	宇井 秋子(	医療の広場61巻3号 Page1	原著論文	葛藤; *育児		ヒト; 成人		医療の広場61巻3号 Page16-21(2021.03)
2.02E+09	0	0	0	0	k environm	1	retention	turnover	病児保育施	本研究の目	中村 幸代(	日本健康医学会雑誌(1343-	原著論文/	育児; 看護		ヒト; 成人	10.20685/	日本健康医学会雑誌(1343-0025)30巻1号 Page55-64(2021.04)
2.02E+09	0	0	0	0	lental healt	1	overtime a	overtime c	宮城県教職	【背景】近	宗像 正徳(	日本職業・災害医学会会誌	原著論文/	質問紙法;	自己評価式	ヒト; 成人		日本職業・災害医学会会誌(1345-2592)69巻3号 Page127-135(2021.05)
2.02E+09	0	0	0	0	k environm	1	engagement		クリティカ	本研究の目	渡邉 成美(	日本クリティカルケア看護	原著論文	回帰分析;		ヒト; 成人	10.11153/	日本クリティカルケア看護学会誌(1880-8913)16巻 Page85-93(2020.03)
2.02E+09	0	0	0	0	related dis	0	Musle ske	MusleSke	フォトグラ	【目的】フ	塩崎 郁哉(	全日本鍼灸学会雑誌(0285-	原著論文/	質問紙法;	圧痛(治療)	ヒト; 成人	10.3777/jj	全日本鍼灸学会雑誌(0285-9955)71巻2号 Page107-115(2021.05)
2.02E+09	0	0	0	0	rk and bala	1	rework	rework	職場の問題	本研究の目	渡邉 明寿	認知療法研究(1883-2296)	原著論文	質問紙法;		ヒト; 成人		認知療法研究(1883-2296)14巻2号 Page195-204(2021.06)
2.02E+09	0	0	0	0	k environm	0	engagement		就労初妊婦	女性が妊娠	中村 康香(	日本母性看護学会誌(1345-	原著論文/	*労働衛生		ヒト; 妊娠	10.32305/	日本母性看護学会誌(1345-773X)21巻2号 Page19-26(2021.03)
2.02E+09	0	0	0	0	egnancy ar	0	fertility		不妊治療を	2020年8月	松崎 政代(	医療の広場61巻6号 Page1	原著論文	回帰分析;	実態調査	ヒト; 成人		医療の広場61巻6号 Page17-25(2021.06)
2.02E+09	0	0	0	0	rk and bala	6	WFC	child-rear	産業看護職	子育て中の	中村 侑加(	ヘルスプロモーション・リ	原著論文	*育児; 労働		ヒト; 成人		ヘルスプロモーション・リサーチ13巻1号 Page22-30(2020.12)
2.02E+09	0	0	0	0	k environm	6	retention	turnover	介護職にお	目的 国内で	富永 真己(	日本公衆衛生雑誌(0546-17	原著論文	専門職間人	介護者-患者	ヒト; 成人	10.11236/	日本公衆衛生雑誌(0546-1766)68巻7号 Page468-476(2021.07)
2.02E+09	0	0	0	0	lental healt	0	occupational stress		女性病院看	女性看護師	井奈波 良	日本健康医学会雑誌(1343-	原著論文	看護師; *		ヒト; 成人	10.20685/	日本健康医学会雑誌(1343-0025)30巻2号 Page170-178(2021.07)
2.02E+09	0	0	0	0	related dis	0	Musle ske	MusleSke	病棟看護師	【目的】看	田上 裕記(	理学療法学(0289-3770)48	原著論文	*看護師; *		ヒト; 成人		理学療法学(0289-3770)48巻4号 Page413-419(2021.08)
2.02E+09	0	0	0	0	lental healt	1	occupatio	sleep	公立小学校	目的 わが国	堀 大介(筑	厚生の指標(0452-6104)68	原著論文/	*学校; 労働	実態調査;	ヒト; 成人		厚生の指標(0452-6104)68巻6号 Page14-23(2021.06)
2.02E+09	0	0	0	0	ed disease,	0	lifestyle	smoke	職業別喫煙	目的 わが国	田中 宏和(	日本公衆衛生雑誌(0546-17	原著論文/	*タバコ喫		ヒト; 成人	10.11236/	日本公衆衛生雑誌(0546-1766)68巻6号 Page433-443(2021.06)
2.02E+09	0	0	0	0	n/ satisfac	1	education	health prc	新潟県にお	目的 近年,	諸橋 麻紀,	日本助産学会誌(0917-635	原著論文	質問紙法;	家庭訪問;	ヒト; 成人	10.3418/jj	日本助産学会誌(0917-6357)35巻1号 Page72-87(2021.06)
2.02E+09	0	0	0	0	rk and bala	0	rework	rework	看護実践上	看護職は女	神戸 美輪	日本医学看護学教育学会誌	原著論文	*看護; 看護	*看護実践	ヒト; 成人		日本医学看護学教育学会誌29-3号 Page22-29(2021.03)
2.02E+09	0	0	0	0	egnancy ar	6	balance w	balance w	有職高年初	本研究は、	戸村 佳美(	母性衛生(0388-1512)62巻	原著論文	*経産回数		ヒト; 妊娠		母性衛生(0388-1512)62巻2号 Page390-398(2021.07)
2.02E+09	0	0	0	0	opause, an	0	menstratio	月経	就労する性	目的:就労す	生方 尚絵(	母性衛生(0388-1512)62巻	原著論文/	*月経困難		ヒト; 成人		母性衛生(0388-1512)62巻2号 Page436-443(2021.07)
2.02E+09	0	0	0	0	n/ satisfac	0	career	career,tea	福祉施設の	本研究は、	宮城 和美(	共創福祉15巻2号 Page9-2	原著論文	質問紙法;	*興味	ヒト; 成人		共創福祉15巻2号 Page9-20(2021.03)
2.02E+09	0	0	0	0	ed disease,	0	others	checkup	埼玉県在住	日本人女性	宮下 ルリ	日本女性医学学会雑誌(218	原著論文	食行動; 身	家事	ヒト; 中年		日本女性医学学会雑誌(2185-8861)28巻4号 Page554-562(2021.07)
2.02E+09	0	0	0	0	lental healt	0	occupatio	predictor	産業看護職	日本産業衛	田中 佳世	ストレス科学(1349-4813)	原著論文	*看護師; *		ヒト; 成人		ストレス科学(1349-4813)35巻4号 Page348-356(2021.06)
2.02E+09	0	0	0	0	rk and bala	1	WFC	child-rear	共働き世帯	目的 近年,	細川 陸也(	厚生の指標(0452-6104)68	原著論文	育児; 回帰		ヒト; 男; 女		厚生の指標(0452-6104)68巻11号 Page32-36(2021.09)
2.02E+09	0	0	0	0	k environm	1	engagement		製造業の労	笑いとワー	足立 勝宣(	日本精神科看護学術集会誌	原著論文/	質問紙法;		ヒト; 成人		日本精神科看護学術集会誌63巻2号 Page7-11(2021.10)
2.02E+09	0	0	0	0	k environm	1	engagement		ワーク・コ	ワーク・コ	足立 勝宣(	日本精神科看護学術集会誌	原著論文/	質問紙法;		ヒト; 成人		日本精神科看護学術集会誌63巻2号 Page12-16(2021.10)
2.02E+09	0	0	0	0	k environm	1	engagement		既存データ	目的:40歳	仲 文子(旭	日本地域看護学会誌(1346-	原著論文/	質問紙法;		ヒト; 成人	10.20746/	日本地域看護学会誌(1346-9657)24巻2号 Page67-75(2021.08)
2.02E+09	0	0	0	0	related dis	4	Musle ske	MusleSke	看護職員の	看護職員の	田巻 洋子(	日本精神科看護学術集会誌	原著論文	*精神看護	DVD (メテ	ヒト; 成人		日本精神科看護学術集会誌64巻1号 Page406-407(2021.06)
2.02E+09	0	0	0	0	k environm	1	engagement		働く母親の	目的:働く	伊草 綾香(	日本保健福祉学会誌(1340-	原著論文	*労働衛生		ヒト; 成人		日本保健福祉学会誌(1340-8194)28巻1号 Page15-25(2021.12)
2.02E+09	0	0	0	0	opause, an	6	Repro_ca	婦人科がん	婦人科がん	本研究の目	木全 明子(	日本がん看護学会誌(0914-	原著論文	癌看護; 自	内容分析	ヒト; 成人		日本がん看護学会誌(0914-6423)35巻 Page261-272(2021.09)
2.02E+09	0	0	0	0	rk and bala	1	WFC	WFC	手術室に勤	手術室看護	有本 信江(	日本健康学会誌(2432-671	原著論文	回帰分析;	GHQ (精神	ヒト; 成人	10.3861/k	日本健康学会誌(2432-6712)87巻5号 Page229-236(2021.09)
2.02E+09	0	0	0	0	opause, an	0	Repro_ca	婦人科がん	全国大学女	全国国立大	河野 美江(	CAMPUS HEALTH(1341-	原著論文/	質問紙法;	受診率	ヒト; 女		CAMPUS HEALTH(1341-4313)58巻1号 Page64-66(2021.03)
2.02E+09	0	0	0	0	related dis	0	shift	shift	妊娠期にお	本研究の目	大重 育美(	日本看護管理学会誌(1347-	原著論文	*運動活性	自己評価式	ヒト; 妊娠	10.19012/	日本看護管理学会誌(1347-0140)25巻1号 Page262-271(2021.)
2.02E+09	0	0	0	0	k environm	6	retention	turnover	潜在看護師	【目的】看	高 綾子(東	日本看護管理学会誌(1347-	原著論文	*育児; 家族		ヒト; 成人		日本看護管理学会誌(1347-0140)25巻1号 Page151-160(2021.)
